# Highly stereoselective spirocyclopropanation of various diazooxindoles with olefins catalyzed using Ru(ii)-complex†

**DOI:** 10.1039/c8ra09212e

**Published:** 2018-11-28

**Authors:** Masaya Tone, Yoko Nakagawa, Soda Chanthamath, Ikuhide Fujisawa, Naofumi Nakayama, Hitoshi Goto, Kazutaka Shibatomi, Seiji Iwasa

**Affiliations:** Department of Environmental and Life Sciences, Toyohashi University of Technology Tempaku-cho Toyohashi 441-8580 Japan iwasa@ens.tut.ac.jp; CONFLEX Corporation, Shinagawa Center Building 6F, 3-23-17 Takanawa Minato-ku Tokyo 108-0074 Japan; Department of Computer Science and Engineering, Toyohashi University of Technology Tempaku-cho Toyohashi 441-8580 Japan

## Abstract

Optically active spirocyclopropyloxindole derivatives were efficiently synthesized from diazooxindoles and olefins in the presence of a Ru(ii)-Pheox catalyst. Among a series of Ru(ii)-Pheox catalysts, Ru(ii)-Pheox 6e was determined to be the best catalyst for spirocyclopropanation reactions of diazooxindoles with various olefins in high yields (up to 98%) with high diastereoselectivities (up to *trans*:*cis* = >99:1<) and enantioselectivities (up to 99% ee). Furthermore, as the first catalytic asymmetric synthesis, anti-HIV active candidate 4a and a bioactive compound of AMPK modulator 4c were easily synthesized from the corresponding diazooxindoles 1i and 1b, respectively, in high yields with high enantioselectivities (4a: 82% yield, 95% ee, 4b: 99% yield, 93% ee).

## Abstract 2

Optically active spirocyclopropyloxindole derivatives were efficiently synthesized from diazooxindoles and olefins in the presence of a Ru(ii)-Pheox catalyst.

## Graphical abstract

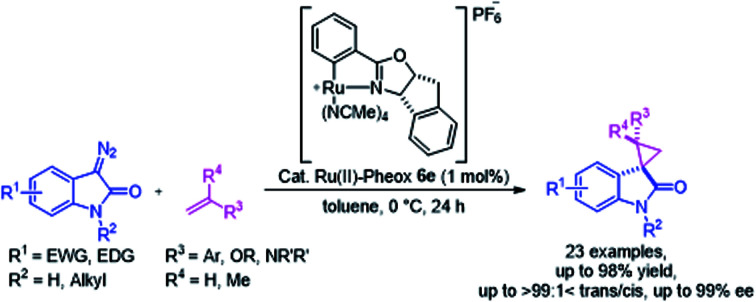

The discovery of a new class of optically active cyclopropyl oxindoles, as shown below (Fig. 1), has stimulated intensive research interest in the development of biologically active new organic molecules in both academia and industry.^1,2^ These compounds were observed to be pharmacologically important substrates with potentially interesting biological activities—e.g., potent HIV inhibitors,^3^ and anti-cancer^4^ and inotrope agents.^5^ Furthermore, chiral spirocyclopropyl oxindoles are useful key intermediates for the ring-expansion reaction of spirocyclopropane rings *via* a concerted mechanism in the presence of a Lewis acid catalyst.^6^ As for the synthetic methodology, the transition-metal-catalyzed asymmetric cyclopropanation of diazooxindoles with olefins has been reported, which is one of the most direct and efficient pathways for the synthesis of chiral spirocyclopropyl oxindoles. After the first report by Arai^7^ and Zhou^8^ independently with Rh and Hg catalysts, Ding and co-workers reported the asymmetric cyclopropanation of diazooxindoles with alkenes using a *C*_2_-symmetric spiroketal bisphosphine/Au(i) complex with good enantioselectivity.^9^ More recently, Xu and co-workers reported efficient dirhodium catalysts, which exhibited good to excellent enantioselectivity control with alkyl alkenes.^10^ Although effective catalysts and catalytic systems have been reported for the asymmetric cyclopropanation of diazooxindoles with olefins, the development of effective and powerful methodologies to prepare the unique structural motif of a spirocyclopropyloxindole remains an active research area.

**Fig. 1:**
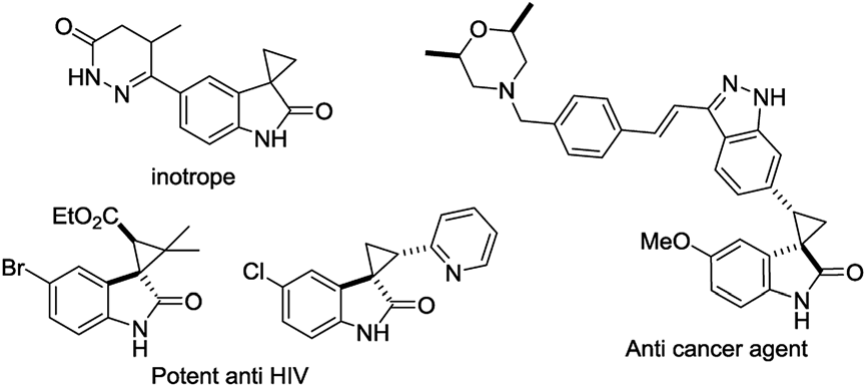
Bioactive spirocyclopropyl oxindoles.

We reported a series of Ru(ii) phenyloxazoline catalysts (Ru(ii)-Pheox) in 2010, which effectively promoted the asymmetric intra- and intermolecular carbene transfer reactions to olefins, C–H, N–H, and Si–H bonds in high yields with high enantioselectivities.^11^ Based on this study, we employed Ru(ii)-Pheox catalyst to synthesize a spirocyclopropanated oxindole from 5-bromo-3-methylene-indolin-2-one and diazoacetate.^12^ However, in this study, the reactivities and stereoselectivities were reported with moderate, or even high, diastereoselectivities. To improve the yield and enantioselectivity, we initially used Ru(ii)-Pheox for the reaction of a diazooxindole of an olefin (Fig. 2).

**Fig. 2:**
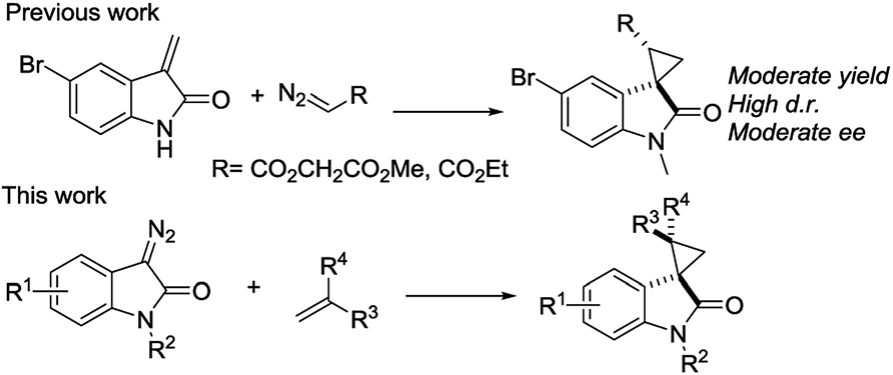
Synthetic strategies of optically active spirocyclopropyl oxindole.

First, we attempted a catalytic asymmetric cyclopropanation of diazooxindole 1a with styrene 2a catalyzed by authorized complexes such as 4^13^ and 5^14^ and a series of Ru(ii)-Pheox catalysts. The results are summarized in Table 1. The cyclopropanation with the commonly used catalysts 4 and 5 proceeded with low stereoselectivities (Table 1, entries 1 and 2). A series of Ru(ii)-Pheox catalysts was also tested for the spirocyclopropanation reaction. The reaction of diazooxindole 1a proceeded smoothly at room temperature to afford the desired cyclopropane product 3a in high yield with moderate enantioselectivity (Table 1, entry 3).

**Table 1:** Screening of various catalysts^a^

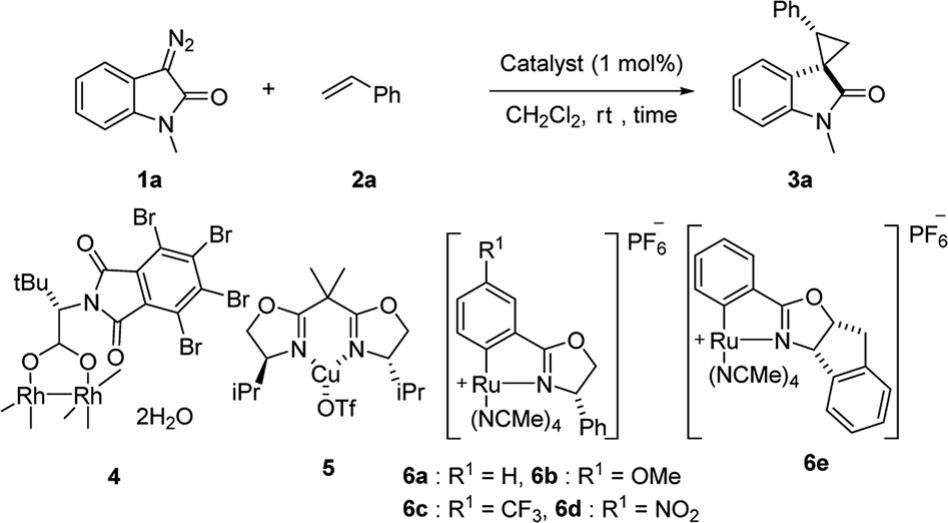					
Entry	Catalyst	Time [h]	Yield^b^ [%]	*trans*/*cis*^c^	ee^d^ [%]
1	4	10	90	69:31	−92
2	5	4 days	75	81:19	48
3	6a	6	95	95:5	50
4	6b	3 day	95	97:3	46
5	6c	24	93	95:5	49
6	6d	5	91	95:5	61
7	6e	6	91	94:6	92

a Reaction condition: catalyst (1 mol%) and styrene 2a (5.0 equiv., 1 mmol) were dissolved in CH_2_Cl_2_ (2.0 mL), and diazooxindole 1a (0.2 mmol) in CH_2_Cl_2_ (2 mL) was added.

b Isolated yield.

c Determined by NMR.

d Determined by chiral HPLC analysis.

To improve the enantioselectivity, the Ru(ii)-Pheox 6a catalyst was modified in terms of electron density on the aromatic ring connecting with Ru(ii) and a chiral environment (6b–e). Catalyst screening of various Ru(ii)-Pheox 6 catalysts is shown in Table 1 (entries 3–7). Consequently, we determined that the reactivity- and enantioselectivity-related electronic effect on the aromatic ring was slightly effective and increased the enantioselectivity to 61% ee with the use of Ru(ii)-Pheox 6d having an electron-withdrawing group such as NO_2_ (Table 1, entry 6). However, a much more effective catalyst, Ru(ii)-Pheox 6e^15^ having an indane-derived chiral environment, was observed to have higher activity and enantioselectivity than the other Ru(ii)-Pheox 6a catalysts, resulting in the corresponding product in high yield (98%) with excellent diastereoselectivity (94:6) and high enantioselectivity (92% ee) (Table 1, entry 7).

To improve the diastereoselectivity and enantioselectivity of this catalytic system further, we examined the cyclopropanation in various solvents (Table 2, entries 1–5). For all tested solvents, spirocyclopropanation proceeded smoothly in high yield but with differing diastereoselectivities and enantioselectivities. Toluene led to an improvement of trans-selectivity (*trans*/*cis* = 97:3) and enantioselectivity (94% ee). Toluene and dichloromethane were observed to be suitable solvents for the spirocyclopropanation of diazooxindole and olefins catalyzed by Ru(ii)-Pheox 6e. The effect of temperature on the reaction was also investigated (Table 2, entries 6–9), showing that enantioselectivity could be improved to 96% ee at a temperature of 0 °C (Table 2, entry 7). As such, 0 °C was determined to be the optimal temperature for this catalytic system.

**Table 2:** Optimization of reaction conditions^a^

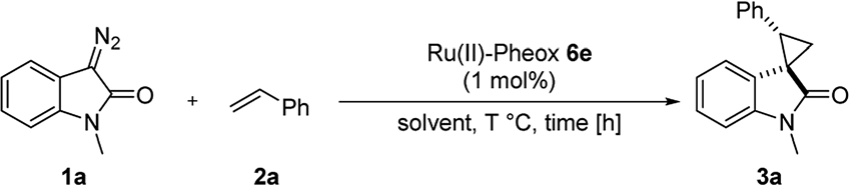						
Entry	Solvent	*T* [°C]	Time [h]	Yield^b^ [%]	*trans*/*cis*^c^	ee^d^ [%]
1	THF	rt	7	77	96:4	94
2	CH_2_Cl_2_	rt	6	91	94:6	92
3	Acetone	rt	6	87	94:6	91
4	CH_3_CN	rt	42	53	77:23	76
5^e^	Toluene : CH_2_Cl_2_	rt	6	97	91:9	95
6	Toluene	rt	24	95	97:3	94
7	Toluene	0	24	94	94:6	96
8	Toluene	−10	24	92	98:2	92
9	Toluene	−20	24	92	92:8	91

a Reaction condition: catalyst (1 mol%) and styrene 2a (5.0 equiv., 1 mmol) were dissolved in CH_2_Cl_2_ (2.0 mL), and diazooxindole 1a (0.2 mmol) in CH_2_Cl_2_ (2 mL) was added.

b Isolated yield.

c Determined by NMR.

d Determined by chiral HPLC analysis.

e Toluene : CH_2_Cl_2_ = 1 : 1.

Subsequently, we briefly examined the effect of N-substituted group under these optimized conditions. The result is summarized in Table 3. N-Alkyl diazooxindoles were subjected to these conditions with 2a. N-Benzyl group had some effects on the yield and resulted in the highest stereoselectivity in terms of both diastereoselectivity and enantioselectivity even far from the reactive site (Table 3, entry 4). When i-Pr group was used to substitute nitrogen, the yield was slightly decreased (Table 3, entry 3).

**Table 3:** Substituent effect on nitrogen of diazooxindole 1a–1d^a^

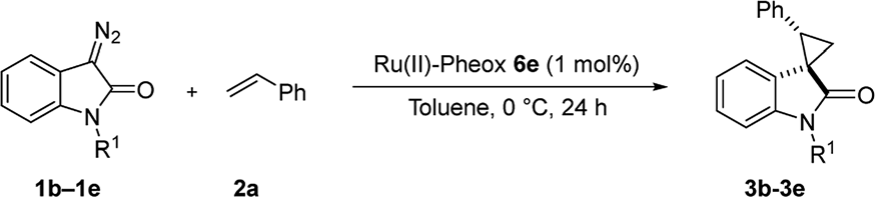					
Entry	R^1^	Product	Yield^b^ [%]	*trans*/*cis*^c^	ee^d^ [%]
1^e^	H	3b	98	93:7	92
2	Et	3c	94	97:3	97
3	iPr	3d	86	96:4	95
4	Bn	3e	97	>99:1<	98

a Reaction condition: catalyst (1 mol%) and styrene 2 (5.0 equiv., 1 mmol) were dissolved in CH_2_Cl_2_ (2.0 mL), and diazooxindole 1 (0.2 mmol) in CH_2_Cl_2_ (2 mL) was added.

b Isolated yield.

c Determined by NMR.

d Determined by chiral HPLC analysis.

e CH_2_Cl_2_ was used as the solvent.

We subsequently explored the scope and generality of the catalytic system (Table 4). Various vinyl arenes were reacted with diazooxindole 1a in the presence of Ru(ii)-Pheox 6e. Most of the vinyl arenes provided the corresponding spirocyclopropanation products in high yields (up to 98% yields) with high diastereoselectivities and enantioselectivities 93–99% ee (Table 4, entries 1–11). Germinal disubstituted olefin, α-methylstyrene 2g, also provided the product 3k in good yield with excellent diastereoselectivity and enantioselectivity (97% ee) (Table 4, entry 7). However, low enantioselectivity of 24% ee was observed in the case of cyclopropanation of 4-Me_2_N-substituted-styrene, which might be unstable during the purification following cyclopropane ring-opening reaction assisted by an electron-donating group (Table 4, entry 12).

**Table 4:** Spirocyclopropanation of diazooxindole 1a with styrene derivatives 2^a^

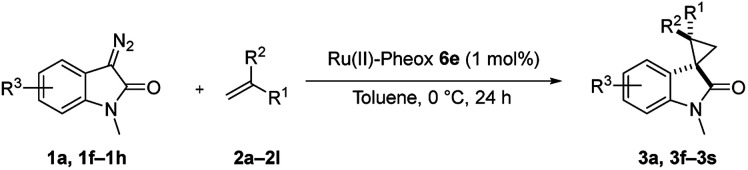							
Entry	R^1^	R^2^	R^3^	Product	Yield^b^ [%]	*trans*/*cis*^c^	ee^d^ [%]
1	Ph	H	H	3a	94	94:6	96
2	*o*-MeC_6_H_4_	H	H	3f	92	92:8	95
3	*m*-MeC_6_H_4_	H	H	3g	80	>99:1<	99
4	*p*-MeC_6_H_4_	H	H	3h	96	>99:1<	96
5	*p*-tBuC_6_H_4_	H	H	3i	97	>99:1<	95
6^e^	Np	H	H	3j	83	>99:1<	96
7^f^	Ph	Me	H	3k	93	98:2	97
8^e^	*p*-NO_2_C_6_H_4_	H	H	3l	85	96:4	94
9	*p*-BrC_6_H_4_	H	H	3m	98	96:4	94
10	*p*-ClC_6_H_4_	H	H	3n	98	96:4	93
11	*p*-OMeC_6_H_4_	H	H	3o	79	>99:1<	97
12	*p*-NMe_2_C_6_H_4_	H	H	3p	74	>99:1<	24
13	Ph	H	5-Br	3q	93	89:11	87
14	Ph	H	6-Cl	3r	98	96:4	99
15^e^	Ph	H	6-OMe	3s	93	98:2	95

a Reaction condition: catalyst (1 mol%) and styrene 2 (5.0 equiv., 1 mmol) were dissolved in CH_2_Cl_2_ (2.0 mL), and diazooxindole 1 (0.2 mmol) in CH_2_Cl_2_ (2 mL) was added.

b Isolated yield.

c Determined by NMR.

d Determined by chiral HPLC analysis.

e Toluene : CH_2_Cl_2_ was used as the solvent.

f Slow addition for 4 h, and stirring for 20 h.

Furthermore, various oxindoles examined under similar conditions resulted in high yields and stereoselectivities, except 5-Br group (Table 4, entries 13–15). The cyclopropanation of aliphatic alkenes such as 1-hexane, multi-substituted olefins, inner alkene derivatives and α,β-unsaturated carbonyl compounds was also examined; however, no cyclopropane product was observed owing to rapid dimerization from the diazo compound.

Furthermore, hetero-atom-substituted olefins were examined for spirocyclopropanation reactions. The results are summarized in Table 5. Various N-alkyl diazooxindoles were subjected to these conditions with 2a, and all the tested diazooxindoles provided the cyclopropane product in good yields with high diastereoselectivity and enantioselectivity.

**Table 5:** Cyclopropanation of diazooxindole 1a with vinyl ester and vinyl amines^a^

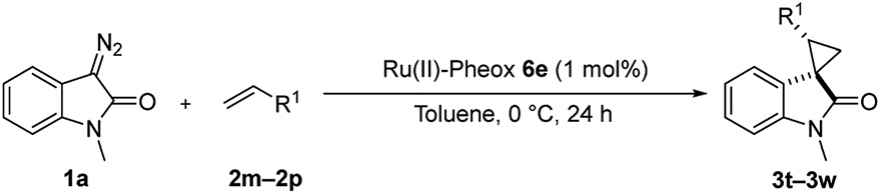					
Entry	R^1^	Product	Yield^b^ [%]	*trans*/*cis*^c^	ee^d^ [%]
1^e^^,^^f^	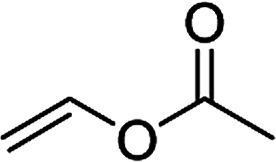	3t	84	98:2	90
2^e^^,^^f^	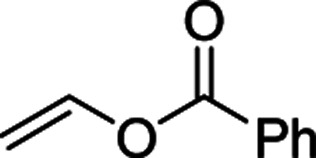	3u	85	>99:1<	92
3	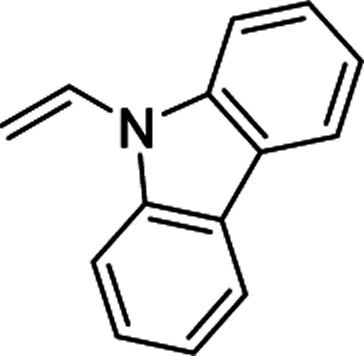	3v	92	92:8	78
4^e^^,^^f^^,^^g^	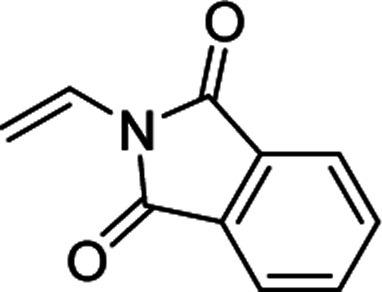	3w	37	86:14	93

a Reaction condition: catalyst (1 mol%) and styrene 2 (5.0 equiv., 1 mmol) were dissolved in CH_2_Cl_2_ (2.0 mL), and diazooxindole 1 (0.2 mmol) in CH_2_Cl_2_ (2 mL) was added.

b Isolated yield.

c Determined by NMR.

d Determined by chiral HPLC analysis.

e Toluene : CH_2_Cl_2_ was used as the solvent.

f Slow addition for 4 h, and stirring for 20 h.

g 98% conversion.

When the substrate has low solubility in toluene, a mixed solvent system such as CH_2_Cl_2_ : toluene = 1 : 1 is efficient to improve the reactivity without decreasing the stereoselectivity (Table 5, entries 1, 2, and 4). Proton nuclear magnetic resonance (H-NMR) analysis suggested the quantitative yield for the spirocyclopropanation reaction of vinyl phthalimide 2o although the isolated yield was 37%. The product 3w from vinyl phthalimide was unstable during the purification through column chromatography on both silica gel and alumina (Table 5, entry 4).

Furthermore, we investigated the estimation of the transition state related to the reaction mechanism using computational chemical analysis for the metal–carbene complex derived from diazooxindole and Ru(ii)-Pheox catalyst (Table S1, Fig. 3 and S1–S3†).^17–19^ The computational chemical analysis shows the Ru-oxindole carbene complex, which suggests that π–π interaction between oxindole and the indane aromatic ring controls the chiral environment to induce high enantioselectivity. Thus, an olefin may approach from only one side to provide high stereoselectivity as shown in Fig. 3. The absolute stereochemistry was determined using X-ray analysis (Scheme 1).

**Fig. 3:**
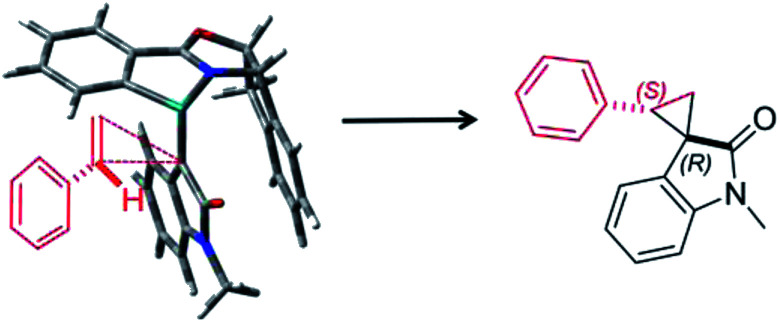
Plausible transition state of spirocyclopropanation.

**Scheme 1:**
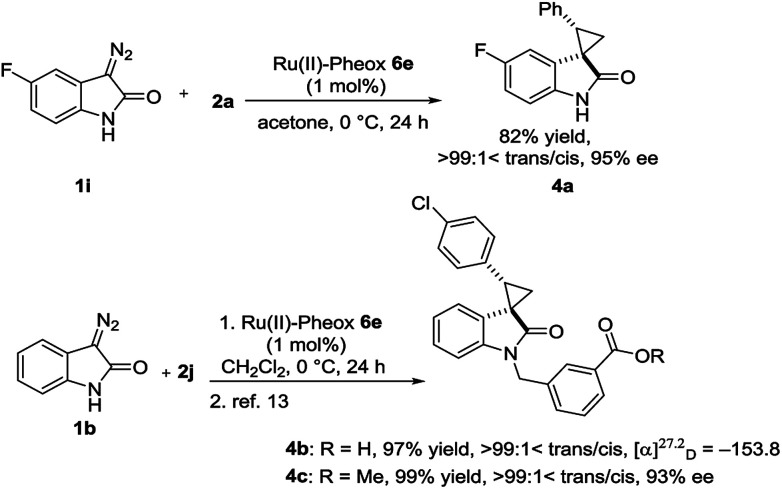
Synthesis of bioactive compounds.

To demonstrate the utility of our direct enantioselective cyclopropyloxindole synthesis, we demonstrated the first catalytic asymmetric synthesis of bioactive compounds such as anti-HIV activity candidate 4a and AMPK modulator 4c from diazooxindoles 1i and 1b, respectively. Both spirocyclopropanations proceeded smoothly, resulting in high yield with high enantioselectivity (4a: 82% yield, 95% ee, 4b: 99% yield, 93% ee as it is methyl ester).^2*e*,16^

In summary, the catalytic asymmetric cyclopropanation reaction of diazooxindoles and olefins by a series of Ru(ii)-Pheox catalysts is very effective for the synthesis of optically active spirocyclopropyloxindole derivatives. The stereoselectivity is good to excellent in most cases and optically active spirocyclopropyloxindole derivatives can be synthesized under a mild reaction condition in a short step. Furthermore, this strategy could be applied for the first synthesis of optically active bioactive compounds such as anti-HIV candidates and an AMPK modulator.

## Conflicts of interest

There are no conflicts to declare.

## Supplementary Material

RA-008-C8RA09212E-s001

RA-008-C8RA09212E-s002
