# Supplementary material for: Highly stereoselective spirocyclopropanation of various diazooxindoles with olefins catalyzed using Ru(ii)-complex
Source: RSC Adv. 2018 Nov 28;8(70):39865–9. doi: 10.1039/c8ra09212e (PMC9092356; doi:10.1039/c8ra09212e)
Supplement: RA-008-C8RA09212E-s001 [file RA-008-C8RA09212E-s001.pdf]

Electronic Supplementary Material (ESI) for RSC Advances.
This journal is © The Royal Society of Chemistry 2018

Supporting Information

CONTENTS:

1. Preparation of Various Diazooxindoles 2
2. General Procedure for catalytic Asymmetric Intermolecular Cyclopropanation of Diazooxindoles with Olefins. 3
3. Analytical Date for Asymmetric Cyclopropanation Reaction Products. 3
4. Ssynthesis of Bioactive Compound. 13
5. X-ray Crystal Structure 16
6. NMR Spectral Data 23
7. HPLC Spectral Data 53
8. Calculation Method 79
9. Calculation result 79
10. Plausible Mechanism 80
11. Reference 81

General: All reactions were performed under an atmosphere of argon unless otherwise noted. Dichloromethane (CH_2Cl_2) was purchased from Kanto Chemical Co., Inc.. All reactions were monitored by thin layer chromatography (TLC), glass plates pre-coated with silica gel Merck KGaA 60 F_{254}, layer thickness 0.2 mm. The products were visualized by irradiation with UV light or by treatment with a solution of phosphomolybdic acid or by treatment with a solution of *p*-anisaldehyde. Flash column chromatography was performed using silica gel (Merck, Art. No. 7734). ^{1}H NMR (500 MHz, 400 MHz) and ^{13}C NMR (125 MHz, 100 MHz) spectra were recorded on JEOL JNM-ECX 500, JEOL JNM-ECS 400 spectrometer. Chemical shifts are reported as δ values (ppm) relative to internal tetramethylsilane (0.00 ppm) in $CDCl_3$. Optical rotations were performed with a JASCO P-1030 polarimeter at the sodium D line (1.0 mL sample cell). Enantiomeric excesses were determined by high-performance liquid chromatography (HPLC) analyses with a JASCO GULLIVER using Daicel CHIRALPAK or CHIRALCEL columns. DART mass (positive mode) analyses were performed on a LC-TOF JMS-T100LP.

1. Preparation of Various Diazooxindoles

Diazooxindoles, (**1a**[1], **1b**[2], **1c**[3], **1d**[2], **1e**[2], **1f**[4], **1g**[2,4], and **1h**[5]) were synthesized by following the literature [1]-[5].

3-Diazo-1-ethylindolin-2-one (1c)

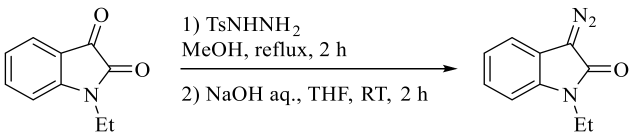

1c

1-Ethylindoline-2, 3-dione (155.9 mg, 0.89 mmol, 1 equiv.) and tosylhydrazine (182.5 mg, 0.98 mmol, 1.1 equiv.) were dissolved in MeOH (5 mL). The reaction mixture was refluxed for 2 h and then allowed to reach room temperature and the solid was filtered off. The residue was suspended in THF (5 mL) and treated with 0.2M NaOH (8.9 mL, 1.8 mmol) water solution at room temperature. The reaction mixture was stirred for 2 h, then neutralized by addition of dry-ice, diluted with brine and extracted with EtOAc. The combined organic layers were dried over Na_2SO_4, filtered and concentrated. The residue was purified by flash column chromatography with Hexane/EtOAc to give **1c** as red oil (88% yield, 146.6 mg, 0.780 mmol). ^{1}H NMR (400 MHz, $CDCl_3$) δ 7.24–7.16 (m, 2H), 7.08 (t, *J* = 7.64 Hz, 1H), 6.95 (d, *J* = 8.03 Hz, 1H), 3.88 (q, *J* = 7.13 Hz, 2H, -NC**H$_2$**CH$_3$), 1.30 (t, *J* = 7.26 Hz, 3H, -CH$_2$C**H$_3$**) ppm. ^{13}C NMR (100 MHz, $CDCl_3$) δ 166.55 (-NC=O), 133.63, 125.49, 121.97, 118.47, 117.03, 108.82, 35.52, 13.41 ppm. HRMS (DART) calcd for $C_{17}H_{16}NO$ $[M+H]^+$: 188.0823 found: 188.0823.

5-Bromo-3-diazo-1-methylindolin-2-one (1f)

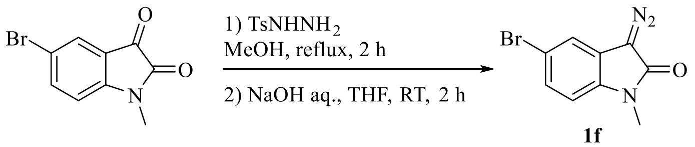

1f

5-Bromo-1-methylindoline-2, 3-dione (504.13 mg, 2.1 mmol, 1 equiv.) and tosylhydrazine (430.19 mg, 2.31 mmol, 1.1 equiv.) were dissolved in MeOH (15 mL). The reaction mixture was refluxed for 2 h and then allowed to reach room temperature and the solid was filtered off. The residue was suspended in THF (20 mL) and treated with 0.2M NaOH (20 mL, 4.2 mmol) water solution at room temperature. The reaction mixture was stirred for 2 h, then neutralized by addition of dry-ice, diluted with brine and extracted with EtOAc. The combined organic layers were dried over Na_2SO_4, filtered and concentrated. The residue was purified by flash column chromatography with Hexane/EtOAc to give **1f** as red solid (28% yield, 148.7 mg, 0.59 mmol). ^{1}H NMR (500 MHz, $CDCl_3$) δ 7.39–7.23 (m, 2H), 6.82–6.73 (m, 1H), 3.30 (s, 3H, -NC**H$_3$**) ppm. ^{13}C NMR (125 MHz, $CDCl_3$) δ 166.16 (-NC=O), 133.51, 128.30, 120.96, 118.66, 114.63, 109.92, 27.03 ppm. HRMS (DART) calcd for $C_9H_7BrN_3O$ $[M+H]^+$: 251.9772 found: 251.9772.

6-Chloro-3-diazo-1-methylindolin-2-one (1g)

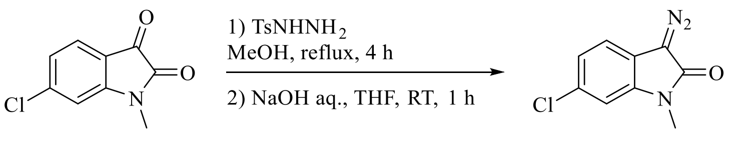

6-Chloro-1-methylindoline-2, 3-dione (154.5 mg, 0.8 mmol, 1 equiv.) and tosylhydrazine (134.4 mg, 0.87 mmol, 1.1 equiv.) were dissolved in MeOH (5 mL). The reaction mixture was refluxed for 4 h and then allowed to reach room temperature and the solid was filtered off. The residue was suspended in THF (5 mL) and treated with 0.2M NaOH water solution (7.9 mL, 1.6 mmol) at room temperature. The reaction mixture was stirred for 1 h, then neutralized by addition of dry-ice, diluted with brine and extracted with EtOAc. The combined organic layers were dried over Na_2SO_4, filtered and concentrated. The residue was purified by flash column chromatography with Hexane/EtOAc to give **1g** as pale-orange solid (72% yield, 118.1 mg, 0.58 mmol). ^{1}H NMR (500 MHz, $CDCl_3$) δ 7.13–7.05 (m, 2H), 6.93 (d, *J* = 1.53 Hz, 1H), 3.32 (s, 3H, -NC**H₃**) ppm. ^{13}C NMR (125 MHz, $CDCl_3$) δ 166.85 (-NC=O), 135.53, 131.42, 122.19, 118.95, 115.14, 109.47, 27.01 ppm. HRMS (DART) calcd for $C_{17}H_{16}ClNO$ $[M+H]^+$: 208.0277 found: 208.0277

2. General Procedure for catalytic Asymmetric Intermolecular Cyclopropanation of Diazooxindoles with Olefins.

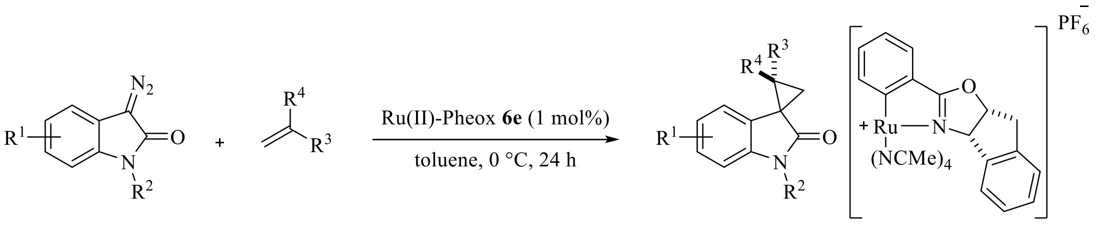

The solution of diazooxindole (0.2 mmol) in toluene (2 mL) was slowly added to a mixture of Ru(II)-Pheox **6e** (0.002 mmol) and olefins (1.0 mmol) in toluene (2 mL) for 2 min under argon atmosphere at 0 °C. After the addition completed, the reaction mixture was then stirred for 24 h at 0 °C. The progress of the reaction was monitored by TLC. Upon completion, solvent was removed and residue was purified by column chromatography on silica gel eluted with EtOAc/*n*-Hexane to give desired product. The trans/cis ratio was determined from the crude ^{1}H NMR spectra, and ee value was determined by chiral HPLC analysis.

3. Analytical Date for Asymmetric Cyclopropanation Reaction Products.

(1*R*,2*S*)-1'-Methyl-2-phenylspiro[cyclopropane-1,3'-indolin]-2'-one (3a)

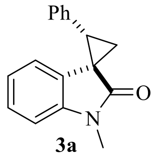

3a

This compound was prepared according to the typical procedure for asymmetric intermolecular cyclopropanation reaction of between styrene **2a** (104.2 mg, 1mmol) and 3-diazo-1-methylindolin-2-one **1a** (34.6 mg, 0.2 mmol). The reaction mixture was purified by silica gel column chromatography with EtOAc/n-Hexane as an eluent to give **3a** in 94% yield as red oil (46.9 mg, 0.188 mmol), *trans/cis* = 94:6, 96% *trans* ee. $[\alpha]^{26.5}_D$ = –100.6 (c 1.0, acetone). ^{1}H NMR (500

MHz, $CDCl_3$) δ 7.34– 7.22 (m, 3H), 7.21–7.17 (m, 2H), 7.14 (td, *J* = 7.84, 1.15 Hz, 1H), 6.86 (d, *J* = 7.64 Hz, 1H) 6.68 (td, *J* = 7.64, 0.76 Hz, 1H), 5.96 (d, *J* = 7.26 Hz, 1H), 3.34 (t, *J* = 8.79 Hz, 1H, -C**H** (cyclopropane)), 3.32 (s, 3H, -NC**H₃**) 2.18 (dd, *J* = 9.17, 4.59 Hz, 1H, -C**Hβ**H (cyclopropane)), 1.99 (dd, *J* = 8.03, 4.59 Hz, 1H, -CH**Hα** (cyclopropane)) ppm. ^{13}C NMR (100 MHz, $CDCl_3$) δ 176.55 (-NC=O), 143.90, 135.25, 130.01, 128.45, 127.59, 127.46, 126.66, 121.56, 120.75, 107.86, 35.89, 33.37, 26.73, 22.53 ppm. The ee value was determined by HPLC analysis. Column (Chiral AD-H), UV 230 nm, eluent: Hexane/IPA = 19:1, Flow rate = 1.0 mL/min, tR = 8.6 min (major product), tR = 11.0 min (minor product). HRMS (DART) calcd for $C_{17}H_{16}NO$ $[M+H]^+$: 250.1231 found: 250.1231.

(1*R*,2*S*)-2-Phenylspiro[cyclopropane-1,3'-indolin]-2'-one (3b)

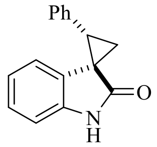

3b

The solution of diazooxindole (0.2 mmol) in CH_2Cl_2 (2 mL) was slowly added to a mixture of Ru(II)-Pheox **6e** (0.002 mmol) and olefins (1.0 mmol) in CH_2Cl_2 (2 mL) for 2 min under argon atmosphere at 0 °C. This compound was prepared according to the typical procedure for asymmetric intermolecular cyclopropanation reaction of between styrene **2a** (104.2 mg, 1 mmol) and 3-diazoindolin-2-one **1b** (31.8 mg, 0.2 mmol). CH_2Cl_2 was used as solvent. The reaction mixture was purified by silica gel column chromatography with EtOAc/n-Hexane as an eluent to give **3b** in 98% yield as red solid (46.1 mg, 0.196 mmol). *trans/cis* = 93:7, 92% *trans* ee. $[\alpha]^{24.8}_D$ = –89.4 (c 1.0, $CHCl_3$). ^1H NMR (500 MHz, $CDCl_3$) δ 8.74 (s, 1H, -N**H**), 7.38–7.23 (m, 3H), 7.20 (d, *J* = 6.88 Hz, 2H), 7.09 (td, *J* = 7.74, 0.89 Hz, 1H), 6.95 (d, *J* = 7.64 Hz, 1H), 6.67 (t, *J* = 7.45 Hz, 1H), 5.95 (d, *J* = 7.64 Hz, 1H), 3.36 (t, *J* = 8.60 Hz, 1H, -C**H** (cyclopropane)), 2.22 (dd, *J* = 9.17, 4.59 Hz, 1H, -C**Hβ**H (cyclopropane)), 2.03 (dd, *J* = 8.03, 4.59 Hz, 1H, -CH**Hα** (cyclopropane)) ppm. ^{13}C NMR (100 MHz, $CDCl_3$) δ 178.78 (-NC=O), 141.03, 135.08, 130.10, 128.54, 128.05, 127.60, 126.73, 121.61, 121.12, 109.74, 36.30, 33.83, 22.81 ppm. The ee value was determined by HPLC analysis. Column (Chiral AD-H), UV 230 nm, eluent: Hexane/IPA = 9/1, Flow rate = 1.0 mL/min, tR = 9.8 min (major product), tR = 14.0 min (minor product). HRMS (DART) calcd for $C_{16}H_{14}NO$ $[M+H]^+$: 236.1075 found: 236.1074.

(1*R*,2*S*)-1'-Ethyl-2-phenylspiro[cyclopropane-1,3'-indolin]-2'-one (3c)

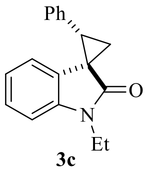

3c

This compound was prepared according to the typical procedure for asymmetric intermolecular cyclopropanation reaction of between styrene **2a** (104.2 mg, 1 mmol) and 1-ethylindoline-2,3-dione **1c** (37.4 mg, 0.2 mmol) The reaction mixture was purified by silica gel column chromatography with EtOAc/n-Hexane as an eluent to give **3c** in 94% yield as red solid (49.5 mg, 0.188 mmol). *trans/cis* = 97:3, 97% *trans* ee. $[\alpha]^{24.3}_D$ = –72.3 (c 1.0, $CHCl_3$). ^1H NMR (500 MHz, $CDCl_3$) δ 7.33–7.22 (m, 3H), 7.18 (dd, *J* = 6.88, 1.15 Hz, 2H), 7.13 (td, *J* = 7.84, 1.15 Hz, 1H), 6.89 (d, *J* = 7.64 Hz, 1H), 6.67 (td, *J* = 7.55, 1.02 Hz, 1H), 5.96 (dd, *J* = 7.64, 0.76 Hz, 1H), 3.88 (q, *J* = 7.26 Hz, 2H, -N**CH₂**CH₃), 3.33 (t, *J* = 8.60 Hz, 1H, -C**H** (cyclopropane)), 2.18 (dd, *J* = 9.17, 4.59 Hz, 1H, -C**Hβ**H (cyclopropane)), 1.98 (dd, *J* = 8.03, 4.59 Hz, 1H, -CH**Hα** (cyclopropane)), 1.33 (t, *J* = 7.26 Hz, 3H, -NCH₂**CH₃**) ppm. ^{13}C NMR (100 MHz, $CDCl_3$) δ 176.11 (-NC=O), 143.02, 135.32, 130.05, 128.46, 127.87, 127.46, 126.60, 121.33, 120.93, 108.02, 35.90, 35.21, 33.34, 22.65, 13.15 ppm. The ee value was determined by HPLC analysis. Column (Chiral AD-H), UV 230 nm, eluent: Hexane/IPA = 20/1, Flow rate = 1.0 mL/min, tR = 7.9 min (major product), tR = 9.6 min (minor product).

HRMS (DART) calcd for $C_{18}H_{18}NO$ $[M+H]^+$: 264.1388 found: 264.1389.

(1*R*,2*S*)-1'-Isopropyl-2-phenylspiro[cyclopropane-1,3'-indolin]-2'-one (3d)

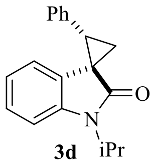

3d

This compound was prepared according to the typical procedure for asymmetric intermolecular cyclopropanation reaction of between styrene **2a** (104.2 mg, 1 mmol) and 3-diazo-1-isopropylindolin-2-one **3d** (40.3 mg, 0.2 mmol). The reaction mixture was purified by silica gel column chromatography with EtOAc/n-Hexane as an eluent to give **3d** in 86% yield as red oil (47.7 mg, 0.172 mmol). *trans/cis* = 96:4, 95% *trans* ee. $[\alpha]^{24.3}_D$ = –78.8 (c 1.0, $CHCl_3$). ^1H NMR (500 MHz, $CDCl_3$) δ 7.33–7.22 (m, 3H), 7.18 (d, *J* = 6.88 Hz, 2H), 7.11 (td, *J* = 7.64, 1.15 Hz, 1H), 7.04 (d, *J* = 7.64 Hz, 1H), 6.65 (t, *J* = 7.45 Hz, 1H), 5.96 (d, *J* = 7.26 Hz, 1H), 4.77 (hept, *J* = 6.88 Hz, 1H, -NC**H**(CH_3)$_2$), 3.32 (t, *J* = 8.51 Hz, 1H, -C**H** (cyclopropane)), 2.16 (dd, *J* = 9.17, 4.30 Hz, 1H, -C**Hβ**H (cyclopropane)), 1.96 (dd, *J* = 7.84, 4.30 Hz, 1H, -CH**Hα** (cyclopropane)), 1.54 (t, *J* = 6.88 Hz, 6H. –NH (C**H$_3$**)$_2$) ppm. ^{13}C NMR (100 MHz, $CDCl_3$) δ 176.14 (-NC=O), 142.52, 135.35, 130.10, 128.47, 128.17, 127.46, 126.33, 120.97, 109.68, 44.18, 36.16, 33.25, 22.93, 19.83, 19.81 ppm. The ee value was determined by HPLC analysis. Column (Chiral IE-3), UV 230 nm, eluent: Hexane/IPA = 100/1, Flow rate = 1.0 mL/min, tR = 32.7 min (major product), tR = 31.0 (minor product). HRMS (DART) calcd for $C_{19}H_{19}NO$ $[M]^+$: 277.1466 found: 277.1466.

(1*R*,2*S*)-1'-Benzyl-2-phenylspiro[cyclopropane-1,3'-indolin]-2'-one (3e)

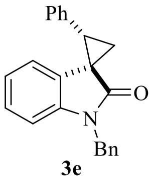

3e

This compound was prepared according to the typical procedure for asymmetric intermolecular cyclopropanation reaction of between styrene **2a** (104.2 mg, 1 mmol) and 1-benzyl-3-diazoindolin-2-one **1e** (52.7 mg, 0.2 mmol). The reaction mixture was purified by silica gel column chromatography with EtOAc/n-Hexane as an eluent to give **3e** in 97% yield as red solid (65.9 mg, 0.194 mmol). *trans/cis* = >99:1<, 98% *trans* ee. $[\alpha]^{25.1}_D$ = –122.8 (c 1.0, $CHCl_3$). ^1H NMR (500 MHz, $CDCl_3$) δ 7.37–7.22 (m, 8H), 7.19 (d, *J* = 7.26 Hz, 2H), 7.02 (t, *J* = 7.84 Hz, 1H), 6.75 (d, *J* = 7.64 Hz, 1H), 6.64 (t, *J* = 7.64 Hz, 1H), 5.96 (d, *J* = 7.26 Hz, 1H), 5.06 (*J* = 15.67 Hz, 1H, -NC**H**HAr), 4.99 (d, *J* = 15.67 Hz, 1H, -NCH**H**Ar), 3.41 (t, *J* = 8.60 Hz, 1H, -C**H** (cyclopropane)), 2.27 (dd, *J* = 9.17, 4.40 Hz, 1H, -C**Hβ**H (cyclopropane)), 2.04 (dd, *J* = 8.03, 4.40 Hz, 1H, -CH**Hα** (cyclopropane)) ppm. ^{13}C NMR (100 MHz, $CDCl_3$) δ 176.68 (-NC=O), 143.03, 136.35, 135.20, 130.08, 128.89, 128.51, 127.66, 127.58, 127.53, 127.40, 126.61, 121.61, 120.86, 108.90, 44.29, 36.26, 33.34, 22.79 ppm. The ee value was determined by HPLC analysis. Column (Chiral IE-3), UV 230 nm, eluent: Hexane/IPA = 10/1, Flow rate = 1.0 mL/min, tR = 17.1 min (major product), tR = 15.1 min (minor product). HRMS (DART) calcd for $C_{23}H_{20}NO$ $[M+H]^+$: 326.1544 found: 326.1549.

(1*R*,2*S*)-1'-Methyl-2-(o-tolyl)spiro[cyclopropane-1,3'-indolin]-2'-one (3f)

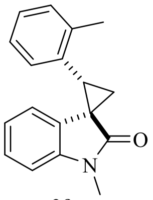

3f

This compound was prepared according to the typical procedure for asymmetric intermolecular cyclopropanation reaction of between 2-methylstyrene **2b** (118.2 mg, 1 mmol) and 3-diazo-1-methylindolin-2-one **1a** (34.6 mg, 0.2 mmol). The reaction mixture was purified by silica gel column chromatography with EtOAc/n-Hexane as an eluent to give **3f** in 92% yield as

red solid (48.5 mg, 0.184 mmol). *trans/cis* = 92:8, 95% *trans* ee. $[\alpha]^{23.9}_{D}$ = –110.3 (c 1.0, $CHCl_3$). ^1H NMR (500 MHz, $CDCl_3$) δ 7.37 (d, *J* = 7.64 Hz, 1H), 7.26–7.21 (m, 1H), 7.19 (t, *J* = 7.45 Hz, 1H), 7.14 (td, *J* = 7.84, 1.15 Hz), 7.02 (d, *J* = 7.64 Hz, 1H), 6.86 (d, *J* = 7.64 Hz, 1H), 6.64 (td, *J* = 7.64, 0.76 Hz, 1H), 5.84 (d, *J* = 7.64 Hz, 1H), 3.34 (s, 3H, -N**CH₃**), 3.14 (t, *J* = 8.60 Hz, 1H, -C**H** (cyclopropane)), 2.23 (dd, *J* = 9.17, 4.59 Hz, 1H, -C**Hβ**H (cyclopropane)), 2.03 (dd, *J* = 8.03, 4.59 Hz, 1H, -CH**Hα** (cyclopropane)), 1.75 (s, 3H, Ar-C**H₃**) ppm. ^{13}C NMR (100 MHz, $CDCl_3$) δ 176.55 (-NC=O), 143.67, 139.29, 134.10 129.94, 128.72, 127.69, 127.50, 126.68, 125.89, 121.61, 119.92, 107.77, 35.08, 33.07, 26.79, 22.52, 19.20 ppm. The ee value was determined by HPLC analysis. Column (Chiral AD-H), UV 230 nm, eluent: Hexane/IPA = 20/1, Flow rate = 1.0 mL/min, tR = 8.1 min (major product), tR = 9.1 min (minor product). HRMS (DART) calcd for $C_{18}H_{18}NO$ $[M+H]^+$: 264.1388 found: 264.1386.

(1*R*,2*S*)-1'-Methyl-2-(m-tolyl)spiro[cyclopropane-1,3'-indolin]-2'-one (3g)

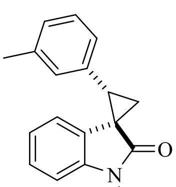

3g

This compound was prepared according to the typical procedure for asymmetric intermolecular cyclopropanation reaction of between 3-methylstyrene **2c** (118.2 mg, 1 mmol) and 3-diazo-1-methylindolin-2-one **1a** (34.6 mg, 0.2 mmol). The reaction mixture was purified by silica gel column chromatography with EtOAc/n-Hexane as an eluent to give **3g** in 80% yield as red solid (42.1 mg, 0.160 mmol). *trans/cis* = >99:1<, 99% *trans* ee. $[\alpha]^{24.2}_{D}$ = –84.9 (c 1.0, $CHCl_3$). ^1H NMR (500 MHz, $CDCl_3$) δ 7.19–7.12 (m, 2H), 7.06 (d, *J* = 6.88 Hz, 1H), 7.02 (s, 1H), 6.97 (d, *J* = 7.64 Hz, 1H), 6.87 (d, *J* = 7.64 Hz, 1H), 6.70 (td, *J* = 7.55, 0.88 Hz, 1H), 6.01 (d, *J* = 7.64 Hz, 1H), 3.33 (s, 3H, -N**CH₃**), 3.31 (t, *J* = 8.60 Hz, 1H, -C**H** (cyclopropane)), 2.30 (s, 3H, Ar-C**H₃**), 2.16 (dd, *J* = 9.17, 4.59 Hz, 1H, -C**Hβ**H (cyclopropane)), 1.99 (dd, *J* = 8.03, 4.59 Hz, 1H, -CH**Hα** (cyclopropane)) ppm. ^{13}C NMR δ (100 MHz, $CDCl_3$) 176.62 (-NC=O), 143.91, 138.06, 135.12, 130.74, 128.30, 128.23, 127.75, 126.99, 126.61, 121.59, 120.83, 107.84, 35.92, 33.41, 26.74, 22.62, 21.48 ppm. The ee value was determined by HPLC analysis. Column (Chiral AD-H), UV 230 nm, eluent: Hexane/IPA = 19/1, Flow rate = 1.0 mL/min, tR = 7.6 min (major product), tR = 8.7 min (minor product). HRMS (DART) calcd for $C_{18}H_{18}NO$ $[M+H]^+$: 264.1388 found: 264.1383.

(1*R*,2*S*)-1'-Methyl-2-(p-tolyl)spiro[cyclopropane-1,3'-indolin]-2'-one (3h)

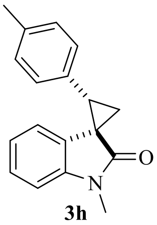

3h

This compound was prepared according to the typical procedure for asymmetric intermolecular cyclopropanation reaction of between 4-methylstyrene **2d** (118.2 mg, 1 mmol) and 3-diazo-1-methylindolin-2-one **1a** (34.6 mg, 0.2 mmol). The reaction mixture was purified by silica gel column chromatography with EtOAc/n-Hexane as an eluent to give **3h** in 96% yield as red solid (50.6 mg, 0.192 mmol). *trans/cis* = >99:1<, 96% *trans* ee. $[\alpha]^{26.3}_{D}$ = –129.2 (c 1.0, $CHCl_3$). ^1H NMR (500 MHz, $CDCl_3$) δ 7.15 (td, *J* = 7.74, 0.89 Hz, 1H), 7.09 (d, *J* = 8.41 Hz, 2H), 7.06 (d, *J* = 8.41, 2H), 6.86 (d, *J* = 7.64 Hz, 1H), 6.71 (t, *J* = 7.45 Hz, 1H), 6.00 (d, *J* = 7.64 Hz, 1H), 3.32 (s, 1H, -NC**H₃**), 3.30 (t, *J* = 8.60 Hz, 1H, -C**H** (cyclopropane)), 2.32 (s, 3H, Ar-C**H₃**), 2.17 (dd, *J* = 9.17, 4.20 Hz, 1H, -C**Hβ**H (cyclopropane)), 1.97 (dd, *J* = 8.03, 4.20 Hz, 1H, -CH**Hα** (cyclopropane)) ppm. ^{13}C NMR (100 MHz, $CDCl_3$) δ 176.60 (-NC=O), 143.87, 137.07, 132.11, 129.82, 129.14, 127.73, 126.55, 121.54, 120.80, 107.79, 35.72, 33.38, 26.69, 22.64, 21.25 ppm. The ee value was determined by HPLC analysis. Column (Chiral IA-3), UV 230 nm, eluent: Hexane/IPA = 10/1, Flow rate = 1.0 mL/min, tR = 6.6 min (major product), tR = 7.2 min (minor product). HRMS (DART) calcd for

$C_{18}H_{18}NO$ [M+H]$^+$: 264.1388 found: 264.1388.

(1*R*,2*S*)-2-(4-(Tert-butyl)phenyl)-1'-methylspiro[cyclopropane-1,3'-indolin]-2'-one (3i)

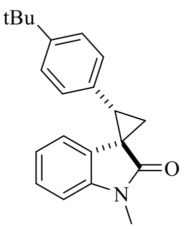

3i

This compound was prepared according to the typical procedure for asymmetric intermolecular cyclopropanation reaction of between 4-tert-butylstyrene **2e** (160.3 mg, 1 mmol) and 3-diazo-1-methylindolin-2-one **1a** (34.6 mg, 0.2 mmol). The reaction mixture was purified by silica gel column chromatography with EtOAc/n-Hexane as an eluent to give **3i** in 97% yield as red oil (59.3 mg, 0.194 mmol). *trans/cis* = >99:1<, 95% *trans* ee. $[\alpha]^{24.5}_D$ = –142.3 (c 1.0, $CHCl_3$). ^{1}H NMR (400 MHz, $CDCl_3$) δ 7.31–7.28 (m, 2H), 7.17–7.08 (m, 3H), 6.86 (d, *J* = 7.63 Hz, 1H), 6.69 (td, *J* = 7.63, 0.92 Hz, 1H), 6.00 (d, *J* = 7.63 Hz, 1H), 3.32 (s, 3H, -NC**H$_3$**), 3.29 (t, *J* = 8.55 Hz, 1H, -C**H** (cyclopropane)), 2.17 (dd, *J* = 9.16, 4.58 Hz, 1H, -C**Hβ**H (cyclopropane)), 1.98 (dd, *J* = 7.93, 4.58 Hz, 1H, -CH**Hα** (cyclopropane)), 1.30 (s, 9H, Ar-(C**H$_3$**)$_3$) ppm. ^{13}C NMR (100 MHz, $CDCl_3$) δ 176.68 (-NC=O), 150.44, 143.92, 132.14, 129.64, 127.82, 126.58, 125.33, 121.52, 120.87, 107.80, 35.70, 34.62, 33.47, 31.43, 26.74, 22.72 ppm. The ee value was determined by HPLC analysis. Column (Chiral OJ-H), UV 230 nm, eluent: Hexane/IPA = 20/1, Flow rate = 1.0 mL/min, tR = 8.2 min (major product), tR = 10.7 min (minor product). HRMS (DART) calcd for $C_{21}H_{24}NO$ [M+H]$^+$: 306.1857 found: 305.1856.

(1*R*,2*S*)-1'-Methyl-2-(naphthalen-2-yl)spiro[cyclopropane-1,3'-indolin]-2'-one (3j)

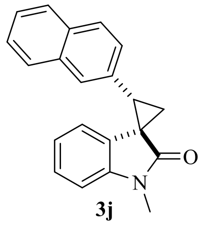

3j

The solution of diazooxindole (0.2 mmol) in toluene:CH_2Cl_2 = 1:1 (2 mL) was slowly added to a mixture of Ru(II)-Pheox **6e** (0.002 mmol) and olefins (1.0 mmol) in toluene:CH_2Cl_2 = 1:1 (2 mL) for 2 min under argon atmosphere at 0 °C. This compound was prepared according to the typical procedure for asymmetric intermolecular cyclopropanation reaction of between 2-vinylnaphthalene **2f** (154.2 mg, 1 mmol) and 3-diazo-1-methylindolin-2-one **1a** (34.6 mg, 0.2 mmol). The reaction mixture was purified by silica gel column chromatography with EtOAc/n-Hexane as an eluent to give **3j** in 83% yield as white solid (50 mg, 0.166 mmol). *trans/cis* = >99:1<, 96% *trans* ee. $[\alpha]^{24.9}_D$ = –359.5 (c 1.0, $CHCl_3$). ^{1}H NMR (500 MHz, $CDCl_3$) δ 7.84–7.77 (m, 2H), 7.74 (s, 1H), 7.69 (d, *J* = 8.41 Hz, 1H), 7.51–7.42 (m, 2H), 7.18 (dd, *J* = 8.41, 1.53 Hz, 1H), 7.10 (td, *J* = 7.64, 1.15 Hz, 1H), 6.85 (d, *J* = 8.03 Hz, 1H), 6.56 (t, *J* = 7.45 Hz, 1H), 5.94 (d, *J* = 7.64 Hz, 1H), 3.47 (t, *J* = 8.51 Hz, 1H, -C**H** (cyclopropane)), 3.33 (s, 3H, -NC**H$_3$**), 2.26 (dd, *J* = 9.17, 4.50 Hz, 1H, -C**Hβ**H (cyclopropane)), 2.13 (dd, *J* = 7.84 , 4.50 Hz, 1H, -CH**Hα** (cyclopropane)) ppm. ^{13}C NMR (100 MHz, $CDCl_3$) δ 176.53 (-NC=O), 143.90, 133.31, 133.00, 132.77, 128.34, 128.31, 128.15, 127.87, 127.82, 127.50, 126.68, 126.29, 126.08, 121.68, 120.70, 107.89, 36.10, 33.49, 26.77, 22.68 ppm. The ee value was determined by HPLC analysis. Column (Chiral AD-H), UV 230 nm, eluent: Hexane/IPA = 20/1, Flow rate = 1.0 mL/min, tR = 13.5 min (major product), tR = 15.6 min (minor product). HRMS (DART) calcd for $C_{21}H_{18}NO$ [M+H]$^+$: 300.1388 found: 300.1388.

(1*R*,2*S*)-1',2-Dimethyl-2-phenylspiro[cyclopropane-1,3'-indolin]-2'-one (3k)

The solution of diazooxindole (0.2 mmol) in toluene (2 mL) was slowly added to a mixture of Ru(II)-Pheox **6e** (0.002 mmol) and olefins (1.0 mmol) in toluene (2 mL) for 4 h under argon atmosphere at 0 °C. This compound

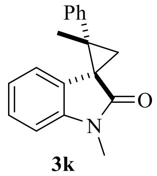

3k

was prepared according to the typical procedure for asymmetric intermolecular cyclopropanation reaction of between α-methylstyrene **2g** (118.2 mg, 1 mmol) and 3-diazo-1-methylindolin-2-one **1a** (34.6 mg, 0.2 mmol). The reaction mixture was purified by silica gel column chromatography with EtOAc/n-Hexane as an eluent to give **3k** in 93% yield as red solid (50 mg, 0.186 mmol). *trans/cis* = 98:2, 97% *trans* ee. $[\alpha]^{24.1}_{D}$ = –52.9 (c 1.0, $CHCl_3$). ^{1}H NMR (400 MHz, $CDCl_3$) δ 7.39–6.56 (m, 8H), 5.49 (d, *J* = 7.02 Hz, 1H), 3.32 (s, 3H, -NC**H$_3$**), 2.10 (d, *J* = 4.89 Hz, 1H, -C**H** (cyclopropane)), 2.09 (d, *J* = 4.89, 1H, -C**H** (cyclopropane)), 1.86 (s, 3H, cyclopropane-C**H$_3$**) ppm. ^{13}C NMR (100 MHz, $(CD_3)_2CO$) δ 174.95 (-NC=O), 143.63, 141.98, 129.86, 128.85, 128.62, 127.63, 126.70, 121.20, 121.06, 108.31, 41.39, 36.26, 29.58, 26.84, 21.18 ppm. The ee value was determined by HPLC analysis. Column (Chiral AD-H), UV 230 nm, eluent: Hexane/IPA = 19/1, Flow rate = 1.0 mL/min, tR = 10.7 min (major product), tR = 13.8 min (minor product). HRMS (DART) calcd for $C_{18}H_{18}NO$ $[M+H]^+$: 264.1388 found: 264.1381.

(1*R*,2*S*)-1'-Methyl-2-(4-nitrophenyl)spiro[cyclopropane-1,3'-indolin]-2'-one (3l)

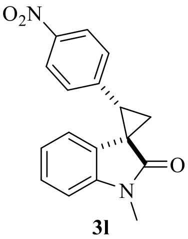

3l

The solution of diazooxindole (0.2 mmol) in toluene:CH_2Cl_2 = 1:1 (2 mL) was slowly added to a mixture of Ru(II)-Pheox **6e** (0.002 mmol) and olefins (1.0 mmol) in toluene:CH_2Cl_2 = 1:1 (2 mL) for 2 min under argon atmosphere at 0 °C. This compound was prepared according to the typical procedure for asymmetric intermolecular cyclopropanation reaction of between 4-nitrostyrene **2h** (149.2 mg, 1 mmol) and 3-diazo-1-methylindolin-2-one **1a** (34.6 mg, 0.2 mmol). The reaction mixture was purified by silica gel column chromatography with EtOAc/n-Hexane as an eluent to give **3l** in 85% yield as yellow solid (50.0 mg, 0.170 mmol). *trans/cis* = 96:4, 94% *trans* ee. $[\alpha]^{26.4}_{D}$ = –156.0 (c 1.2, $CHCl_3$). ^{1}H NMR (500 MHz, $CDCl_3$) δ 8.17 (dd, *J* = 8.41 Hz, 2H), 7.37 (d, *J* = 8.41 Hz, 2H), 7.19 (td, *J* = 7.64, 1.15 Hz, 1H), 6.90 (d, *J* = 7.64 Hz, 1H), 6.72 (td, *J* = 7.55,1.34 Hz, 1H), 5.93 (d, *J* = 6.88 Hz, 1H), 3.37–3.31 (m, 4H, -NC**H$_3$**, -C**H** (cyclopropane)), 2.27 (dd, *J* = 8.98, 4.89 Hz, 1H, -C**Hβ**H (cyclopropane)), 2.02 (dd, *J* = 8.03, 4.89 Hz, 1H, -CH**Hα** (cyclopropane)) ppm. ^{13}C NMR (100 MHz, $CDCl_3$) δ 175.80 (-NC=O), 147.29, 144.07, 143.10, 130.94, 127.37, 126.46, 123.76, 121.89, 120.48, 108.32, 34.94, 33.61, 26.86, 22.13 ppm. The ee value was determined by HPLC analysis. Column (Chiral IF-3), UV 230 nm, eluent: Hexane/IPA = 15/1, Flow rate = 1.0 mL/min, tR = 24.3 min (major product), tR = 32.9 min (minor product). HRMS (DART) calcd for $C_{17}H_{15}N_2O_3$ $[M+H]^+$: 295.1082 found: 295.1082.

(1*R*,2*S*)-2-(4-Bromophenyl)-1'-methylspiro[cyclopropane-1,3'-indolin]-2'-one (3m)

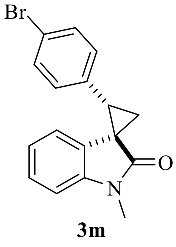

3m

This compound was prepared according to the typical procedure for asymmetric intermolecular cyclopropanation reaction of between 4-bromostyrene **2i** (183.1 mg, 1 mmol) and 3-diazo-1-methylindolin-2-one **1a** (34.6 mg, 0.2 mmol). The reaction mixture was purified by silica gel column chromatography with EtOAc/n-Hexane as an eluent to give **3m** in 98% yield as red solid (64.3 mg, 0.196 mmol). *trans/cis* = 96:4, 94% *trans* ee. $[\alpha]^{24.9}_{D}$ = –127.1 (c 1.0, $CHCl_3$). ^{1}H NMR (500 MHz, $CDCl_3$) δ 7.41 (d, *J* = 8.22 Hz, 2H), 7.18 (t, *J* = 7.64 Hz, 1H), 7.06 (d, *J* = 8.22 Hz, 2H), 6.88 (d, *J* = 7.64 Hz, 1H), 6.74 (d, *J* = 7.64 Hz, 1H), 5.97 (d, *J* = 7.64 Hz, 1H), 3.32 (s, 3H, -NC**H$_3$**), 3.24 (t, *J* = 8.60 Hz, 1H, -C**H** (cyclopropane)), 2.18 (dd, *J* = 9.17, 4.59 Hz, 1H, -C**Hβ**H

(cyclopropane)), 1.93 (dd, *J* = 8.03, 4.59 Hz, 1H, -CH**Hα** (cyclopropane)) ppm. ^{13}C NMR (100 MHz, $CDCl_3$) δ 176.29 (-NC=O), 143.96, 134.42, 131.76, 131.65, 127.18, 126.92, 121.77, 121.49, 120.77, 108.04, 35.07, 33.27, 26.79, 22.45 ppm. The ee value was determined by HPLC analysis. Column (Chiral OZ-H), UV 230 nm, eluent: Hexane/IPA = 10/1, Flow rate = 1.0 mL/min, tR = 12.6 min (major product), tR = 15.5 min (minor product). HRMS (DART) calcd for $C_{17}H_{15}BrNO$ $[M+H]^+$: 328.0337 found: 328.0330.

(1*R*,2*S*)-2-(4-Chlorophenyl)-1'-methylspiro[cyclopropane-1,3'-indolin]-2'-one (3n)

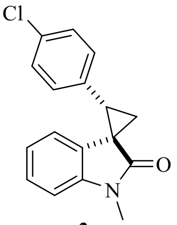

3n

This compound was prepared according to the typical procedure for asymmetric intermolecular cyclopropanation reaction of between 4-chlorostyrene **2j** (138.6 mg, 1 mmol) and 3-diazo-1-methylindolin-2-one **1a** (34.6 mg, 0.2 mmol). The reaction mixture was purified by silica gel column chromatography with EtOAc/n-Hexane as an eluent to give **3n** in 98% yield as red solid (55.6 mg, 0.196 mmol). *trans/cis* = 96:4, 93% *trans* ee. $[\alpha]^{25.3}_{D}$ = –144.9 (c 1.0, $CHCl_3$). ^{1}H NMR (500 MHz, $CDCl_3$) δ 7.26 (d, *J* = 8.41 Hz, 2H), 7.17 (td, *J* = 7.74, 1.02 Hz, 1H), 7.11 (d, *J* = 8.41 Hz, 2H), 6.88 (d, *J* = 7.64 Hz, 1H), 6.73 (dd, *J* = 7.45 Hz, 1H), 5.97 (d, *J* = 7.26 Hz, 1H), 3.32 (s, 3H, -NC**H$_3$**), 3.26 (t, *J* = 8.60 Hz, 1H, -C**H** (cyclopropane)), 2.18 (dd, *J* = 9.17, 4.59 Hz, 1H, -C**Hβ**H (cyclopropane)), 1.93 (dd, *J* = 8.03, 4.59 Hz, 1H, -CH**Hα** (cyclopropane)) ppm. ^{13}C NMR (100 MHz, $CDCl_3$) δ 176.26 (-NC=O), 143.94, 143.94, 133.88, 133.30, 131.36, 128.67, 127.17, 126.88, 121.71, 120.73, 108.00, 34.99, 33.28, 26.74, 22.47 ppm. The ee value was determined by HPLC analysis. Column (Chiral AD-H), UV 230 nm, eluent: Hexane/IPA = 60/1, Flow rate = 1.0 mL/min, tR = 20.4 min (major product), tR = 26.1 min (minor product). HRMS (DART) calcd for $C_{17}H_{15}ClNO$ $[M+H]^+$: 284.0842 found: 284.0842.

(1*R*,2*S*)-2-(4-Methoxyphenyl)-1'-methylspiro[cyclopropane-1,3'-indolin]-2'-one (3o)

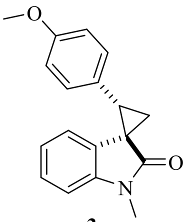

3o

This compound was prepared according to the typical procedure for asymmetric intermolecular cyclopropanation reaction of between 4-methoxystyrene **2k** (134.2 mg, 1mmol) and 3-diazo-1-methylindolin-2-one **1a** (34.6 mg, 0.2 mmol). The reaction mixture was purified by silica gel column chromatography with EtOAc/n-Hexane as an eluent to give **3o** in 79% yield as white solid (44.1 mg, 0.158 mmol). *trans/cis* = >99:1<, 97% *trans* ee. $[\alpha]^{25.2}_{D}$ = –133.4 (c 1.0, $CHCl_3$). ^{1}H NMR (500 MHz, $CDCl_3$) δ 7.15 (td, *J* = 7.74, 1.02 Hz, 1H), 7.09 (d, *J* = 8.60 Hz, 2H), 6.86 (d, *J* = 7.64 Hz, 1H), 6.81 (d, *J* = 8.60 Hz, 2H), 6.70 (t, *J* = 7.45 Hz, 1H), 5.99 (d, *J* = 7.64 Hz, 1H), 3.78 (s, 3H, Ar-OC**H$_3$**), 3.32 (s, 3H, -NC**H$_3$**), 3.27 (t, *J* = 8.41, 8.51 Hz, 1H, -C**H** (cyclopropane)), 2.16 (dd, *J* = 8.98, 4.40 Hz, 1H, -C**Hβ**H (cyclopropane)), 1.94 (dd, *J* = 7.84, 4.40 Hz, 1H, -CH**Hα** (cyclopropane)) ppm. ^{13}C NMR δ (100 MHz, $CDCl_3$) 176.61 (-NC=O), 158.87, 143.89, 131.08, 127.75, 127.31, 126.56, 121.57, 120.85, 113.83, 107.80, 55.34, 35.39, 33.43, 26.71, 22.89 ppm. The ee value was determined by HPLC analysis. Column (Chiral AD-3), UV 230 nm, eluent: Hexane/IPA = 60/1, Flow rate = 1.0 mL/min, tR = 28.7 min (major product), tR = 36.9 min (minor product). HRMS (DART) calcd for $C_{18}H_{18}NO_2$ $[M+H]^+$: 280.1337 found: 280.1336.

(1*R*,2*S*)-2-(4-(Dimethylamino)phenyl)-1'-methylspiro[cyclopropane-1,3'-indolin]-2'-one (3p)

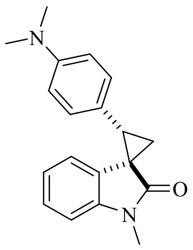

3p

This compound was prepared according to the typical procedure for asymmetric intermolecular cyclopropanation reaction of between N'N-dimethyl-4-vinylaniline **2l** (134.2 mg, 1 mmol) and 3-diazo-1-methylindolin-2-one **1a** (34.6 mg, 0.2 mmol). The reaction mixture was purified by silica gel column chromatography with EtOAc/n-Hexane as an eluent to give **3p** in 74% yield as white solid (44.1 mg, 0.158 mmol). *trans/cis* = >99:1<, 24% *trans* ee. $[\alpha]^{24.6}_D$ = –37.9 (c 1.0, $CHCl_3$). ^1H NMR (500 MHz, $CDCl_3$) δ 7.14 (td, *J* = 7.74, 1.02 Hz, 1H), 7.04 (d, *J* = 8.60 Hz, 2H), 6.85 (d, *J* = 7.58 Hz, 1H), 6.71 (td, *J* = 7.55, 0.89 Hz, 1H), 6.64 (d, *J* = 8.60 Hz, 2H), 6.07 (d, *J* = 7.26 Hz, 1H), 3.31 (s, 3H, -NC**H$_3$**), 3.26 (t, *J* = 8.51 Hz, 1H, -C**H** (cyclopropane)), 2.92 (s, 6H, Ar-N(**CH$_3$**)$_2$), 2.14 (dd, *J* = 9.17, 4.50 Hz, -C**Hβ**H (cyclopropane)), 1.96 (dd, *J* = 7.84, 4.50 Hz, -CH**Hα** (cyclopropane)) ppm. ^{13}C NMR (100 MHz, $CDCl_3$) δ 176.82 (-NC=O), 149.84, 143.85, 130.66, 128.11, 126.35, 122.79, 122.56, 120.94, 112.41, 107.69, 40.66, 35.85, 33.66, 26.69, 23.02 ppm. The ee value was determined by HPLC analysis. Column (Chiral AD-H), UV 230 nm, eluent: Hexane/IPA = 20/1, Flow rate = 1.0 mL/min, tR = 14.3 min (major product), tR = 16.2 min (minor product). HRMS (DART) calcd for $C_{19}H_{21}N_2O$ $[M+H]^+$: 293.1653 found: 293.1654.

(1R,2S)-5'-Bromo-1'-methyl-2-phenylspiro[cyclopropane-1,3'-indolin]-2'-one (3q)

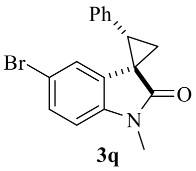

3q

This compound was prepared according to the typical procedure for asymmetric intermolecular cyclopropanation reaction of between styrene **2a** (104.2 mg, 1.0 mmol) and 5-bromo-3-diazo-1-methylindolin-2-one **1f** (50.4 mg, 0.2 mmol). The reaction mixture was purified by silica gel column chromatography with EtOAc/n-Hexane as an eluent to give **3q** in 93% yield as red oil (61.05mg, 0.188 mmol). *trans/cis* = 89:11, 87% *trans* ee. $[\alpha]^{24}_D$ = 80 (c 0.9, $CHCl_3$). ^1H NMR (500 MHz, $CDCl_3$) δ 7.35–7.23 (m, 4H), 7.19–7.13 (m, 2H), 6.72 (d, *J* = 8.03 Hz, 1H), 6.02 (d, *J* = 1.91 Hz,, 1H), 3.37 (t, *J* = 8.41 Hz, 1H, -C**H** (cyclopropane)), 3.30 (s, 3H, -NC**H$_3$**), 2.21 (dd, *J* = 8.79, 4.59 Hz, 1H, -C**Hβ**H (cyclopropane)), 2.01 (dd, *J* = 8.03, 4.59 Hz, 1H, -CH**Hα** (cyclopropane)) ppm. ^{13}C NMR (100 MHz, $CDCl_3$) δ 176.00 (-NC=O), 142.91, 134.59, 129.92, 129.79, 129.40, 128.65, 127.87, 123.92, 114.32, 109.16, 36.47, 33.40, 26.85, 22.96. The ee value was determined by HPLC analysis. Column (Chiral IE-3), UV 230 nm, eluent: Hexane/IPA = 15/1, Flow rate = 1.0 mL/min, tR = 14.5 min (major product), tR = 15.7 min (minor product). HRMS (DART) calcd for $C_{17}H_{15}BrNO$ $[M+H]^+$: 328.0337 found: 328.0337.

(1*R*,2*S*)-6'-Chloro-1'-methyl-2-phenylspiro[cyclopropane-1,3'-indolin]-2'-one (3r)

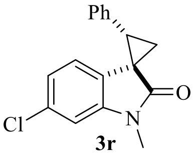

3r

This compound was prepared according to the typical procedure for asymmetric intermolecular cyclopropanation reaction of between styrene **1a** (104.2 mg, 1 mmol) and 6-chloro-3-diazo-1-methylindolin-2-one **1g** (41.5 mg, 0.2 mmol). The reaction mixture was purified by silica gel column chromatography with EtOAc/n-Hexane as an eluent to give **3r** in 98% yield as yellow oil (55.6 mg, 0.196 mmol). *trans/cis* = 96:4, 99% *trans* ee. $[\alpha]^{25.1}_D$ = –104.3 (c 1.0, $CHCl_3$). ^1H NMR (500 MHz, $CDCl_3$) δ 7.32–7.23 (m, 3H), 7.16 (d, *J* = 6.88 Hz, 1H), 6.85 (d, *J* = 1.91 Hz, 1H), 6.64 (td, *J* = 8.03, 1.91 Hz, 1H), 5.83 (d, *J* = 8.03 Hz, 1H), 3.34 (t, *J* = 8.60 Hz, 1H, -C**H** (cyclopropane)), 3.30 (s, 3H, -NC**H$_3$**), 2.20 (dd, *J* = 9.17, 4.59 Hz, 1H, -C**Hβ**H (cyclopropane)), 2.00 (dd, *J* = 8.03, 4.59 Hz, 1H, -CH**Hα** (cyclopropane)) ppm. ^{13}C NMR (100 MHz, $CDCl_3$) δ 176.49 (-NC=O), 145.01, 134.91, 132.59, 129.95, 128.58, 127.68, 125.99,

121.48, 121.41, 108.62, 36.20, 33.19, 26.85, 22.64. The ee value was determined by HPLC analysis. Column (Chiral IF-3), UV 230 nm, eluent: Hexane/IPA = 80/1, Flow rate = 1.0 mL/min, tR = 27.2 min (major product), tR = 69.6 min (minor product). HRMS (DART) calcd for $C_{17}H_{15}ClNO$ $[M+H]^+$: 284.0842 found: 284.0842.

(1*R*,2*S*)-6'-Methoxy-1'-methyl-2-phenylspiro[cyclopropane-1,3'-indolin]-2'-one (3s)

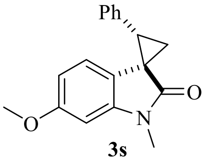

3s

The solution of diazooxindole (0.2 mmol) in toluene:CH_2Cl_2 = 1:1 (2 mL) was slowly added to a mixture of Ru(II)-Pheox **6e** (0.002 mmol) and olefins (1.0 mmol) in toluene:CH_2Cl_2 = 1:1 (2 mL) for 2 min under argon atmosphere at 0 °C.This compound was prepared according to the typical procedure for asymmetric intermolecular cyclopropanation reaction of between styrene **2a** (104.2 mg, 1 mmol) and 3-diazo-6-methoxy-1-methylindolin-2-one **1h** (40.6 mg, 0.2 mmol). The reaction mixture was purified by silica gel column chromatography with EtOAc/n-Hexane as an eluent to give **3s** in 93% yield as red solid (52 mg, 0.186 mmol). *trans/cis* = 98:2, 95% *trans* ee. $[\alpha]^{25.4}_{D}$ = –94.4 (c 1.0, $CHCl_3$). ^1H NMR (500 MHz, $CDCl_3$) δ 7.32–7.22 (m, 3H), 7.18 (d, *J* = 6.88 Hz, 2H), 6.47 (d, *J* = 2.29 Hz, 1H), 6.20 (td, *J* = 8.41, 2.29 Hz, 1H), 5.84 (d, *J* = 8.41 Hz, 1H), 3.74 (s, 3H, Ar-OC**H$_3$**), 3.29 (s, 3H, -NC**H$_3$**), 3.26 (t, *J* = 8.51 Hz, 1H, -C**H** (cyclopropane)), 2.12 (8.98, 4.50 Hz, 1H, -C**Hβ**H (cyclopropane)), 1.92 (dd, *J* = 8.03, 4.50 Hz, 1H, -CH**Hα** (cyclopropane)) ppm. ^{13}C NMR (100 MHz, $CDCl_3$) δ 177.25 (-NC=O), 159.41, 145.08, 135.52, 130.01, 128.44, 127.38, 121.26, 119.46, 105.56, 96.20, 55.55, 35.20, 32.99, 26.76, 21.99. The ee value was determined by HPLC analysis. Column (Chiral IE-3), UV 230 nm, eluent: Hexane/IPA =, Flow rate = 1.0 mL/min, tR = 39.4 min (major product), tR = 37.6 min (minor product). HRMS (DART) calcd for $C_{18}H_{18}NO_2$ $[M+H]^+$: 280.1337 found: 280.1337.

(1*S*,2*R*)-1'-Methyl-2'-oxospiro[cyclopropane-1,3'-indolin]-2-yl acetate (3t)

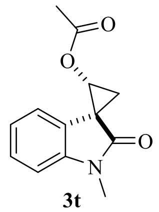

3t

The solution of diazooxindole (0.2 mmol) in toluene:CH_2Cl_2 = 1:1 (2 mL) was slowly added to a mixture of Ru(II)-Pheox **6e** (0.002 mmol) and olefins (1.0 mmol) in toluene:CH_2Cl_2 = 1:1 (2 mL) for 4 h under argon atmosphere at 0 °C. This compound was prepared according to the typical procedure for asymmetric intermolecular cyclopropanation reaction of between vinyl acetate **2m** (86.1 mg, 1 mmol) and 3-diazo-1-methylindolin-2-one **1a** (34.6 mg, 0.2 mmol). The reaction mixture was purified by silica gel column chromatography with EtOAc/n-Hexane as an eluent to give **3t** in 84% yield as yellow solid (38.9 mg, 0.168 mmol). *trans/cis* = 98:2, 90% *trans* ee. $[\alpha]^{23.6}_{D}$ = +142.0 (c 1.0, $CHCl_3$). ^1H NMR (500 MHz, $CDCl_3$) δ 7.28 (td, *J* = 7.74, 1.28 Hz, 1H), 7.02 (td, *J* =7.55, 0.89 Hz, 1H), 6.96 (dd, *J* = 7.26, 0.76 Hz), 6.90 (d, *J* = 7.64 Hz, 1H), 4.73 (dd, *J* = 7.07, 5.16 Hz, 1H, -C**H** (cyclopropane)), 3.28 (s, 3H, -NC**H$_3$**), 2.11 (t, *J* = 6.69 Hz, 1H, -C**Hβ**H (cyclopropane)), 2.03 (s, 3H, CO-C**H$_3$**), 1.87 (dd, *J* = 6.31, 5.16 Hz, -CH**Hα** (cyclopropane)) ppm. ^{13}C NMR (125 MHz, $CDCl_3$) δ 174.70, 170.23, 144.38, 127.49, 126.08, 122.0, 120.61, 108.47, 60.17, 32.29, 26.71, 21.07, 20.54 ppm. The ee value was determined by HPLC analysis. Column (Chiral IE-3), UV 230 nm, eluent: Hexane/IPA = 8/1, Flow rate = 1.0 mL/min, tR = 40.8 min (major product), tR = 51.0 min (minor product). HRMS (DART) calcd for $C_{13}H_{14}NO_3$ $[M+H]^+$: 232.0973 found: 232.0970.

(1*S*,2*R*)-1'-Methyl-2'-oxospiro[cyclopropane-1,3'-indolin]-2-yl benzoate (3u)

The solution of diazooxindole (0.2 mmol) in toluene:CH_2Cl_2 = 1:1 (2 mL) was slowly added to a mixture of

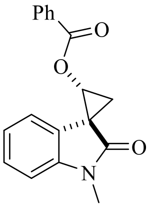

3u

Ru(II)-Pheox **6e** (0.002 mmol) and olefins (1.0 mmol) in toluene:CH_2Cl_2 = 1:1 (2 mL) for 4 h under argon atmosphere at 0 °C. This compound was prepared according to the typical procedure for asymmetric intermolecular cyclopropanation reaction of between vinyl benzoate **2n** (148.2 mg, 1 mmol) and 3-diazo-1-methylindolin-2-one **1a** (34.6 mg, 0.2 mmol). The reaction mixture was purified by silica gel column chromatography with EtOAc/n-Hexane as an eluent to give **3u** in 85% yield as white solid (49.9 mg, 0.17 mmol). *trans/cis* = >99:1<, 92% *trans* ee. $[\alpha]^{22.5}_{D}$ = –122.4 (c 1.0, $CHCl_3$). ^{1}H NMR (500 MHz, $CDCl_3$) δ 7.99 (td, *J* = 8.22, 1.34 Hz, 2H), 7.58 (t, *J* = 7.45 Hz, 1H), 7.44–7.41 (m, 2H), 7.23 (td, *J* = 7.74, 1.28 Hz, 1H), 6.96 (d, *J* = 7.26 Hz, 1H), 6.91 (t, *J* = 7.74 Hz, 2H), 4.96 (dd, *J* = 6.88, 4.97 Hz, 1H, -C**H** (cyclopropane)), 3.30 (s, 3H, -NC**H3**), 2.23 (t, *J* = 6.69 Hz, 1H, -C**Hβ**H (cyclopropane)), 2.01 (dd, *J* = 6.50, 4.97 Hz, 1H, -CH**Hα** (cyclopropane)) ppm. ^{13}C NMR (100 MHz, $CDCl_3$) δ 174.75, 165.93, 144.38, 133.63, 129.72, 129.03, 128.69, 127.45, 126.04, 122.09, 120.87, 108.44, 60.69, 32.42, 26.73, 21.55 ppm. The ee value was determined by HPLC analysis. Column (Chiral IF-3), UV 230 nm, eluent: Hexane/IPA = 8/1, Flow rate = 1.0 mL/min, tR = 19.0 min (major product), tR = 22.9 min (minor product). HRMS (DART) calcd for $C_{18}H_{16}NO_3$ $[M+H]^+$: 294.1130 found: 294.1134.

(1*S*,2*R*)-2-(9H-Carbazol-9-yl)-1'-methylspiro[cyclopropane-1,3'-indolin]-2'-one (3v)

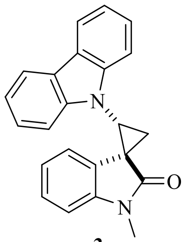

3v

This compound was prepared according to the typical procedure for asymmetric intermolecular cyclopropanation reaction of between 9-vinylcarbazole **2o** (193.3 mg, 1mmol) and 3-diazo-1-methylindolin-2-one **1a** (34.6 mg, 0.2 mmol). The reaction mixture was purified by silica gel column chromatography with EtOAc/n-Hexane as an eluent to give **3v** in 92% yield as white solid (62.3 mg, 0.184 mmol). *trans/cis* = 92:8, 78% *trans* ee. $[\alpha]^{25.4}_{D}$ = –37.7 (c 1.0, $CHCl_3$). ^{1}H NMR (500 MHz, $CDCl_3$) δ 8.10 (d, *J* = 8.03 Hz, 1H) , 7.95 (d, *J* = 7.64 Hz, 1H), 7.76 (d, *J* = 8.03 Hz, 1H), 7.52 (td, *J* = 7.84, 1.15 Hz, 1H), 7.31 (t, *J* = 7.07 Hz, 1H), 7.06 (td, *J* = 7.64, 0.76 Hz, 2H), 7.01 (t, *J* = 7.55 Hz, 1H), 6.89 (d, *J* = 7.64 Hz, 1H), 6.55 (d, *J* = 8.03 Hz, 1H), 6.41 (dd, *J* = 7.07 Hz, 1H), 5.72 (d, *J* = 7.40 Hz, 1H), 4.27 (dd, *J* = 7.64, 6.12, 1H, -C**H** (cyclopropane)), 3.44 (s, 3H, -NC**H3**), 2.68 (dd, *J* = 7.64, 5.35 Hz, 1H, -C**Hβ**H (cyclopropane)), 2.53 (t, *J* = 5.74 Hz, 1H, -CH**Hα** (cyclopropane)) ppm. ^{13}C NMR (100 MHz, $CDCl_3$) δ 174.90 (-NC=O), 143.57, 141.09, 140.69, 127.36, 126.18, 126.05, 125.75, 123.75, 123.03, 122.22, 120.77, 120.50, 120.10, 119.98, 110.13, 110.03, 108.13, 41.00, 33.65, 27.00, 23.48 ppm. The ee value was determined by HPLC analysis. Column (Chiral IF-3), UV 230 nm, eluent: Hexane/IPA = 10/1, Flow rate = 1.1 mL/min, tR = 13.4 min (major product), tR = 23.3 min (minor product). HRMS (DART) calcd for $C_{23}H_{19}N_2O$ $[M+H]^+$: 339.1497 found: 339.1495.

2-((1*S*,2*R*)-1'-Methyl-2'-oxospiro[cyclopropane-1,3'-indolin]-2-yl)isoindoline-1,3-dione (3w)

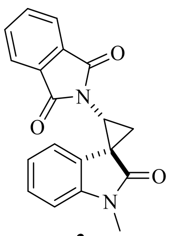

3w

The solution of diazooxindole (0.2 mmol) in toluene:CH_2Cl_2 = 1:1 (2 mL) was slowly added to a mixture of Ru(II)-Pheox **6e** (0.002 mmol) and olefins (1.0 mmol) in toluene:CH_2Cl_2 = 1:1 (2 mL) for 4 h under argon atmosphere at 0 °C. This compound was prepared according to the typical procedure for asymmetric intermolecular cyclopropanation reaction of between N-vinylphthalimide **2p** (173.2 mg, 1 mmol) and 3-diazo-1-methylindolin-2-one **1a** (34.6 mg, 0.2 mmol). The reaction mixture was purified by silica gel column chromatography with EtOAc/n-Hexane as an eluent to give **3w** in 37% yield as white solid (23.6 mg, 0.074 mmol). *trans/cis* = 86:14, 93% *trans* ee. $[\alpha]^{25.1}_{D}$ = –156.7 (c 0.9, $CHCl_3$). ^{1}H NMR (500 MHz, $CDCl_3$) δ 7.86–7.77 (m, 2H), 7.76–7.69 (m, 2H), 7.20 (td, *J* = 7.84, 1.15 Hz, 1H), 6.91 (d, *J* = 8.03 Hz, 1H), 6.76 (td, *J* = 7.55, 0.89 Hz, 1H), 6.47 (dd, *J* = 7.64, 0.76 Hz, 1H), 3.62 (dd, *J* = 8.22, 6.41 Hz, 1H, -C**H** (cyclopropane)), 3.34 (s, 3H, -NC**H₃**), 2.64 (t, *J* = 8.32 Hz, 1H, -C**Hβ**H (cyclopropane)), 2.41 (dd, *J* = 8.41, 6.41 Hz, 1H, -CH**Hα** (cyclopropane)) ppm. ^{13}C NMR (100 MHz, $CDCl_3$) δ 174.93 (-NC=O), 167.99, 144.29, 134.55, 131.34, 127.58, 126.09, 123.68, 122.11, 119.20, 108.56, 37.64, 32.18, 26.85, 19.84 ppm. The ee value was determined by HPLC analysis. Column (Chiral IA-3), UV 230 nm, eluent: Hexane/IPA = 8/1, Flow rate = 0.8 mL/min, tR = 25.8 min (major product), tR = 34.3 min (minor product). HRMS (DART) calcd for $C_{19}H_{15}N_2O_3$ $[M+H]^+$: 319.1082 found: 319.1085.

4. Ssynthesis of Bioactive Compound.

(1*R*,2*S*)-5'-Fluoro-2-phenylspiro[cyclopropane-1,3'-indolin]-2'-one (4a) [7], [8]

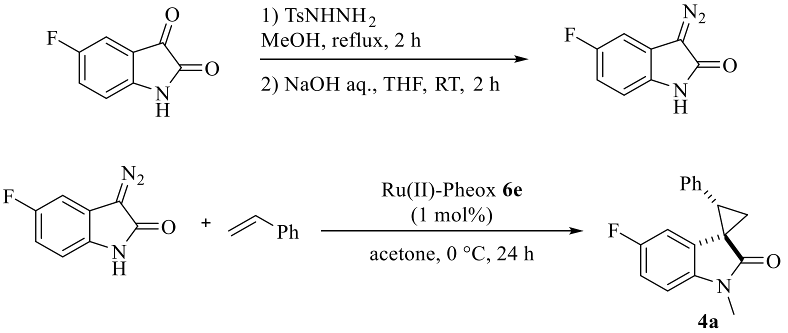

4a

Using the procedure of diazooxindoles, 5-fluoroisatin (102.4 mg, 0.62 mmol, 1 equiv.) and tosylhydrazine (127.0 mg, 0.682 mmol, 1.1 equiv.) were dissolved in MeOH (5 mL). The reaction mixture was refluxed for 2 h, the reaction mixture was concentrated under reduced pressureand and filtered off. The residue was suspended in CH_2Cl_2:H_2O = 1:1 (4 mL) and treated with 0.6M NaOH water solution (2.06 mL, 1.24 mmol). The reaction mixture was stirred for 2 h at 45 °C, and then allowed to reach room temperature. The mixture was neutralized by addition of dry-ice, diluted with brine and extracted with EtOAc. The combined organic layers were dried over Na_2SO_4, filtered and concentrated. The residue was purified by flash column chromatography with Hexane/EtOAc to give 3-diazo-5-fluoroindolin-2-one as red solid (63% yield, 69.1 mg, 0.391 mmol).

The solution of 3-diazo-5-fluoroindolin-2-one (35.4 mg, 0.2 mmol) in acetone (2 mL) was slowly added to a

mixture of Ru(II)-Pheox **6e** (0.002 mmol) and styrene (104.2 mg, 1.0 mmol) in acetone (2 mL) for 2 min under argon atmosphere at 0 °C. After the addition completed, the reaction mixture was then stirred for 24 h at 0 °C. The reaction mixture was purified by silica gel column chromatography with EtOAc/n-Hexane as an eluent to give 4**a** in 82% yield as brown solid (41.5 mg, 0.164 mmol). *trans/cis* = >99:1<, 95% *trans* ee . $[\alpha]^{25.9}_{D}$ = –136.0 (c 1.0, $CHCl_3$). ^{1}H NMR (500 MHz, $CDCl_3$) δ 8.89 (s, 1H, -N**H**), 7.37–7.22 (m, 3H), 7.19 (d, *J* = 6.50 Hz, 2H), 6.86 (td, *J* = 8.41, 4.59 Hz, 1H), 6.76 (dd, *J* = 8.89, 2.49 Hz, 1H), 5.67 (dd, *J* = 8.60, 2.49 Hz, 1H), 3.39 (t, *J* = 8.60 Hz, 1H, -C**H** (cyclopropane)), 2.25 (dd, *J* = 9.17, 4.78 Hz, 1H, -C**Hβ**H (cyclopropane)), 2.03 (dd, *J* = 8.03, 4.78 Hz, -CH**Hα** (cyclopropane)) ppm. ^{13}C NMR (125 MHz, $CDCl_3$) δ 178.82 (-NC=O), 158.46 (d, *J* = 238.66 Hz), 136.96, 134.3, 129.95 (2C), 129.86, 128.73 (2C), 127.95, 113.05 (d, *J* = 23.96 Hz), 110.09 (d, *J* = 8.63 Hz), 36.70, 34.31, 23.18 ppm. The ee value was determined by HPLC analysis. Column (Chiral AD-H), UV 230 nm, eluent: Hexane/IPA = 20/1, Flow rate = 1.0 mL/min, tR = 15.1 min (major product), tR = 18.7 min (minor product). HRMS (DART) calcd for $C_{16}H_{13}FNO$ $[M+H]^+$: 254.0981 found: 254.0981.

3-(((1*R*,2*S*)-2-(4-Chlorophenyl)-2'-oxospiro[cyclopropane-1,3'-indolin]-1'-yl)methyl)benzoic acid (4b, 4c) [1], [2], [9]

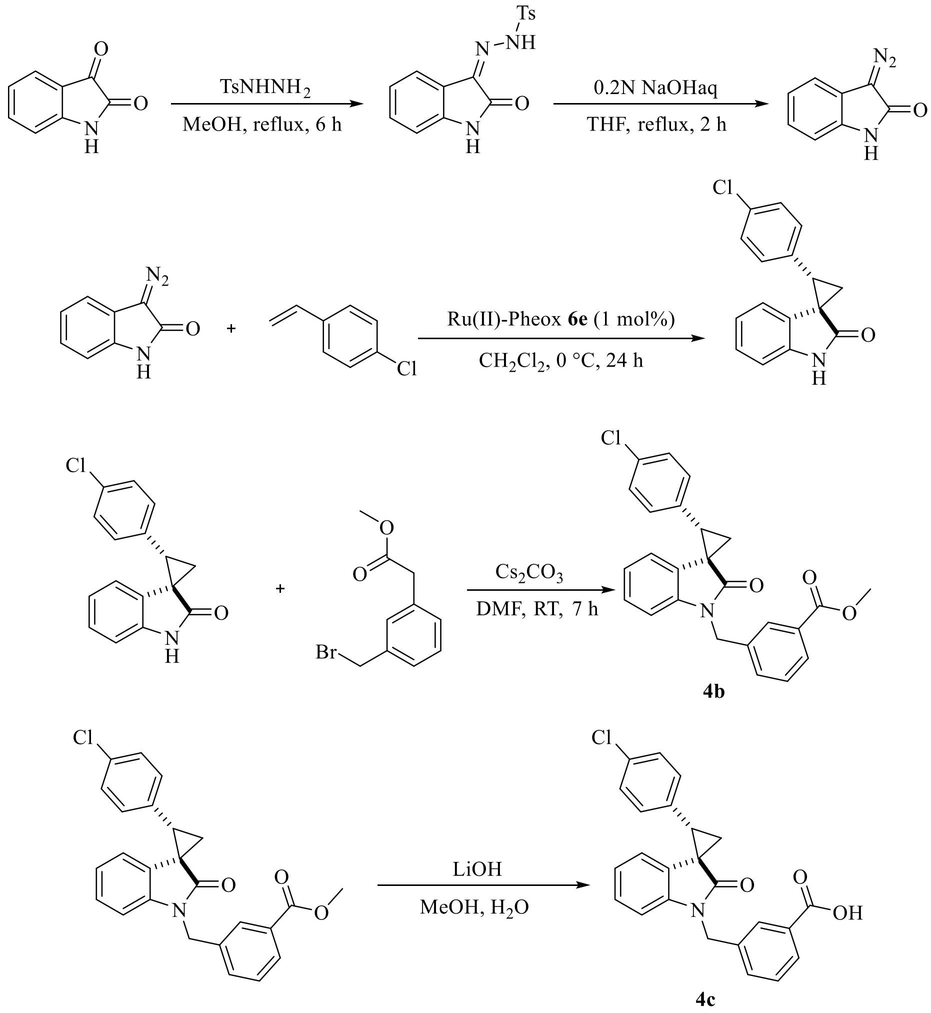

Using the procedure of diazooxindoles, isatin (245.7 mg, 1.67 mmol, 1 equiv.) and tosylhydrazine (342.7 mg, 1.84

mmol, 1.1 equiv.) were dissolved in MeOH (10 mL). The reaction mixture was refluxed for 6 h, the reaction mixture was concentrated under reduced pressureand and filtered off. The residue was suspended in THF (15 mL) and treated with 0.2M NaOH water solution (16.7 mL, 3.34 mmol). The reaction mixture was stirred for 2 h at 45 °C, and then allowed to reach room temperature. The mixture was neutralized by addition of dry-ice, diluted with brine and extracted with EtOAc. The combined organic layers were dried over Na_2SO_4, filtered and concentrated. The residue was purified by flash column chromatography with Hexane/EtOAc to give 3-diazoindolin-2-one as pale-orange solid (82% yield, 217.9 mg, 1.369 mmol).

The solution of 3-diazoindolin-2-one (95.5 mg, 0.6 mmol) in CH_2Cl_2 (4 mL) was slowly added to a mixture of Ru(II)-Pheox **6e** (0.006 mmol) and 4-chlorostyrene (415.8 mg, 3.0 mmol) in CH_2Cl_2 (5 mL) for 2 min under argon atmosphere at 0 °C. After the addition completed, the reaction mixture was then stirred for 24 h at 0 °C. The reaction mixture was purified by silica gel column chromatography with EtOAc/n-Hexane as an eluent to give the (1*R*,2*S*)-2-(4-chlorophenyl)spiro[cyclopropane-1,3'-indolin]-2'-one in 93% yield as white solid (150.5 mg, 0.56 mmol). *trans/cis* = 96:4, 94% *trans* ee. $[\alpha]^{26.0}_D$ = –187.3 (c 0.9, $CHCl_3$). ^1H NMR (500 MHz, $CDCl_3$) δ 8.52 (s, 1H, -N**H**), 7.33–7.23 (m, 3H), 7.17–7.08 (m, 3H), 6.95 (d, *J* = 7.64 Hz, 1H), 6.71 (td, *J* = 7.52, 1.02 Hz, 1H), 5.96 (d, *J* = 7.64 Hz, 1H), 3.28 (t, *J* = 8.51 Hz, 1H, -C**H** (cyclopropane)), 2.22 (dd, *J* = 9.17, 4.69 Hz, 1H), 2.25 (dd, *J* = 9.17, 4.78 Hz, 1H, -C**Hβ**H (cyclopropane)), 1.96 (dd, *J* = 7.84, 4.69 Hz, 1H, -CH**Hα** (cyclopropane)) ppm. ^{13}C NMR (100 MHz, $CDCl_3$) δ 178.35 (-NC=O), 141.00, 133.70, 133.46, 131.43, 128.76, 127.62, 126.94, 121.79, 121.14, 109.82, 35.40, 33.70, 22.77 ppm. The ee value was determined by HPLC analysis. Column (Chiral AD-H), UV 230 nm, eluent: Hexane/IPA = 20/1, Flow rate = 1.0 mL/min, tR = 17.1 min (major product), tR = 21.5 min (minor product). HRMS (DART) calcd for $C_{16}H_{13}ClNO$ $[M+H]^+$: 270.0685 found: 270.0685.

(1*R*,2*S*)-2-(4-chlorophenyl)spiro[cyclopropane-1,3'-indolin]-2'-one (99.8 mg, 0.37 mmol), methyl-(3-bromomethyl)-benzoate (136.1 mg, 0.56 mmol) and Cs_2CO_3 (247.6 mg, 0.74 mmol) were mixture in anhydrous DMF (8 mL) and stirred at room temperature for 7 h. The mixture was extracted with Et_2O. The combined organic layers were dried over Na_2SO_4, filtered and concentrated. The residue was purified by flash column chromatography with Hexane/EtOAc to give the methyl 3-(((1*R*,2*S*)-2-(4-chlorophenyl)-2'-oxospiro[cyclopropane-1,3'-indolin]-1'-yl)methyl)benzoate **4b** in 99% yield as white solid (153.1 mg, 0.366 mmol). *trans/cis* = >99:1<, 93% *trans* ee. $[\alpha]^{25.9}_D$ = –143.4 (c 1.0, $CHCl_3$). ^1H NMR (500 MHz, $CDCl_3$) δ 8.02 (s, 1H), 7.95 (d, *J* = 8.03 Hz, 1H), 7.51 (d, *J* = 7.64 Hz, 1H), 7.41 (t, *J* = 7.64 Hz, 1H), 7.27 (d, *J* = 8.41 Hz, 2H), 7.14 (d, *J* = 8.41 Hz, 2H), 7.05 (t, *J* = 7.84 Hz, 1H), 6.74 (d, *J* = 8.03 Hz, 1H), 6.70, (t, *J* = 7.64 Hz, 1H), 5.98 (d, *J* = 7.64 Hz, 1H), 5.10 (d, *J* = 16.00 Hz, 1H, -N**H**HAr), 5.04 (d, *J* = 16.00 Hz, 1H, -NH**H**Ar), 3.91 (s, 3H, COOC**H$_3$**), 3.35 (t, *J* = 8.51 Hz, 1H, -C**H** (cyclopropane)), 2.29 (dd, *J* = 9.17, 4.69 Hz, 1H, -C**Hβ**H (cyclopropane)), 2.00 (dd, *J* = 7.84, 4.69 Hz, 1H, -CH**Hα** (cyclopropane)) ppm. ^{13}C NMR (125 MHz, $CDCl_3$) δ 176.45 (-NC=O), 166.85, 142.76, 136.75, 133.74, 133.43, 131.87, 131.42, 130.81, 129.13, 129.03, 128.75, 128.48, 127.15, 126.89, 121.93, 120.94, 108.88, 52.33, 43.99, 35.56, 33.28, 22.76 ppm. The ee value was determined by HPLC analysis. Column (Chiral AD-H), UV 230 nm, eluent: Hexane/IPA = 10/1, Flow rate = 1.0 mL/min, tR = 19.8 min (major product), tR = 23.5 min (minor product). HRMS (DART) calcd for $C_{25}H_{21}ClNO_3$

$[M+H]^+$: 418.1210 found: 418.1210.

Methyl-3-(((1*R*,2*S*)-2-(4-chlorophenyl)-2'-oxospiro[cyclopropane-1,3'-indolin]-1'-yl)methyl)benzoate (54.3 mg, 0.13 mmol) was dissolved in 4 mL of methanol; then 0.4 mL of water was added followed by LiOH (11.7 mg, 0.49 mmol). The mixture was stirred for 22 hours at 40 °C. The mixture was extracted with Et_2O. The combined organic layers were dried over Na_2SO_4, filtered and concentrated. The reaction mixture was purified by silica gel column chromatography with CH_2Cl_2/MeOH as an eluent to give **4c** in 98% yield as white solid. *trans/cis* = >99:1<, $[\alpha]^{27.2}_{D}$ = –153.8 (c 0.9, $CHCl_3$). ^1H NMR (500 MHz, $CDCl_3$) δ 8.06 (s, 1H), 8.03 (d, *J* = 8.03 Hz, 1H), 7.58 (d, *J* = 7.64 Hz, 1H), 7.46 (t, *J* = 7.84 Hz, 1H), 7.29 (d, *J* = 8.61 Hz, 2H), 7.15 (d, *J* = 8.61 Hz, 2H), 7.06 (td, *J* = 7.64, 1.15 Hz, 1H), 6.75 (d, *J* = 7.64 Hz, 1H), 6.71 (t, *J* = 7.84 Hz, 1H), 6.00 (d, *J* = 7.64 Hz, 1H), 5.10 (s, 2H, -NC**H₂**Ar), 3.37 (t, *J* = 8.51 Hz, 1H, -C**H** (cyclopropane)), 2.32 (dd, *J* = 8.98, 4.69 Hz, 1H, -C**Hβ**H (cyclopropane)), 2.02 (dd, *J* = 8.03, 4.69 Hz, 1H, -CH**Hα** (cyclopropane)) ppm. ^{13}C NMR (100 MHz, $CDCl_3$) δ 176.65 (-NC=O), 171.51, 142.65, 136.88, 133.71, 133.45, 132.71, 131.45, 130.01, 129.67, 129.31, 128.93, 128.80, 127.17, 126.96, 122.07, 120.99, 108.93, 43.90, 35.77, 33.41, 22.73 ppm. HRMS (DART) calcd for $C_{24}H_{19}ClNO_3$ $[M+H]^+$: 404.1053 found: 404.1053.

5. X-ray Crystal Structure

(1*R*,2*S*)-2-(4-Bromophenyl)-1'-methylspiro[cyclopropane-1,3'-indolin]-2'-one (**3m**)

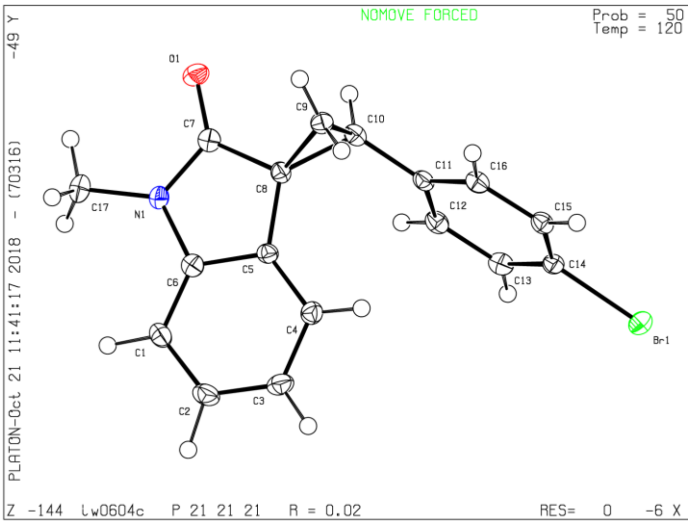

Table S1. Crystal data and structure refinement.

Empirical formula	C17 H14 Br N O	
Formula weight	328.20	
Temperature	120 K	
Wavelength	0.71075 Å	
Crystal system	Orthorhombic	
Space group	$P2_12_12_1$	
Unit cell dimensions	*a* = 6.0888(8) Å	α= 90°.
	b = 12.5645(17) Å	β= 90°.

	c = 17.867(2) Å	γ = 90°.
Volume	1366.9(3) $Å^3$	
Z	4	
Density (calculated)	1.595 Mg/m^3	
Absorption coefficient	3.001 mm^{-1}	
F(000)	664	
Crystal size	0.400 x 0.070 x 0.070 mm^3	
Theta range for data collection	1.982 to 30.033°.	
Index ranges	-8<=h<=8, -17<=k<=17, -24<=l<=24	
Reflections collected	29902	
Independent reflections	3974 [R(int) = 0.0323]	
Completeness to theta = 25.242°	98.9 %	
Absorption correction	Numerical	
Max. and min. transmission	0.677 and 0.267	
Refinement method	Full-matrix least-squares on F^2	
Data / restraints / parameters	3974 / 0 / 226	
Goodness-of-fit on F^2	0.991	
Final R indices [I>2sigma(I)]	R1 = 0.0207, wR2 = 0.0481	
R indices (all data)	R1 = 0.0228, wR2 = 0.0488	
Absolute structure parameter	-0.018(2)	
Extinction coefficient	n/a	
Largest diff. peak and hole	0.964 and -0.278 e.$Å^{-3}$	

Table S2. Atomic coordinates (x 10^4) and equivalent isotropic displacement parameters ($Å^2$x 10^3). U(eq) is defined as one third of the trace of the orthogonalized U^{ij} tensor.

	x	y	z	U(eq)
C(1)	11380(4)	13224(2)	11278(1)	19(1)
C(2)	9556(4)	13468(2)	10839(1)	22(1)
C(3)	8103(4)	12688(2)	10603(1)	21(1)
C(4)	8419(4)	11620(2)	10809(1)	18(1)
C(5)	10225(3)	11363(1)	11239(1)	14(1)
C(6)	11684(3)	12169(2)	11466(1)	16(1)
C(7)	13211(3)	10638(2)	11929(1)	16(1)
C(8)	11101(3)	10353(2)	11547(1)	15(1)
C(9)	9773(4)	9492(2)	11945(1)	17(1)
C(10)	10786(3)	9238(2)	11211(1)	16(1)

C(11)	9407(3)	9091(2)	10533(1)	15(1)
C(12)	10161(4)	9351(1)	9821(1)	17(1)
C(13)	8898(4)	9199(2)	9187(1)	18(1)
C(14)	6800(4)	8776(2)	9269(1)	16(1)
C(15)	5991(4)	8499(2)	9968(1)	16(1)
C(16)	7287(3)	8666(2)	10594(1)	16(1)
Br(1)	4975(1)	8599(1)	8416(1)	19(1)
O(1)	14480(2)	10037(1)	12256(1)	21(1)
N(1)	13413(3)	11722(1)	11884(1)	16(1)
C(17)	15088(4)	12345(2)	12271(1)	21(1)

Table S3. Bond lengths [Å] and angles [°].

C(1)-C(6)	1.381(3)
C(1)-C(2)	1.394(3)
C(2)-C(3)	1.385(3)
C(3)-C(4)	1.405(3)
C(4)-C(5)	1.380(3)
C(5)-C(6)	1.407(3)
C(5)-C(8)	1.483(3)
C(6)-N(1)	1.408(3)
C(7)-O(1)	1.228(3)
C(7)-N(1)	1.370(3)
C(7)-C(8)	1.498(3)
C(8)-C(9)	1.526(3)
C(8)-C(10)	1.536(3)
C(9)-C(10)	1.484(3)
C(10)-C(11)	1.485(3)
C(11)-C(12)	1.392(3)
C(11)-C(16)	1.402(3)
C(12)-C(13)	1.382(3)
C(13)-C(14)	1.392(3)
C(14)-C(15)	1.386(3)
C(14)-Br(1)	1.899(2)
C(15)-C(16)	1.385(3)
N(1)-C(17)	1.459(3)

C(6)-C(1)-C(2)	117.1(2)
C(3)-C(2)-C(1)	121.7(2)
C(2)-C(3)-C(4)	120.5(2)
C(5)-C(4)-C(3)	118.6(2)
C(4)-C(5)-C(6)	119.69(18)
C(4)-C(5)-C(8)	133.95(19)
C(6)-C(5)-C(8)	106.35(18)
C(1)-C(6)-C(5)	122.4(2)
C(1)-C(6)-N(1)	127.8(2)
C(5)-C(6)-N(1)	109.80(18)
O(1)-C(7)-N(1)	125.6(2)
O(1)-C(7)-C(8)	127.6(2)
N(1)-C(7)-C(8)	106.74(17)
C(5)-C(8)-C(7)	105.85(16)
C(5)-C(8)-C(9)	126.08(18)
C(7)-C(8)-C(9)	114.32(18)
C(5)-C(8)-C(10)	126.17(18)
C(7)-C(8)-C(10)	120.21(17)
C(9)-C(8)-C(10)	57.97(13)
C(10)-C(9)-C(8)	61.37(14)
C(9)-C(10)-C(11)	120.84(19)
C(9)-C(10)-C(8)	60.66(13)
C(11)-C(10)-C(8)	120.21(18)
C(12)-C(11)-C(16)	117.7(2)
C(12)-C(11)-C(10)	122.00(19)
C(16)-C(11)-C(10)	120.30(19)
C(13)-C(12)-C(11)	122.2(2)
C(12)-C(13)-C(14)	118.5(2)
C(15)-C(14)-C(13)	121.1(2)
C(15)-C(14)-Br(1)	119.04(16)
C(13)-C(14)-Br(1)	119.83(16)
C(16)-C(15)-C(14)	119.1(2)
C(15)-C(16)-C(11)	121.3(2)
C(7)-N(1)-C(6)	111.11(17)
C(7)-N(1)-C(17)	124.62(18)
C(6)-N(1)-C(17)	124.07(17)

Symmetry transformations used to generate equivalent atoms:

Table S4. Anisotropic displacement parameters ($Å^2$x 10^3) for C:cc. The anisotropic displacement factor exponent takes the form: $-2\pi^2[h^2 a^{*2}U^{11} + ... + 2 h k a^* b^* U^{12}]$

	U^{11}	U^{22}	U^{33}	U^{23}	U^{13}	U^{12}
C(1)	23(1)	15(1)	20(1)	-2(1)	1(1)	-2(1)
C(2)	31(1)	15(1)	22(1)	-1(1)	0(1)	6(1)
C(3)	22(1)	21(1)	19(1)	1(1)	-3(1)	7(1)
C(4)	18(1)	18(1)	18(1)	-2(1)	-2(1)	2(1)
C(5)	17(1)	13(1)	13(1)	-1(1)	0(1)	3(1)
C(6)	17(1)	17(1)	13(1)	-1(1)	3(1)	1(1)
C(7)	15(1)	18(1)	16(1)	-1(1)	3(1)	1(1)
C(8)	14(1)	13(1)	16(1)	-1(1)	0(1)	0(1)
C(9)	19(1)	17(1)	15(1)	1(1)	0(1)	-1(1)
C(10)	16(1)	13(1)	19(1)	0(1)	2(1)	0(1)
C(11)	16(1)	11(1)	19(1)	-1(1)	0(1)	1(1)
C(12)	15(1)	14(1)	22(1)	0(1)	3(1)	1(1)
C(13)	21(1)	16(1)	16(1)	1(1)	4(1)	2(1)
C(14)	18(1)	13(1)	17(1)	-1(1)	-2(1)	3(1)
C(15)	15(1)	14(1)	20(1)	1(1)	2(1)	-1(1)
C(16)	18(1)	14(1)	16(1)	1(1)	4(1)	0(1)
Br(1)	21(1)	22(1)	15(1)	1(1)	-2(1)	0(1)
O(1)	18(1)	22(1)	23(1)	2(1)	-4(1)	3(1)
N(1)	15(1)	16(1)	17(1)	-1(1)	-2(1)	-2(1)
C(17)	19(1)	25(1)	20(1)	-3(1)	-3(1)	-4(1)

Table S5. Hydrogen coordinates (x 10^4) and isotropic displacement parameters ($Å^2$x 10^3).

	x	y	z	U(eq)
H(1)	12420(40)	13810(20)	11420(15)	35(8)
H(2)	9240(40)	14230(20)	10639(14)	29(7)
H(3)	6840(40)	12897(19)	10335(13)	23(7)
H(4)	7400(40)	11121(19)	10645(13)	19(7)
H(5)	8190(40)	9656(18)	11970(13)	17(6)

H(6)	10330(40)	9234(17)	12384(12)	17(6)
H(7)	12150(40)	8810(20)	11227(14)	26(7)
H(8)	11600(40)	9639(18)	9757(13)	15(6)
H(9)	9460(40)	9414(18)	8699(13)	20(6)
H(10)	4610(40)	8215(16)	10024(12)	10(6)
H(11)	6740(40)	8440(19)	11052(14)	21(6)
H(12)	14420	12742	12669	32
H(13)	15766	12827	11924	32
H(14)	16181	11875	12473	32

Table S6. Torsion angles [°].

C(6)-C(1)-C(2)-C(3)	0.3(3)
C(1)-C(2)-C(3)-C(4)	0.6(3)
C(2)-C(3)-C(4)-C(5)	-1.0(3)
C(3)-C(4)-C(5)-C(6)	0.6(3)
C(3)-C(4)-C(5)-C(8)	-178.9(2)
C(2)-C(1)-C(6)-C(5)	-0.8(3)
C(2)-C(1)-C(6)-N(1)	178.2(2)
C(4)-C(5)-C(6)-C(1)	0.4(3)
C(8)-C(5)-C(6)-C(1)	180.0(2)
C(4)-C(5)-C(6)-N(1)	-178.83(18)
C(8)-C(5)-C(6)-N(1)	0.8(2)
C(4)-C(5)-C(8)-C(7)	176.7(2)
C(6)-C(5)-C(8)-C(7)	-2.8(2)
C(4)-C(5)-C(8)-C(9)	-45.8(3)
C(6)-C(5)-C(8)-C(9)	134.6(2)
C(4)-C(5)-C(8)-C(10)	27.9(4)
C(6)-C(5)-C(8)-C(10)	-151.64(19)
O(1)-C(7)-C(8)-C(5)	-179.6(2)
N(1)-C(7)-C(8)-C(5)	3.9(2)
O(1)-C(7)-C(8)-C(9)	37.3(3)
N(1)-C(7)-C(8)-C(9)	-139.26(18)
O(1)-C(7)-C(8)-C(10)	-28.5(3)
N(1)-C(7)-C(8)-C(10)	154.97(18)
C(5)-C(8)-C(9)-C(10)	113.9(2)
C(7)-C(8)-C(9)-C(10)	-111.63(19)

C(8)-C(9)-C(10)-C(11)	-109.7(2)
C(5)-C(8)-C(10)-C(9)	-113.8(2)
C(7)-C(8)-C(10)-C(9)	101.4(2)
C(5)-C(8)-C(10)-C(11)	-3.1(3)
C(7)-C(8)-C(10)-C(11)	-147.9(2)
C(9)-C(8)-C(10)-C(11)	110.7(2)
C(9)-C(10)-C(11)-C(12)	148.82(19)
C(8)-C(10)-C(11)-C(12)	77.0(3)
C(9)-C(10)-C(11)-C(16)	-32.1(3)
C(8)-C(10)-C(11)-C(16)	-103.8(2)
C(16)-C(11)-C(12)-C(13)	-0.3(3)
C(10)-C(11)-C(12)-C(13)	178.88(19)
C(11)-C(12)-C(13)-C(14)	0.3(3)
C(12)-C(13)-C(14)-C(15)	-0.8(3)
C(12)-C(13)-C(14)-Br(1)	177.85(16)
C(13)-C(14)-C(15)-C(16)	1.2(3)
Br(1)-C(14)-C(15)-C(16)	-177.47(16)
C(14)-C(15)-C(16)-C(11)	-1.1(3)
C(12)-C(11)-C(16)-C(15)	0.7(3)
C(10)-C(11)-C(16)-C(15)	-178.5(2)
O(1)-C(7)-N(1)-C(6)	179.8(2)
C(8)-C(7)-N(1)-C(6)	-3.5(2)
O(1)-C(7)-N(1)-C(17)	-5.1(3)
C(8)-C(7)-N(1)-C(17)	171.52(18)
C(1)-C(6)-N(1)-C(7)	-177.4(2)
C(5)-C(6)-N(1)-C(7)	1.8(2)
C(1)-C(6)-N(1)-C(17)	7.5(3)
C(5)-C(6)-N(1)-C(17)	-173.30(18)

Symmetry transformations used to generate equivalent atoms:

Table S7. Hydrogen bonds [Å and °].

D-H...A	*d*(D-H)	*d*(H...A)	*d*(D...A)	<(DHA)
C(17)-H(14)...Br(1)#1	0.96	2.94	3.825(2)	153.0

Symmetry transformations used to generate equivalent atoms:

#1 -x+5/2,-y+2,z+1/2

6. NMR Spectral Data

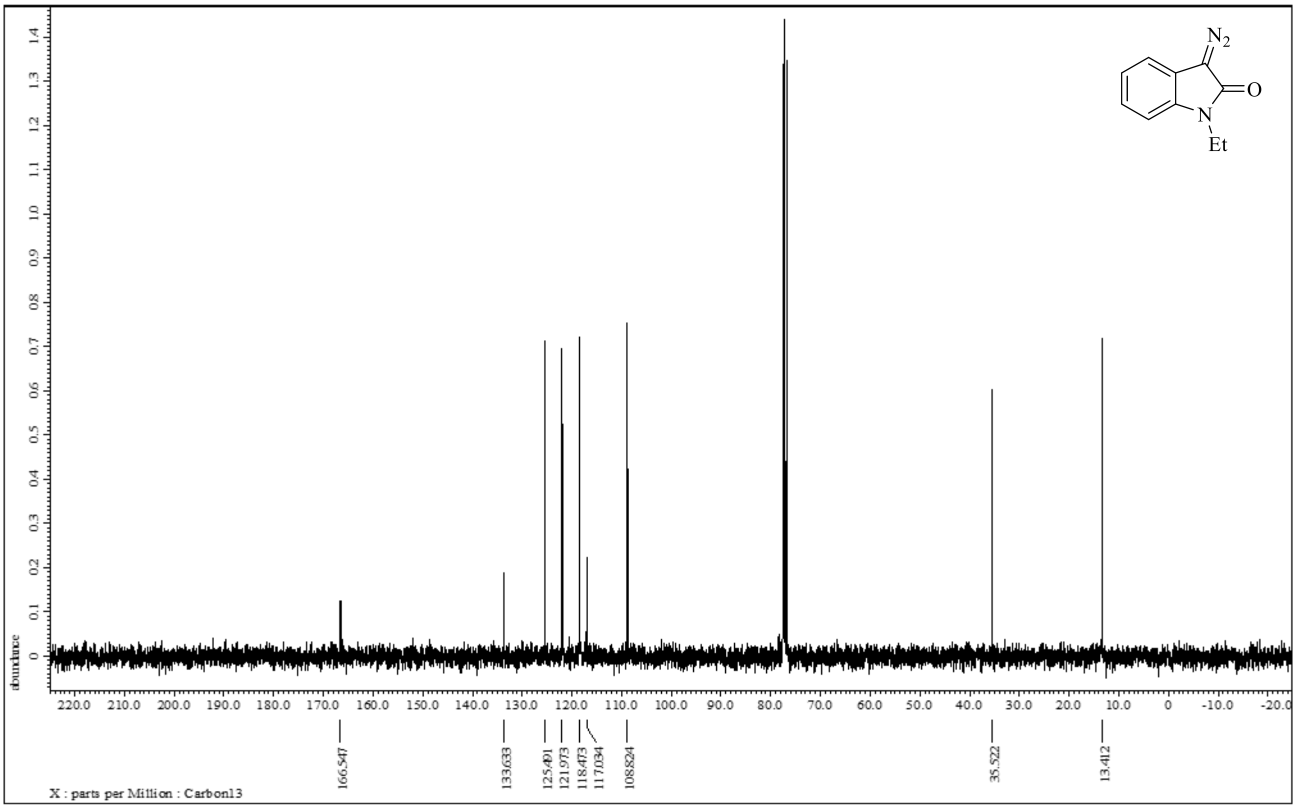

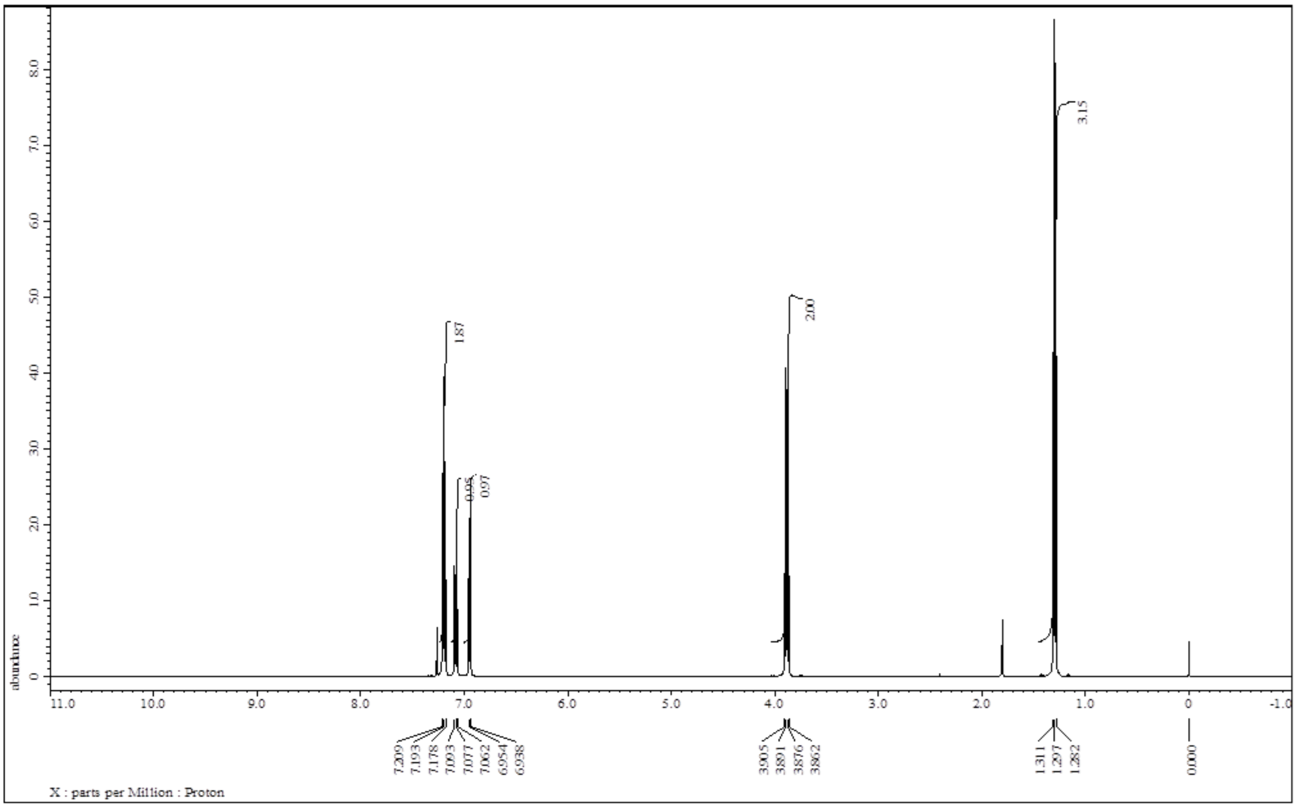

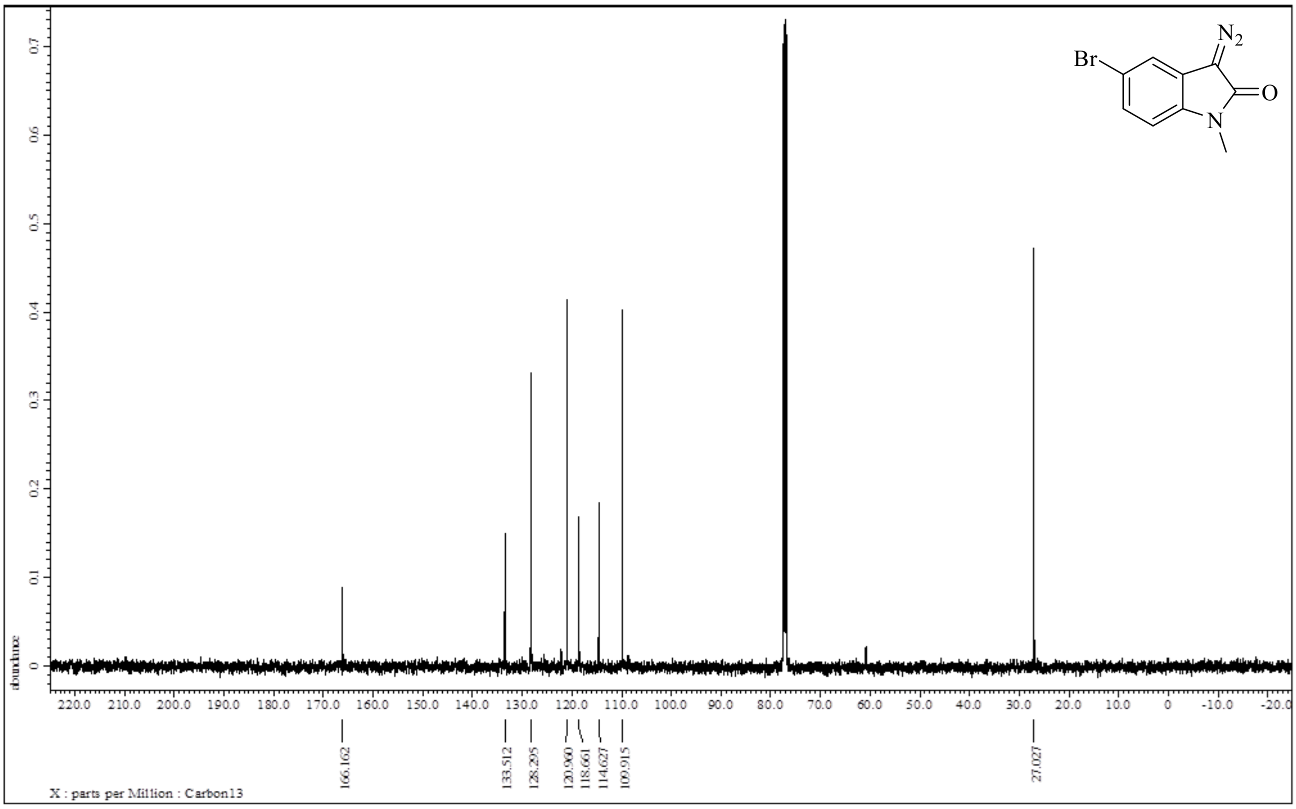
N2
Br
O
N
abundance
0
0.1
0.2
0.3
0.4
0.5
0.6
0.7
220.0 210.0 200.0 190.0 180.0 170.0 160.0 150.0 140.0 130.0 120.0 110.0 100.0 90.0 80.0 70.0 60.0 50.0 40.0 30.0 20.0 10.0 0 -10.0 -20.0
166.162
133.512
128.295
120.960
118.661
114.627
109.915
27.027
X : parts per Million : Carbon13

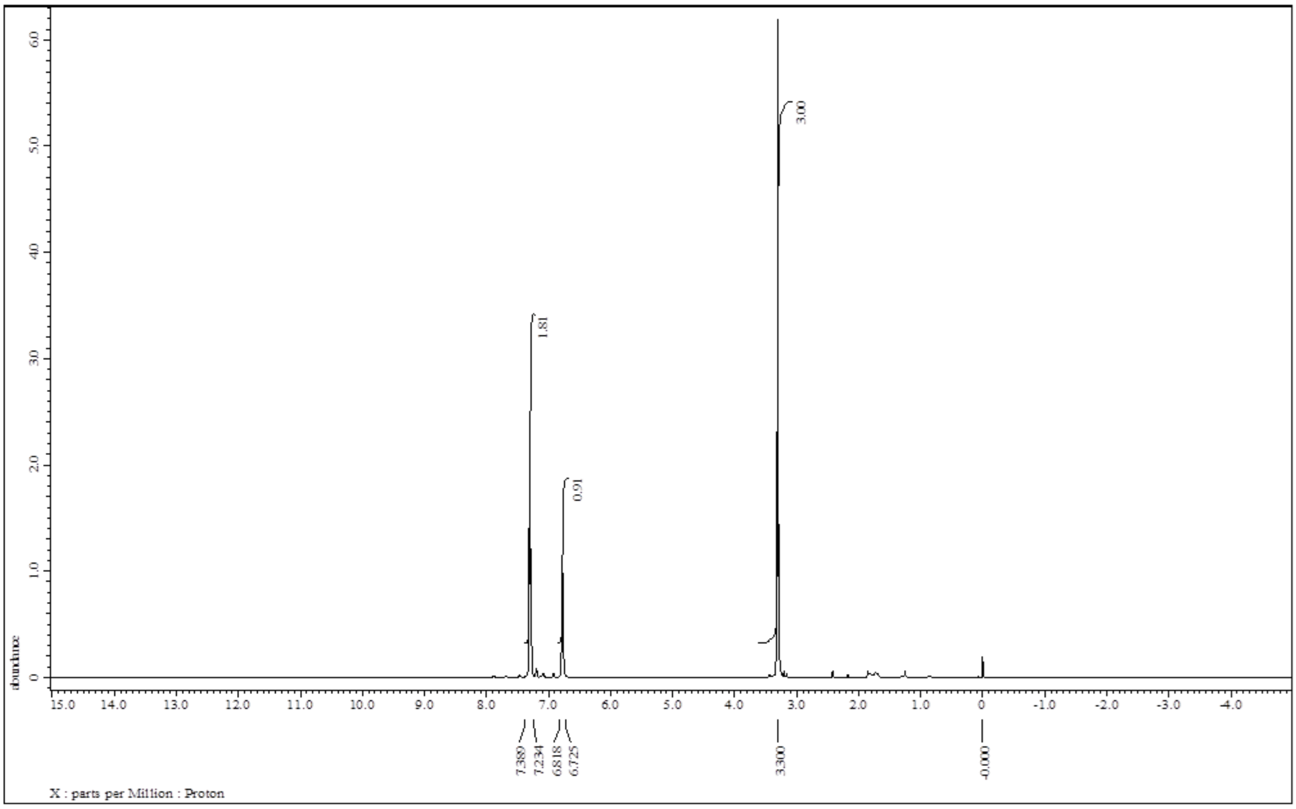
abundance
0
1.0
2.0
3.0
4.0
5.0
6.0
1.81
0.91
3.00
15.0 14.0 13.0 12.0 11.0 10.0 9.0 8.0 7.0 6.0 5.0 4.0 3.0 2.0 1.0 0 -1.0 -2.0 -3.0 -4.0
7.389
7.234
6.818
6.725
3.300
-0.000
X : parts per Million : Proton

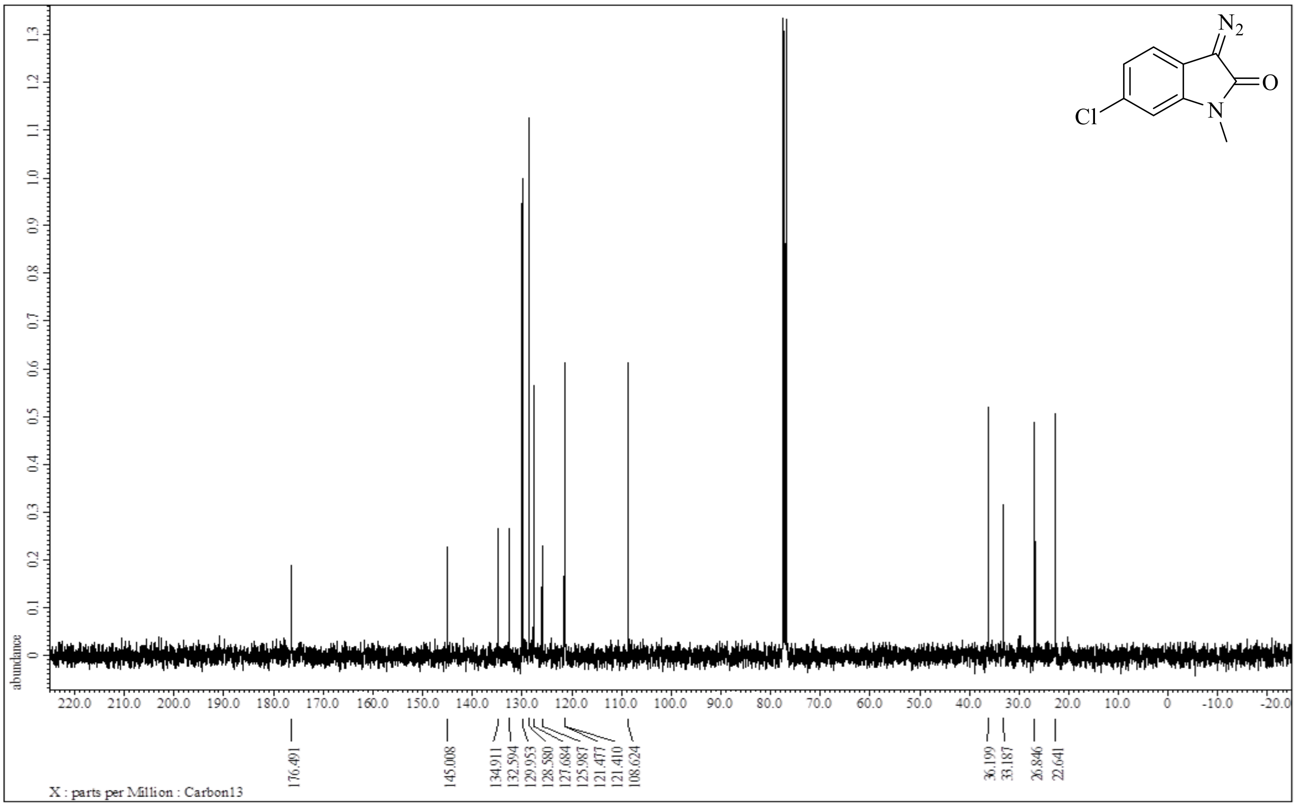

N2
O
N
Cl
abundance
176.491
145.008
134.911
132.594
129.953
128.580
127.684
125.987
121.477
121.410
108.624
36.199
33.187
26.846
22.641
X : parts per Million : Carbon13

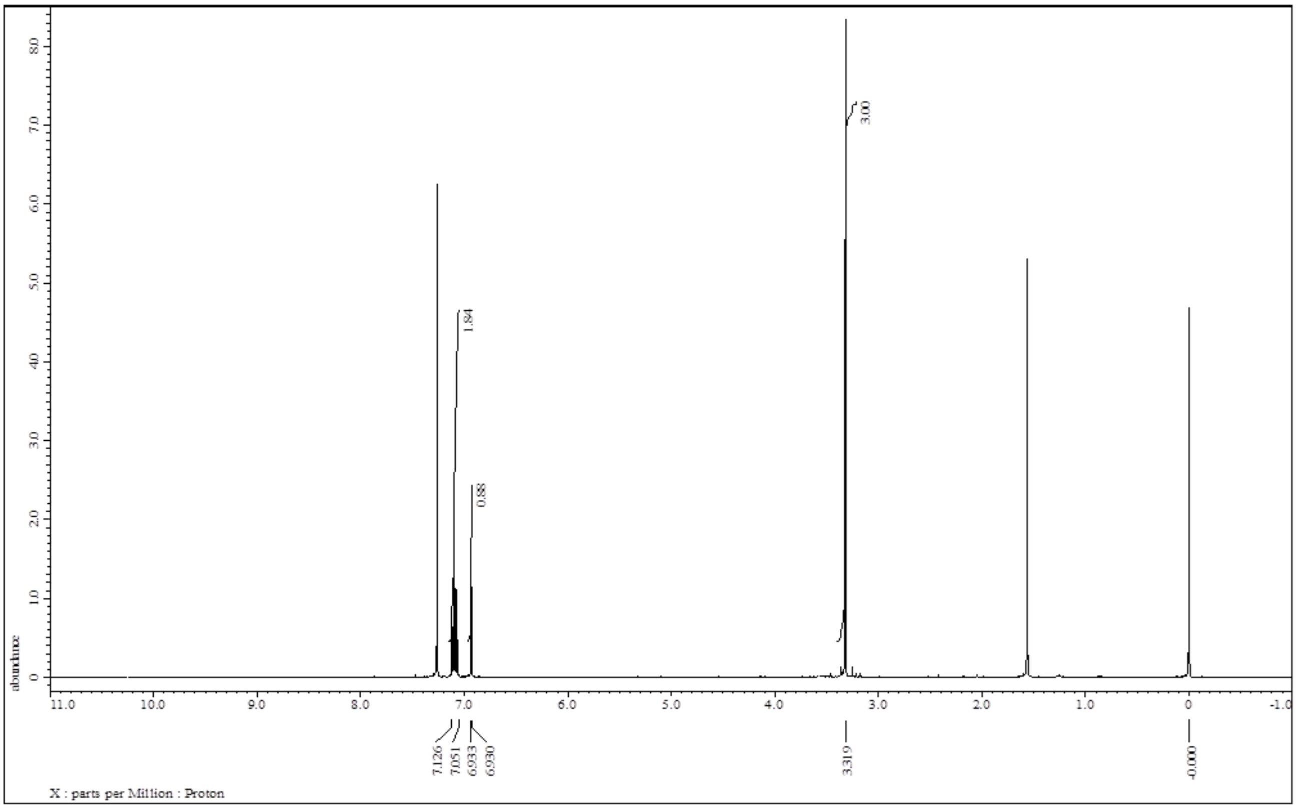

abundance
3.00
1.84
0.88
7.126
7.051
6.933
6.930
3.319
-0.000
X : parts per Million : Proton

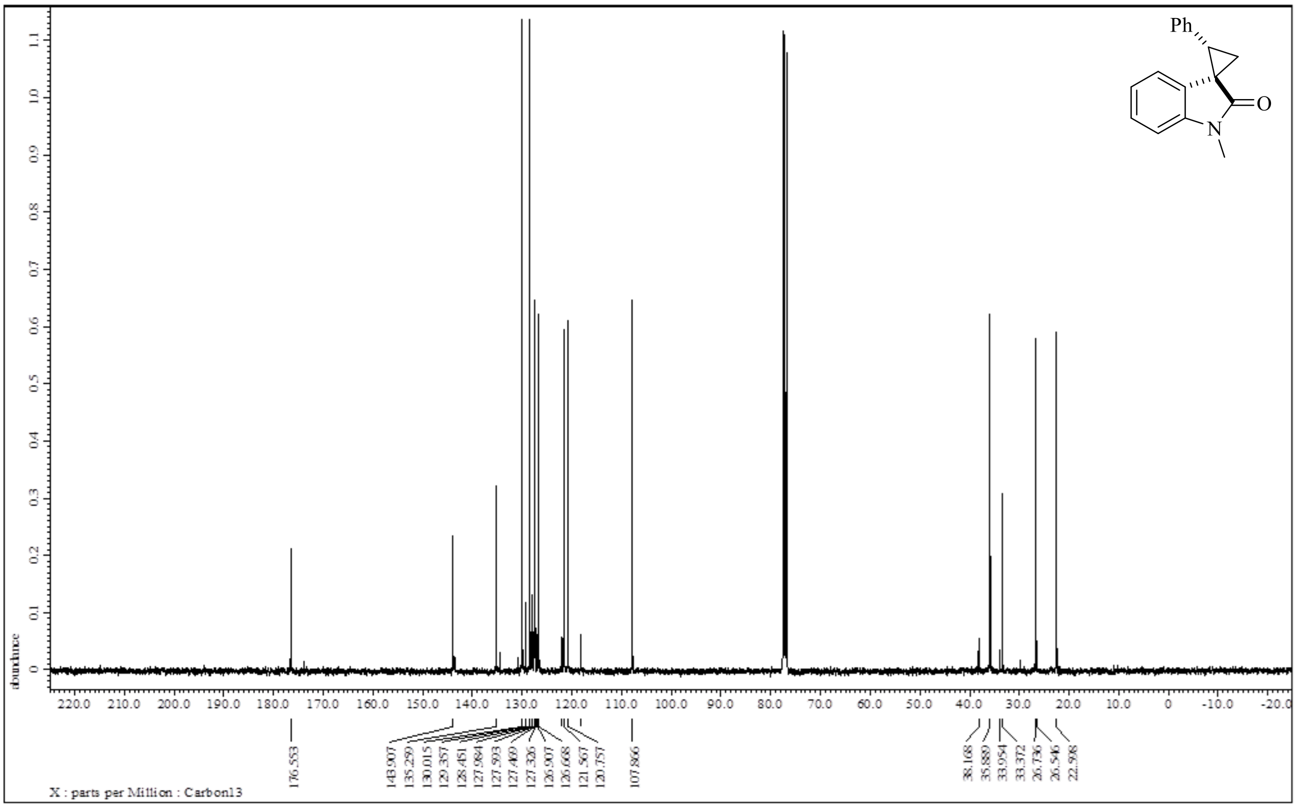
Ph
N
O
abundance
176.553
143.907
135.299
130.015
129.357
128.451
127.984
127.593
127.489
127.326
126.907
126.668
121.567
120.757
107.866
38.168
35.889
33.954
33.372
26.736
26.546
22.998
X : parts per Million : Carbon13

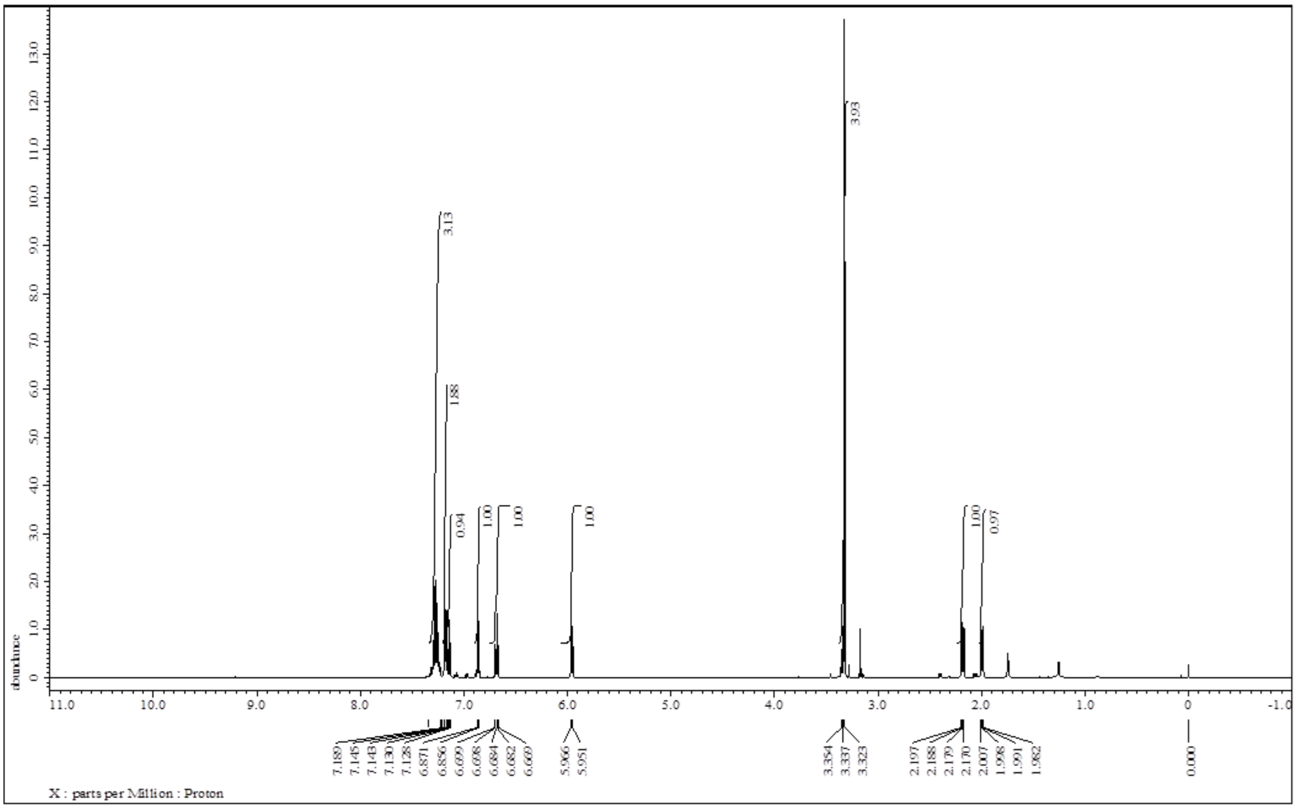
abundance
3.13
1.88
0.94
1.00
1.00
1.00
3.93
1.00
0.97
7.189
7.145
7.143
7.130
7.128
6.871
6.856
6.699
6.698
6.684
6.682
6.669
5.966
5.951
3.354
3.337
3.323
2.197
2.188
2.179
2.170
2.007
1.998
1.991
1.982
0.000
X : parts per Million : Proton

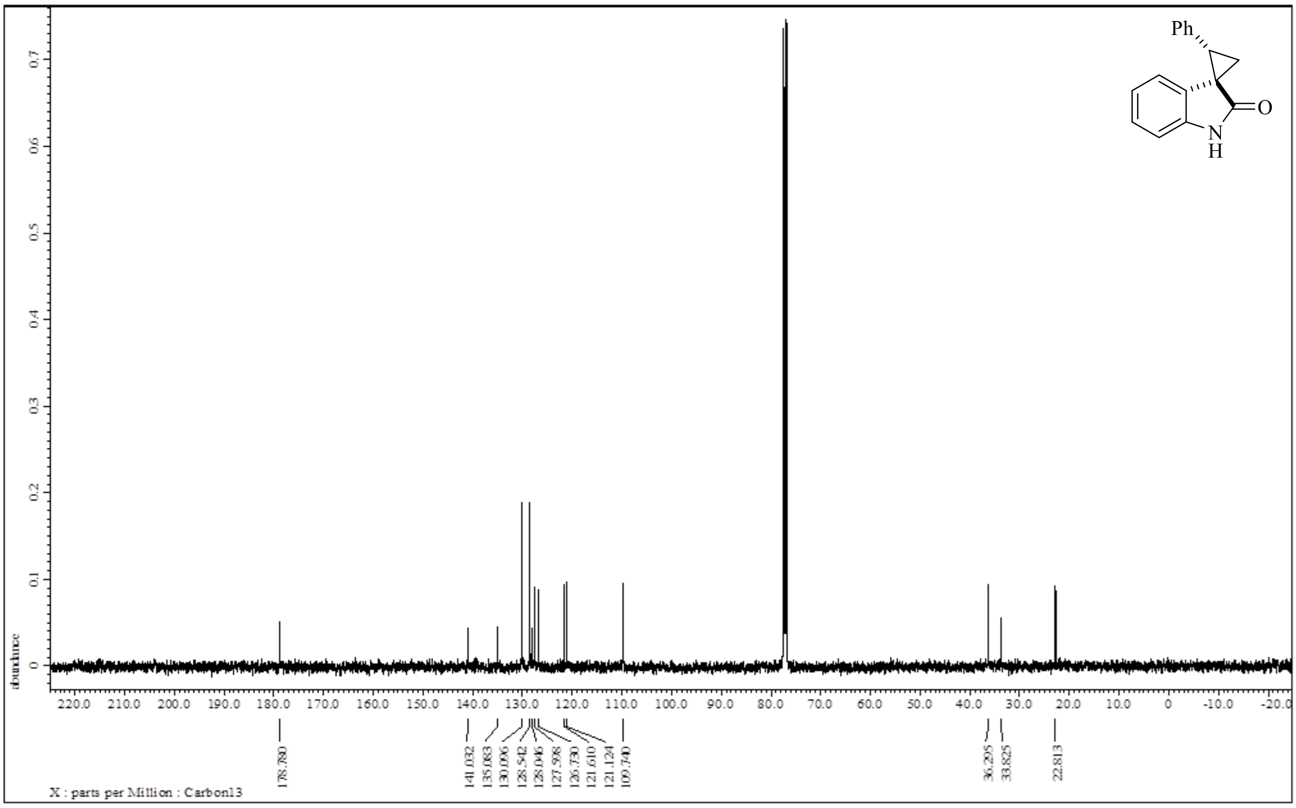
Ph
N
H
O
abundance
178.780
141.032
135.083
130.096
128.542
128.046
127.598
126.730
121.610
121.124
109.740
36.295
33.825
22.813
X : parts per Million : Carbon13

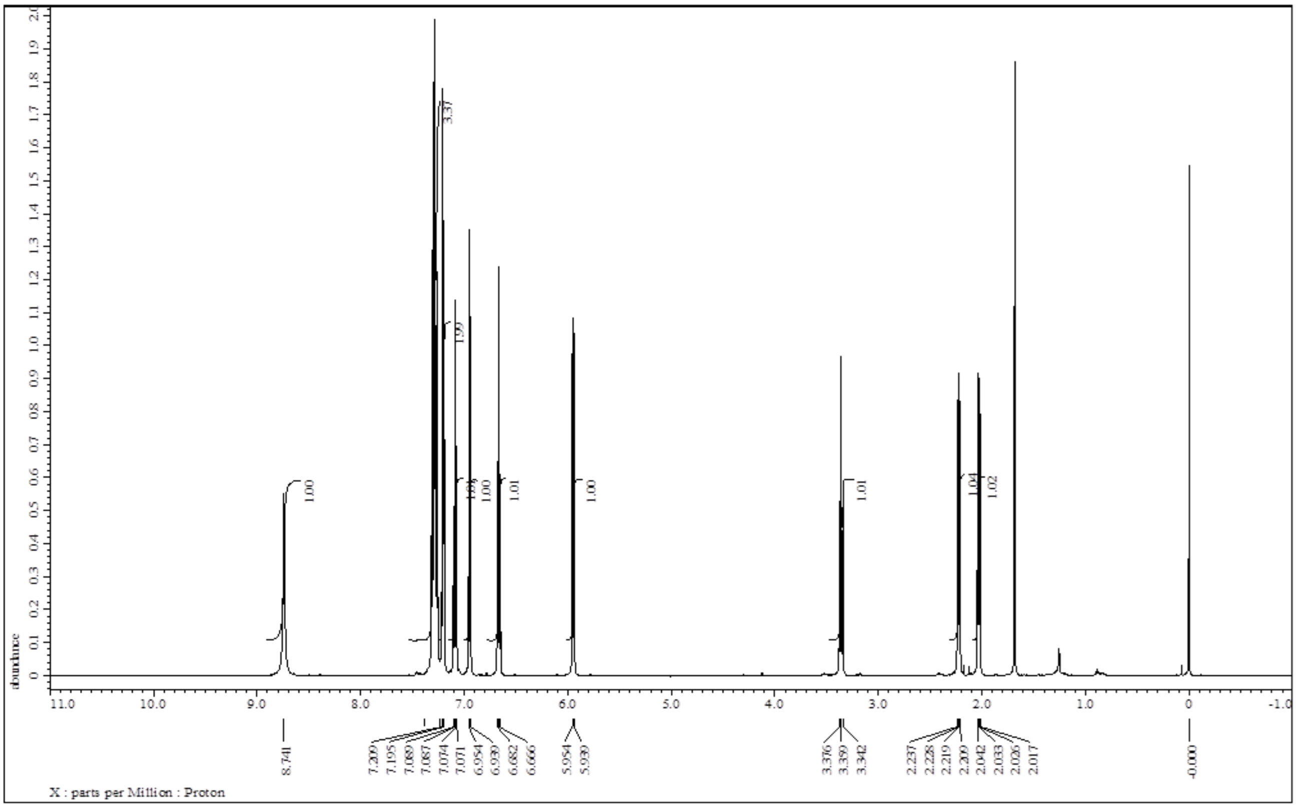
abundance
3.37
1.99
1.00
1.01
1.00
1.01
1.00
1.01
1.04
1.02
8.741
7.209
7.195
7.089
7.087
7.074
7.071
6.954
6.939
6.682
6.666
5.954
5.939
3.376
3.359
3.342
2.237
2.228
2.219
2.209
2.042
2.033
2.026
2.017
-0.000
X : parts per Million : Proton

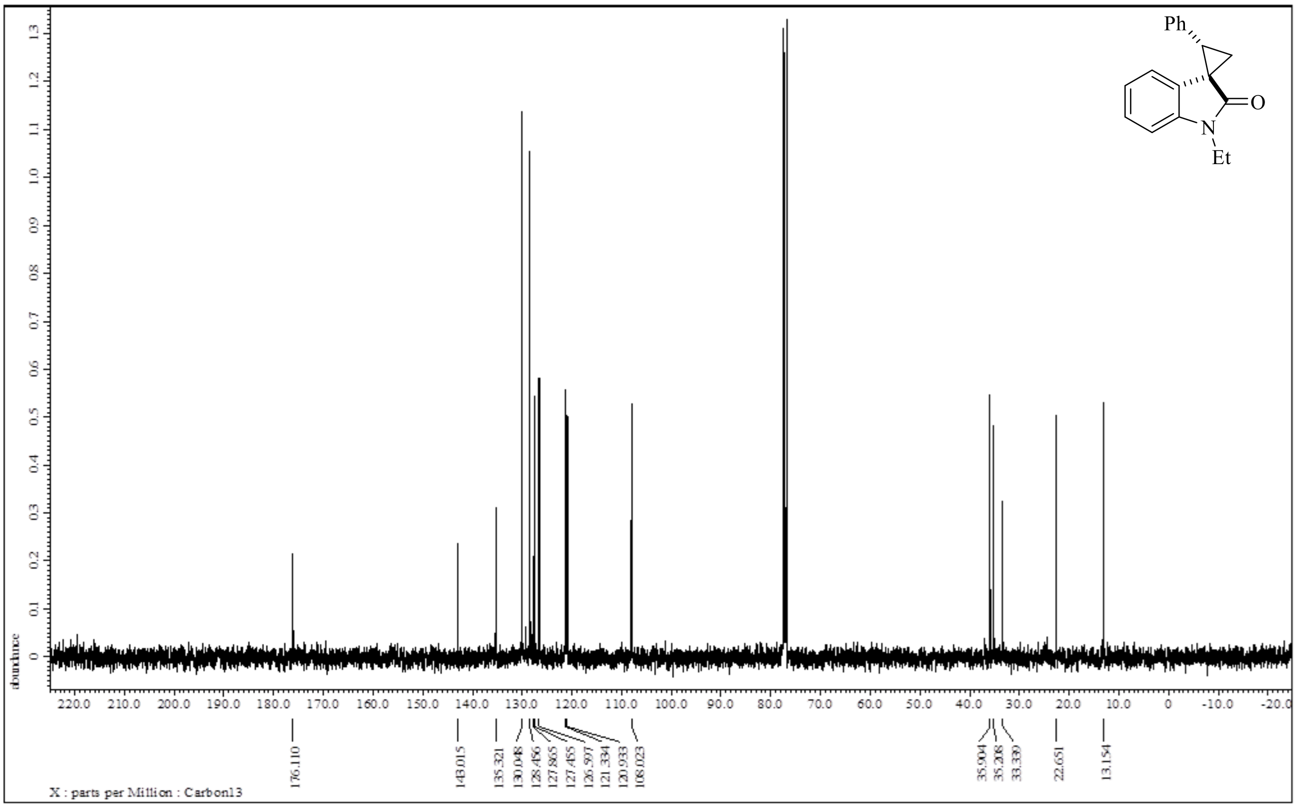
Ph
O
N
Et
abundance
176.110
143.015
135.321
130.048
128.456
127.865
127.455
126.597
121.334
120.933
108.023
35.904
35.208
33.339
22.651
13.154
X : parts per Million : Carbon13

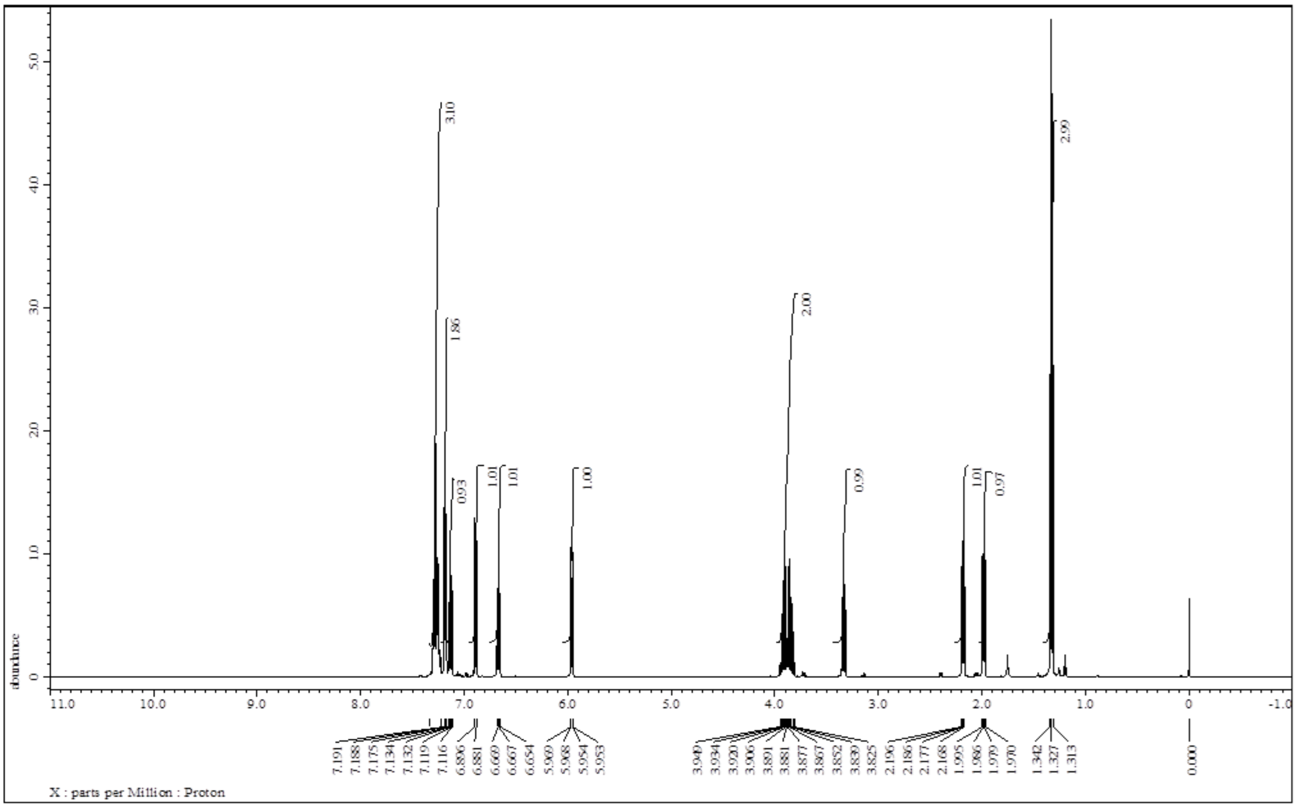
abundance
3.10
1.86
0.93
1.01
1.01
1.00
2.00
0.99
1.01
0.97
2.99
7.191
7.188
7.175
7.134
7.132
7.119
7.116
6.896
6.881
6.669
6.667
6.654
5.969
5.968
5.954
5.953
3.940
3.934
3.920
3.906
3.891
3.881
3.877
3.867
3.852
3.839
3.825
2.196
2.186
2.177
2.168
1.995
1.986
1.979
1.970
1.342
1.327
1.313
0.000
X : parts per Million : Proton

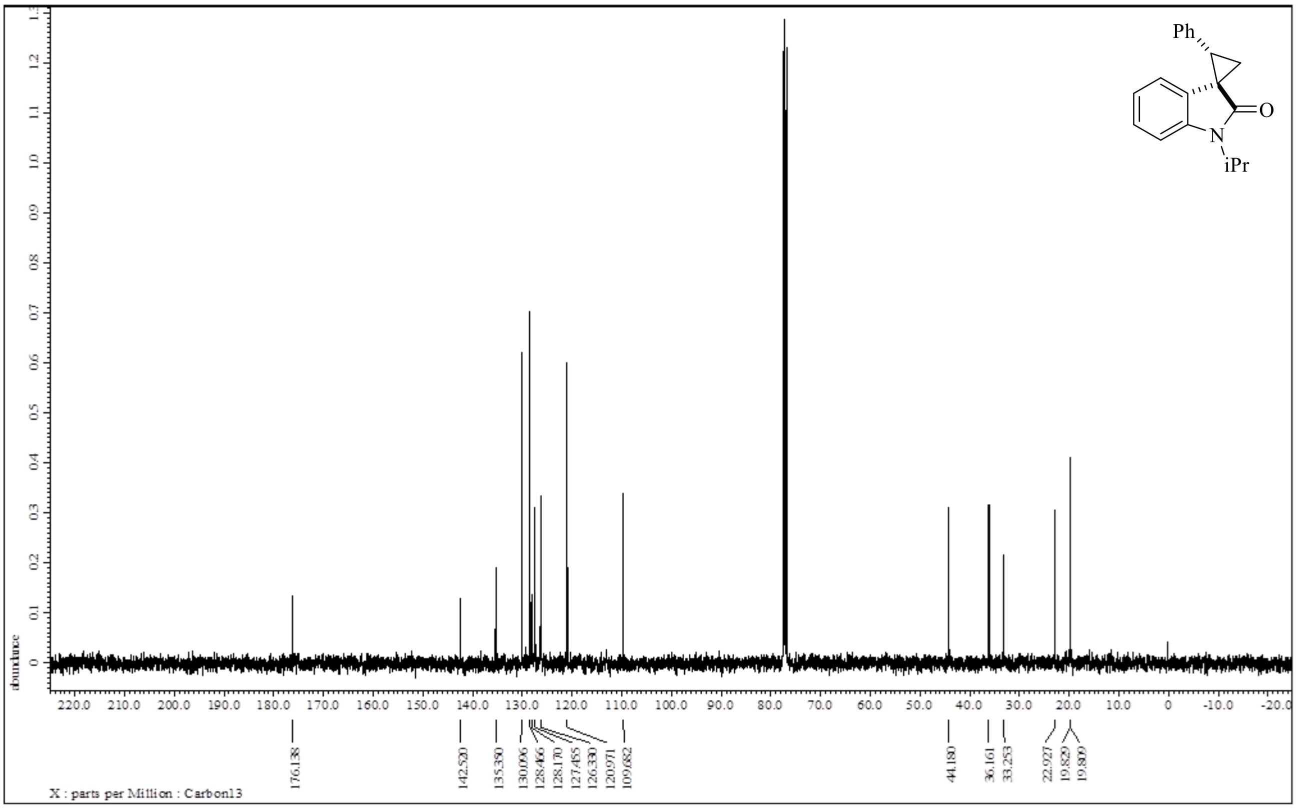
Ph
N
O
iPr
X : parts per Million : Carbon13
abundance

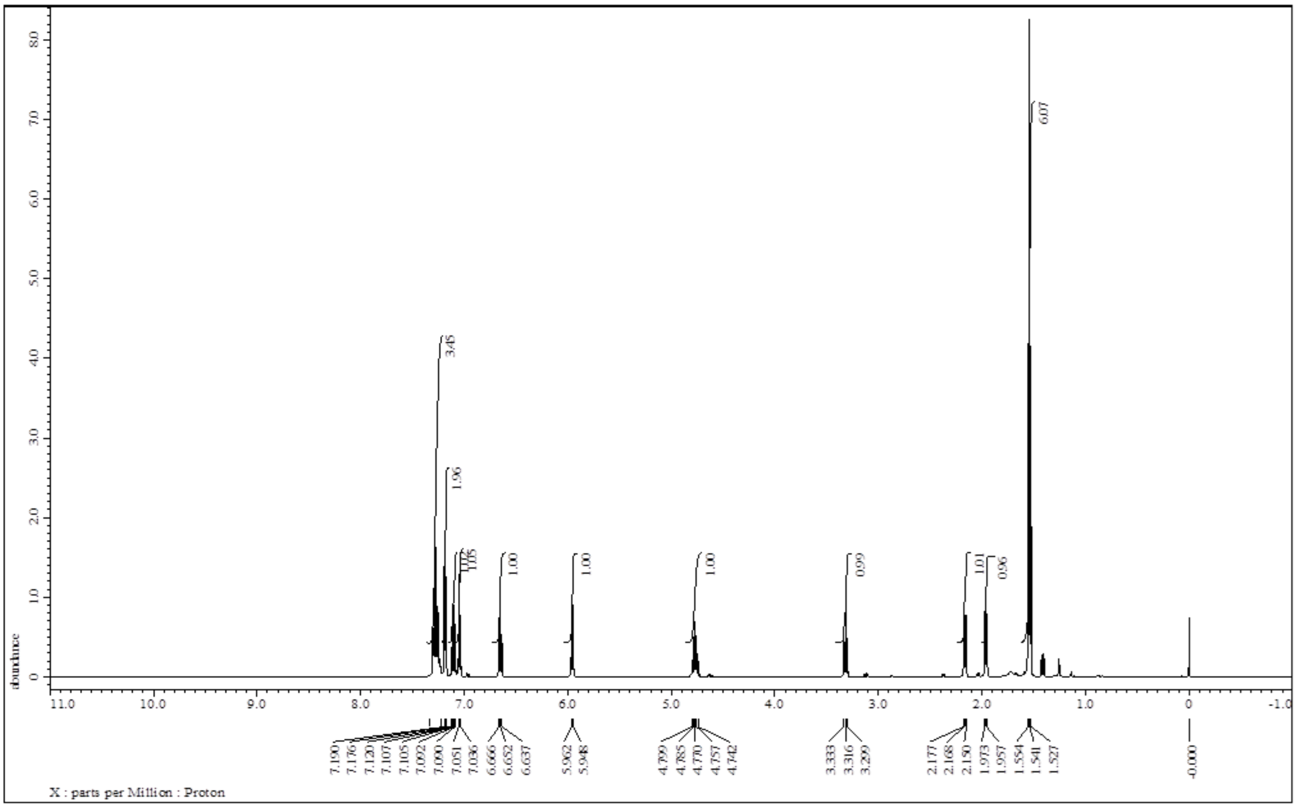
X : parts per Million : Proton
abundance

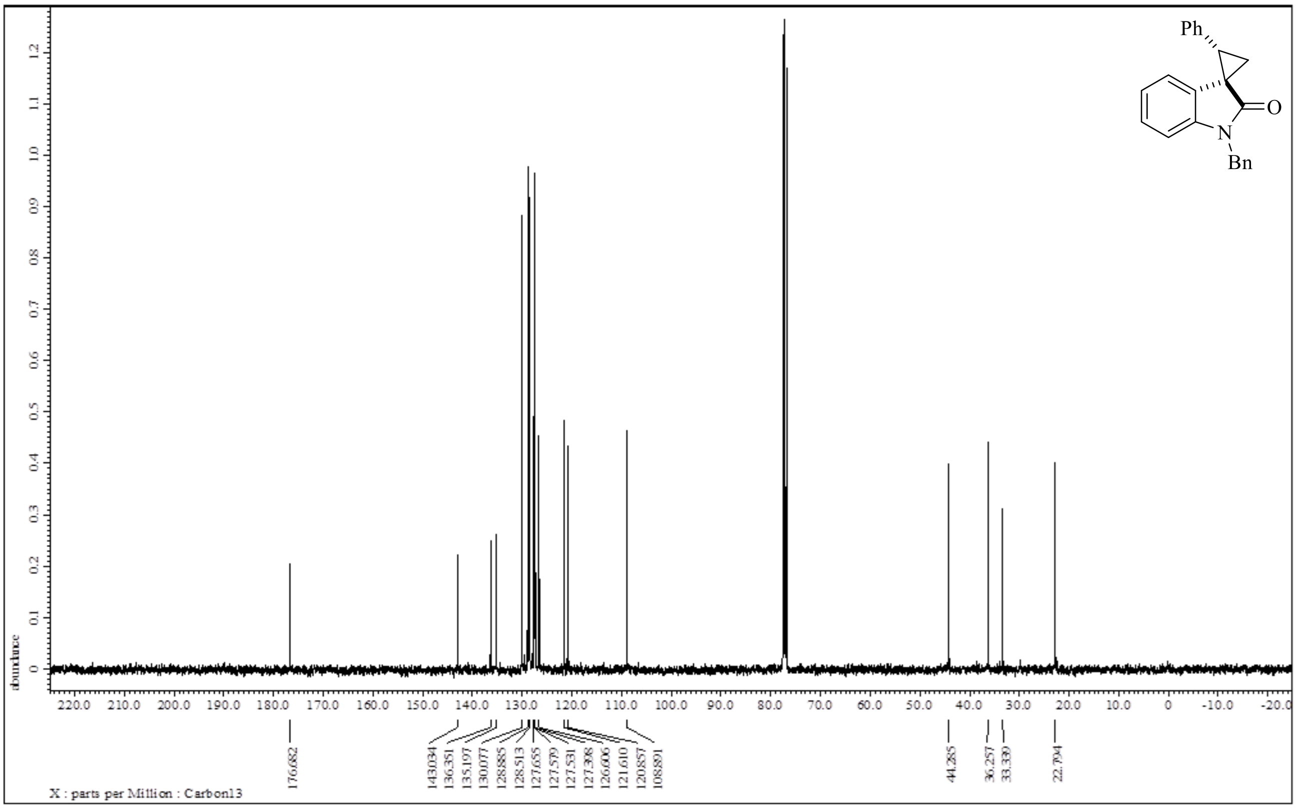
Ph
O
N
Bn
abundance
X : parts per Million : Carbon13
176.682
143.034
136.351
135.197
130.077
128.885
128.513
127.655
127.579
127.531
127.398
126.606
121.610
120.857
108.891
44.285
36.257
33.339
22.794

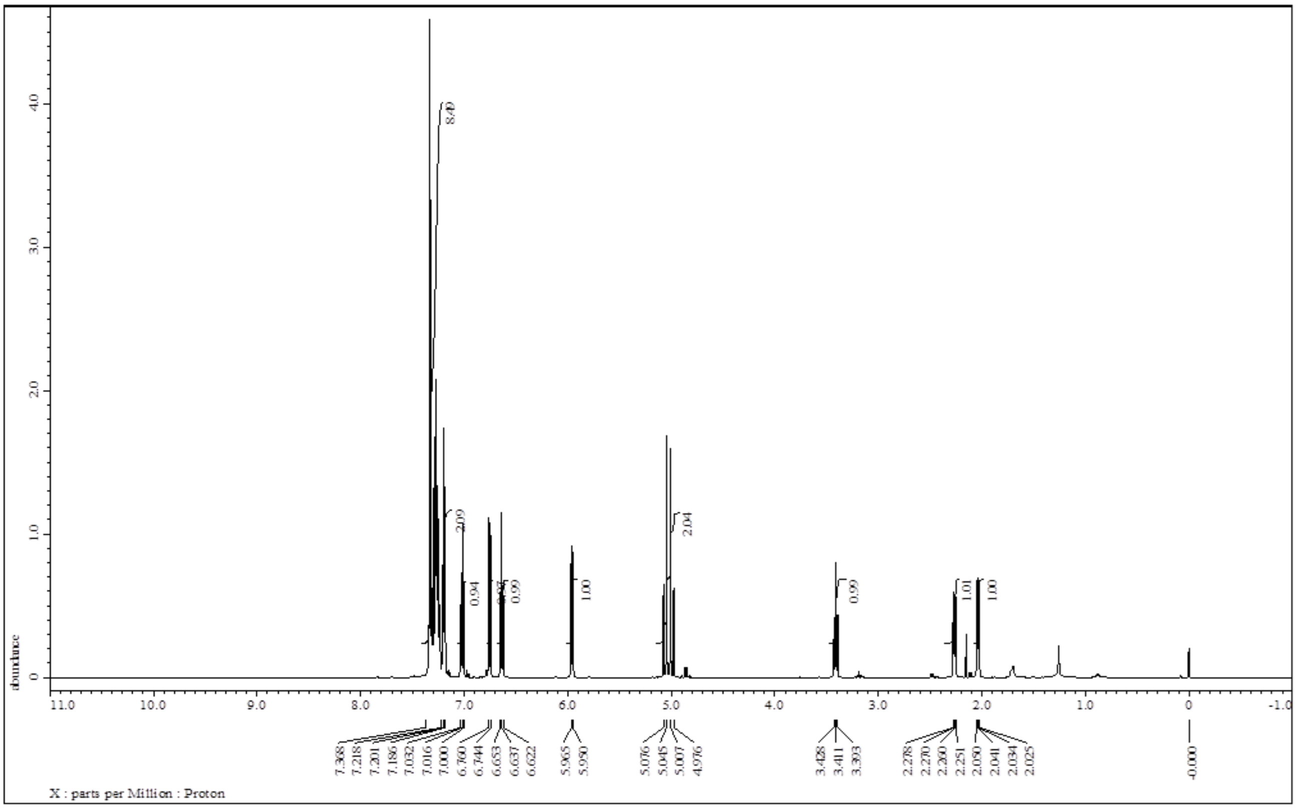
abundance
X : parts per Million : Proton
8.40
2.09
0.94
0.99
1.00
2.04
0.99
1.01
1.00
7.368
7.218
7.201
7.186
7.032
7.016
7.000
6.760
6.744
6.653
6.637
6.622
5.965
5.950
5.076
5.045
5.007
4.976
3.428
3.411
3.393
2.278
2.270
2.260
2.251
2.050
2.041
2.034
2.025
-0.000

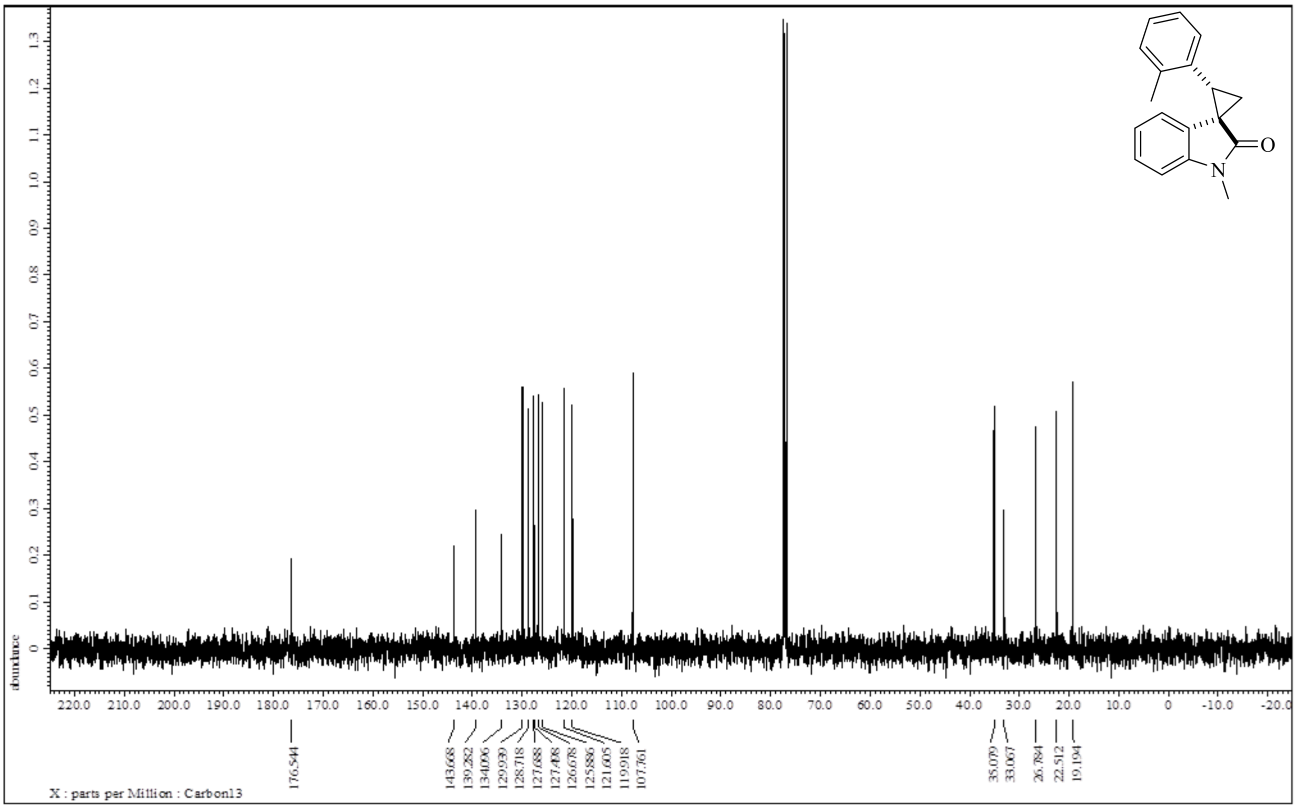
abundance
X : parts per Million : Carbon13
176.544
143.668
139.282
134.066
129.999
128.718
127.688
127.408
126.678
125.886
121.605
119.918
107.761
35.079
33.067
26.784
22.512
19.194
N
O

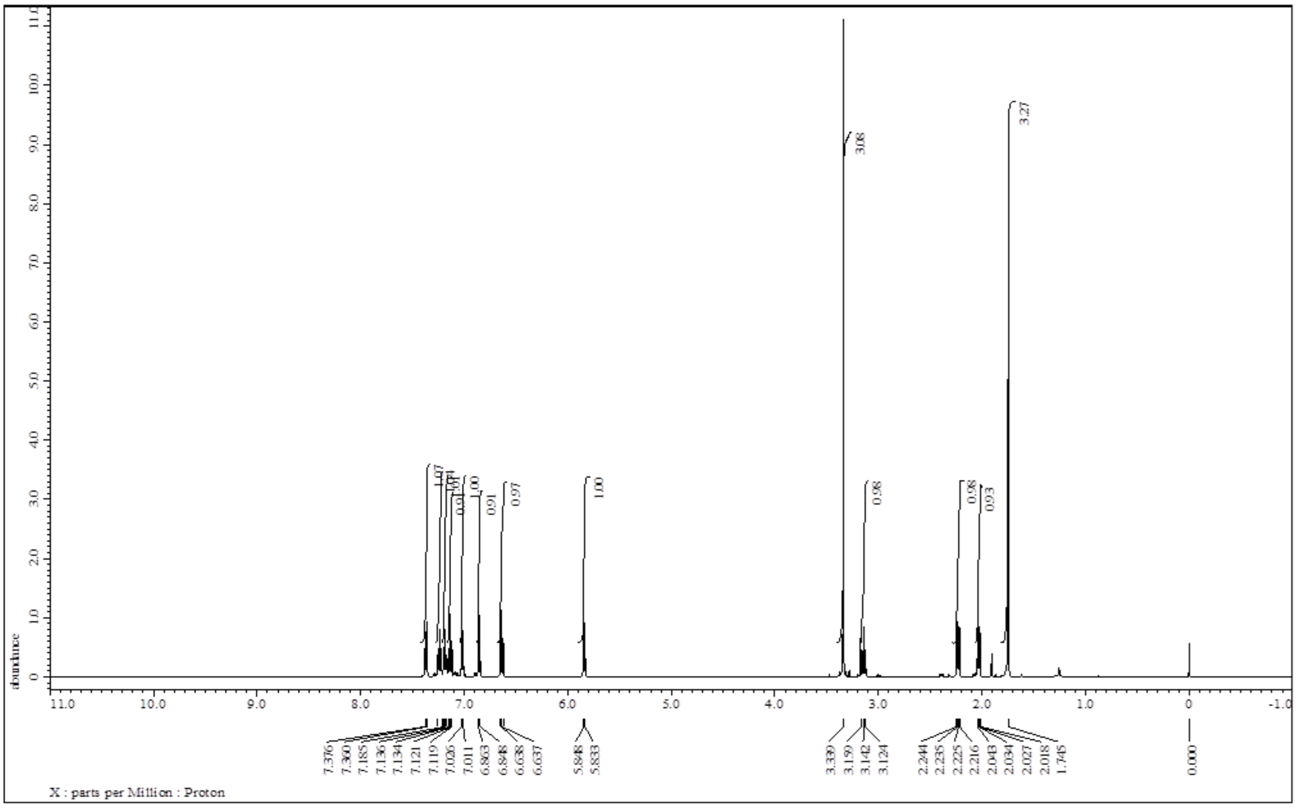
abundance
X : parts per Million : Proton
3.08
3.27
0.98
0.98
0.93
1.00
0.97
7.376
7.360
7.185
7.136
7.134
7.121
7.119
7.026
7.011
6.863
6.848
6.638
6.637
5.848
5.833
3.339
3.159
3.142
3.124
2.244
2.235
2.225
2.216
2.043
2.034
2.027
2.018
1.745
0.000

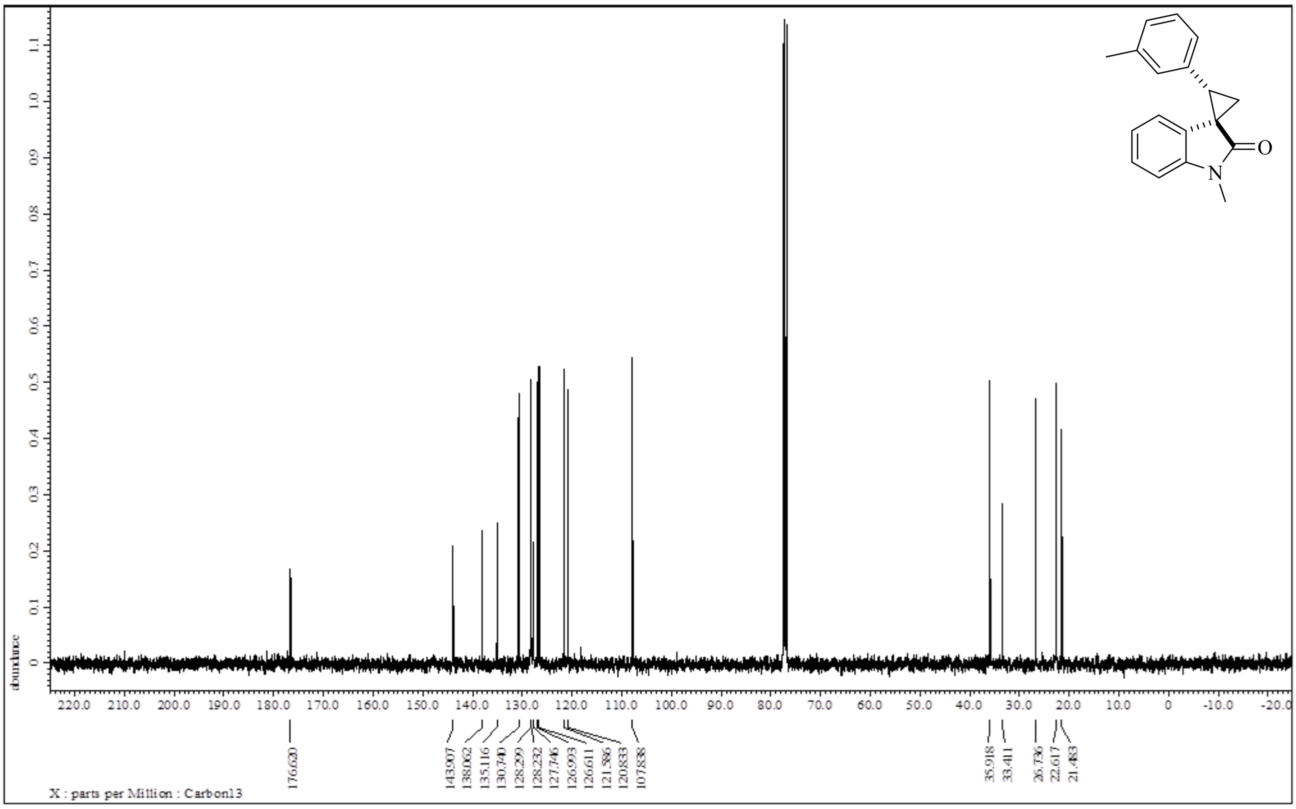
N
O
abundance
X : parts per Million : Carbon13
176.620
143.907
138.062
135.116
130.740
128.299
128.232
127.746
126.993
126.611
121.586
120.833
107.838
35.918
33.411
26.736
22.617
21.483

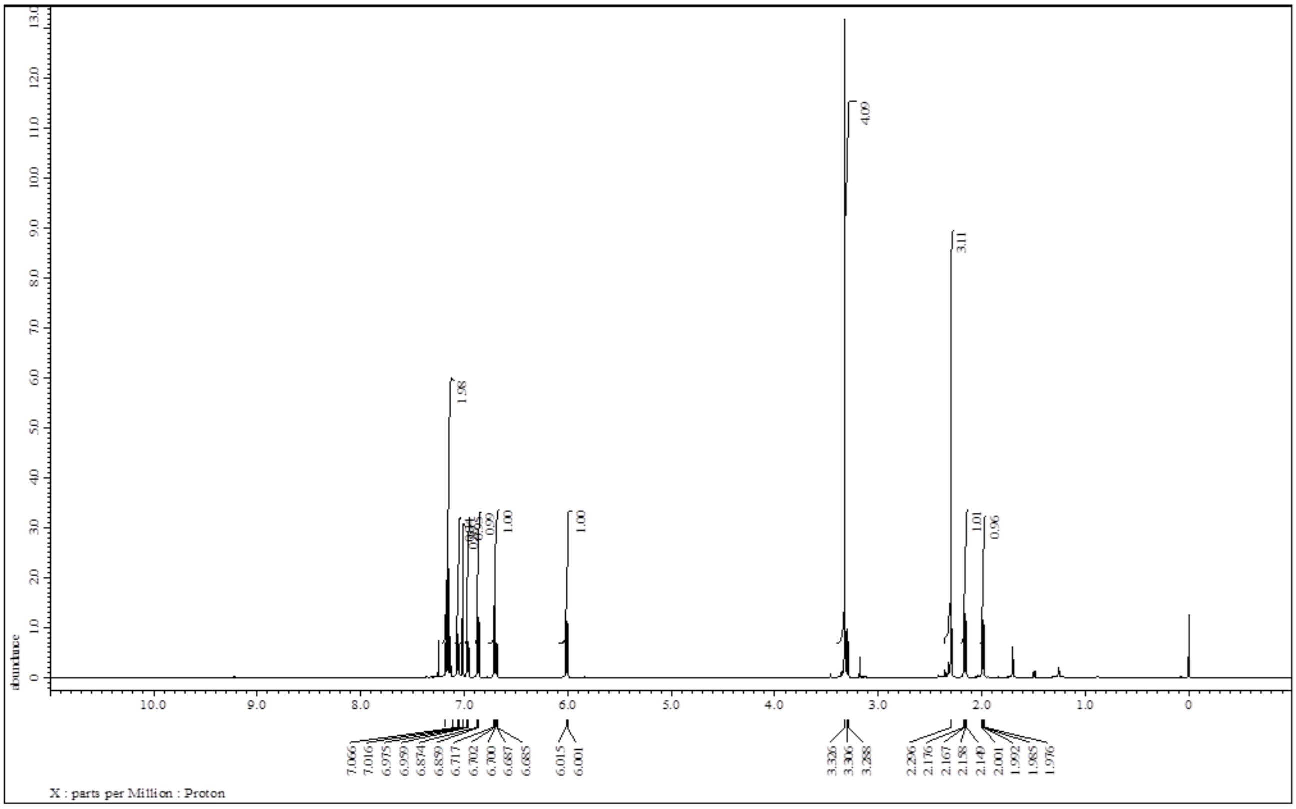
abundance
X : parts per Million : Proton
4.09
3.11
1.98
1.00
1.00
1.01
0.96
7.066
7.016
6.975
6.959
6.874
6.859
6.717
6.702
6.700
6.687
6.685
6.015
6.001
3.326
3.306
3.288
2.296
2.176
2.167
2.158
2.149
2.001
1.992
1.985
1.976

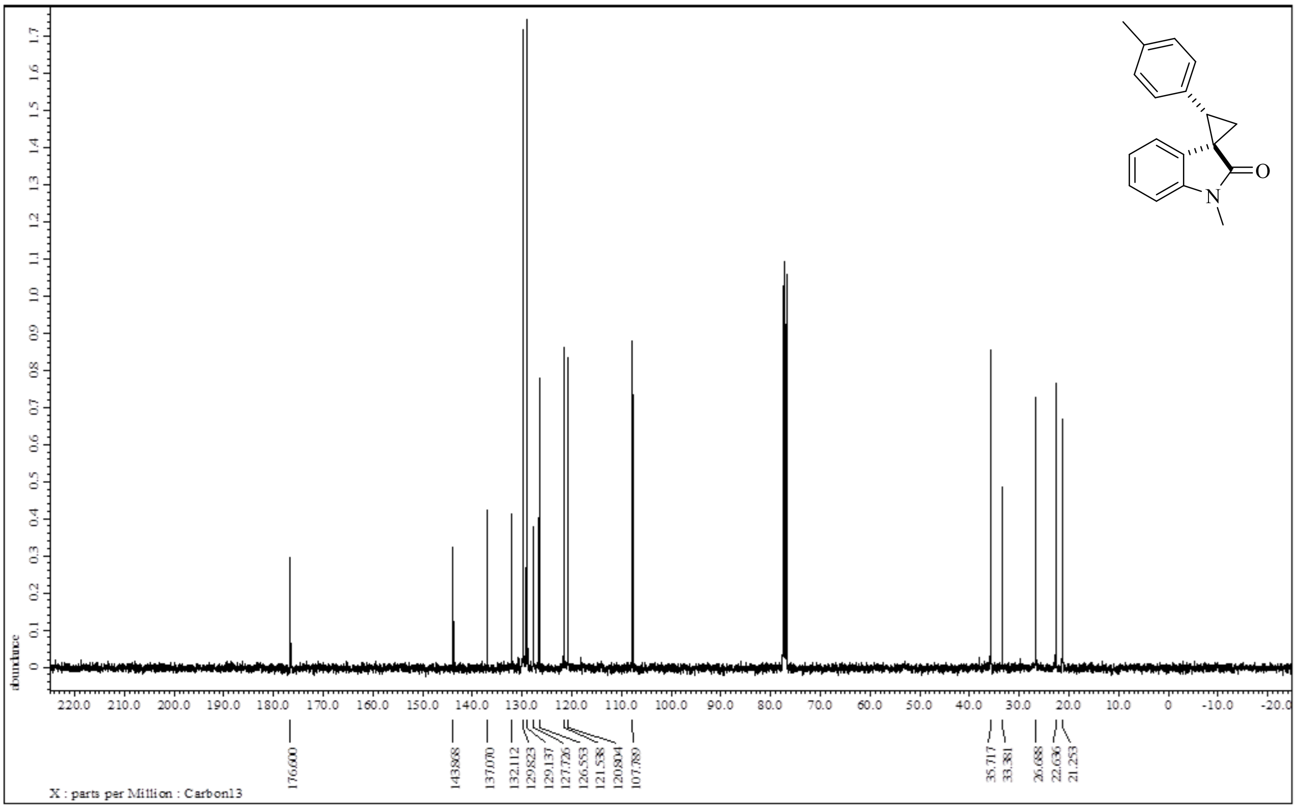
N
O
abundance
176.600
143.868
137.070
132.112
129.823
129.137
127.726
126.553
121.538
120.804
107.789
35.717
33.381
26.688
22.636
21.253
X : parts per Million : Carbon13

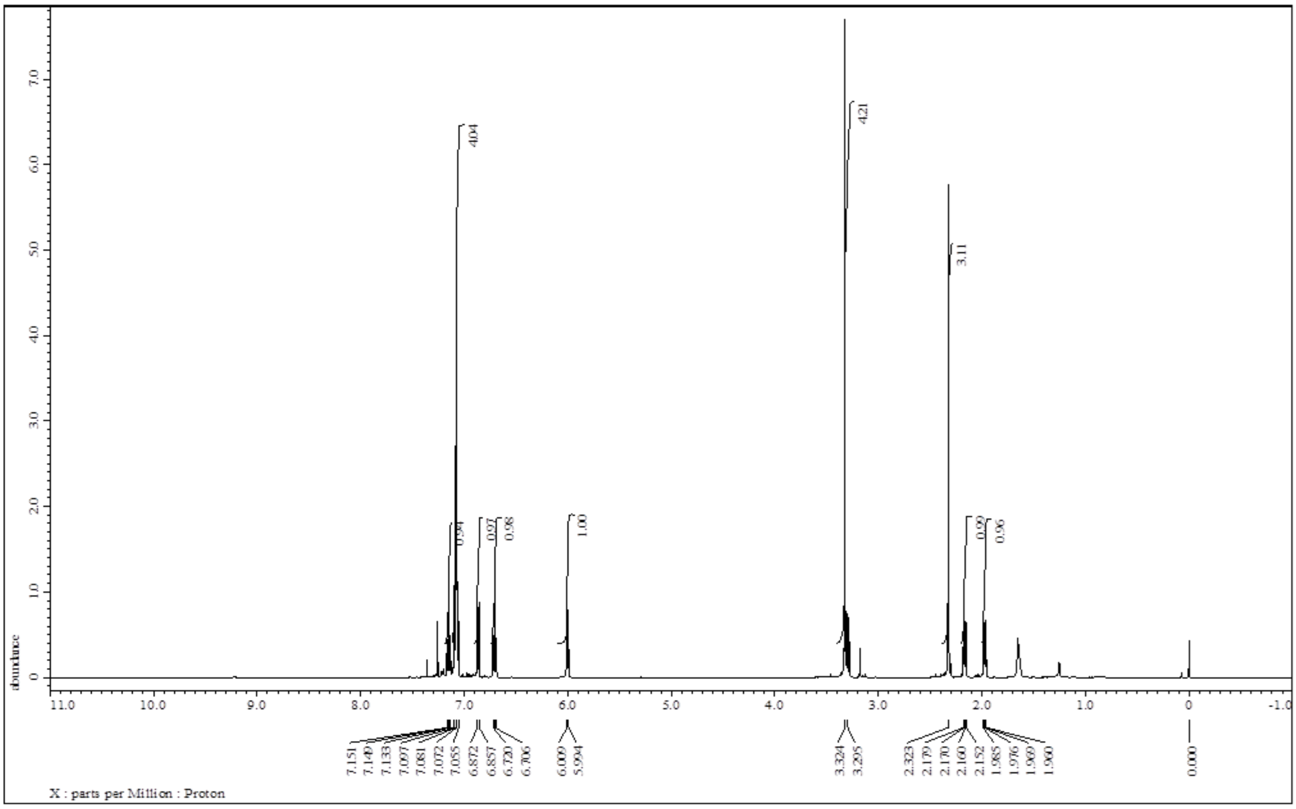
abundance
4.04
0.94
0.97
0.98
1.00
4.21
3.11
0.99
0.96
7.151
7.149
7.133
7.097
7.081
7.072
7.055
6.872
6.857
6.720
6.706
6.009
5.994
3.324
3.295
2.323
2.179
2.170
2.160
2.152
1.985
1.976
1.969
1.960
0.000
X : parts per Million : Proton

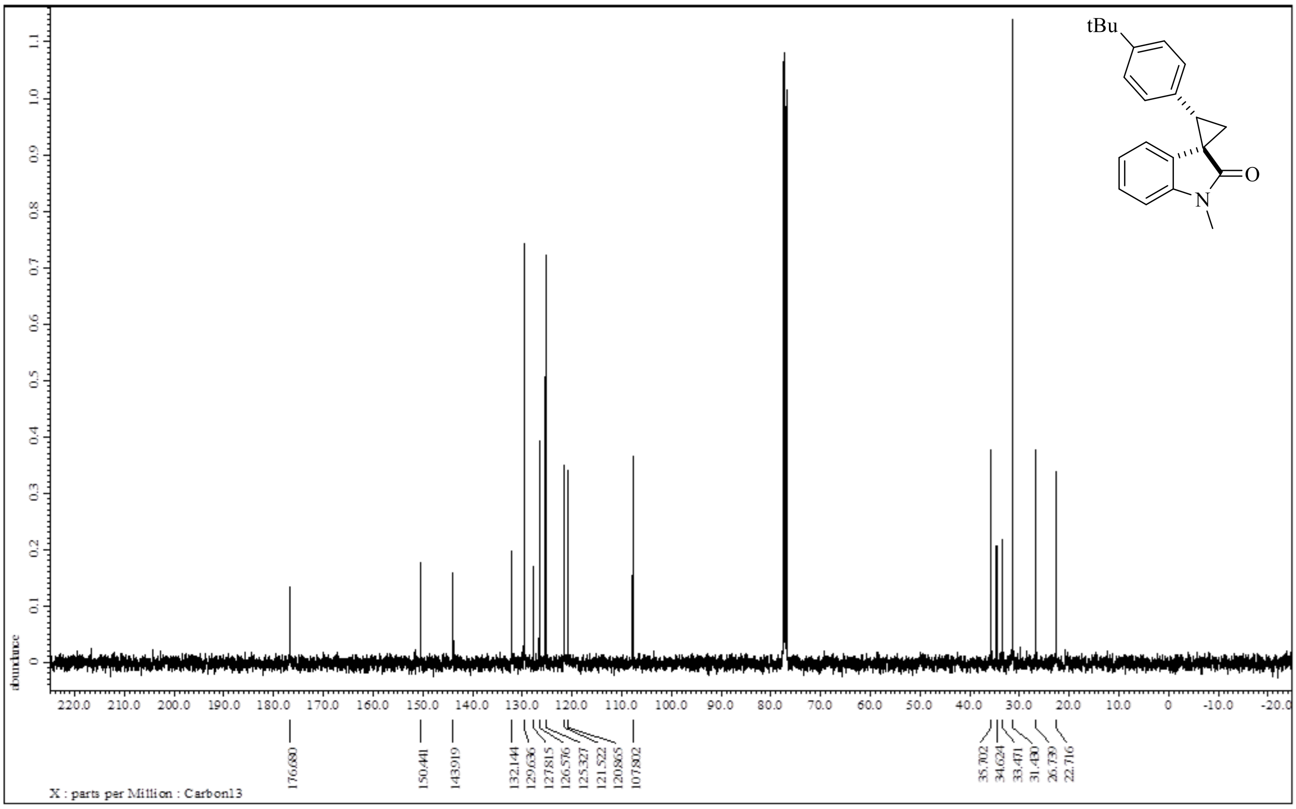
tBu
O
N
abundance
X : parts per Million : Carbon13
176.680
150.441
143.919
132.144
129.636
127.815
126.576
125.327
121.522
120.865
107.802
35.702
34.624
33.471
31.430
26.739
22.716

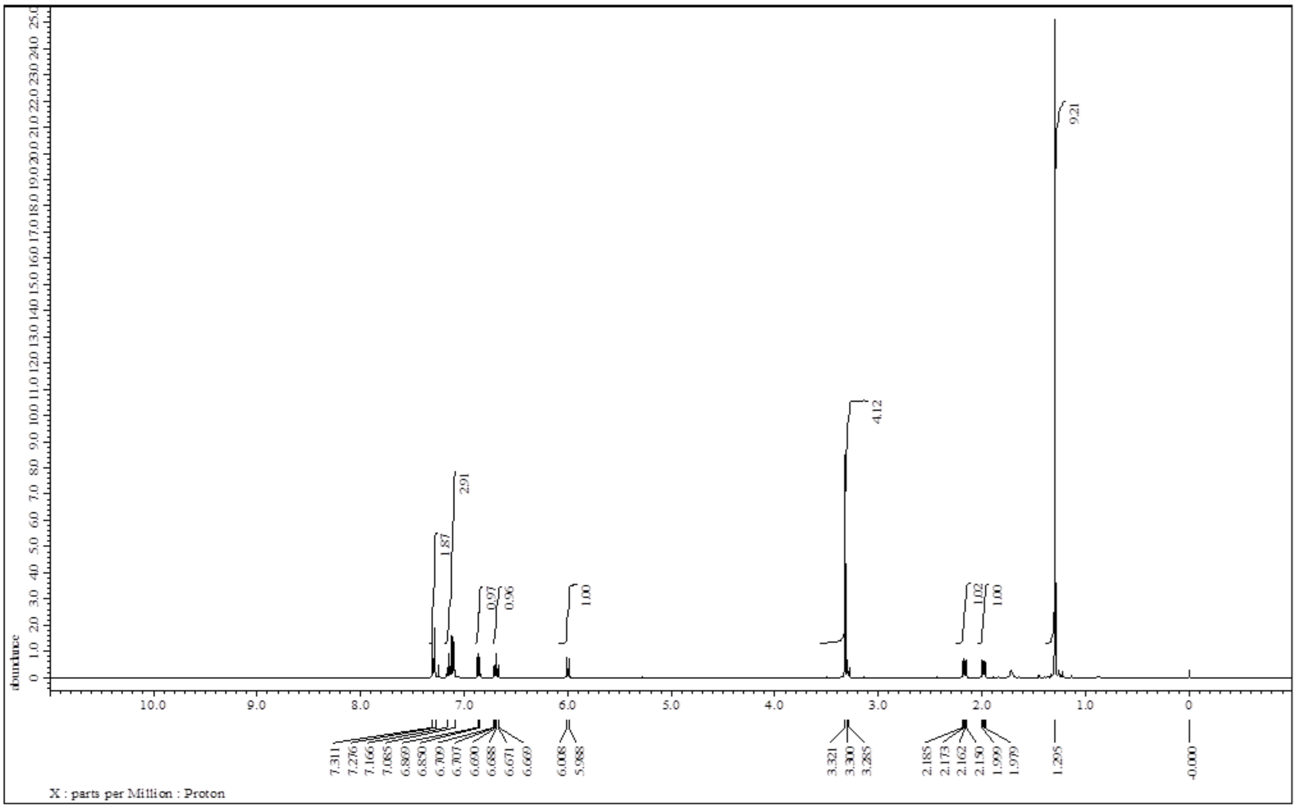
abundance
X : parts per Million : Proton
1.87
2.91
0.97
0.96
1.00
4.12
1.02
1.00
9.21
7.311
7.276
7.166
7.085
6.869
6.850
6.709
6.707
6.690
6.688
6.671
6.669
6.008
5.988
3.321
3.300
3.285
2.185
2.173
2.162
2.150
1.999
1.979
1.295
-0.000

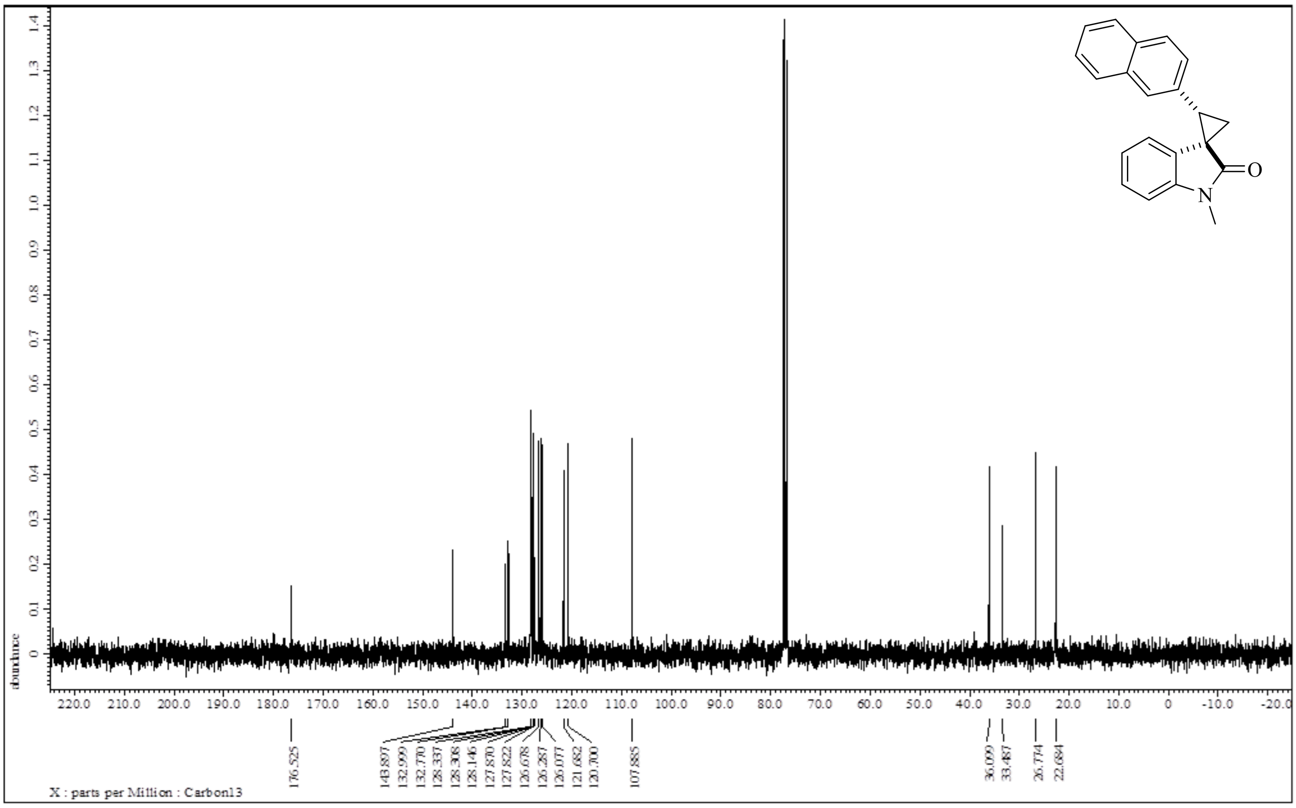

abundance
176.525
143.897
132.999
132.770
128.337
128.308
128.146
127.870
127.822
126.678
126.287
126.077
121.682
120.700
107.885
36.099
33.487
26.774
22.684
X : parts per Million : Carbon13
O
N

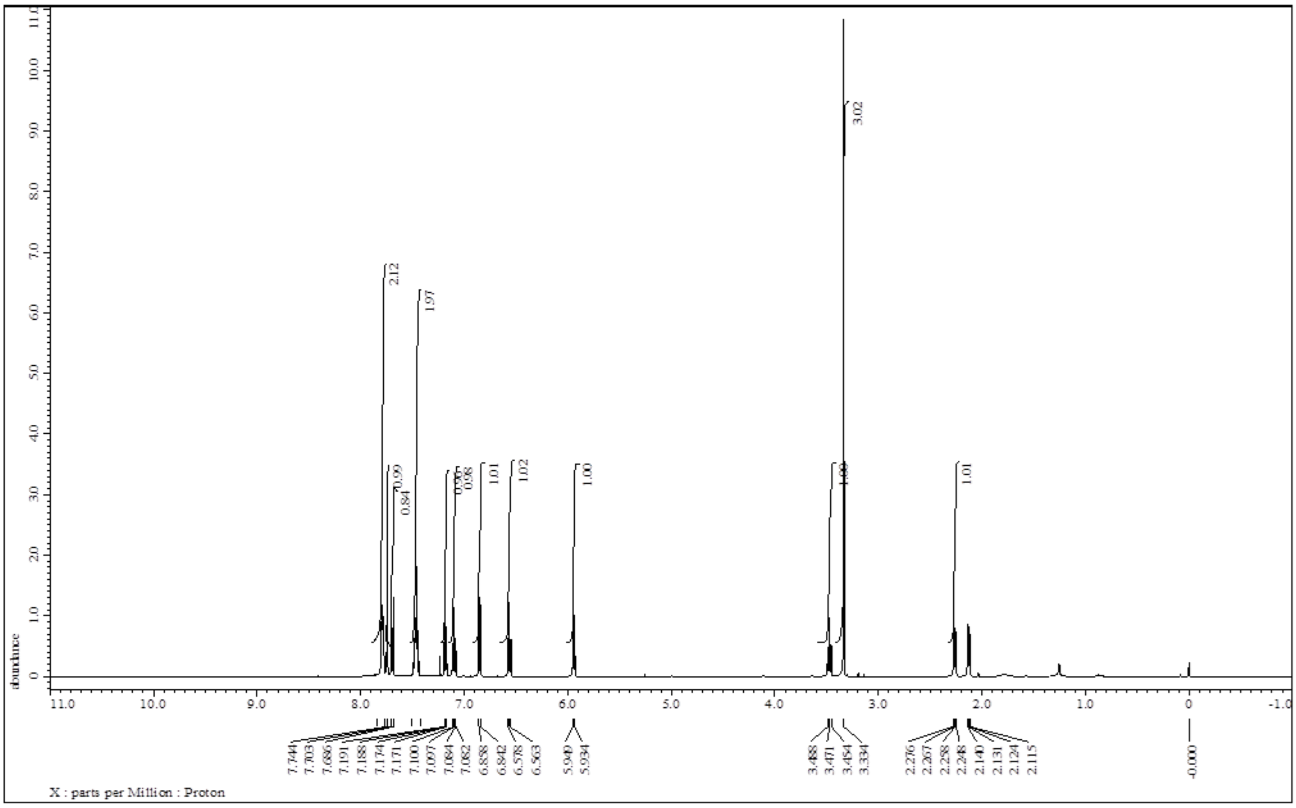

abundance
2.12
1.97
0.99
0.84
0.98
1.01
1.02
1.00
3.02
1.01
7.744
7.703
7.686
7.191
7.188
7.174
7.171
7.100
7.097
7.084
7.082
6.858
6.842
6.578
6.563
5.940
5.934
3.488
3.471
3.454
3.334
2.276
2.267
2.258
2.248
2.140
2.131
2.124
2.115
-0.000
X : parts per Million : Proton

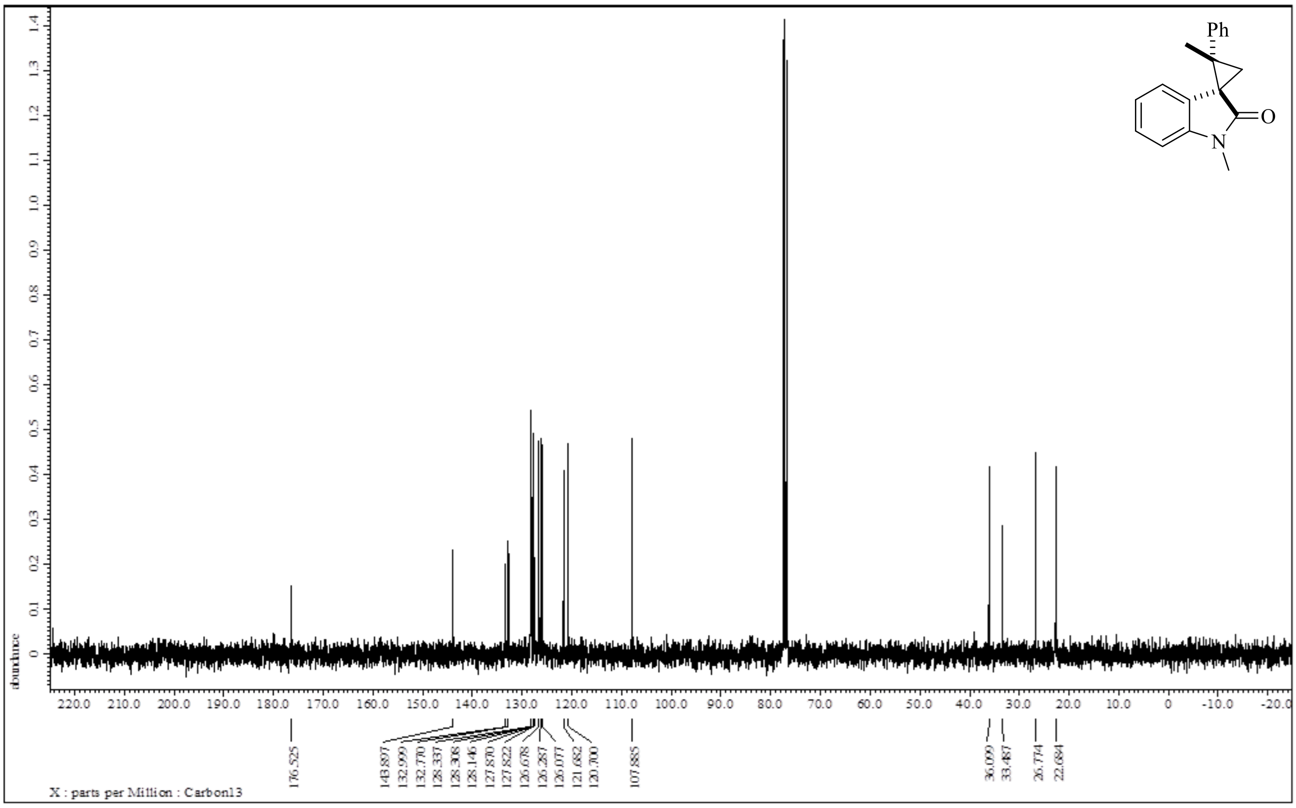

Ph
O
N
abundance
X : parts per Million : Carbon13
176.525
143.897
132.999
132.770
128.337
128.308
128.146
127.870
127.822
126.678
126.287
126.077
121.682
120.700
107.885
36.099
33.487
26.774
22.684

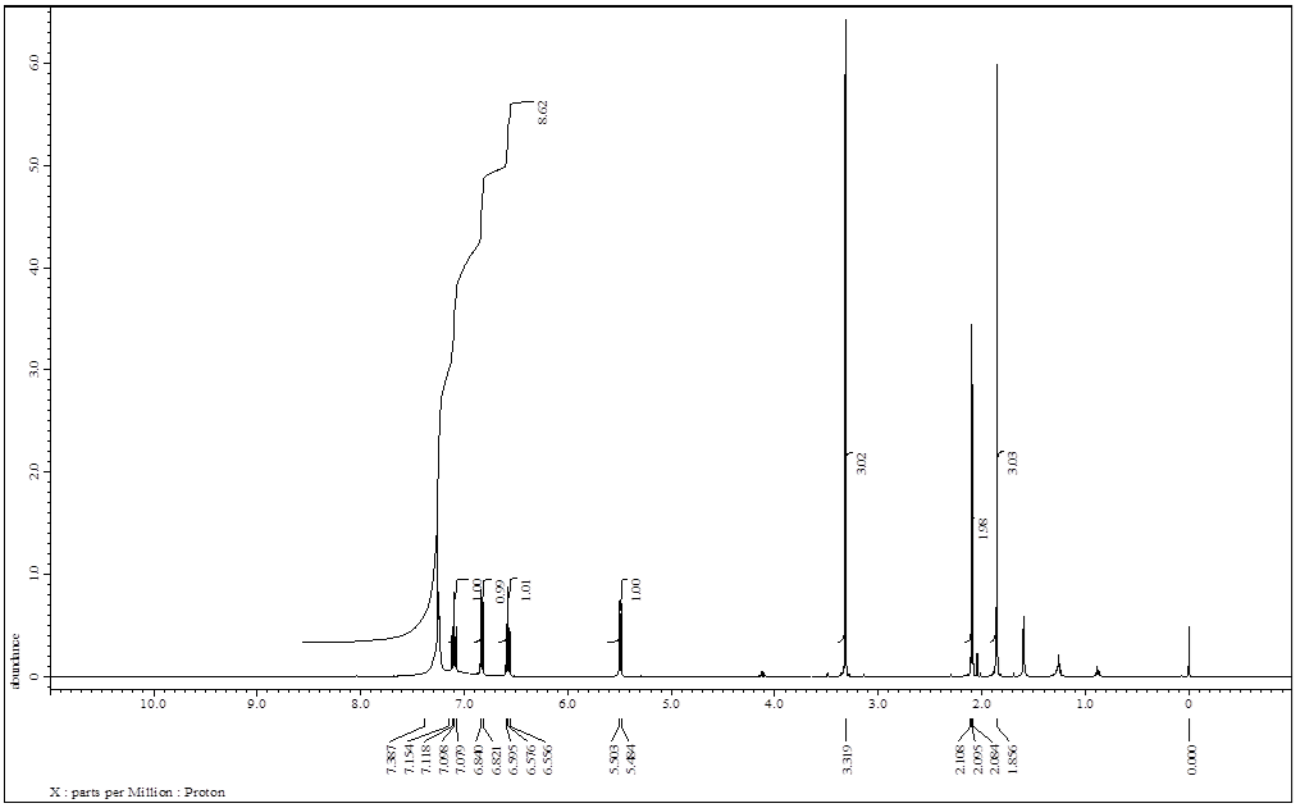

abundance
X : parts per Million : Proton
8.62
1.00
0.99
1.01
1.00
3.02
1.98
3.03
7.387
7.154
7.118
7.098
7.079
6.840
6.821
6.595
6.576
6.556
5.503
5.484
3.319
2.108
2.095
2.084
1.856
0.000

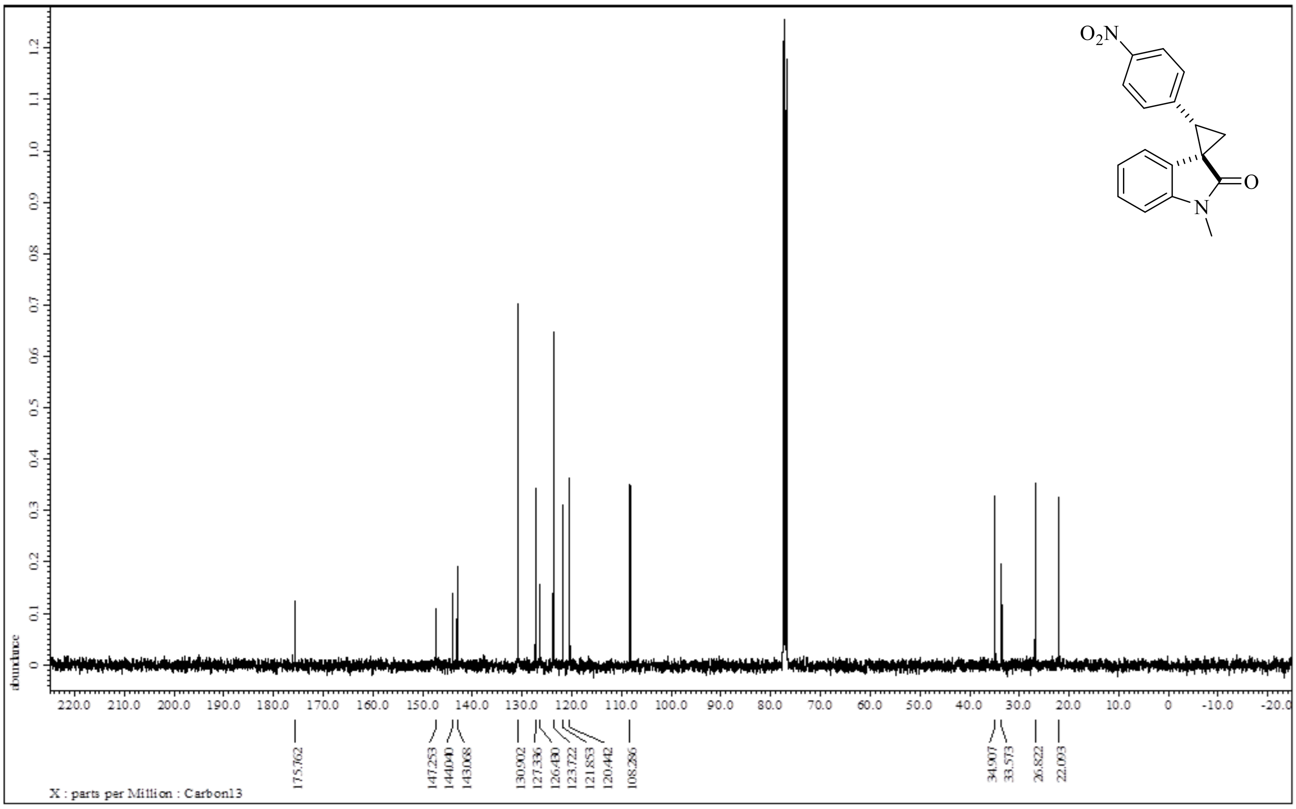
O_2N
O
N
abundance
X : parts per Million : Carbon13
175.762
147.253
144.040
143.068
130.902
127.336
126.430
123.722
121.853
120.442
108.286
34.907
33.573
26.822
22.093

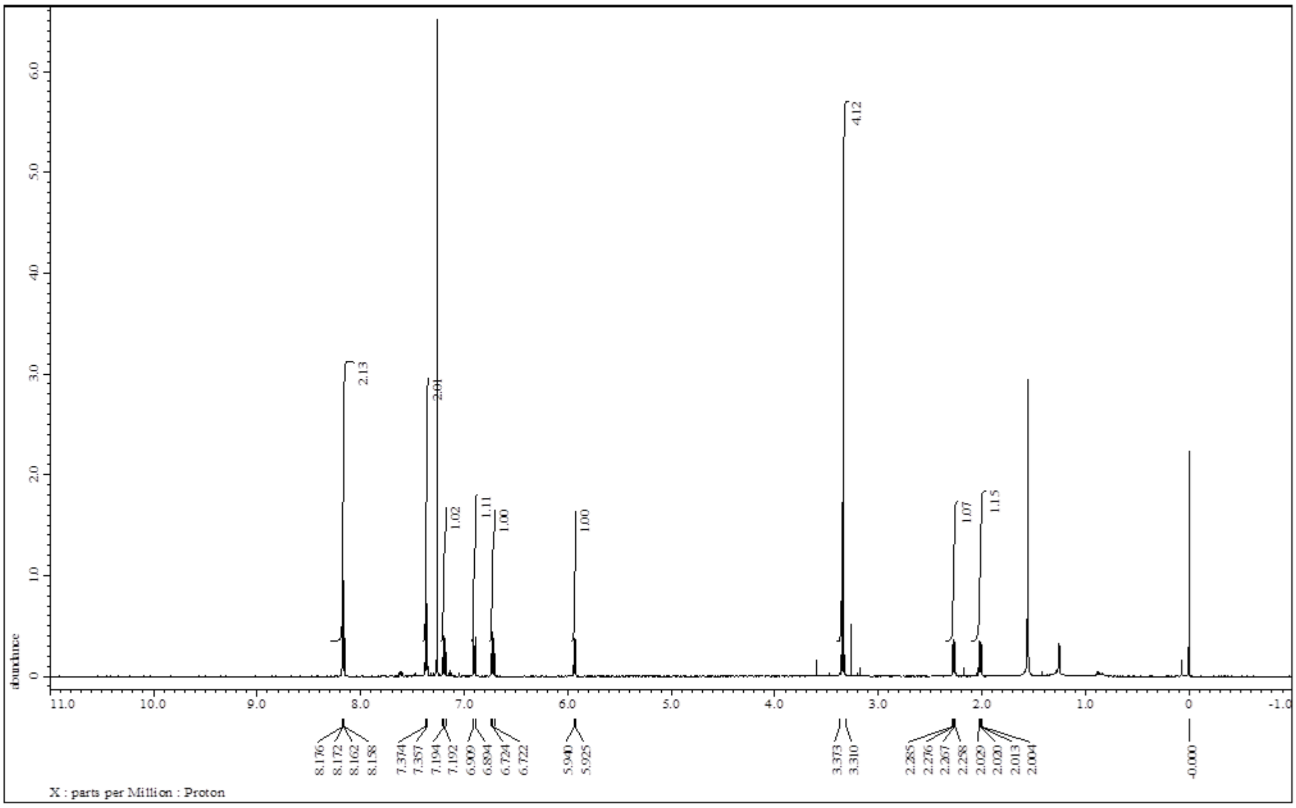
abundance
X : parts per Million : Proton
2.13
2.01
1.02
1.11
1.00
1.00
4.12
1.07
1.15
8.176
8.172
8.162
8.158
7.374
7.357
7.194
7.192
6.909
6.894
6.724
6.722
5.940
5.925
3.373
3.310
2.285
2.276
2.267
2.258
2.029
2.020
2.013
2.004
-0.000

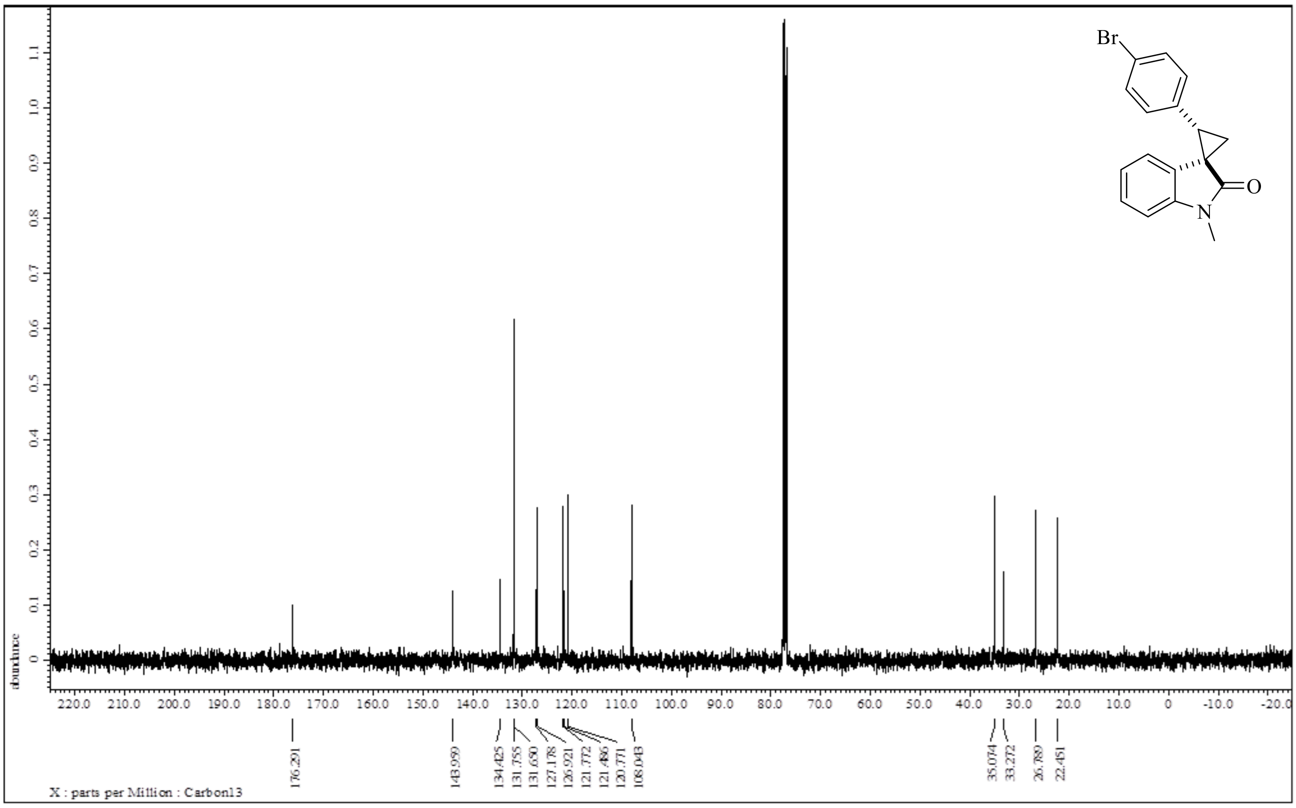
Br
N
O
abundance
176.291
143.959
134.425
131.755
131.650
127.178
126.921
121.772
121.486
120.771
108.043
35.074
33.272
26.789
22.451
X : parts per Million : Carbon13

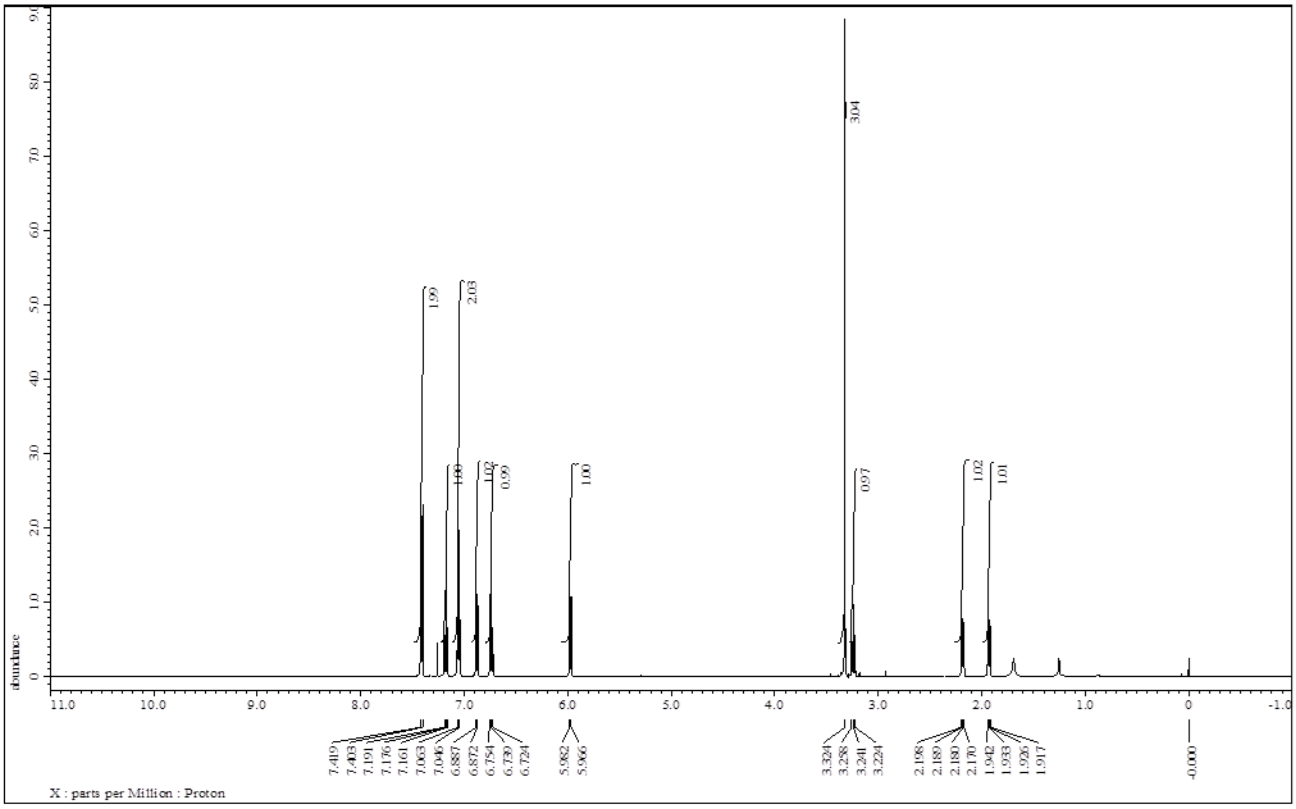
abundance
1.99
2.03
1.00
1.02
0.99
1.00
3.04
0.97
1.02
1.01
7.419
7.403
7.191
7.176
7.161
7.063
7.046
6.887
6.872
6.754
6.739
6.724
5.982
5.966
3.324
3.258
3.241
3.224
2.198
2.189
2.180
2.170
1.942
1.933
1.926
1.917
-0.000
X : parts per Million : Proton

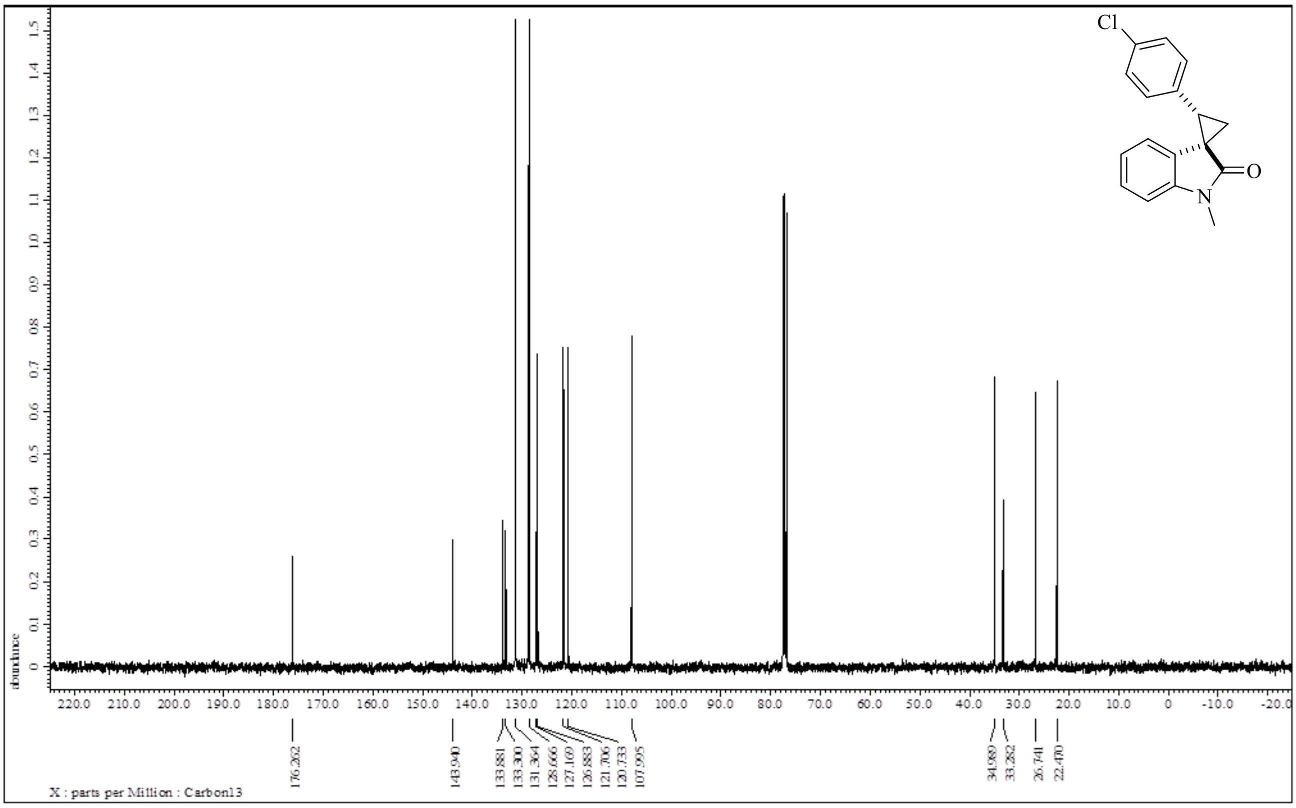

Cl
N
O
X : parts per Million : Carbon13
abundance
176.262
143.940
133.881
133.300
131.364
128.666
127.169
126.883
121.706
120.733
107.995
34.989
33.282
26.741
22.470

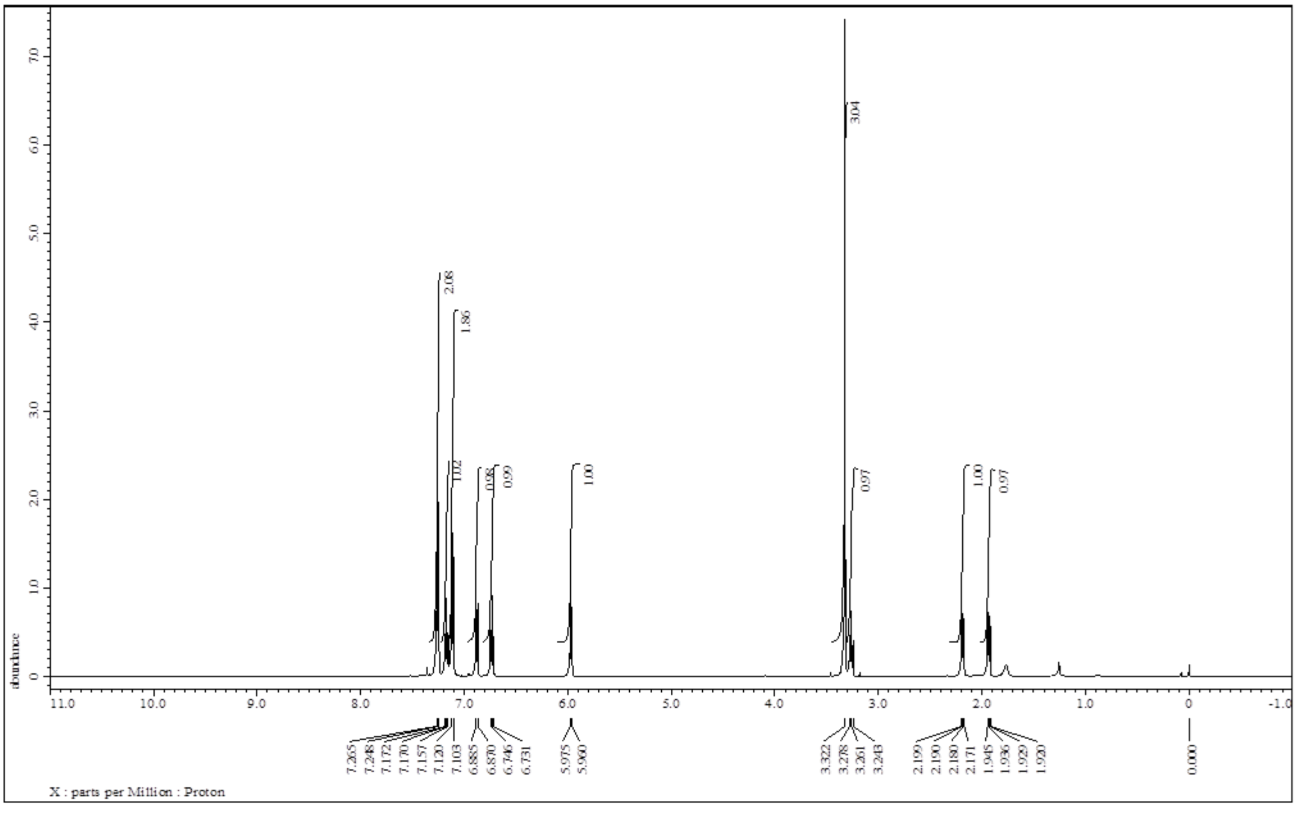

X : parts per Million : Proton
abundance
3.04
2.08
1.86
1.02
0.98
0.99
1.00
0.97
1.00
0.97
7.265
7.248
7.172
7.170
7.157
7.120
7.103
6.885
6.870
6.746
6.731
5.975
5.960
3.322
3.278
3.261
3.243
2.199
2.190
2.180
2.171
1.945
1.936
1.929
1.920
0.000

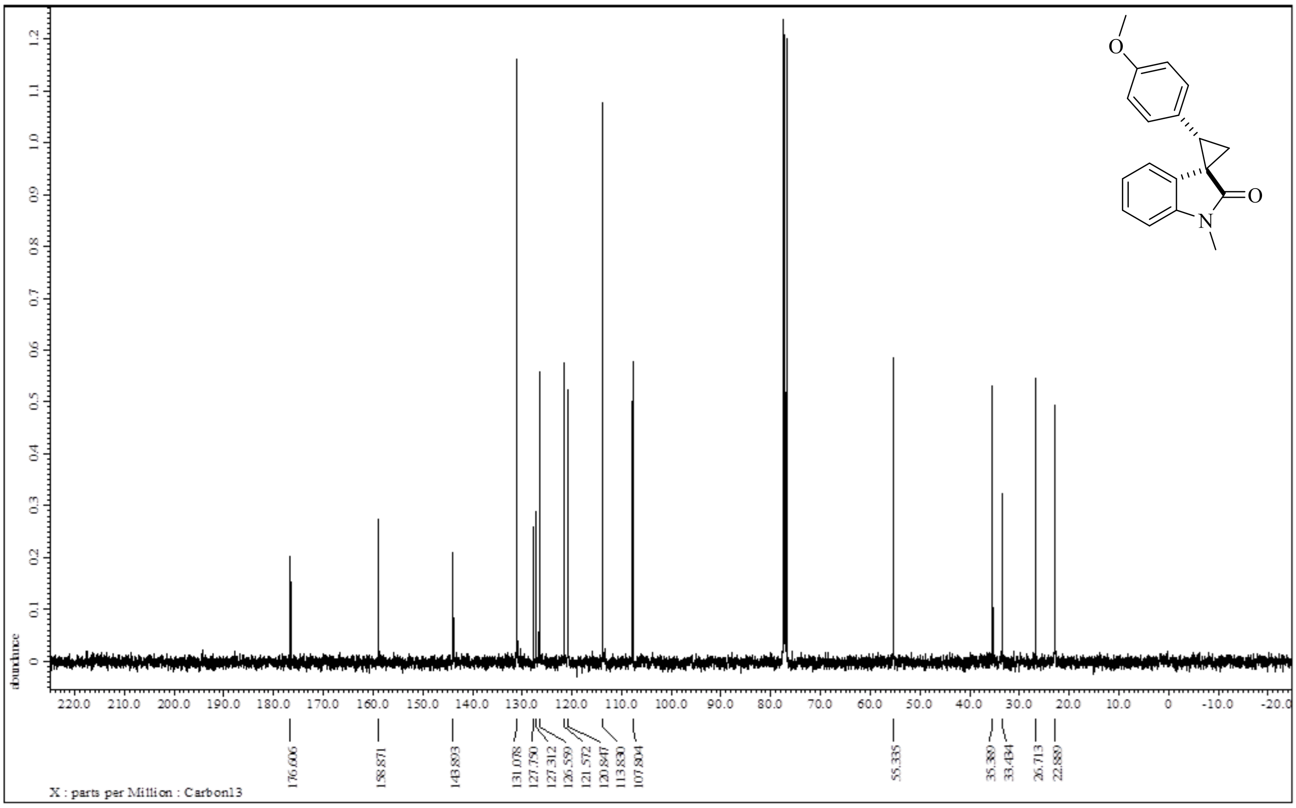
abundance
X : parts per Million : Carbon13
176.606
158.871
143.893
131.078
127.750
127.312
126.599
121.572
120.847
113.830
107.804
55.335
35.389
33.434
26.713
22.889
O
N

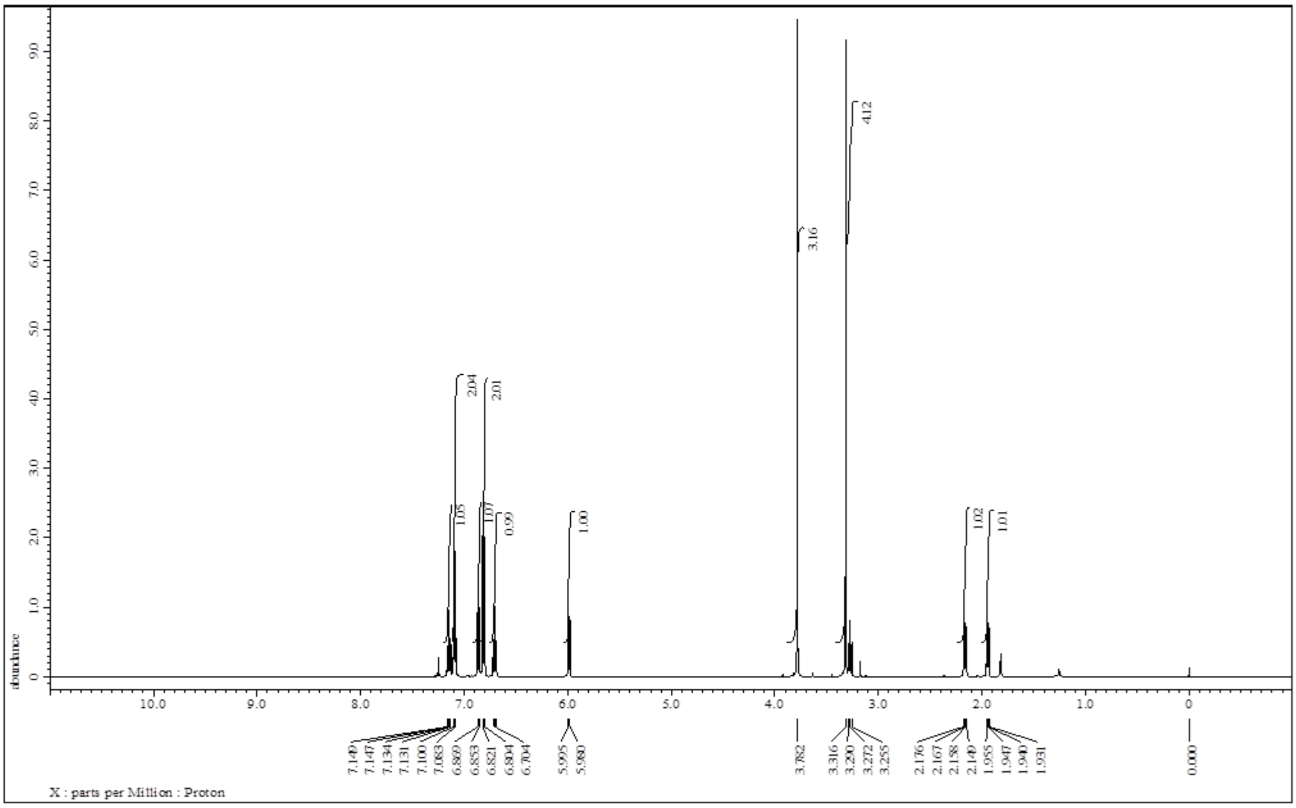
abundance
X : parts per Million : Proton
2.04
2.01
1.05
1.07
0.99
1.00
3.16
4.12
1.02
1.01
7.149
7.147
7.134
7.131
7.100
7.083
6.869
6.853
6.821
6.804
6.704
5.995
5.980
3.782
3.316
3.290
3.272
3.255
2.176
2.167
2.158
2.149
1.955
1.947
1.940
1.931
0.000

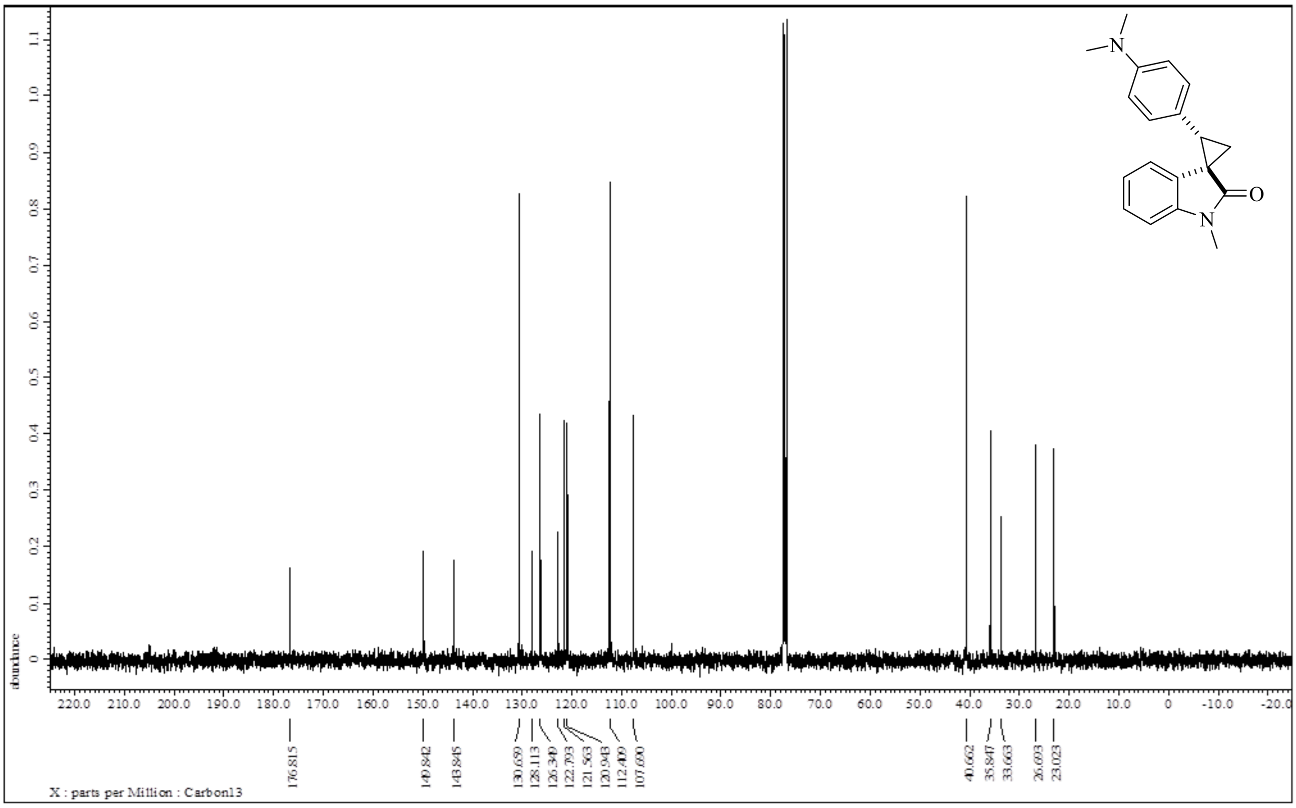
X : parts per Million : Carbon13
abundance
176.815
149.842
143.845
130.699
128.113
126.349
122.793
121.563
120.943
112.409
107.690
40.662
35.847
33.663
26.693
23.023
N
O

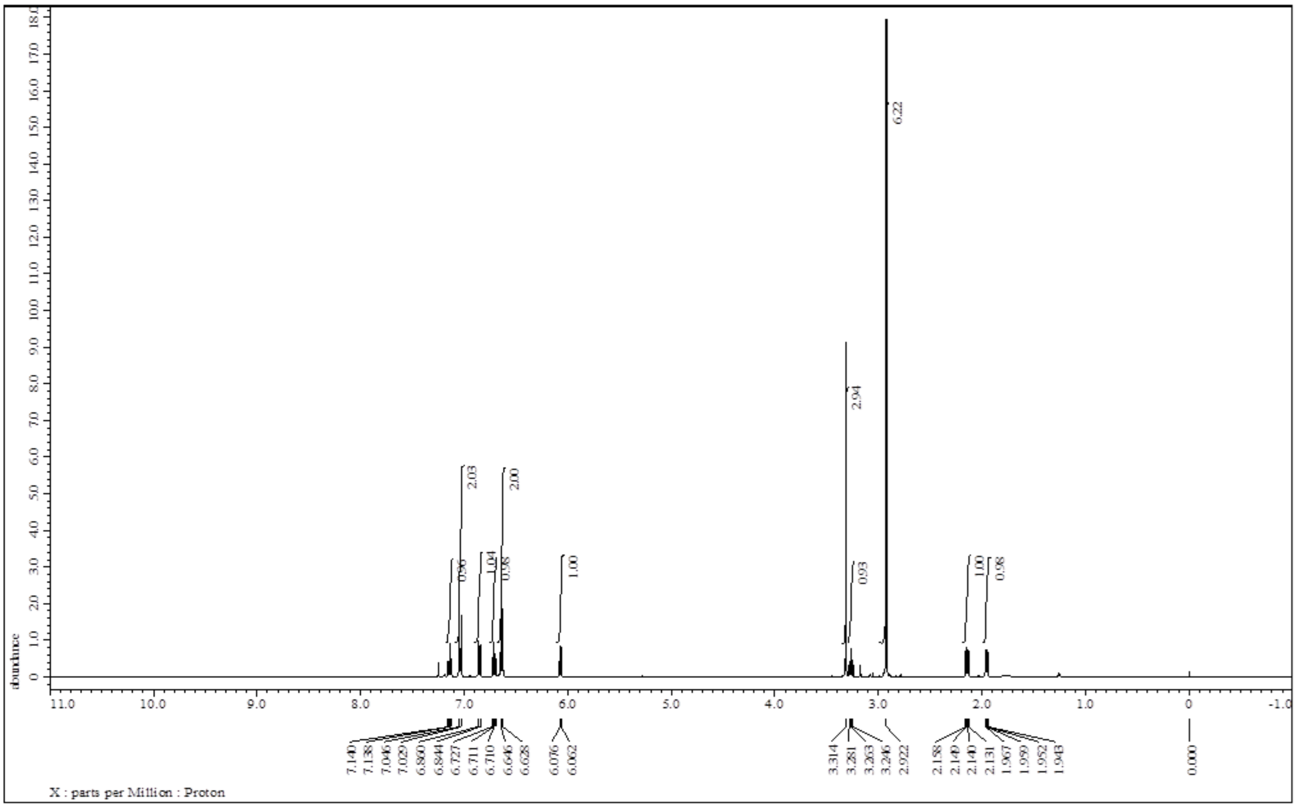
X : parts per Million : Proton
abundance
6.22
2.94
2.03
2.00
1.04
0.96
0.98
1.00
0.93
1.00
0.98
7.140
7.138
7.046
7.029
6.860
6.844
6.727
6.711
6.710
6.646
6.628
6.076
6.062
3.314
3.281
3.263
3.246
2.922
2.158
2.149
2.140
2.131
1.967
1.959
1.952
1.943
0.000

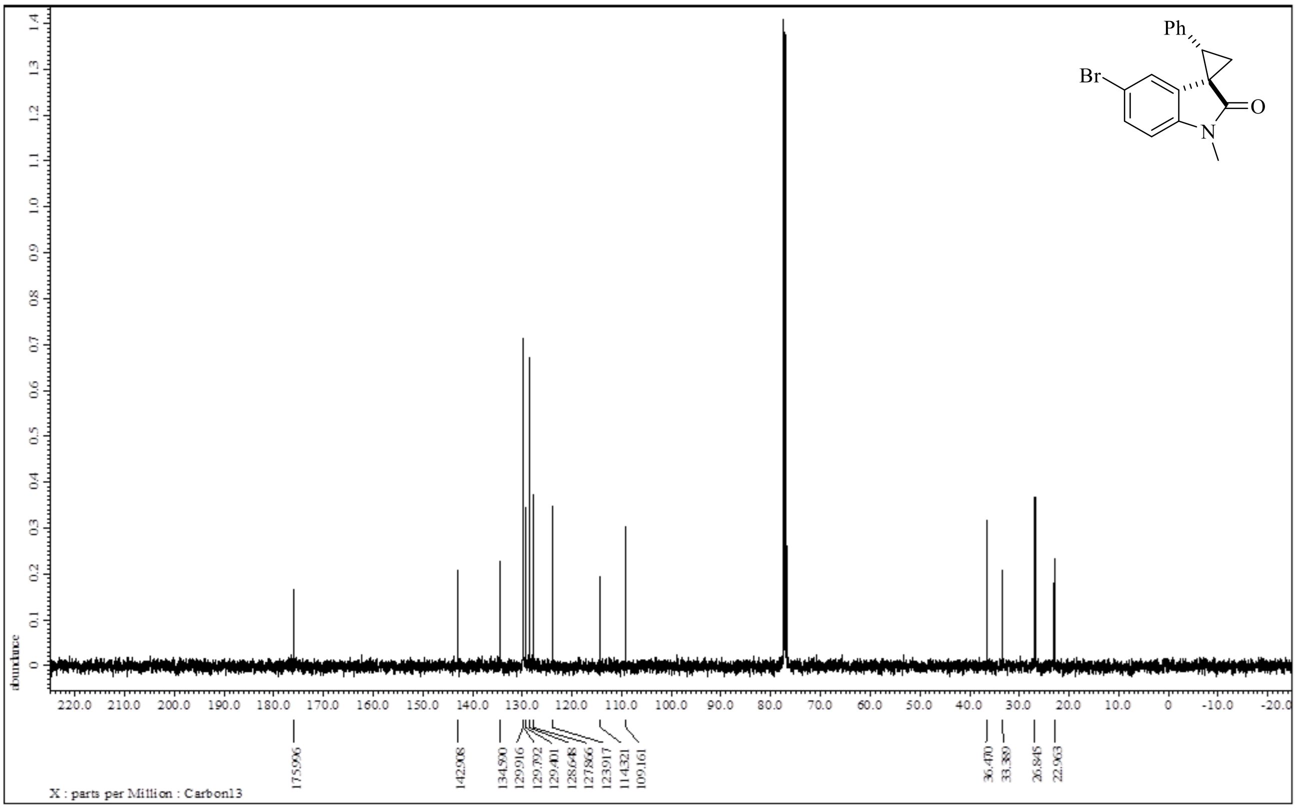
Ph
Br
O
N
175.996
142.908
134.590
129.916
129.792
129.401
128.648
127.866
123.917
114.321
109.161
36.470
33.389
26.845
22.963
X : parts per Million : Carbon13
abundance

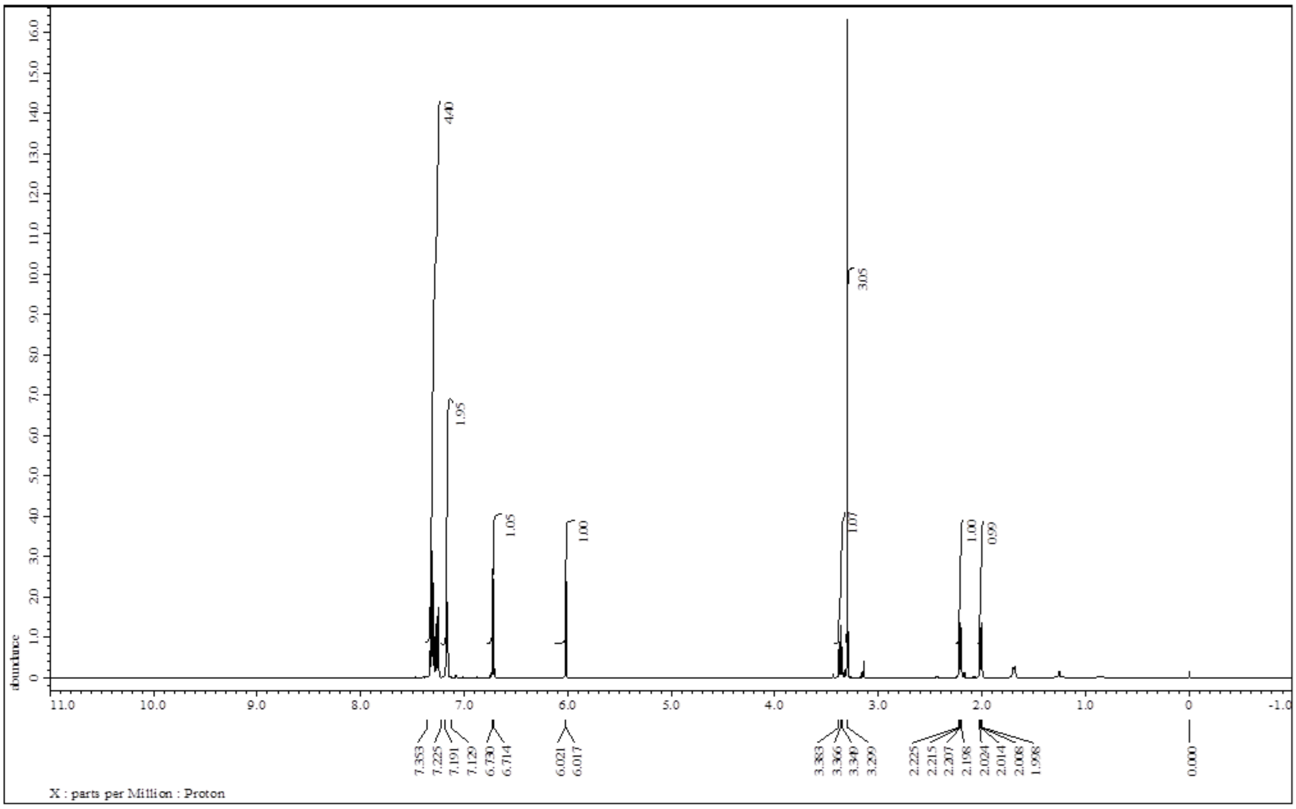
4.40
1.95
1.05
1.00
1.07
3.05
1.00
0.99
7.353
7.225
7.191
7.129
6.730
6.714
6.021
6.017
3.383
3.366
3.349
3.299
2.225
2.215
2.207
2.198
2.024
2.014
2.008
1.998
0.000
X : parts per Million : Proton
abundance

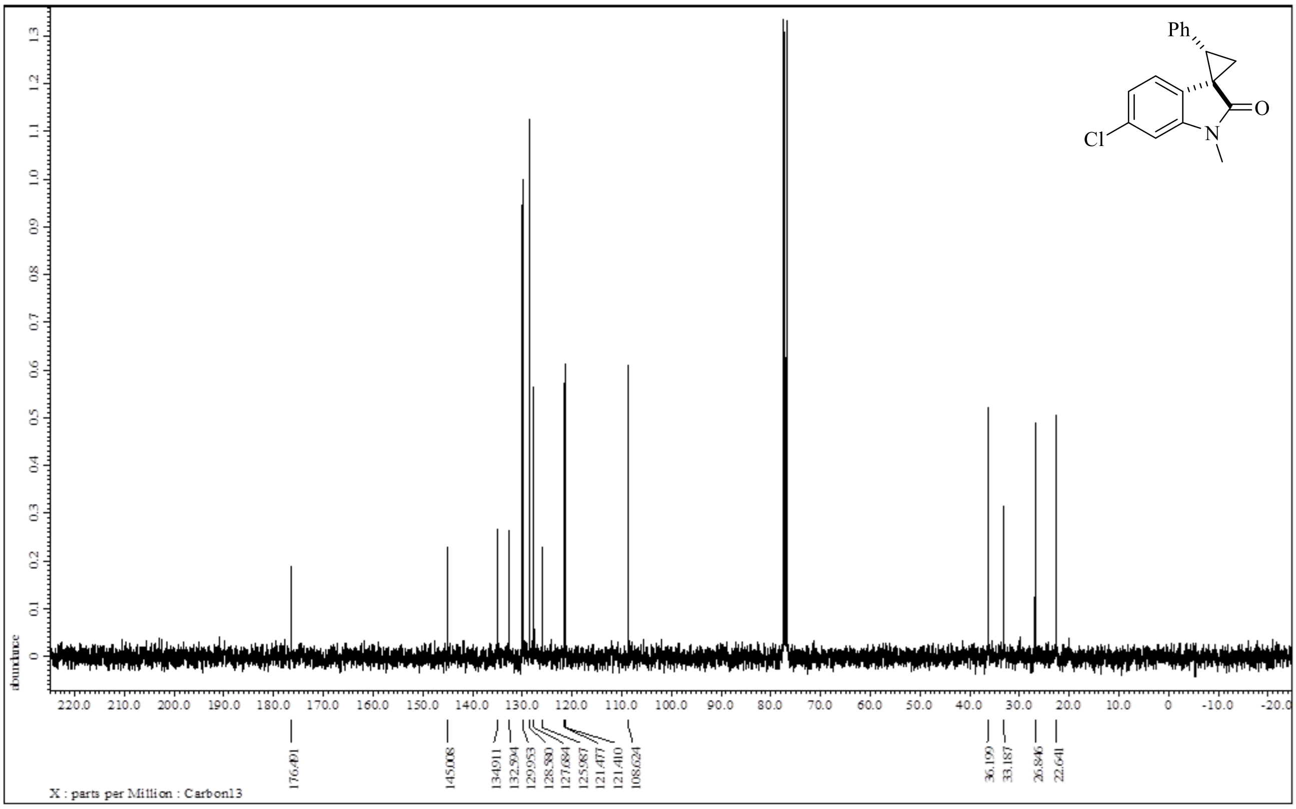
Ph
Cl
N
O
abundance
X : parts per Million : Carbon13
176.491
145.008
134.911
132.994
129.953
128.580
127.684
125.987
121.477
121.410
108.624
36.199
33.187
26.846
22.641

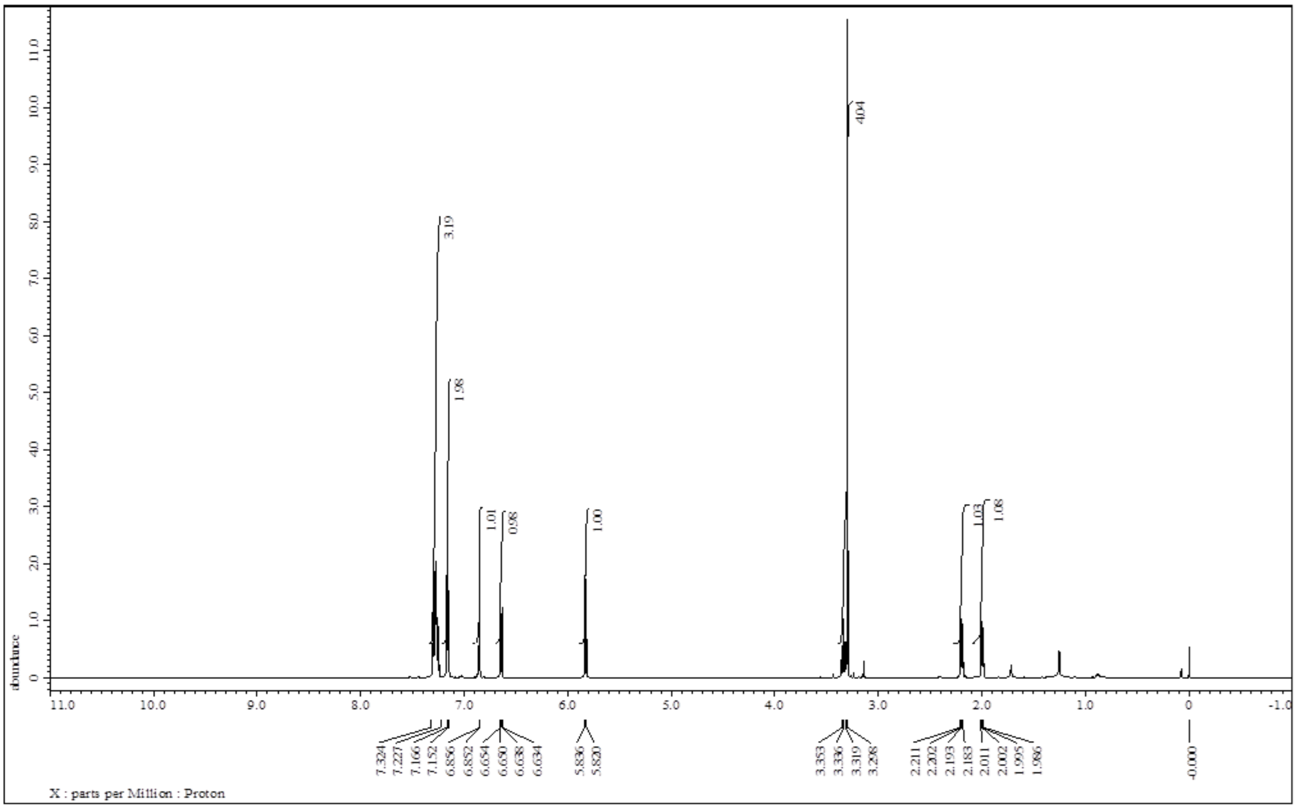
abundance
X : parts per Million : Proton
3.19
1.98
1.01
0.98
1.00
4.04
1.03
1.08
7.324
7.227
7.166
7.152
6.856
6.852
6.654
6.650
6.638
6.634
5.836
5.820
3.353
3.336
3.319
3.298
2.211
2.202
2.193
2.183
2.011
2.002
1.995
1.986
-0.000

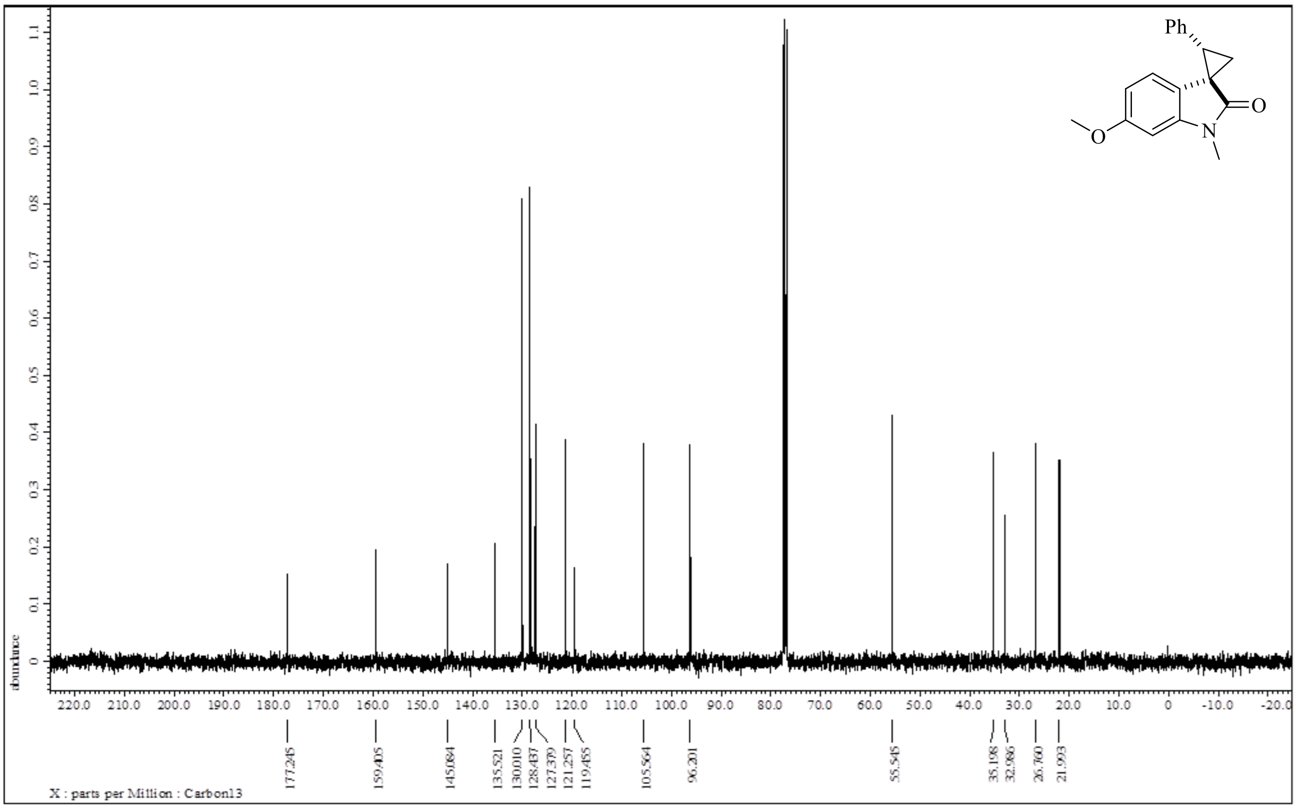
Ph
O
N
abundance
177.245
159.405
145.084
135.521
130.010
128.437
127.379
121.257
119.455
105.564
96.201
55.545
35.198
32.986
26.760
21.993
X : parts per Million : Carbon13

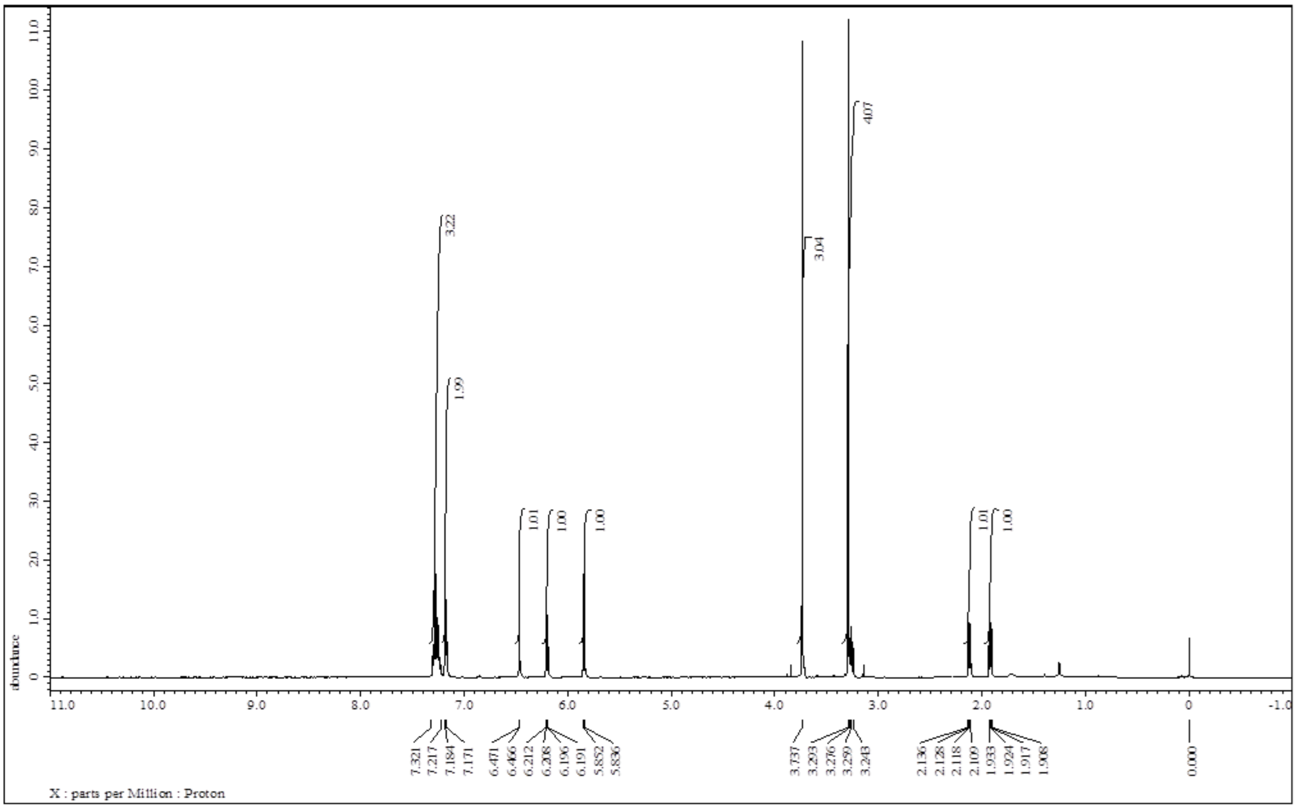
abundance
3.22
1.99
1.01
1.00
1.00
3.04
4.07
1.01
1.00
7.321
7.217
7.184
7.171
6.471
6.466
6.212
6.208
6.196
6.191
5.852
5.836
3.737
3.293
3.276
3.259
3.243
2.136
2.128
2.118
2.109
1.933
1.924
1.917
1.908
0.000
X : parts per Million : Proton

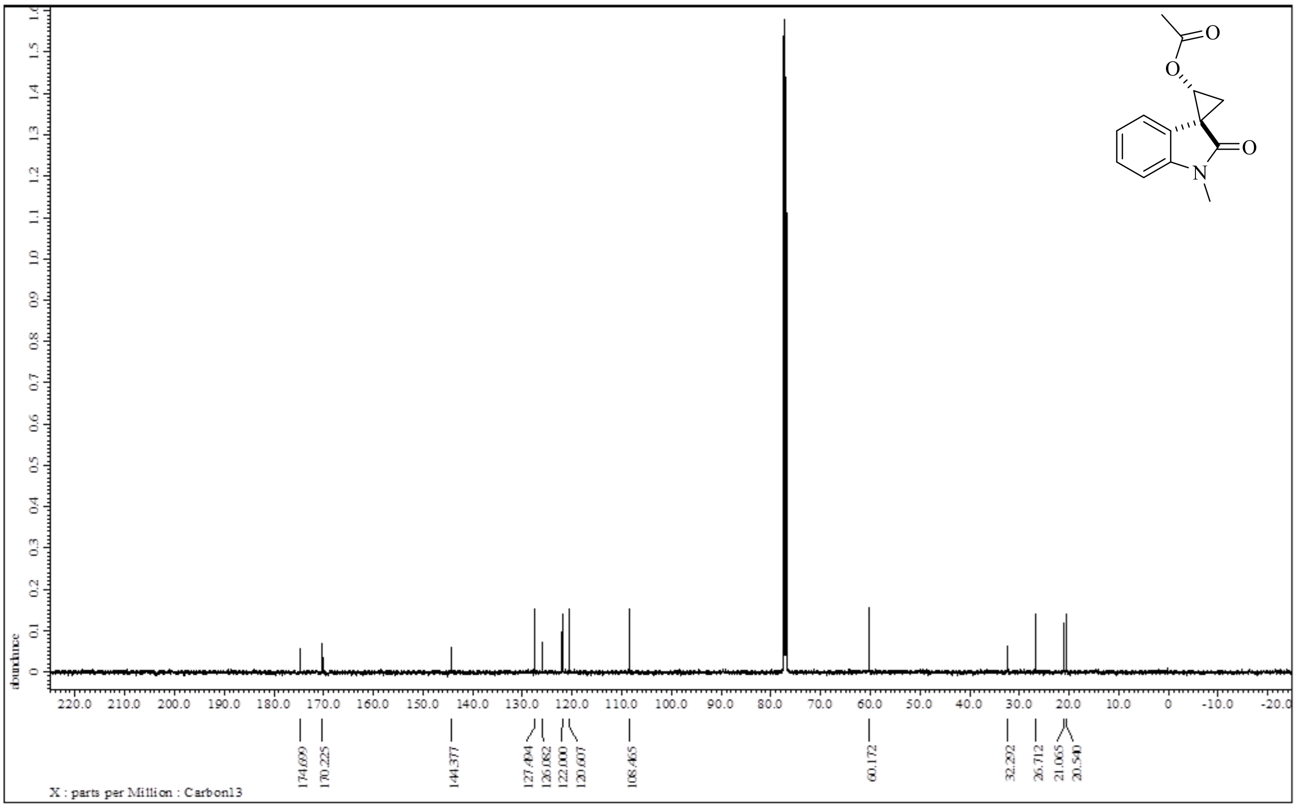

X : parts per Million : Carbon13
abundance
174.699
170.225
144.377
127.494
126.082
122.000
120.607
108.465
60.172
32.292
26.712
21.065
20.540
O
O
O
N

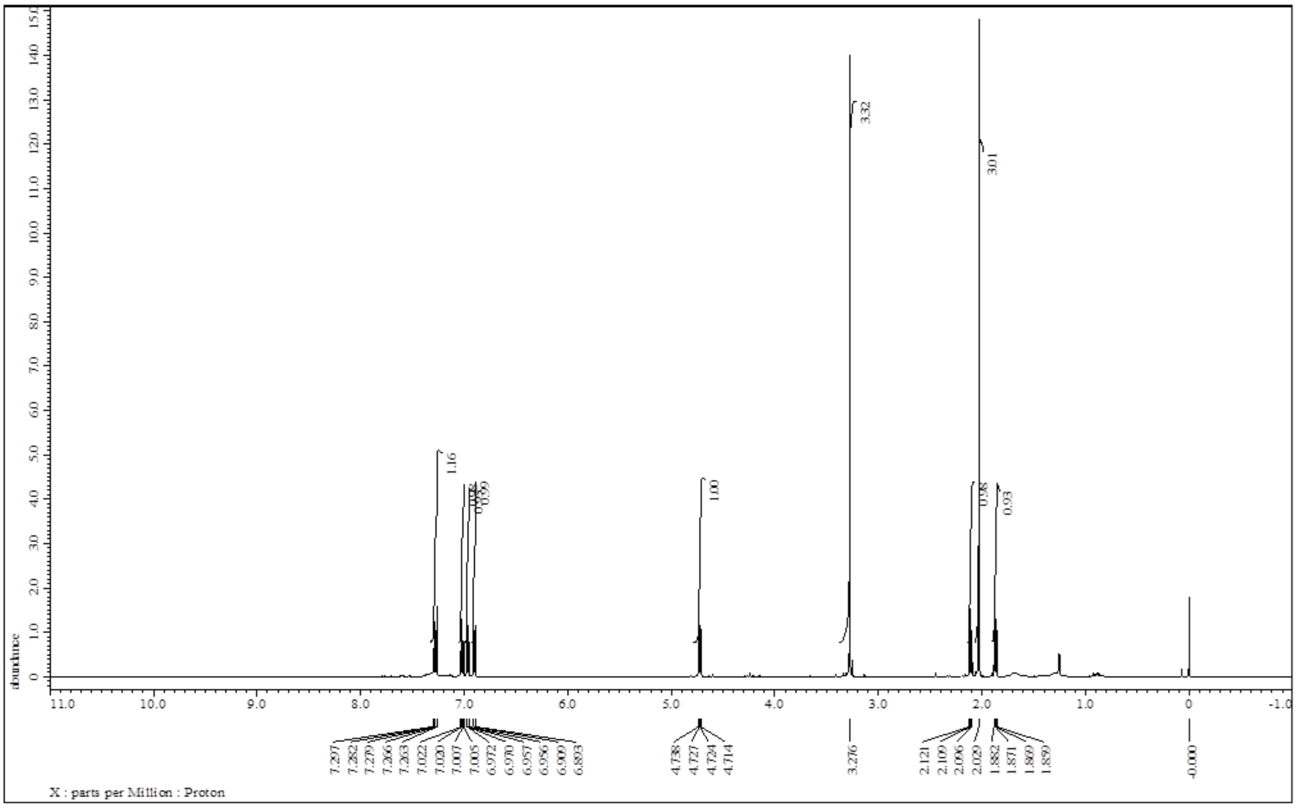

X : parts per Million : Proton
abundance
3.32
3.01
1.16
1.00
0.93
3.276
-0.000

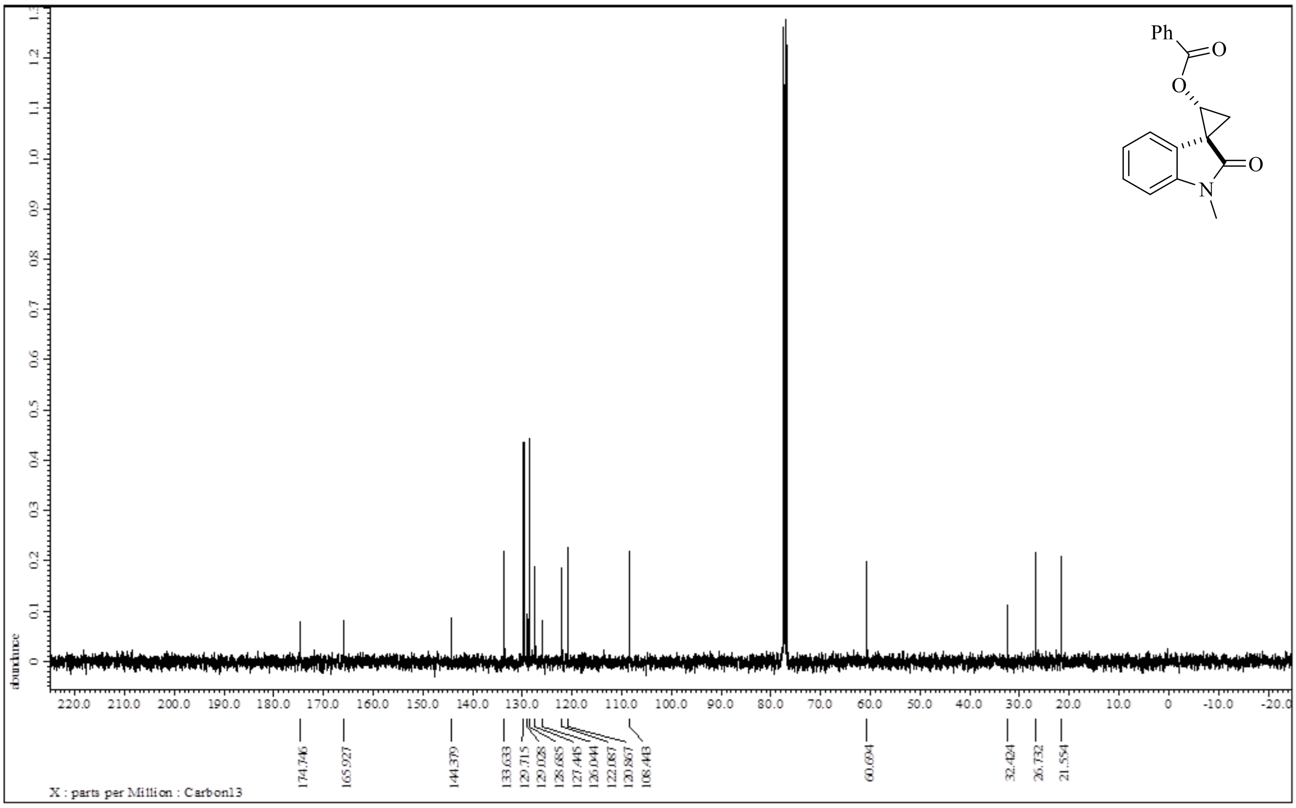
Ph
O
O
N
O
abundance
X : parts per Million : Carbon13
174.746
165.927
144.379
133.633
129.715
129.028
128.685
127.445
126.044
122.087
120.867
108.443
60.694
32.424
26.732
21.554

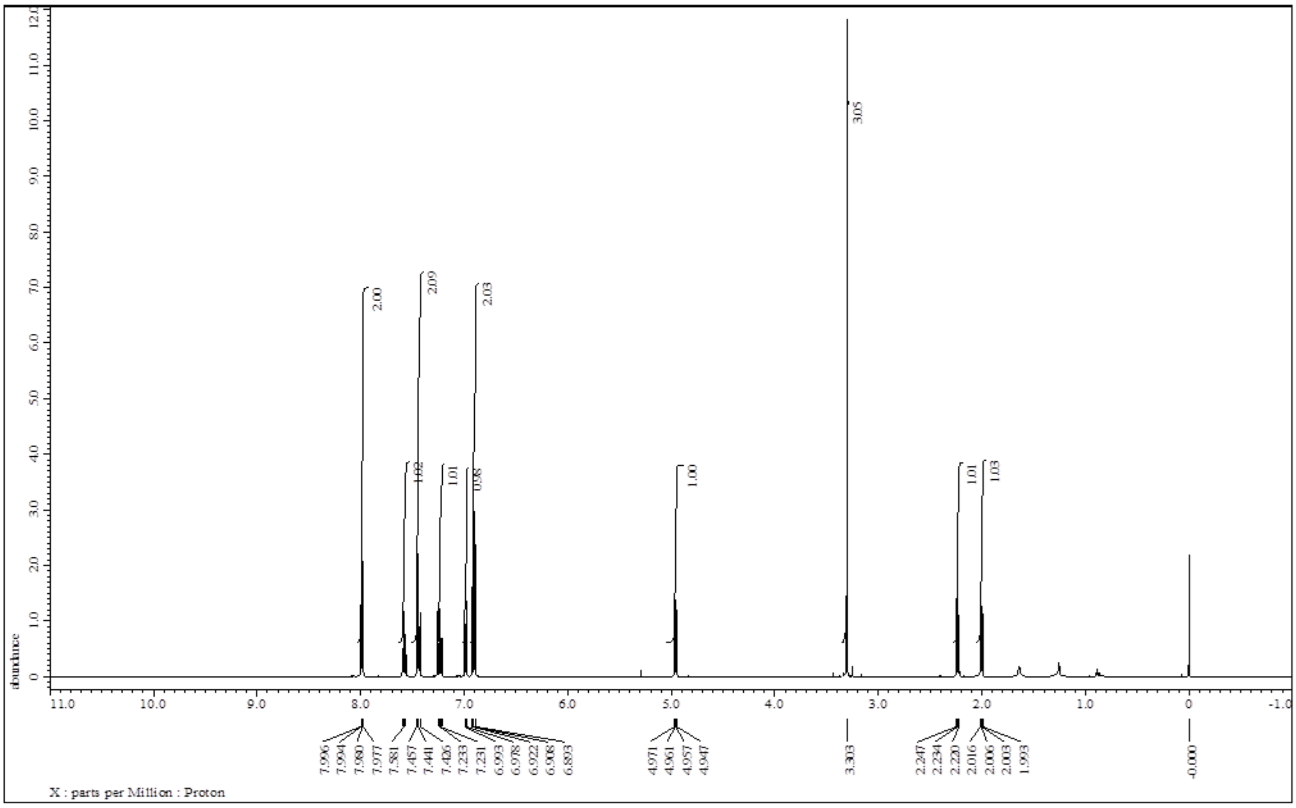
abundance
X : parts per Million : Proton
2.00
1.02
2.09
1.01
0.98
2.03
1.00
3.05
1.01
1.03
7.996
7.994
7.980
7.977
7.581
7.457
7.441
7.426
7.233
7.231
6.993
6.978
6.922
6.908
6.893
4.971
4.961
4.957
4.947
3.303
2.247
2.234
2.220
2.016
2.006
2.003
1.993
-0.000

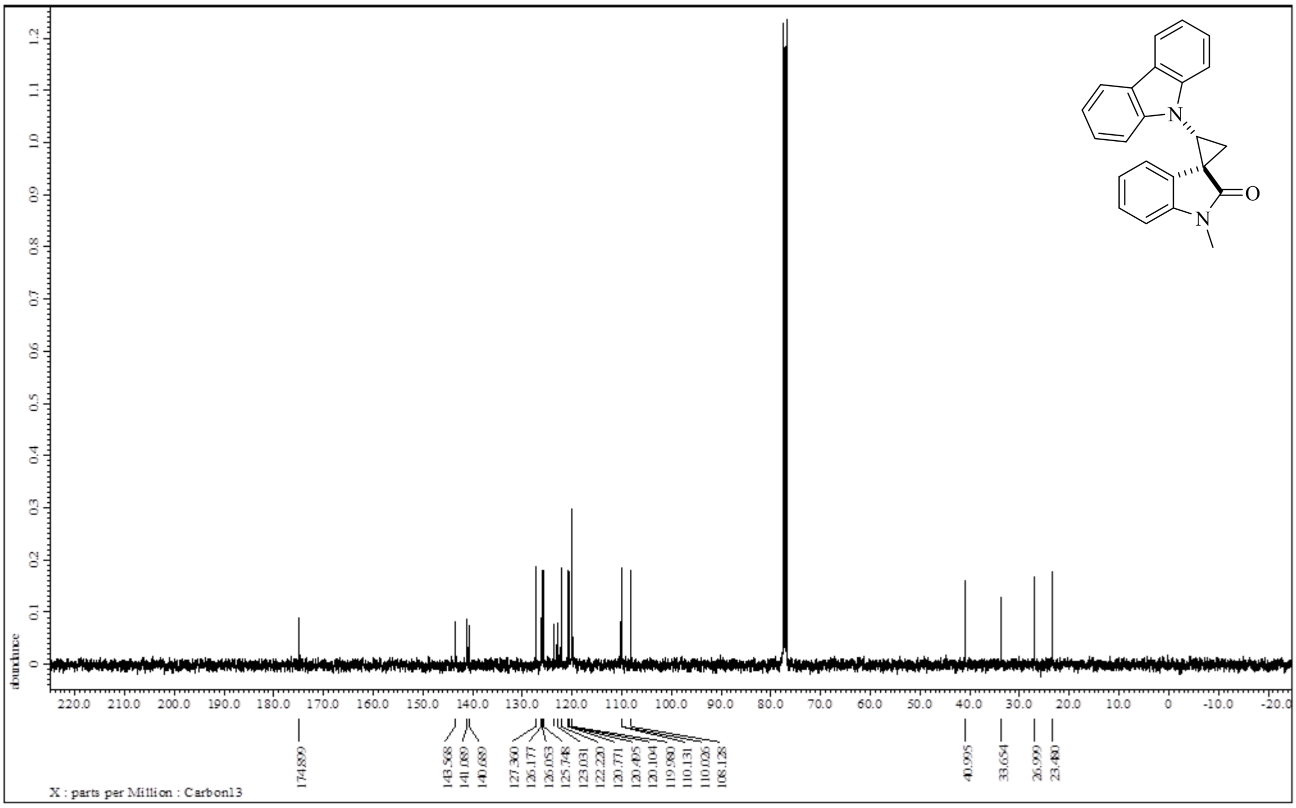
abundance
174.899
143.568
141.089
140.689
127.360
126.177
126.053
125.748
123.031
122.220
120.771
120.405
120.104
119.980
110.131
110.026
108.128
40.995
33.654
26.999
23.480
N
O
X : parts per Million : Carbon13

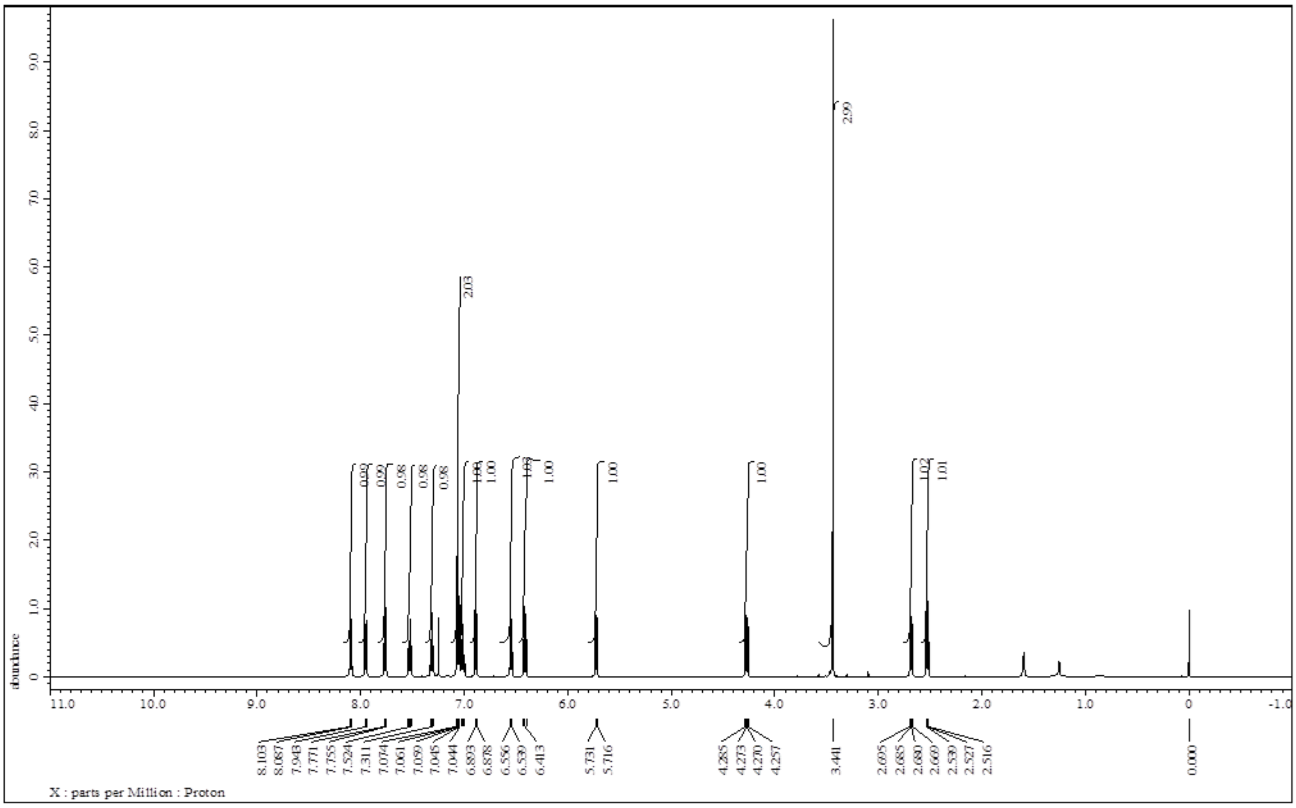
abundance
2.99
2.03
8.103
8.087
7.943
7.771
7.755
7.524
7.311
7.074
7.061
7.059
7.045
7.044
6.893
6.878
6.556
6.539
6.413
5.731
5.716
4.285
4.273
4.270
4.257
3.441
2.695
2.685
2.680
2.669
2.539
2.527
2.516
0.000
X : parts per Million : Proton

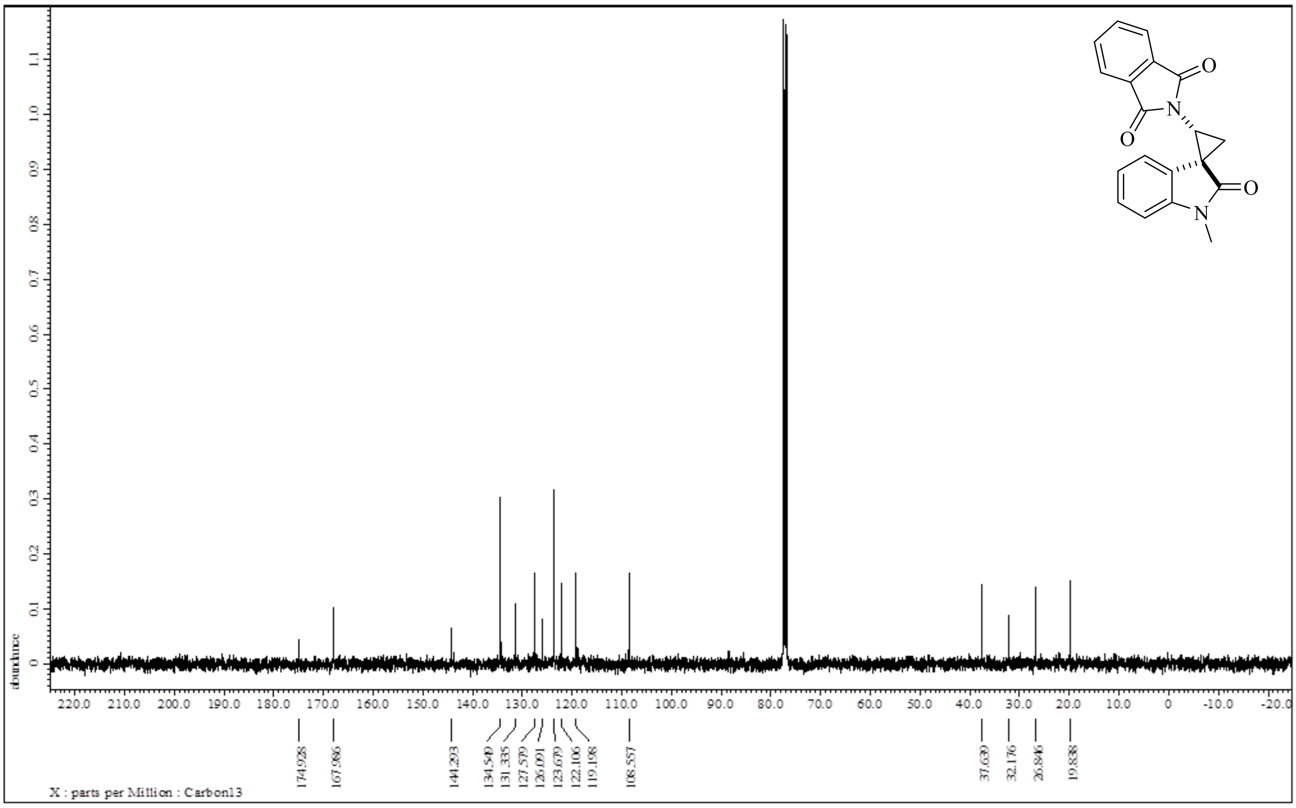
X : parts per Million : Carbon13
abundance
174.928
167.986
144.293
134.549
131.335
127.579
126.091
123.679
122.106
119.198
108.557
37.639
32.176
26.846
19.838
O
N
O
O
N

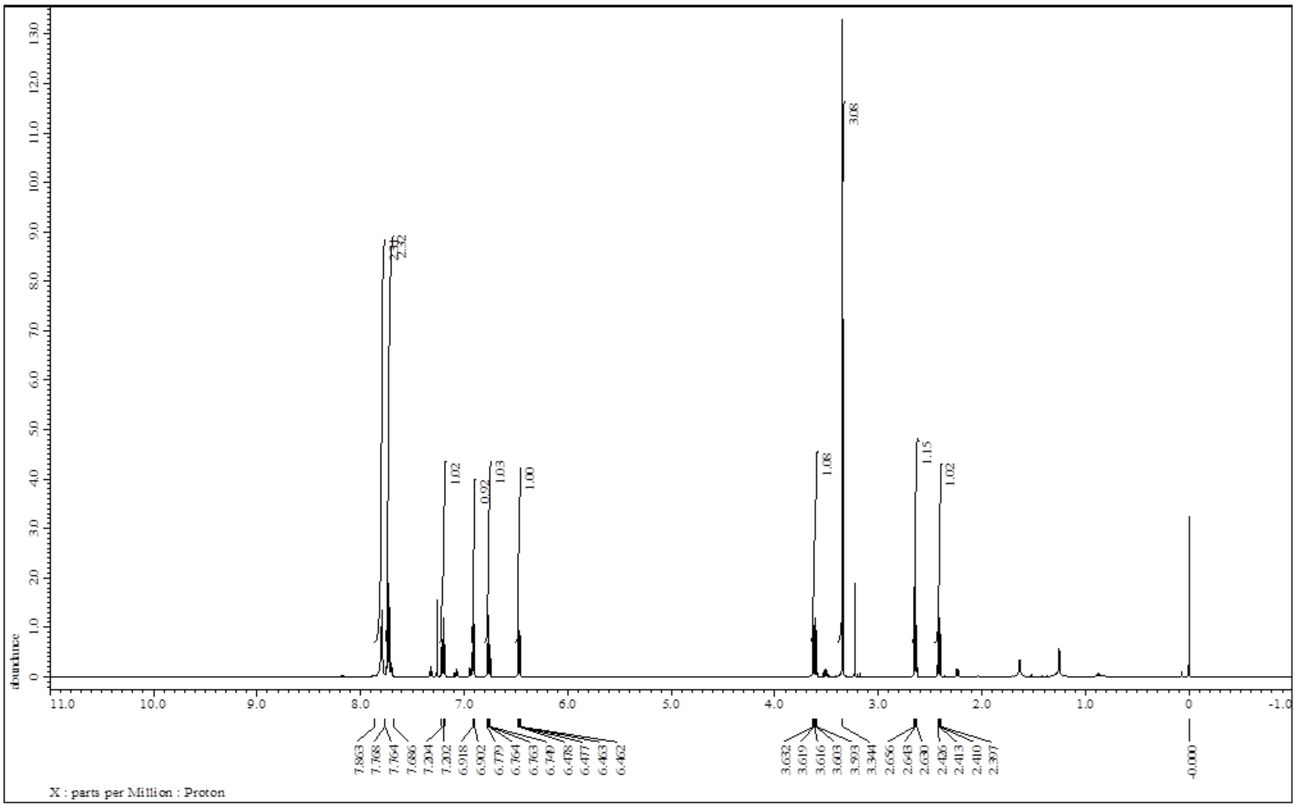
X : parts per Million : Proton
abundance
2.32
2.32
1.02
0.92
1.03
1.00
1.08
3.08
1.15
1.02
7.863
7.768
7.764
7.686
7.204
7.202
6.918
6.902
6.779
6.764
6.763
6.749
6.478
6.477
6.463
6.462
3.632
3.619
3.616
3.603
3.593
3.344
2.656
2.643
2.630
2.426
2.413
2.410
2.397
-0.000

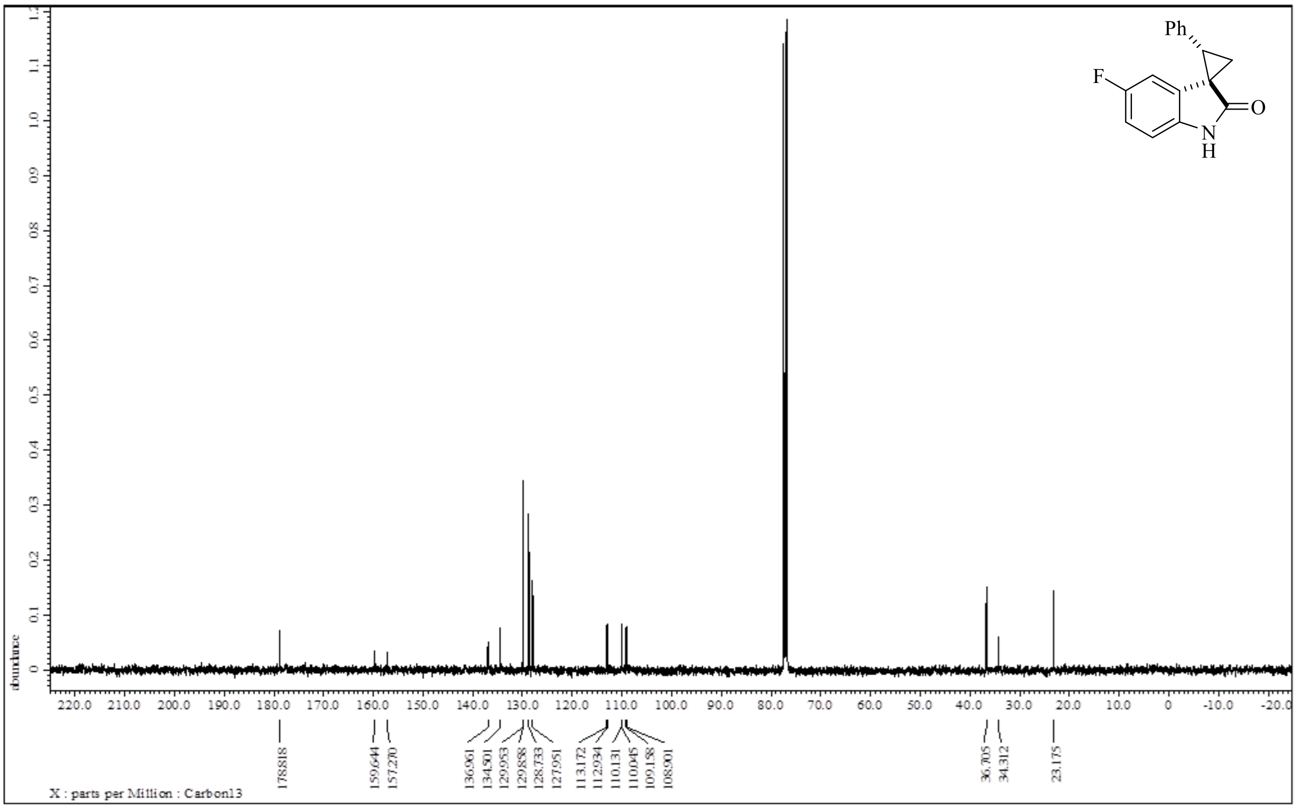
Ph
F
O
N
H
X : parts per Million : Carbon13
178.818
159.644
157.270
136.961
134.501
129.953
129.858
128.733
127.951
113.172
112.934
110.131
110.045
109.158
108.901
36.705
34.312
23.175

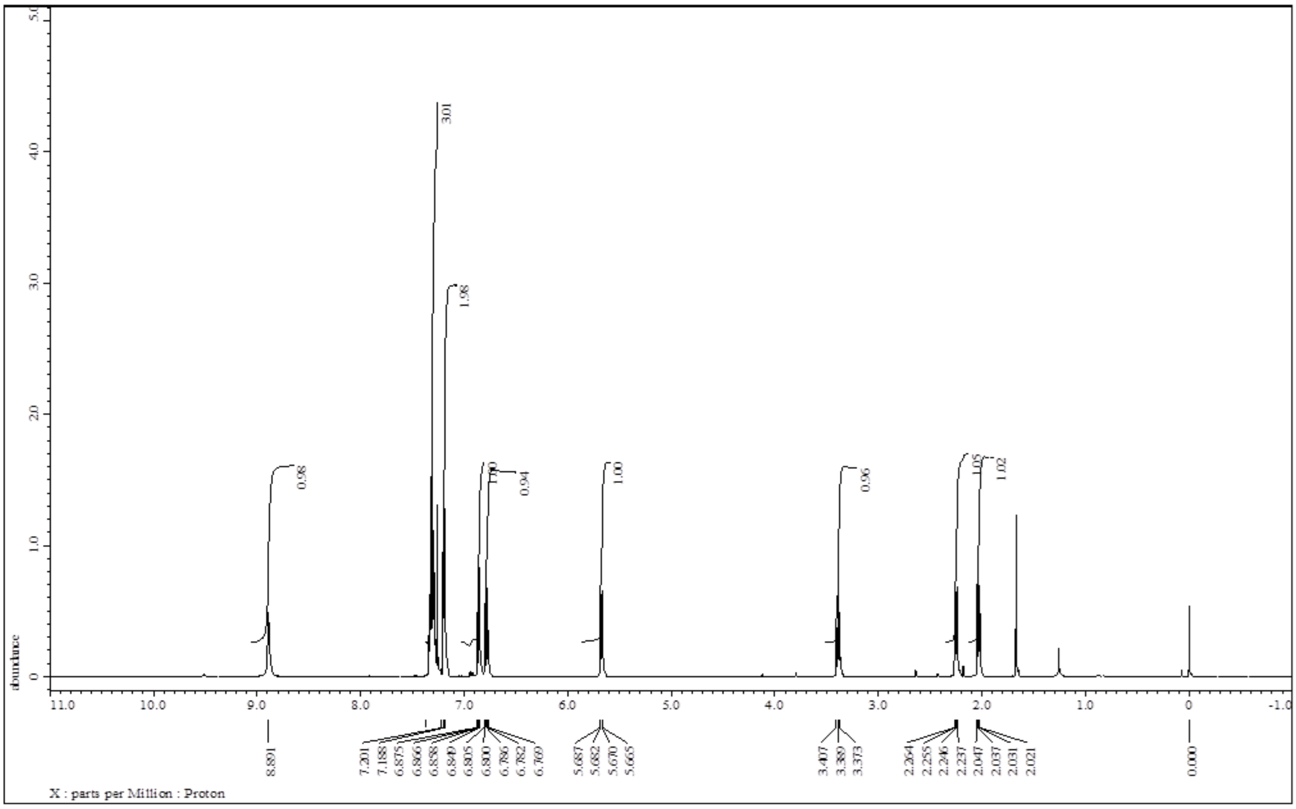
X : parts per Million : Proton
8.891
0.98
3.01
1.98
0.94
1.00
0.96
1.02
5.687
5.682
5.670
5.665
3.407
3.389
3.373
2.264
2.255
2.246
2.237
2.047
2.037
2.031
2.021
0.000

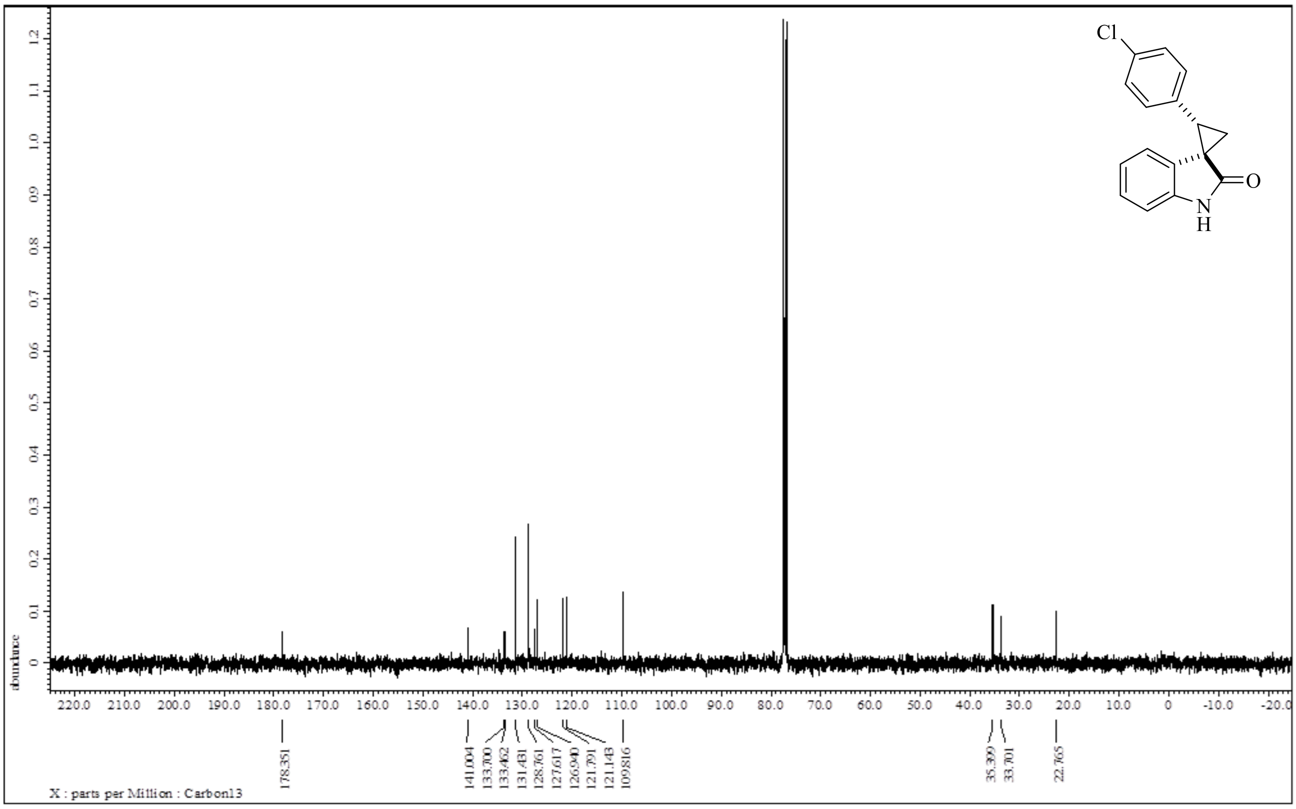
Cl
O
N
H
abundance
X : parts per Million : Carbon13
178.351
141.004
133.700
133.462
131.431
128.761
127.617
126.940
121.791
121.143
109.816
35.399
33.701
22.765

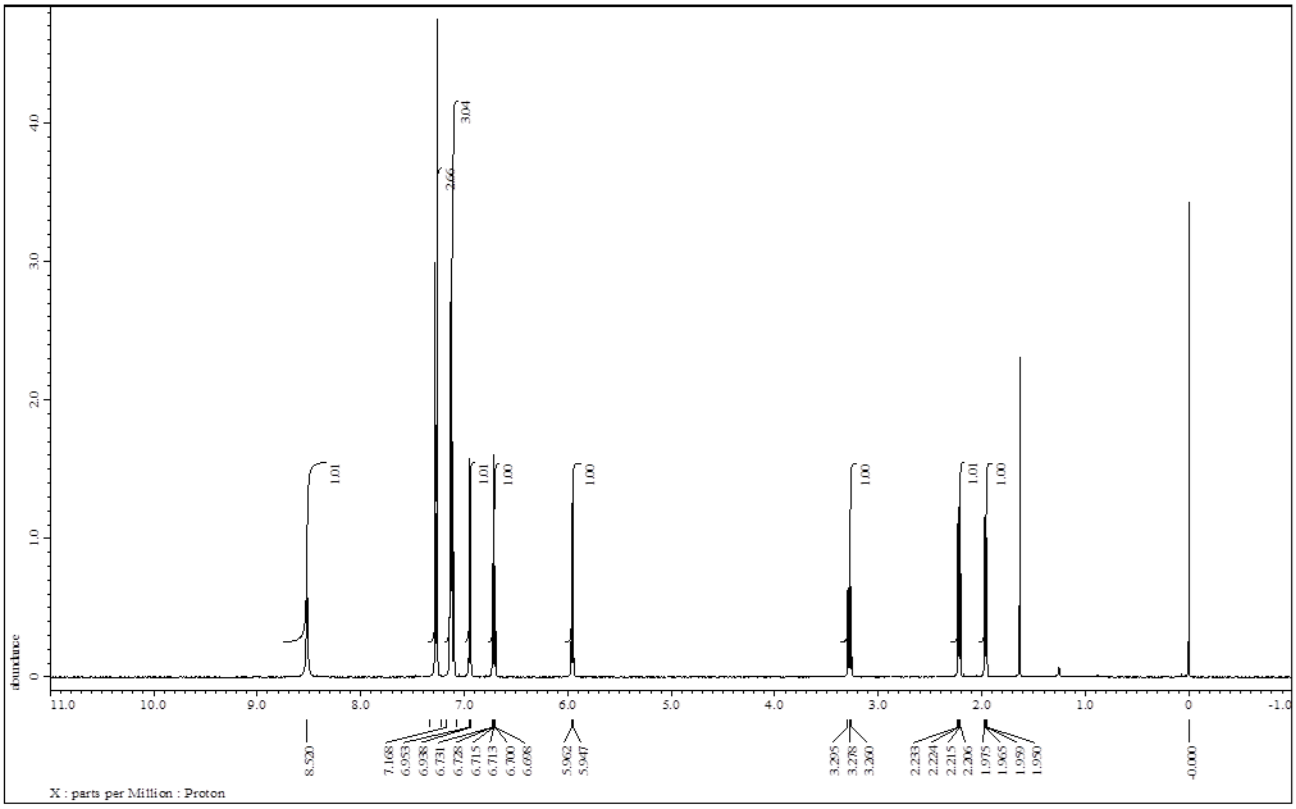
abundance
X : parts per Million : Proton
1.01
2.66
3.04
1.01
1.00
1.00
1.00
1.01
1.00
8.520
7.168
6.953
6.938
6.731
6.728
6.715
6.713
6.700
6.698
5.962
5.947
3.295
3.278
3.260
2.233
2.224
2.215
2.206
1.975
1.965
1.959
1.950
-0.000

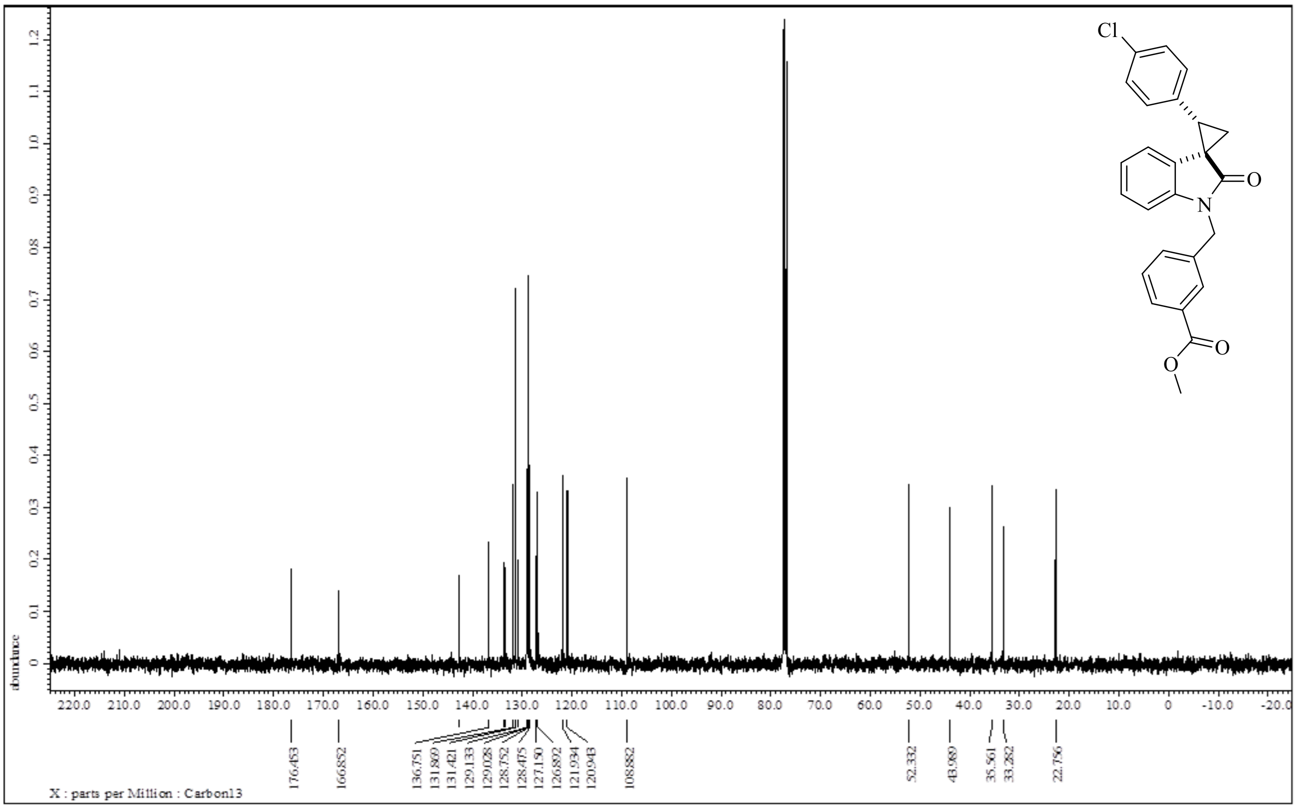
Cl
N
O
O
O
176.453
166.852
136.751
131.869
131.421
129.133
129.026
128.752
128.475
127.150
126.892
121.934
120.943
108.882
52.332
43.989
35.561
33.282
22.756
abundance
X : parts per Million : Carbon13

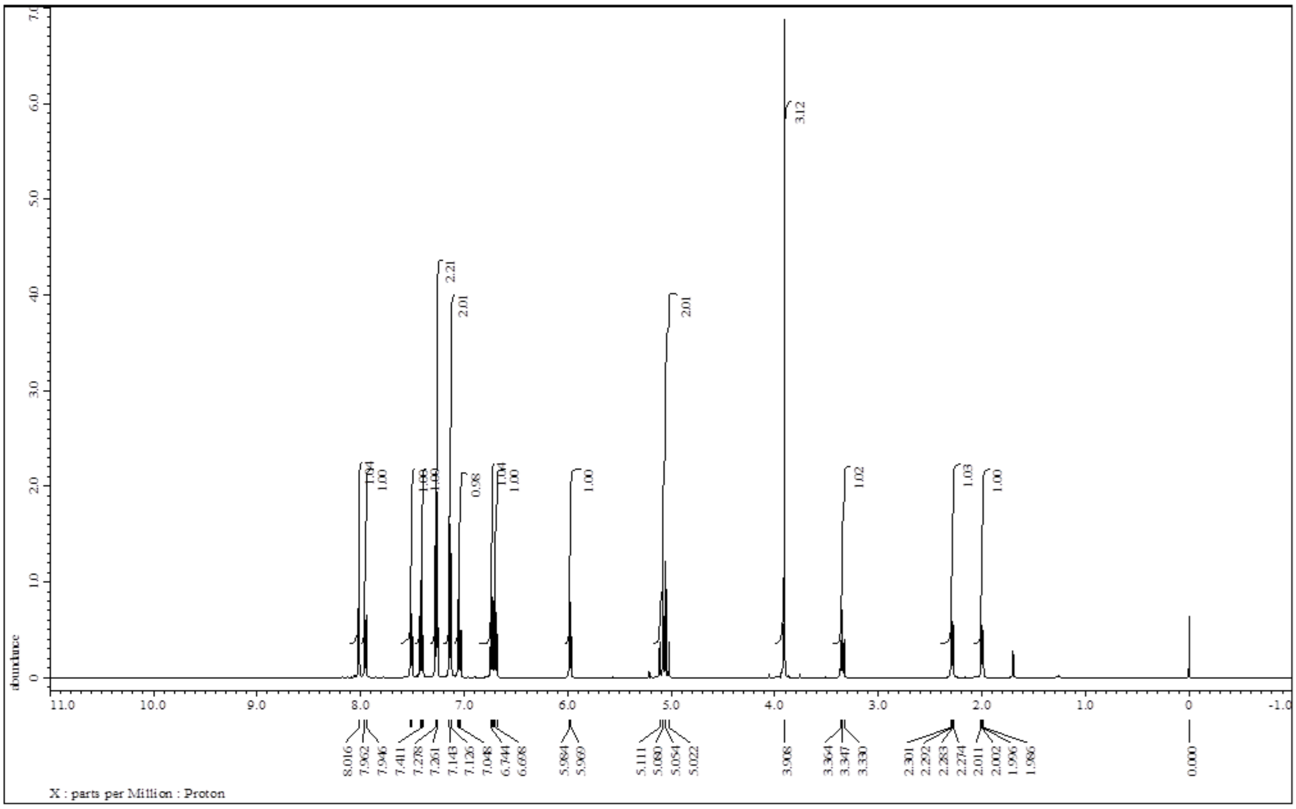
3.12
2.21
2.01
2.01
1.02
1.03
1.00
1.00
8.016
7.962
7.946
7.411
7.278
7.261
7.143
7.126
7.048
6.744
6.698
5.984
5.969
5.111
5.080
5.054
5.022
3.908
3.364
3.347
3.330
2.301
2.292
2.283
2.274
2.011
2.002
1.996
1.986
0.000
abundance
X : parts per Million : Proton

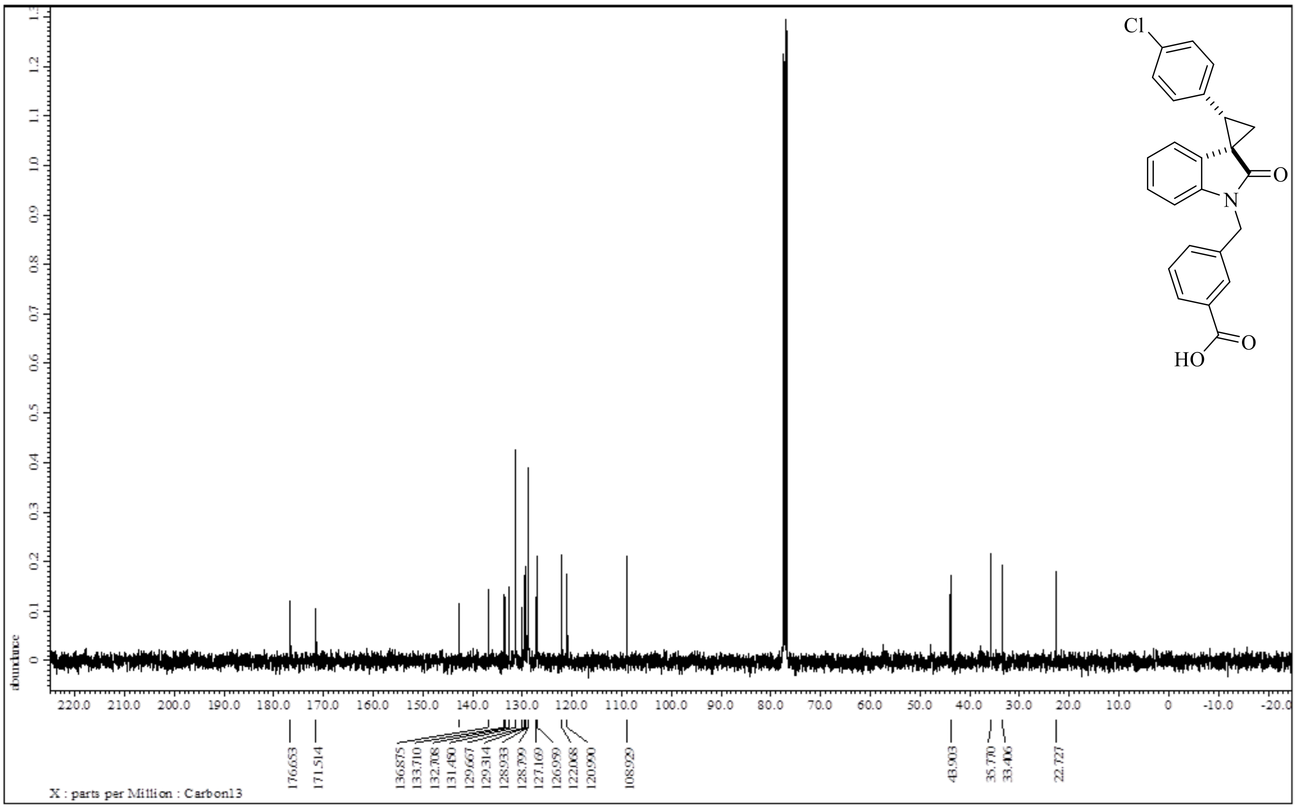
Cl
O
N
HO
O
abundance
176.653
171.514
136.875
133.710
132.708
131.450
129.667
129.314
128.933
128.799
127.169
126.099
122.068
120.990
108.929
43.903
35.770
33.406
22.727
X : parts per Million : Carbon13

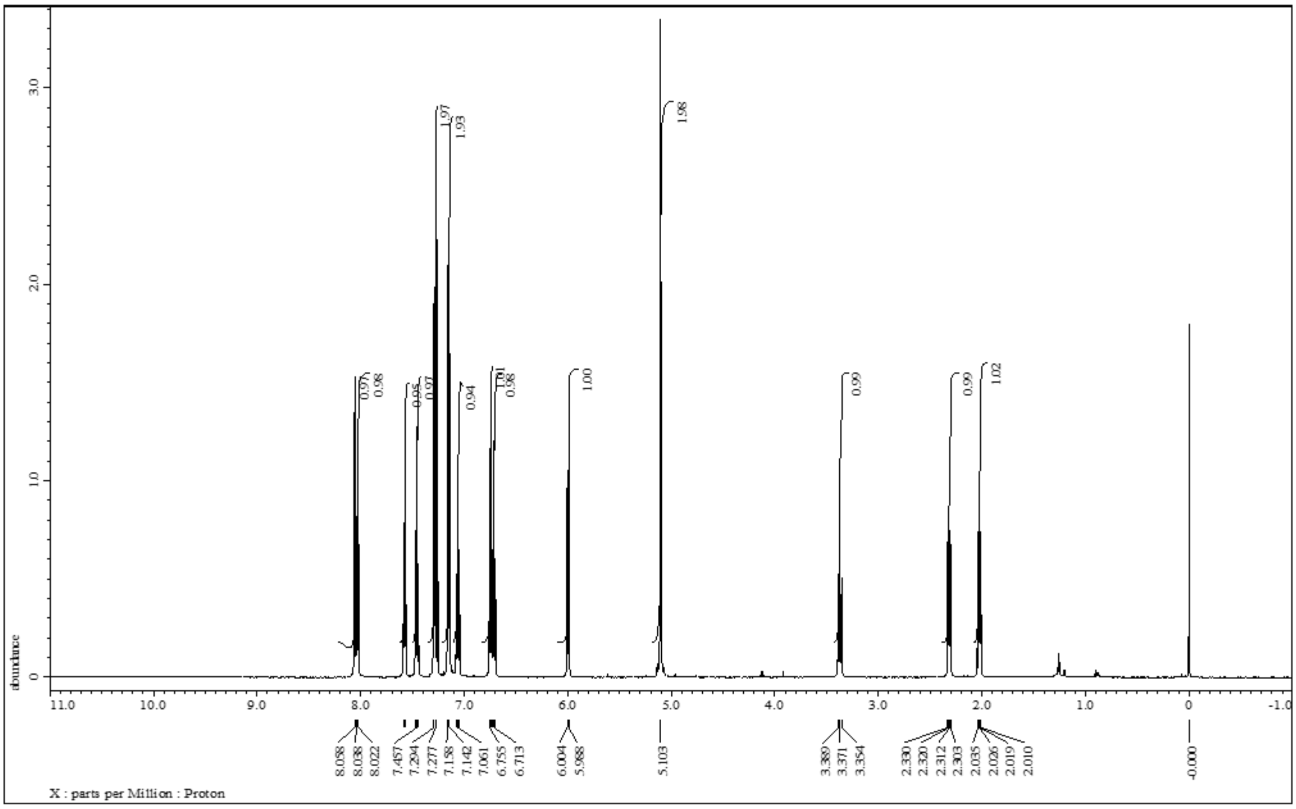
abundance
0.97
0.98
0.95
0.97
1.97
1.93
0.94
1.01
0.98
1.00
1.98
0.99
0.99
1.02
8.058
8.038
8.022
7.457
7.294
7.277
7.158
7.142
7.061
6.755
6.713
6.004
5.988
5.103
3.389
3.371
3.354
2.330
2.320
2.312
2.303
2.035
2.026
2.019
2.010
-0.000
X : parts per Million : Proton

7. HPLC Spectral Data

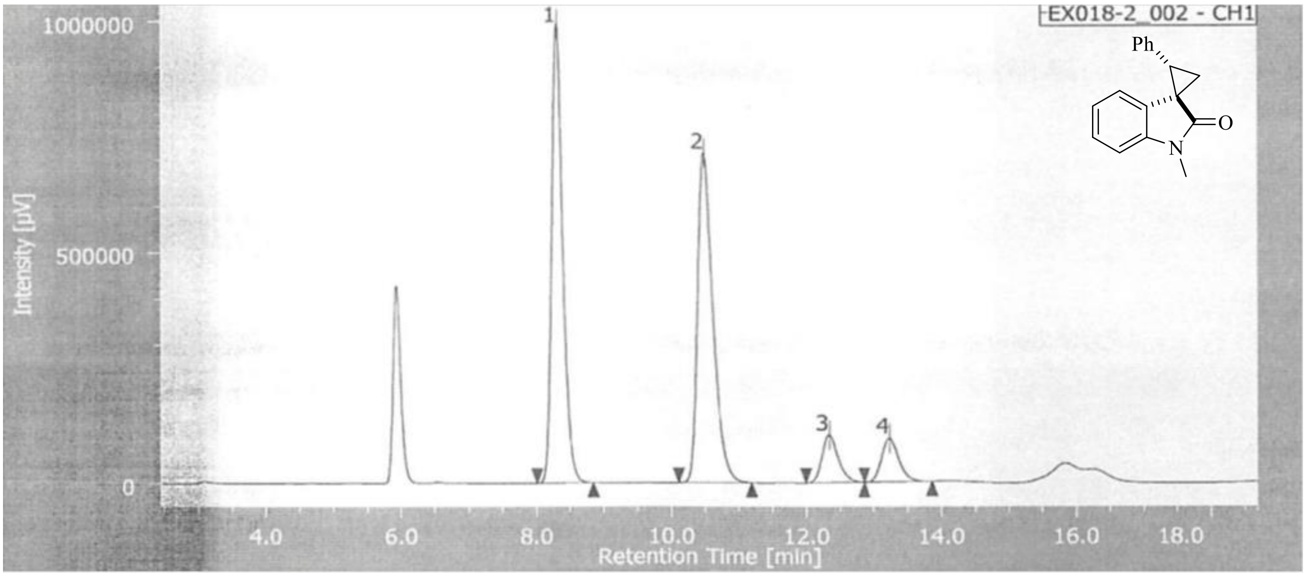

Peak	RT [min]	AREA [µV･sec]	HEIGHT [µV]	AREA %	HEIGHT %
1	8.308	11338703	992411	43.62	52.194
2	10.483	11319166	711437	43.545	37.417
3	12.333	1705650	103273	6.562	5.431
4	13.233	1630551	94257	6.273	4.957

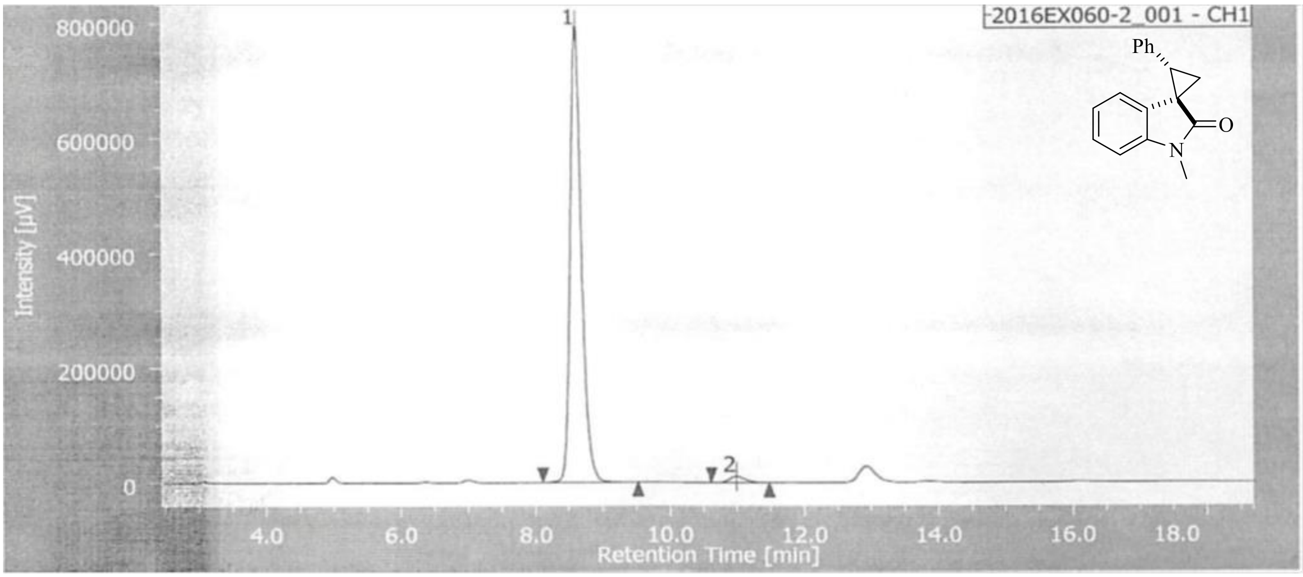

Peak	RT [min]	AREA [µV･sec]	HEIGHT [µV]	AREA %	HEIGHT %
1	8.608	9204295	794574	93.235	95.235
2	10.992	167050	10627	1.693	1.274

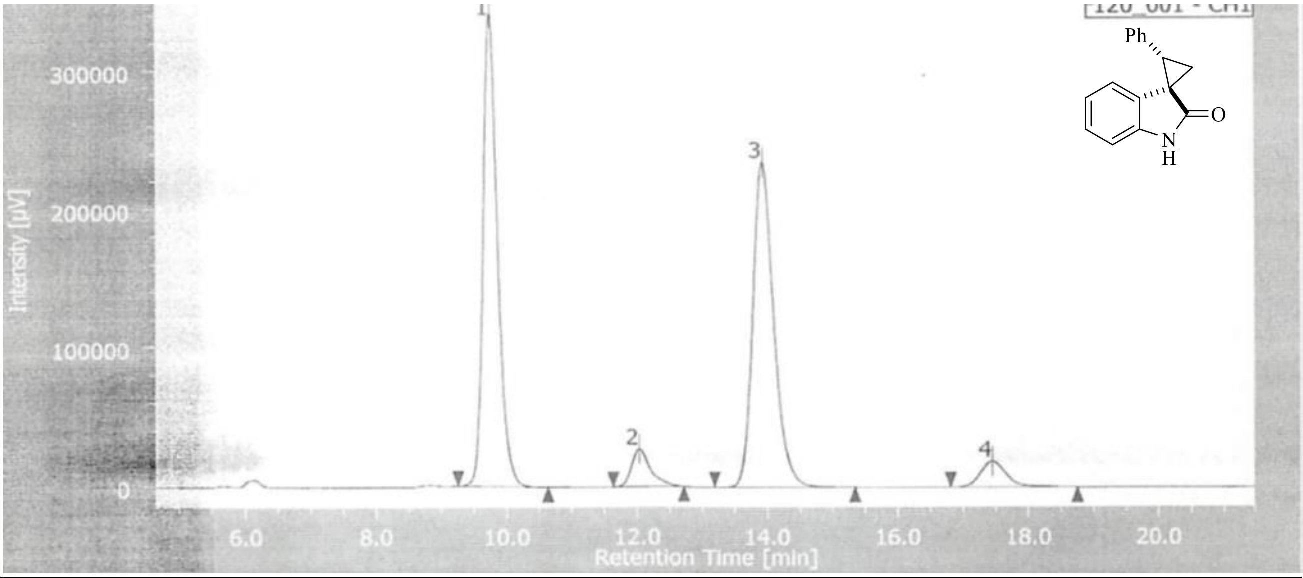

Peak	RT [min]	AREA [µV･sec]	HEIGHT [µV]	AREA %	HEIGHT %
1	9.750	5215730	341203	45.449	54.959
2	12.033	543881	27009	4.739	4.350
3	13.925	5203477	234163	45.342	37.718
4	17.442	512884	18458	4.469	2.973

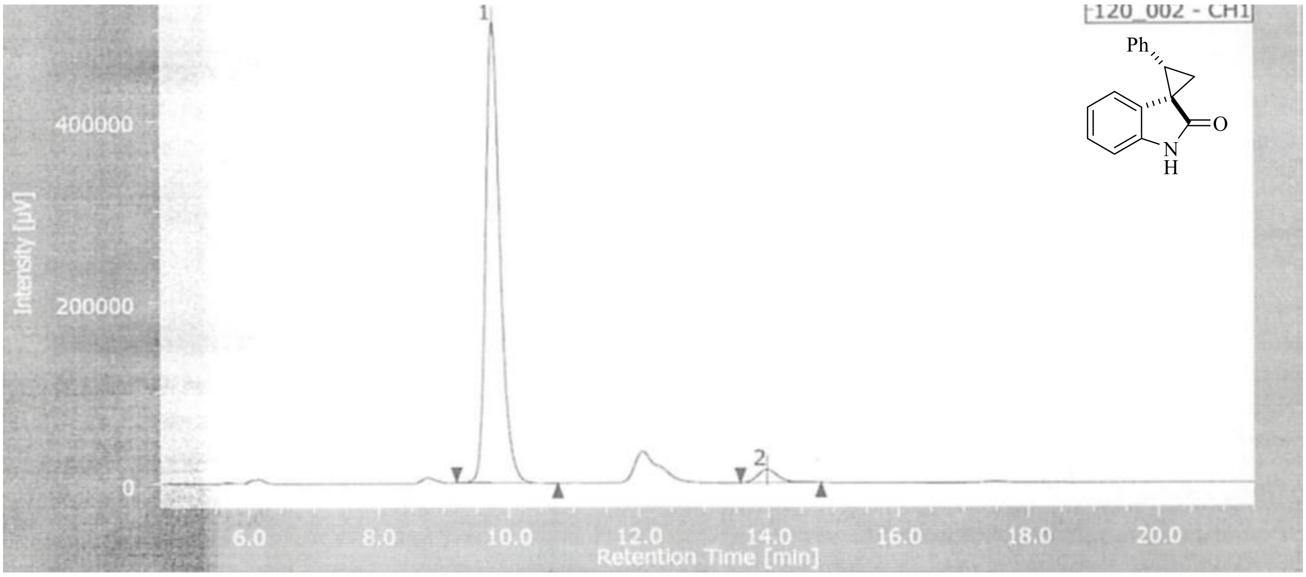

Peak	RT [min]	AREA [µV･sec]	HEIGHT [µV]	AREA %	HEIGHT %
1	9.750	7775113	507841	96.075	97.287
2	13.967	317678	14164	3.925	2.713

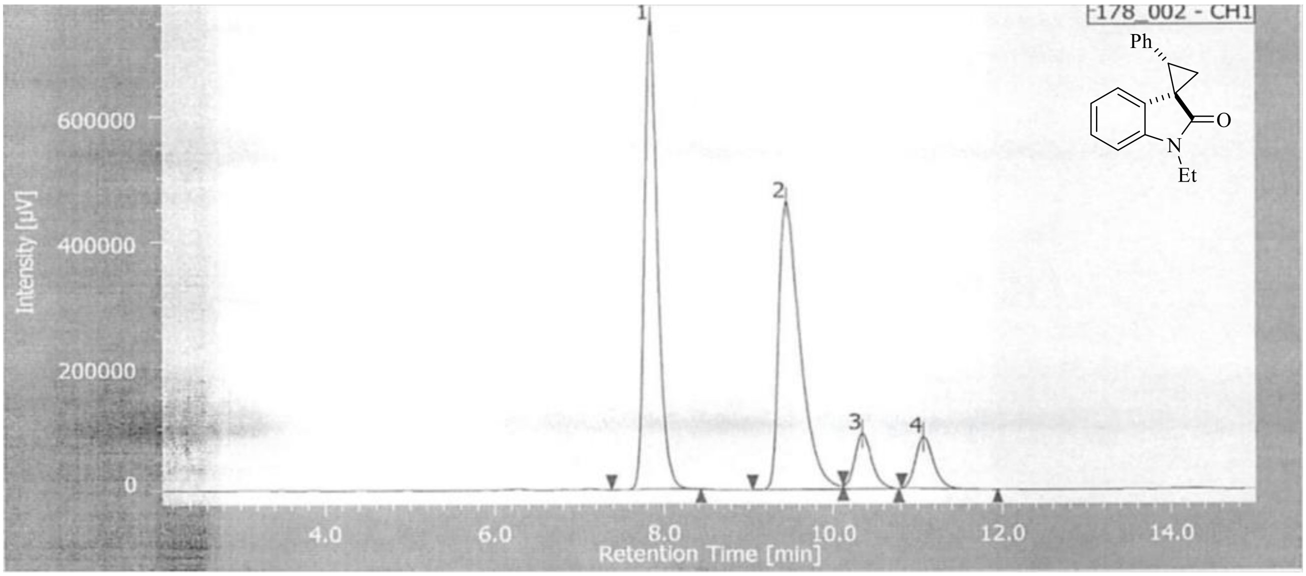

Peak	RT [min]	AREA [μV･sec]	HEIGHT [μV]	AREA %	HEIGHT %
1	7.842	8116125	751033	43.318	54.244
2	9.450	8128680	460897	43.385	33.289
3	10.342	1269959	89399	6.778	6.457
4	11.067	1221413	83212	6.519	6.010

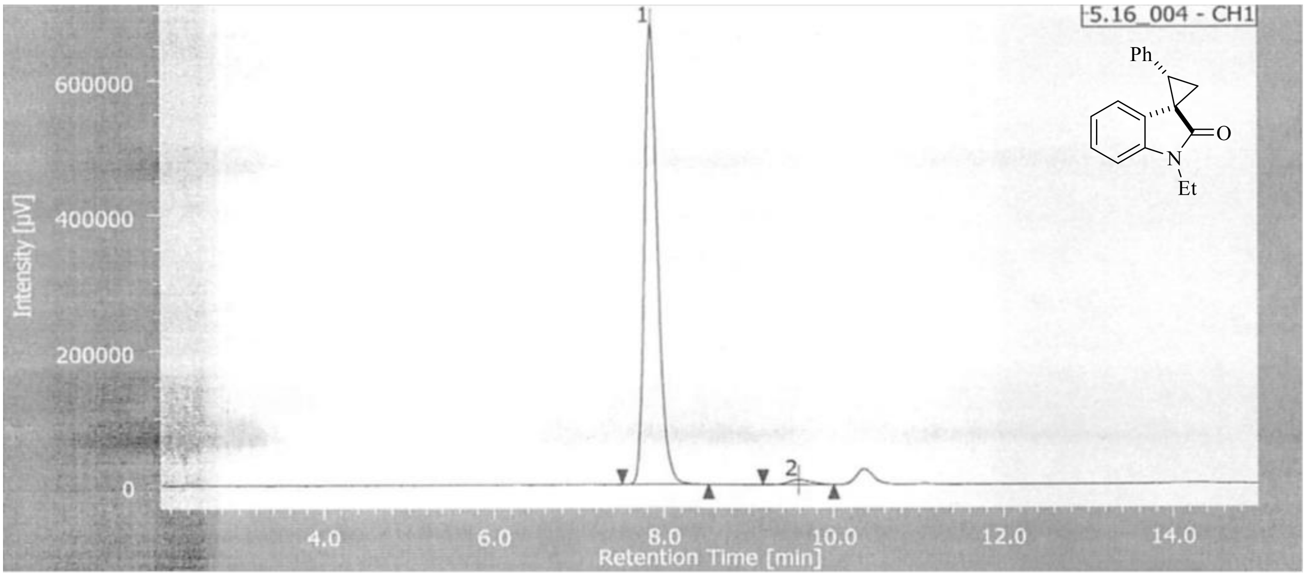

Peak	RT [min]	AREA [μV･sec]	HEIGHT [μV]	AREA %	HEIGHT %
1	7.850	7319986	681193	94.341	95.748
2	9.583	118239	6918	1.524	0.972

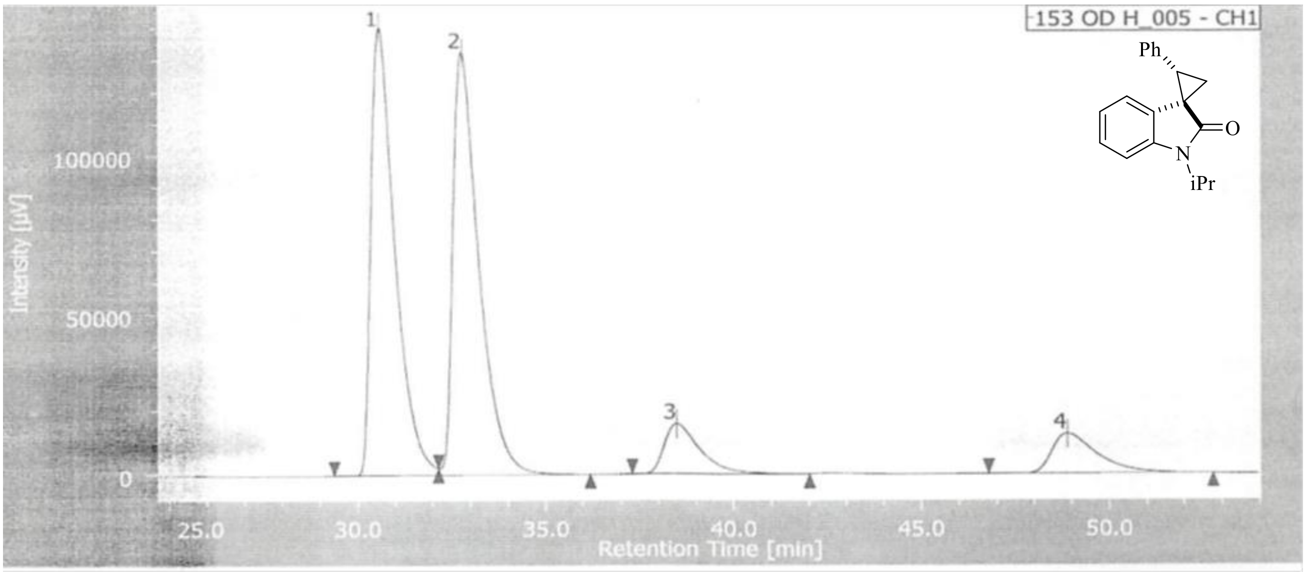

Peak	RT [min]	AREA [µV･sec]	HEIGHT [µV]	AREA %	HEIGHT %
1	30.567	6237086	140459	42.513	46.595
2	32.767	6363770	132628	43.377	43.997
3	38.483	1040495	15801	7.092	5.242
4	48.883	1029500	12559	7.017	4.166

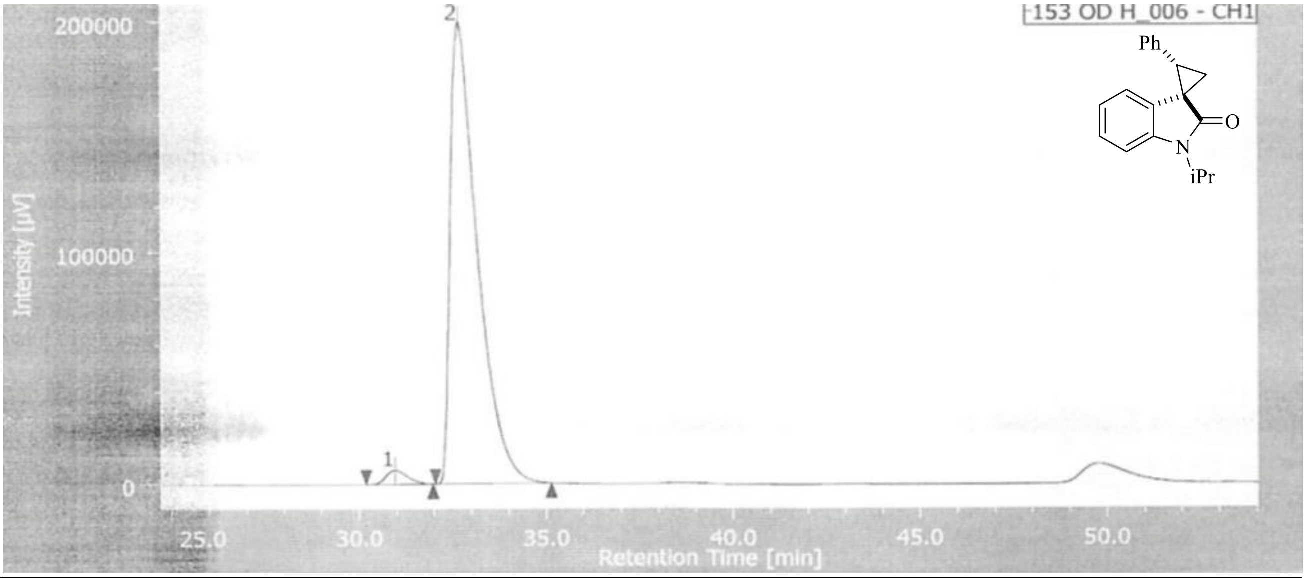

Peak	RT [min]	AREA [µV･sec]	HEIGHT [µV]	AREA %	HEIGHT %
1	30.950	243818	6086	2.367	2.956
2	32.658	10058835	199801	97.633	97.044

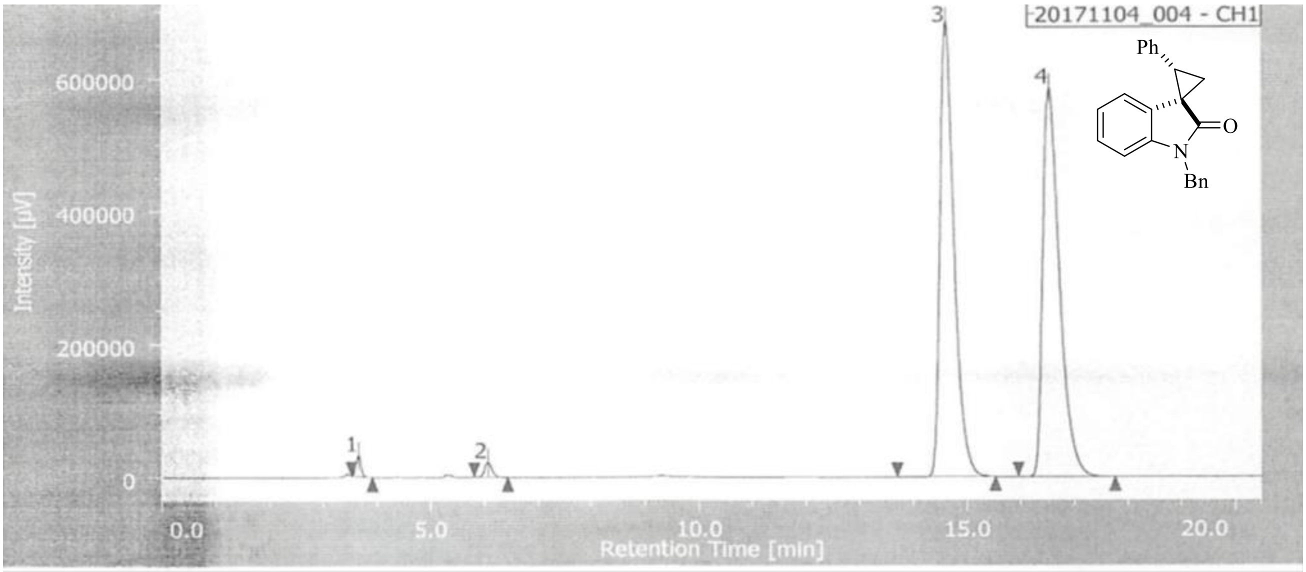

Peak	RT [min]	AREA [µV･sec]	HEIGHT [µV]	AREA %	HEIGHT %
1	3.650	205777	31563	0.814	2.388
2	6.058	205302	22292	0.812	1.686
3	14.600	12438096	683930	49.176	51.737
4	16.533	12443912	584159	49.199	44.189

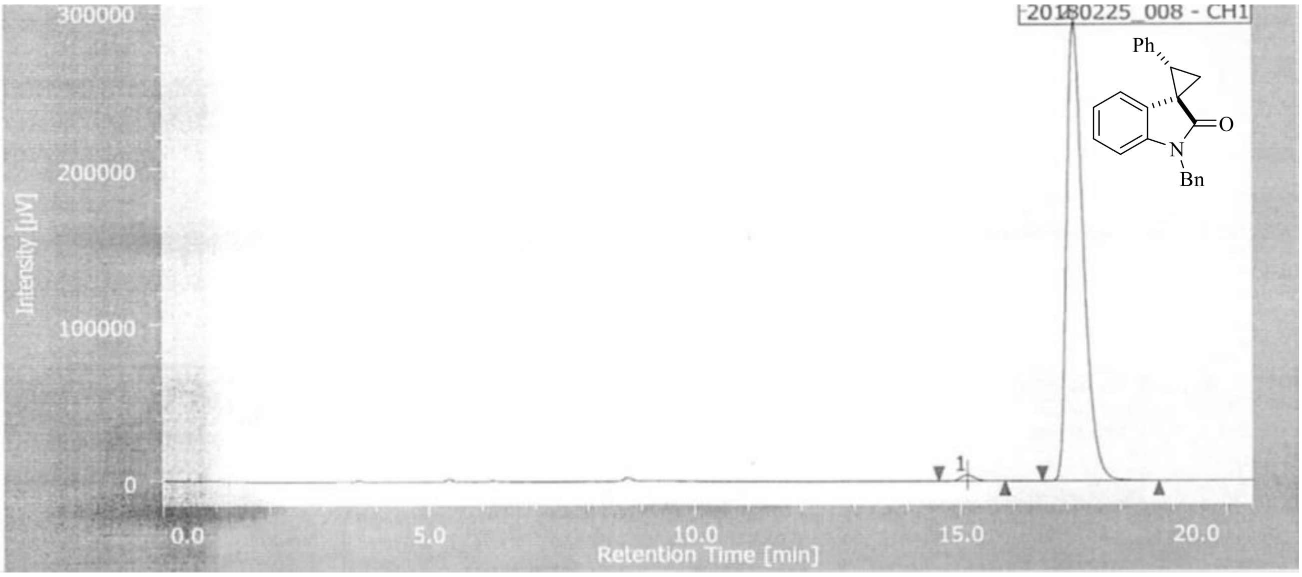

Peak	RT [min]	AREA [µV･sec]	HEIGHT [µV]	AREA %	HEIGHT %
1	15.117	67269	3802	1.044	1.282
2	17.125	6376614	292859	98.956	98.718

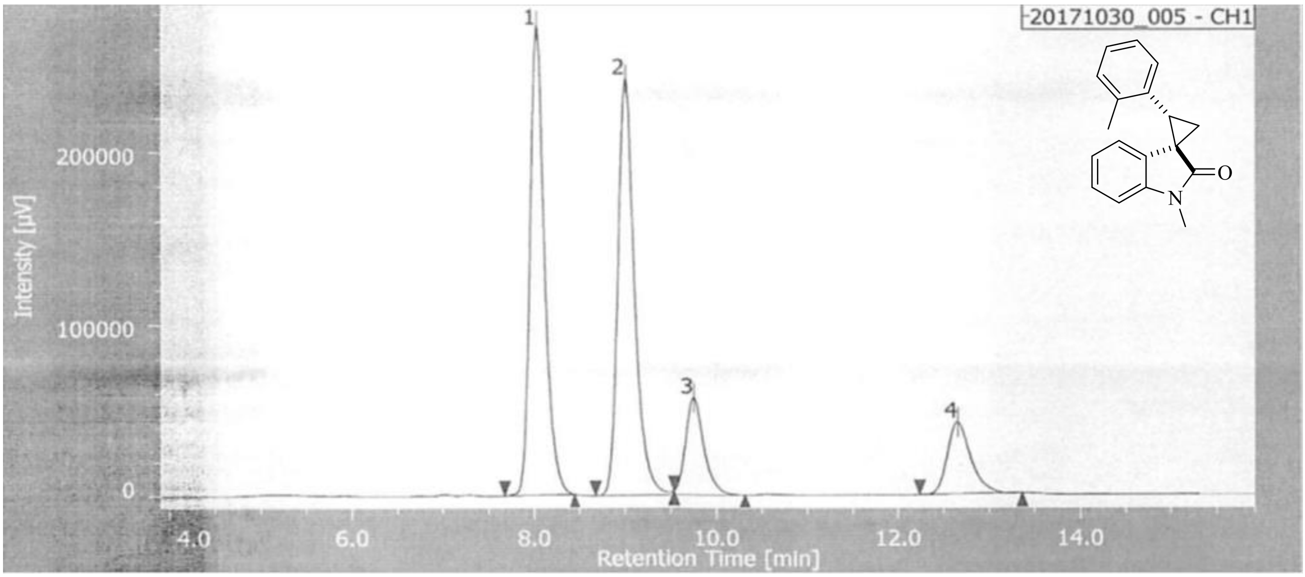

Peak	RT [min]	AREA [μV･sec]	HEIGHT [μV]	AREA %	HEIGHT %
1	8.025	3020645	272768	39.590	44.441
2	9.000	3067864	242102	40.209	39.445
3	9.750	797604	56921	10.454	9.274
4	12.650	743736	41983	9.748	6.840

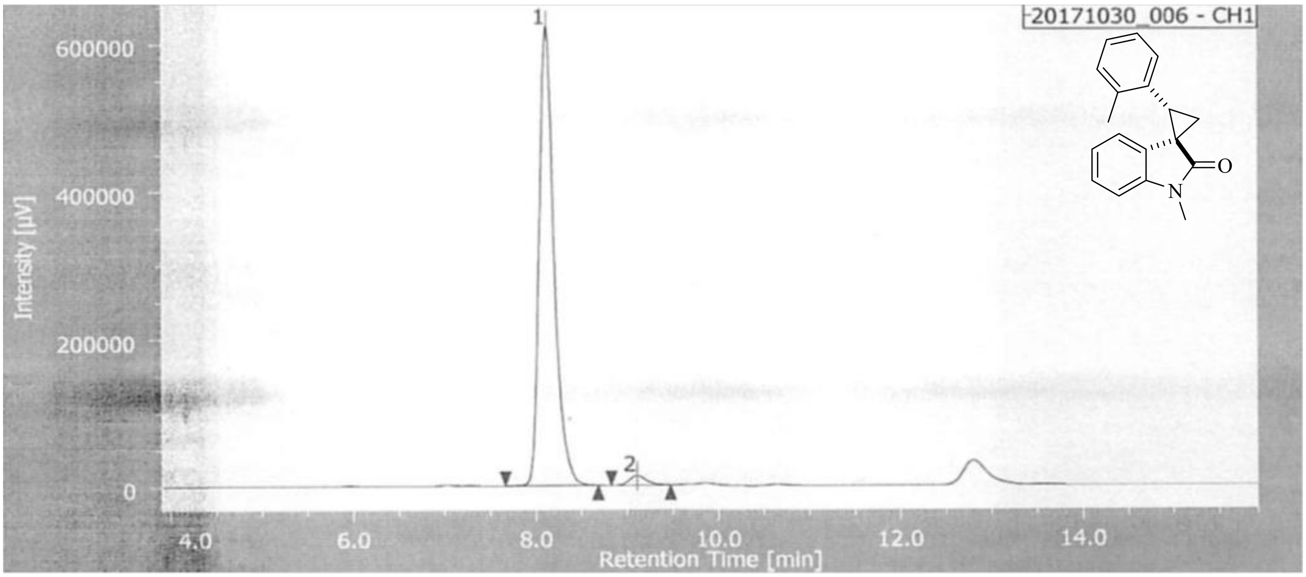

Peak	RT [min]	AREA [μV･sec]	HEIGHT [μV]	AREA %	HEIGHT %
1	8.117	7057092	623612	89.547	92.609
2	9.108	163362	13064	2.073	1.940

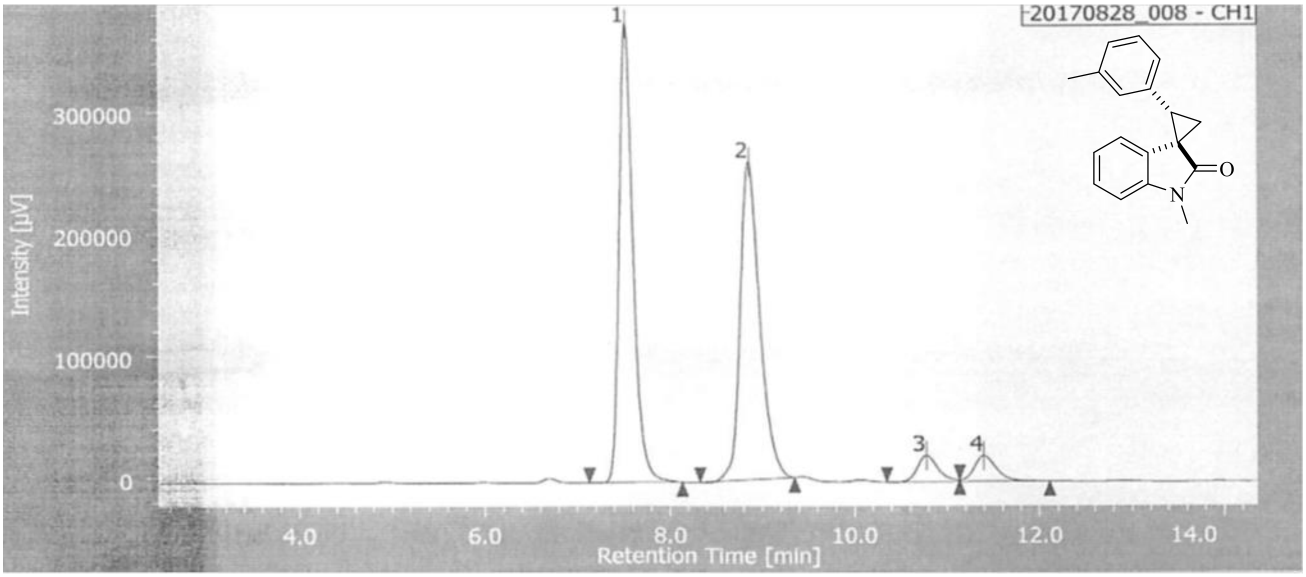

Peak	RT [min]	AREA [µV･sec]	HEIGHT [µV]	AREA %	HEIGHT %
1	7.525	3910023	375183	46.736	55.390
2	8.858	3770110	259248	45.064	38.274
3	10.775	330031	21649	3.945	3.196
4	11.392	355944	21274	4.255	3.141

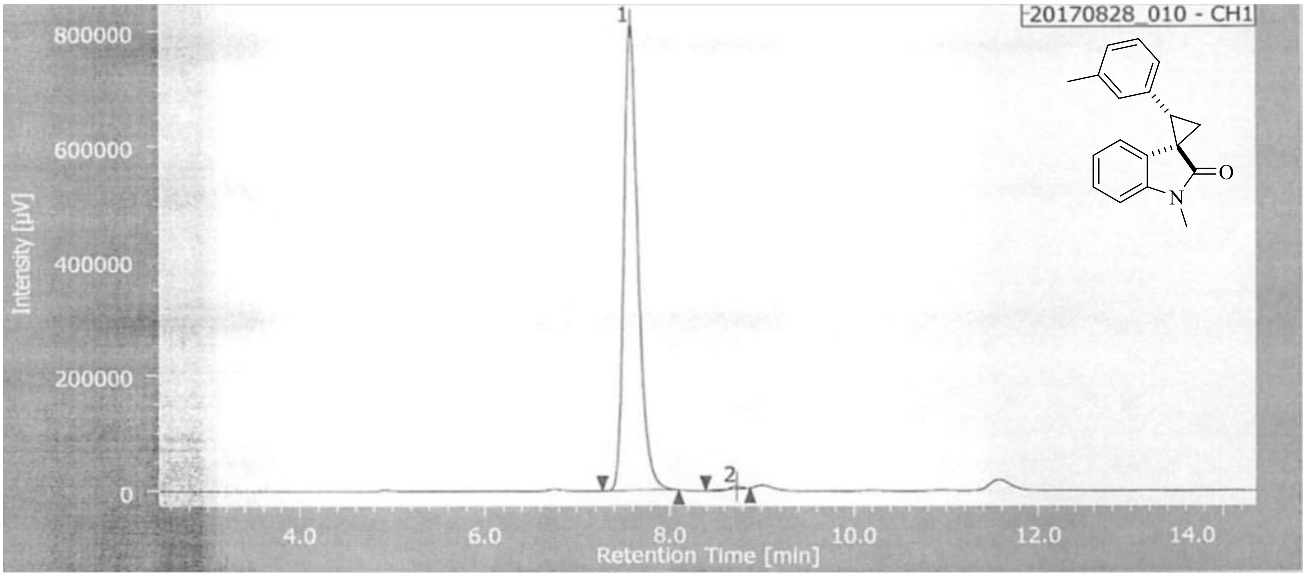

Peak	RT [min]	AREA [µV･sec]	HEIGHT [µV]	AREA %	HEIGHT %
1	7.575	8604621	810746	95.826	96.972
2	8.733	34495	4628	0.384	0.554

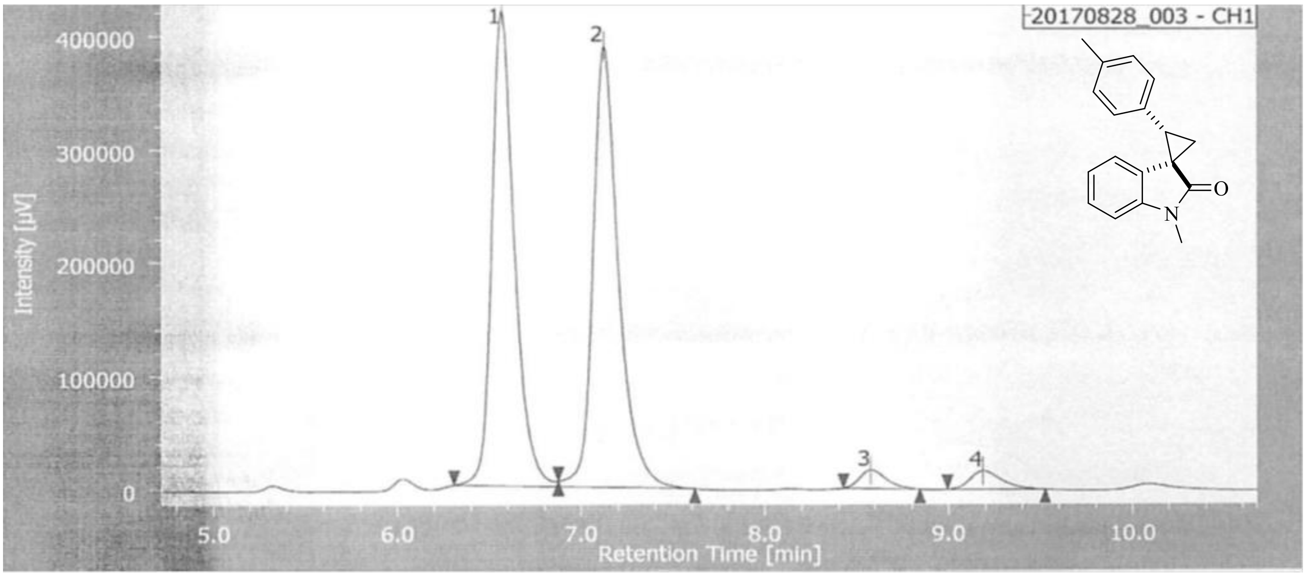

Peak	RT [min]	AREA [µV･sec]	HEIGHT [µV]	AREA %	HEIGHT %
1	6.567	3589171	419606	46.732	49.799
2	7.125	3731985	390247	48.591	46.314
3	8.575	166788	16507	2.172	1.959
4	9.183	192413	16243	2.505	1.928

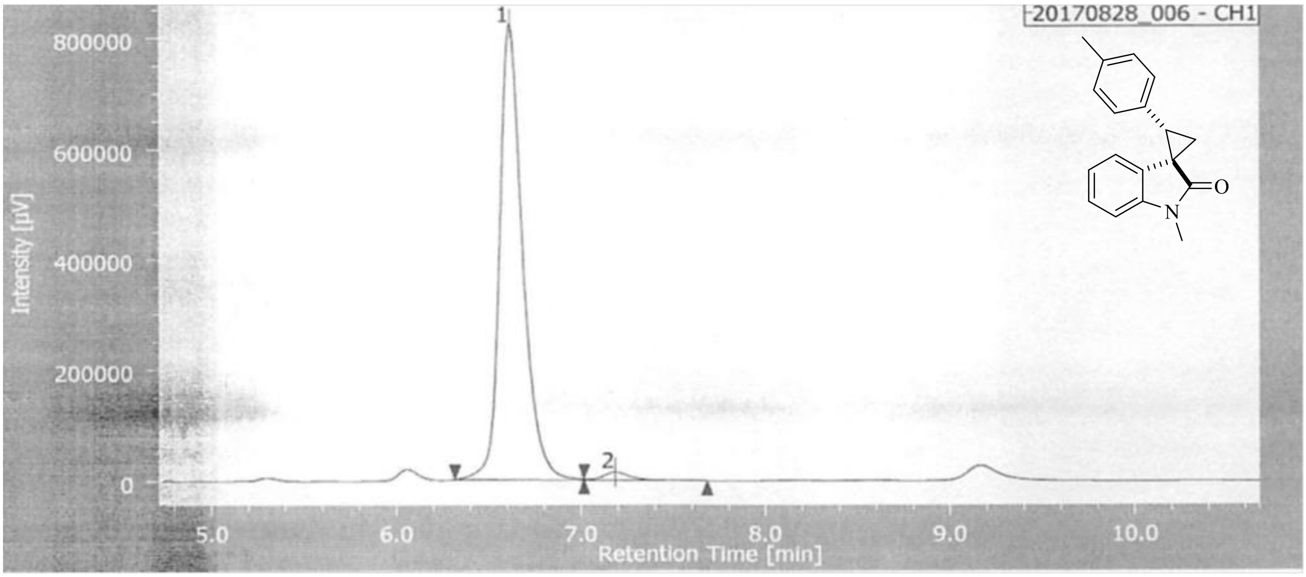

Peak	RT [min]	AREA [µV･sec]	HEIGHT [µV]	AREA %	HEIGHT %
1	6.617	7129752	821850	93.740	95.036
2	7.183	140281	14501	1.844	1.677

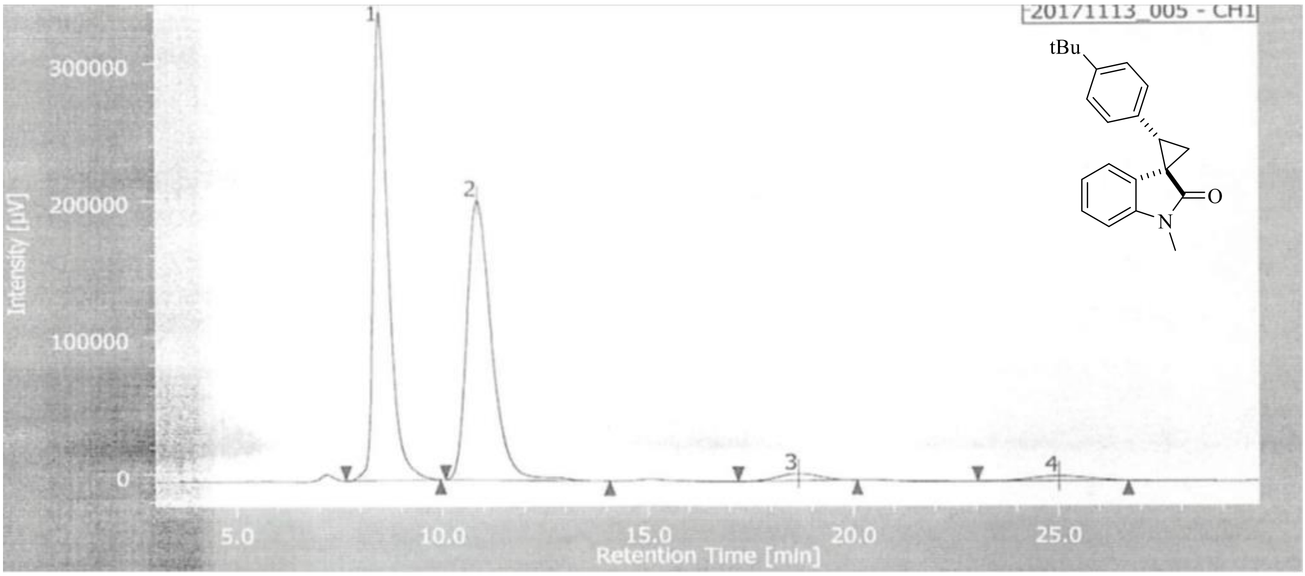

Peak	RT [min]	AREA [μV･sec]	HEIGHT [μV]	AREA %	HEIGHT %
1	8.475	8522084	341384	48.048	61.632
2	10.858	849572	203619	47.900	36.760
3	18.658	377168	5356	2.127	0.967
4	25.000	341482	3552	1.925	0.641

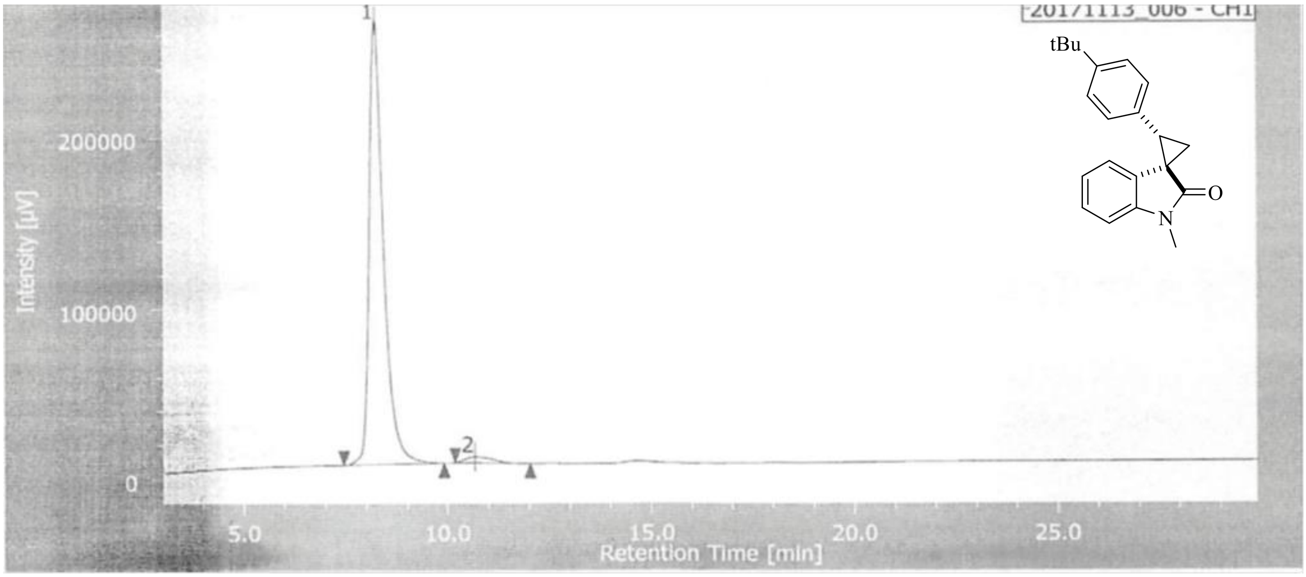

Peak	RT [min]	AREA [μV･sec]	HEIGHT [μV]	AREA %	HEIGHT %
1	8.225	6383041	259726	97.470	98.623
2	10.667	165707	3626	2.530	1.377

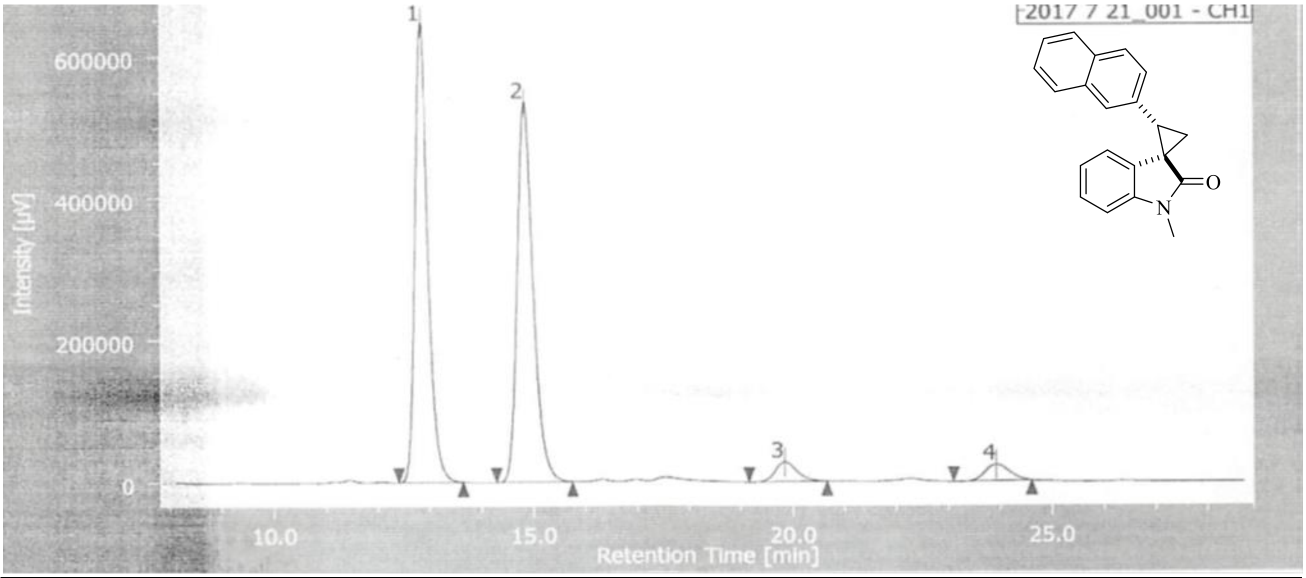

Peak	RT [min]	AREA [µV･sec]	HEIGHT [µV]	AREA %	HEIGHT %
1	12.833	11452879	647524	46.823	52.497
2	14.825	11448280	534828	46.804	43.360
3	19.842	805700	27841	3.294	2.257
4	23.917	752932	23261	3.078	1.886

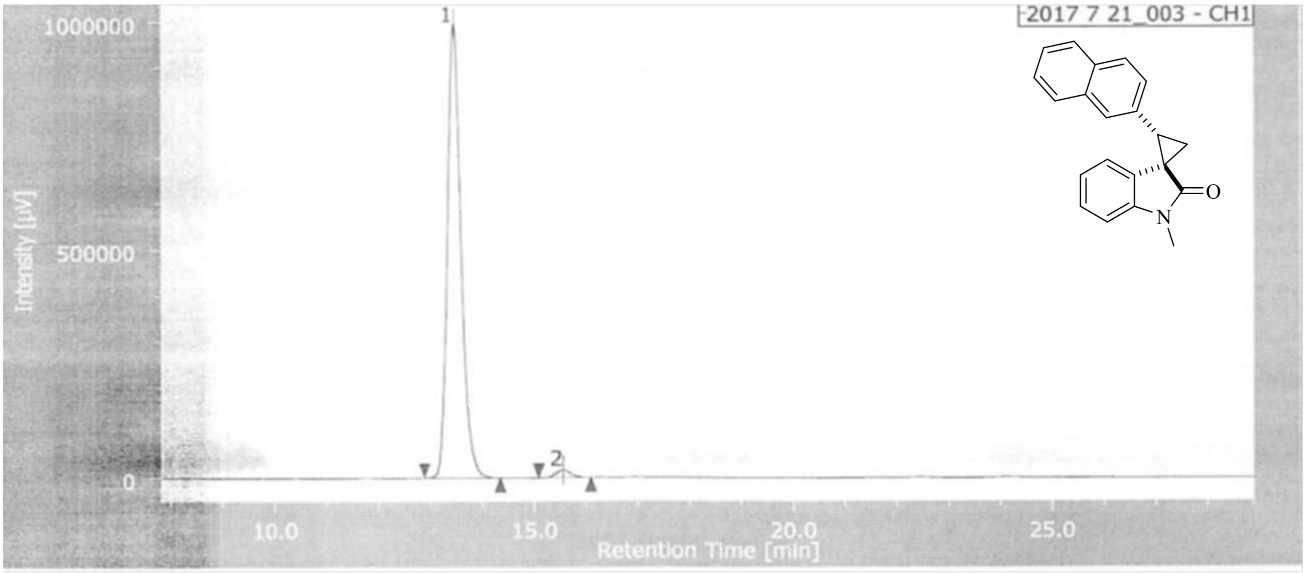

Peak	RT [min]	AREA [µV･sec]	HEIGHT [µV]	AREA %	HEIGHT %
1	13.450	17991952	995908	98.135	98.338
2	15.550	342018	16836	1.865	1.662

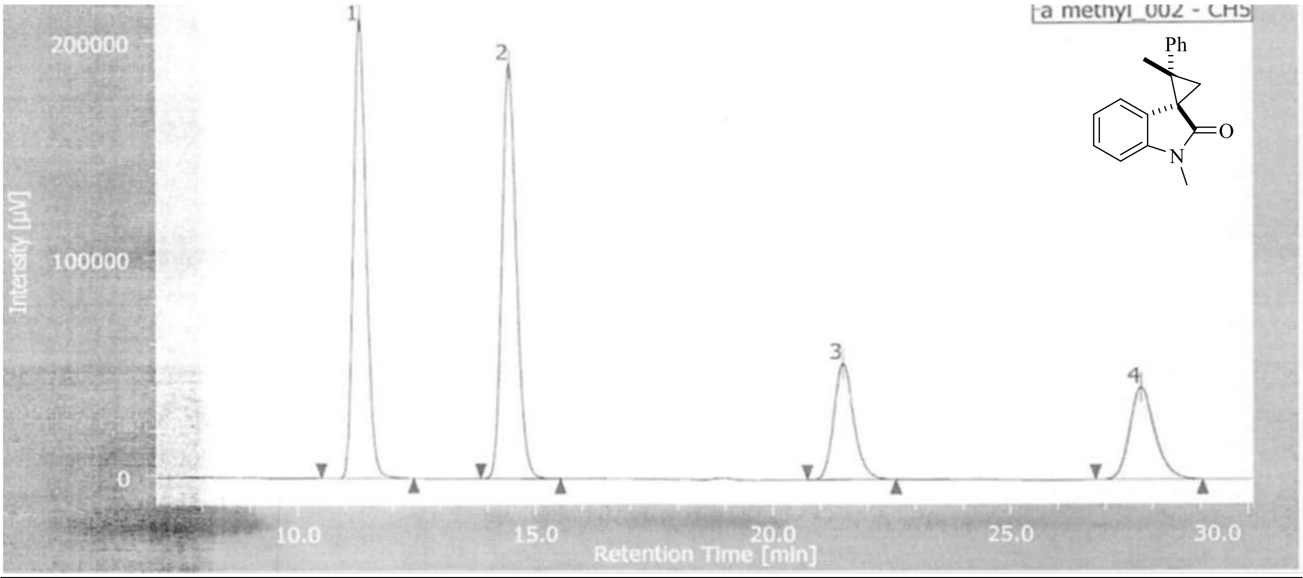

Peak	RT [min]	AREA [µV･sec]	HEIGHT [µV]	AREA %	HEIGHT %
1	11.317	3893136	211736	35.151	42.552
2	14.450	4027366	190421	36.363	38.268
3	21.467	1580955	53320	14.274	10.716
4	27.733	1574081	42120	14.212	8.465

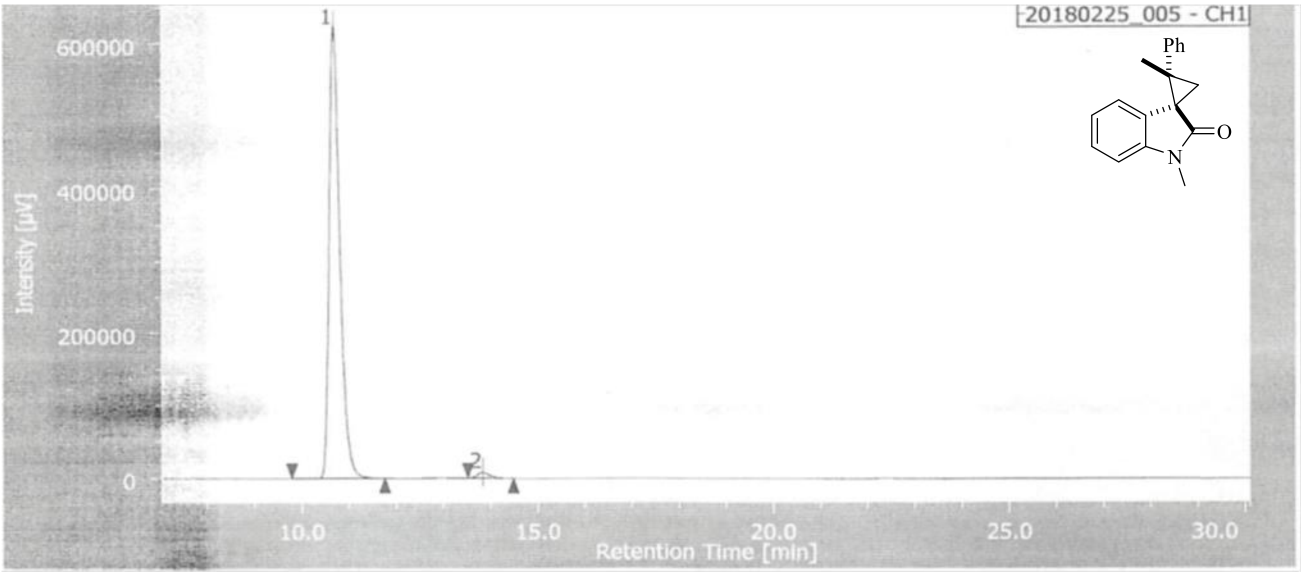

Peak	RT [min]	AREA [µV･sec]	HEIGHT [µV]	AREA %	HEIGHT %
1	10.683	9808470	624031	98.569	98.738
2	13.825	142423	7978	1.431	1.262

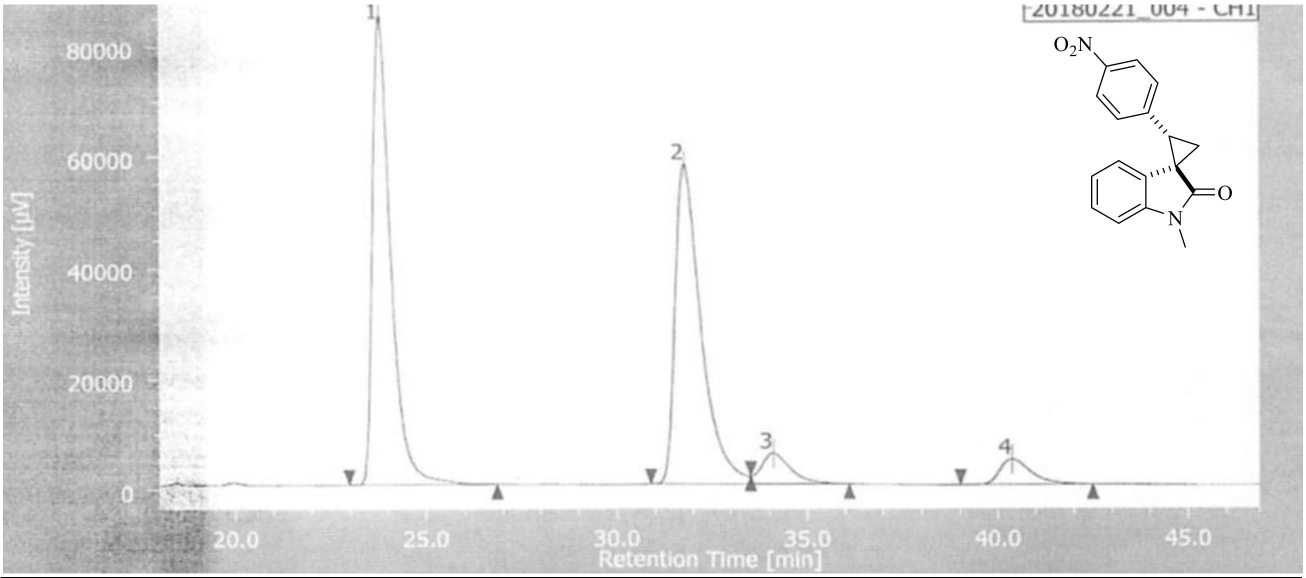

Peak	RT [min]	AREA [μV･sec]	HEIGHT [μV]	AREA %	HEIGHT %
1	23.767	2819214	84551	45.994	55.545
2	31.767	2750635	57482	44.875	37.762
3	34.100	303840	5652	4.957	3.713
4	40.375	255820	4536	4.174	2.980

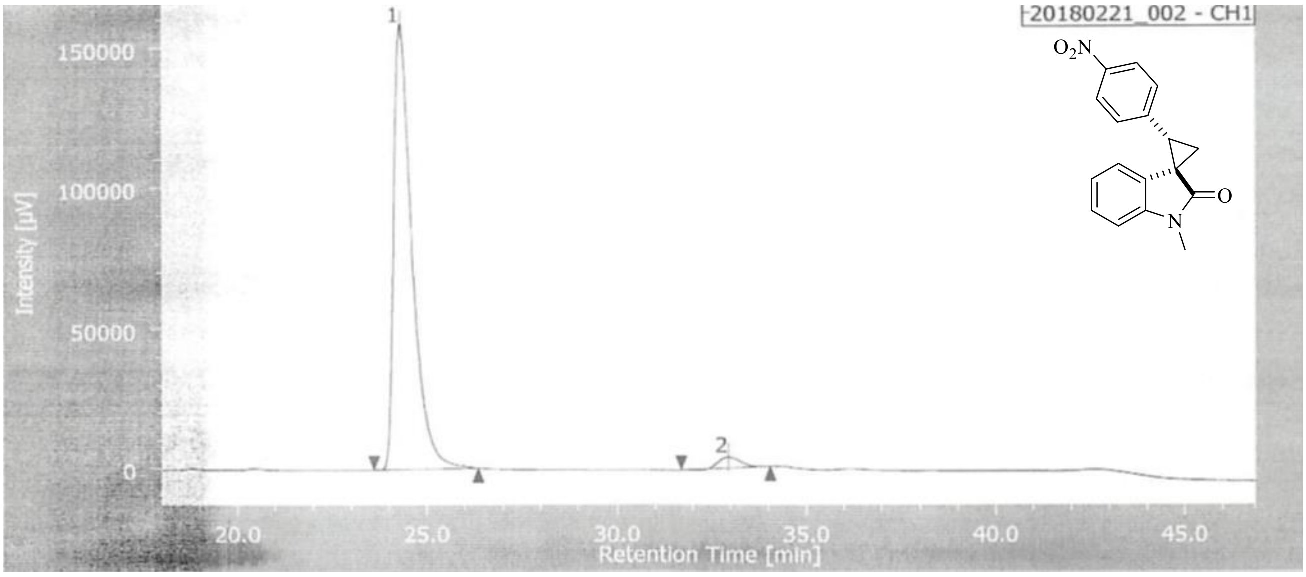

Peak	RT [min]	AREA [μV･sec]	HEIGHT [μV]	AREA %	HEIGHT %
1	24.292	5427210	158810	97.046	97.479
2	32.942	165211	4107	2.954	2.521

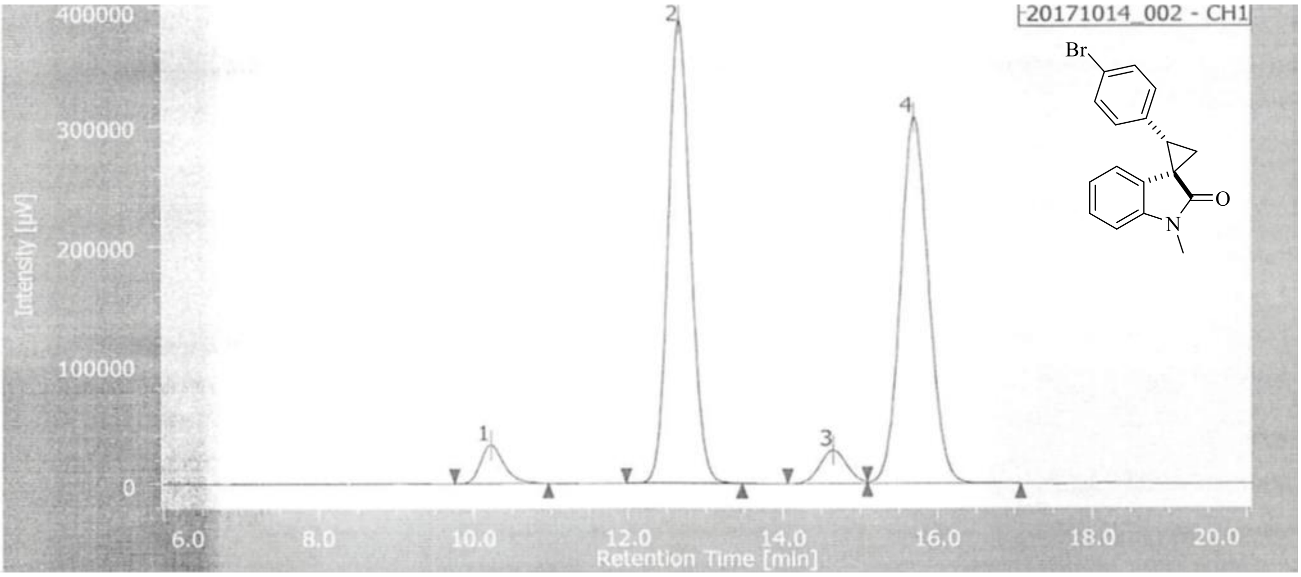

Peak	RT [min]	AREA [µV·sec]	HEIGHT [µV]	AREA %	HEIGHT %
1	10.225	671464	32487	3.953	4.293
2	12.658	7802144	388606	45.935	51.354
3	14.650	685039	28161	4.033	3.721
4	15.700	7826553	307467	46.079	40.631

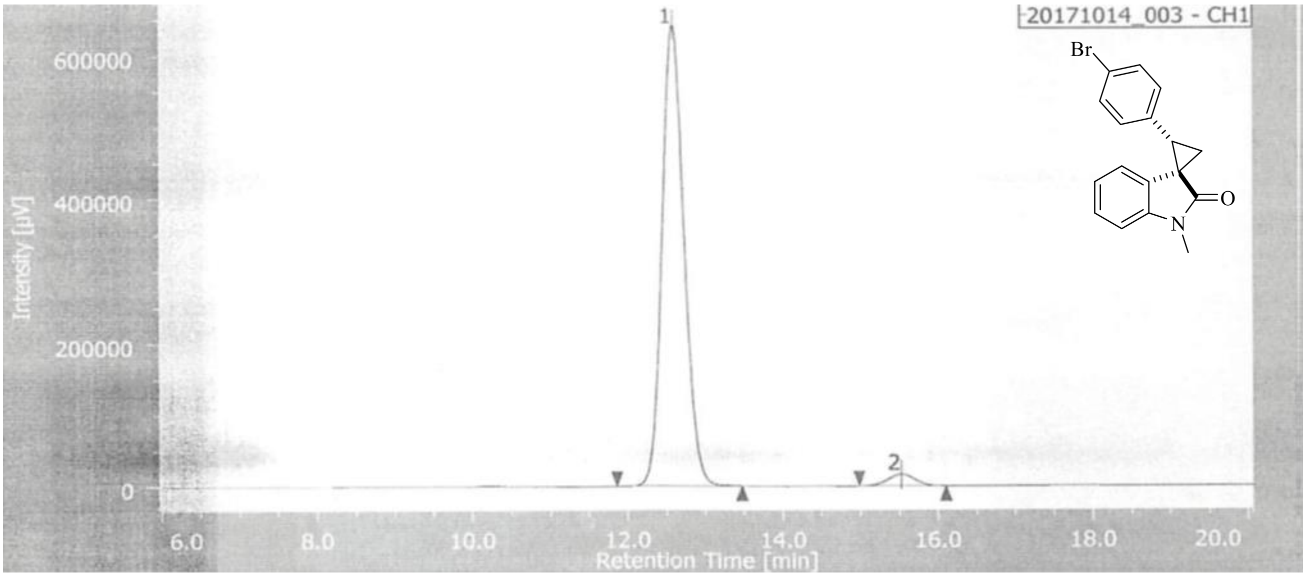

Peak	RT [min]	AREA [µV·sec]	HEIGHT [µV]	AREA %	HEIGHT %
1	12.583	12619787	641908	97.029	97.521
2	15.525	386395	16319	2.971	2.479

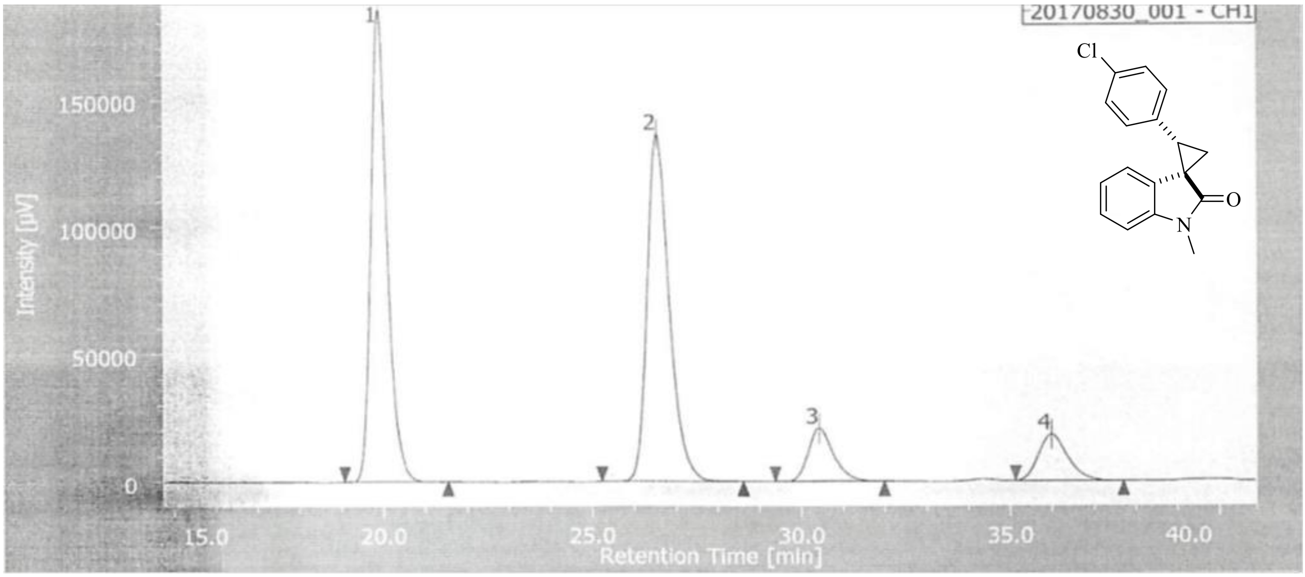

Peak	RT [min]	AREA [µV･sec]	HEIGHT [µV]	AREA %	HEIGHT %
1	19.883	4998774	185273	42.669	51.442
2	26.542	4981822	135932	42.524	37.742
3	30.417	871076	20842	7.435	5.787
4	35.975	863672	18111	7.372	5.029

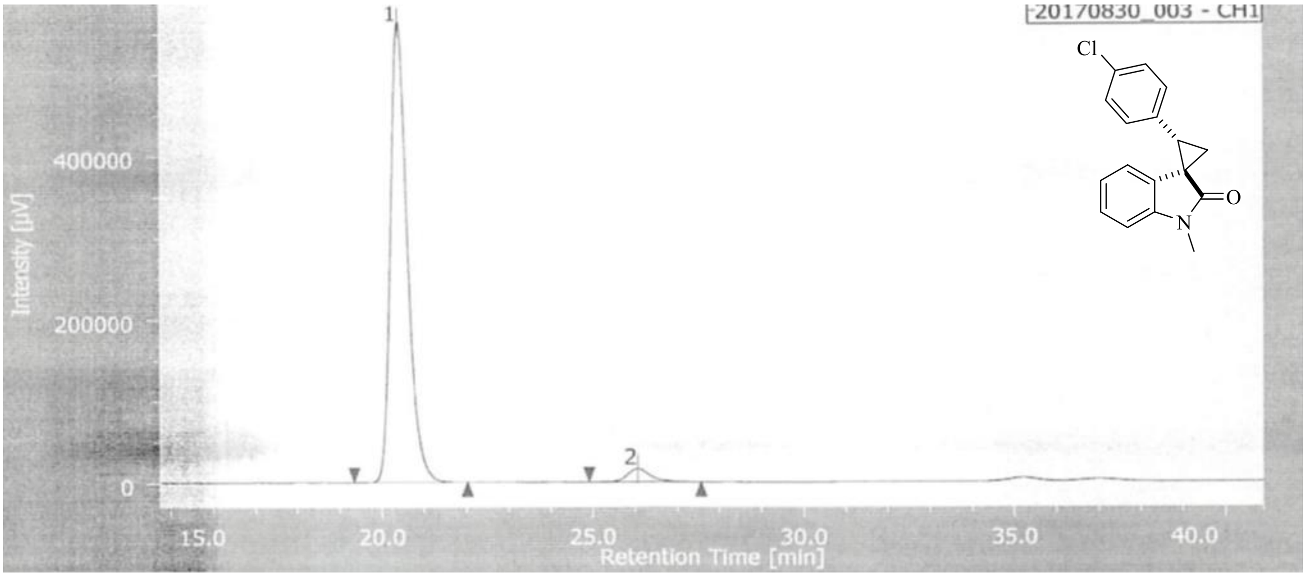

Peak	RT [min]	AREA [µV･sec]	HEIGHT [µV]	AREA %	HEIGHT %
1	20.383	15617327	562187	96.619	97.315
2	26.050	546532	15511	3.381	2.685

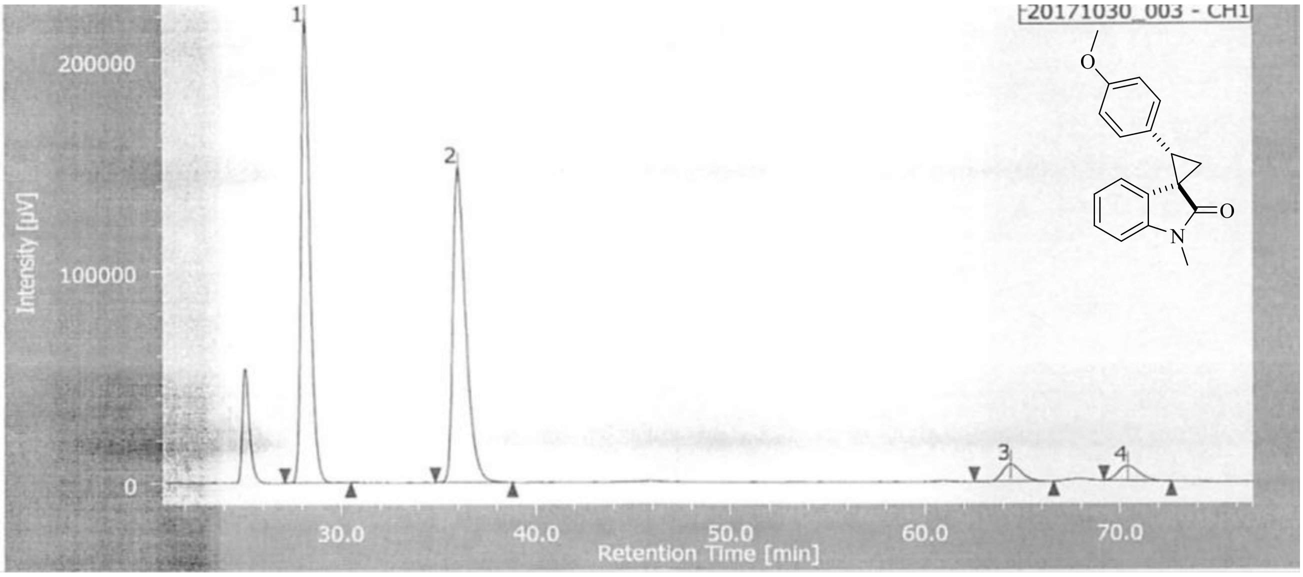

Peak	RT [min]	AREA [µV･sec]	HEIGHT [µV]	AREA %	HEIGHT %
1	28.175	6867927	219109	46.518	57.205
2	36.000	6825369	148841	46.230	38.859
3	64.392	550253	8112	3.727	2.118
4	70.392	520446	6965	3.525	1.818

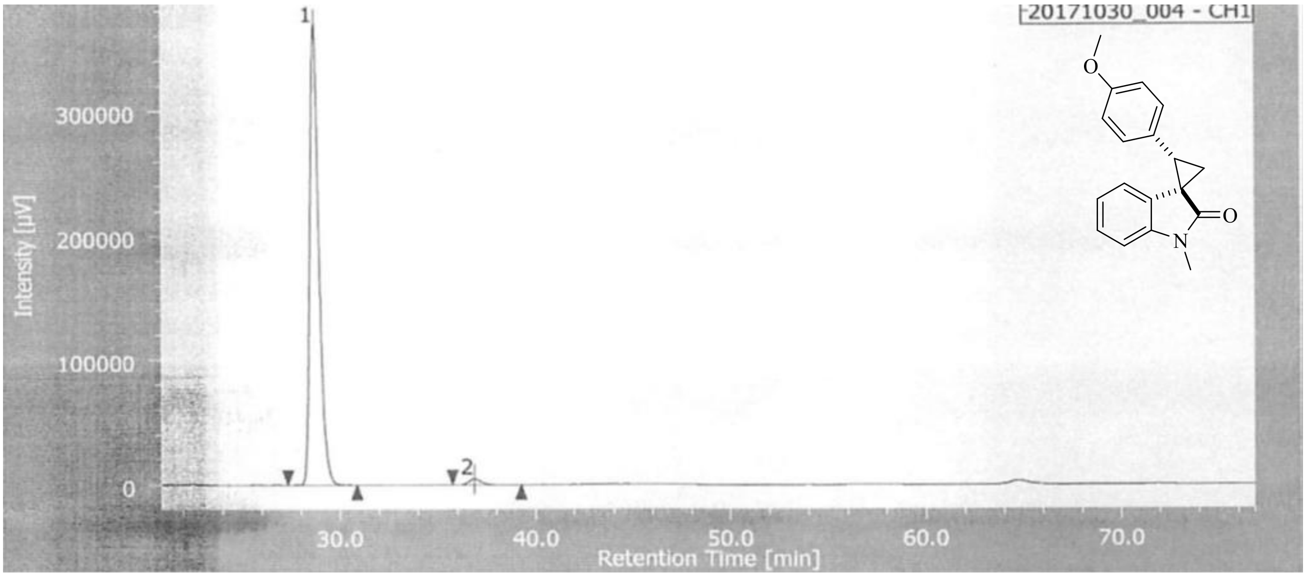

Peak	RT [min]	AREA [µV･sec]	HEIGHT [µV]	AREA %	HEIGHT %
1	28.692	11794216	369688	96.475	97.852
2	36.867	207076	4705	1.694	1.245

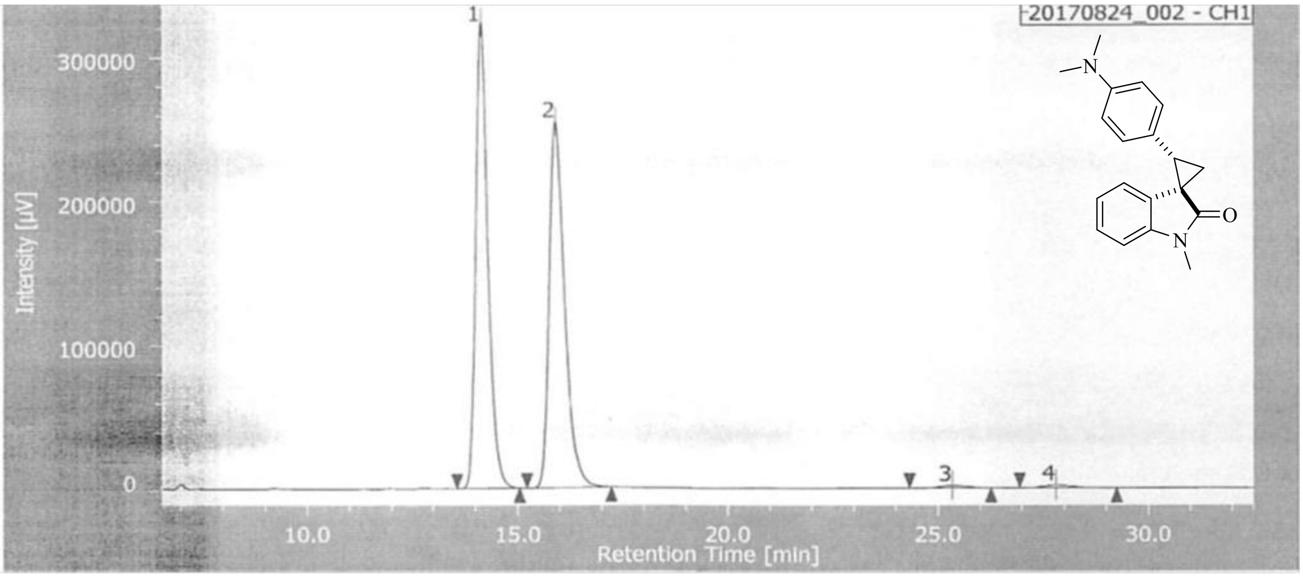

Peak	RT [min]	AREA [μV･sec]	HEIGHT [μV]	AREA %	HEIGHT %
1	14.133	6521324	322765	49.475	55.649
2	15.900	6512801	253566	49.410	43.718
3	25.333	66943	1702	0.508	0.293
4	27.800	80014	1972	0.607	0.340

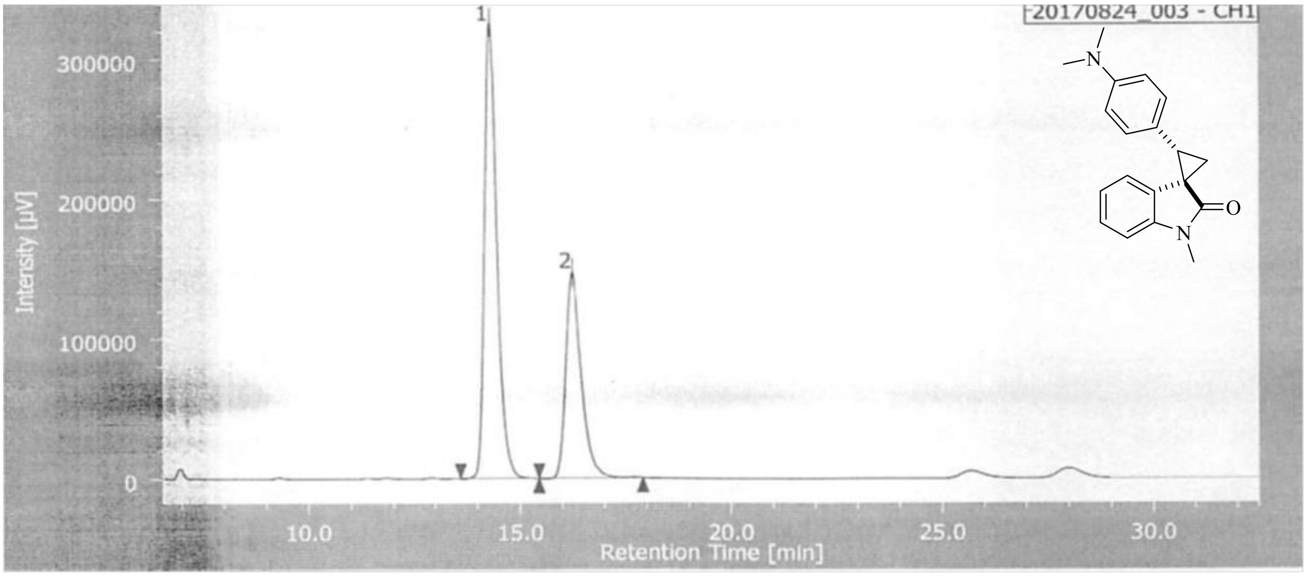

Peak	RT [min]	AREA [μV･sec]	HEIGHT [μV]	AREA %	HEIGHT %
1	14.292	6619517	325812	60.536	67.165
2	16.233	3820478	146781	34.938	30.259

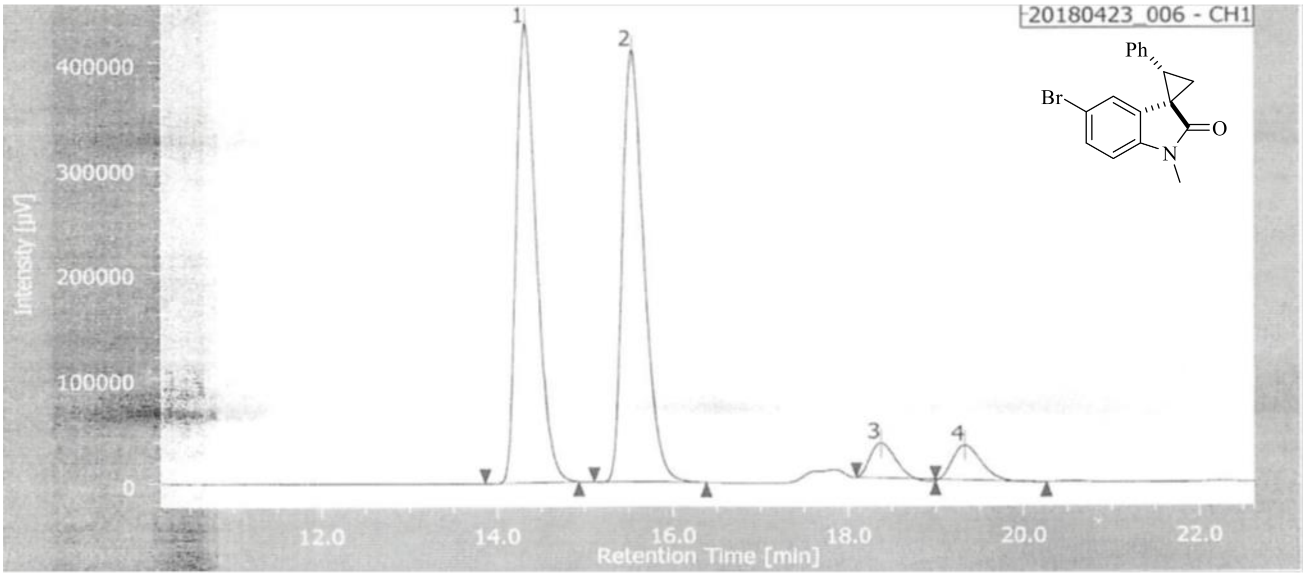

Peak	RT [min]	AREA [µV・sec]	HEIGHT [µV]	AREA %	HEIGHT %
1	15.317	6945968	436478	45.279	47.733
2	15.533	6980843	410403	45.506	44.882
3	18.375	664400	33972	4.331	3.715
4	19.325	749211	33555	4.884	3.670

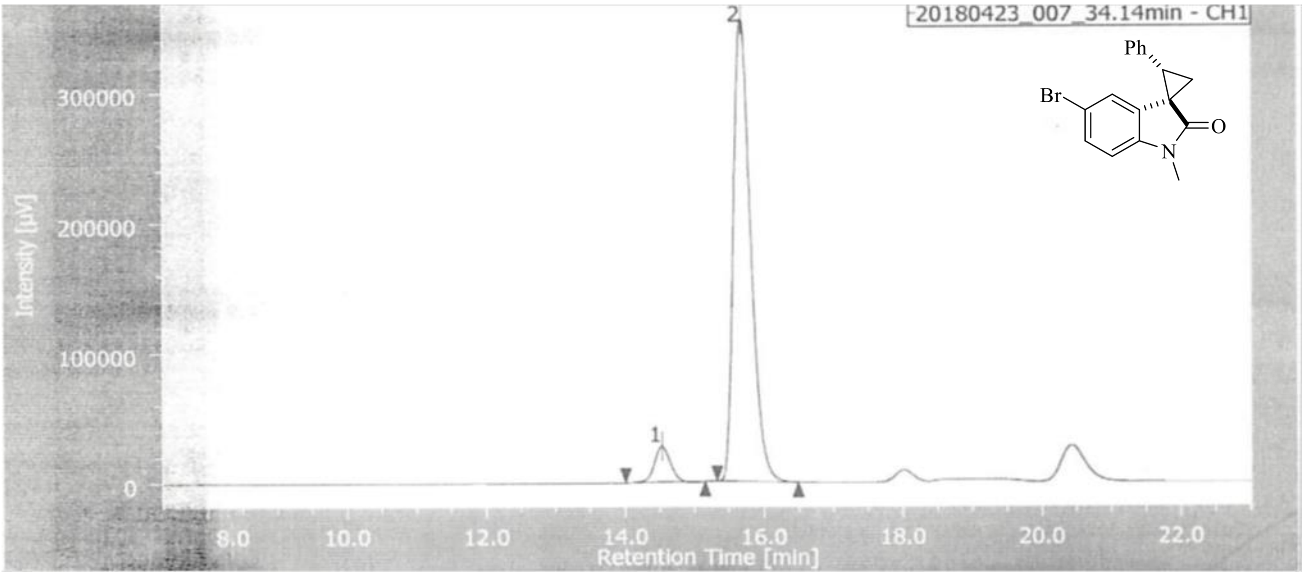

Peak	RT [min]	AREA [µV・sec]	HEIGHT [µV]	AREA %	HEIGHT %
1	14.533	423676	27192	6.569	7.117
2	15.683	6026235	354857	93.431	92.883

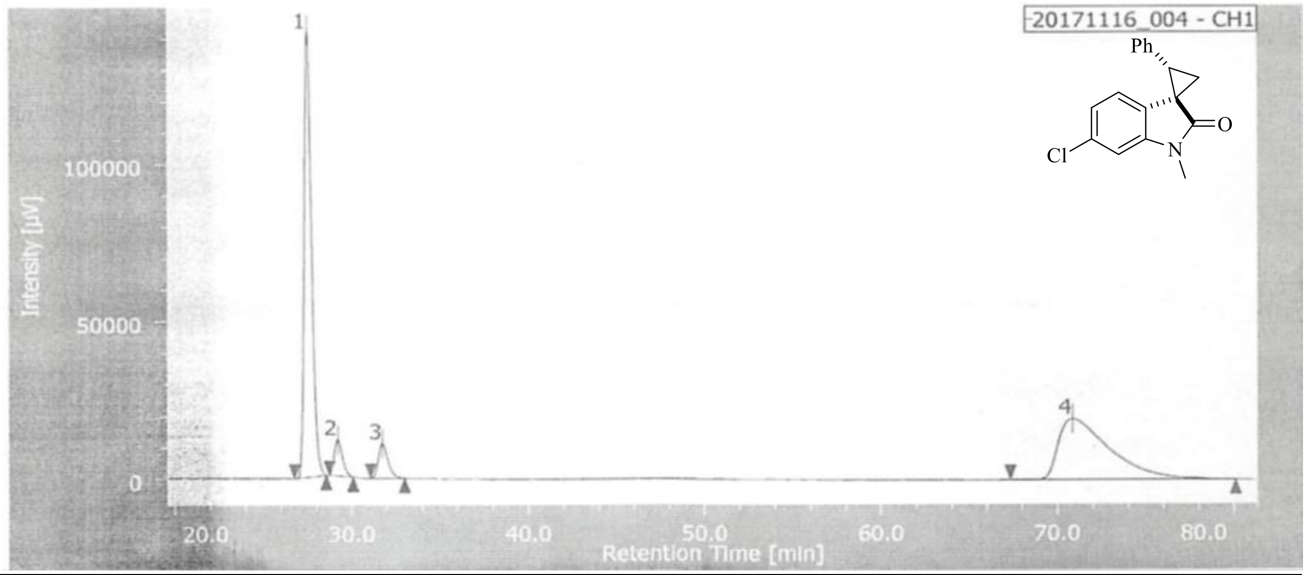

Peak	RT [min]	AREA [μV･sec]	HEIGHT [μV]	AREA %	HEIGHT %
1	27.500	4039549	142111	46.335	77.251
2	29.208	370042	11431	4.244	6.214
3	31.733	414552	11018	4.755	5.959
4	70.833	3894050	19402	44.666	10.547

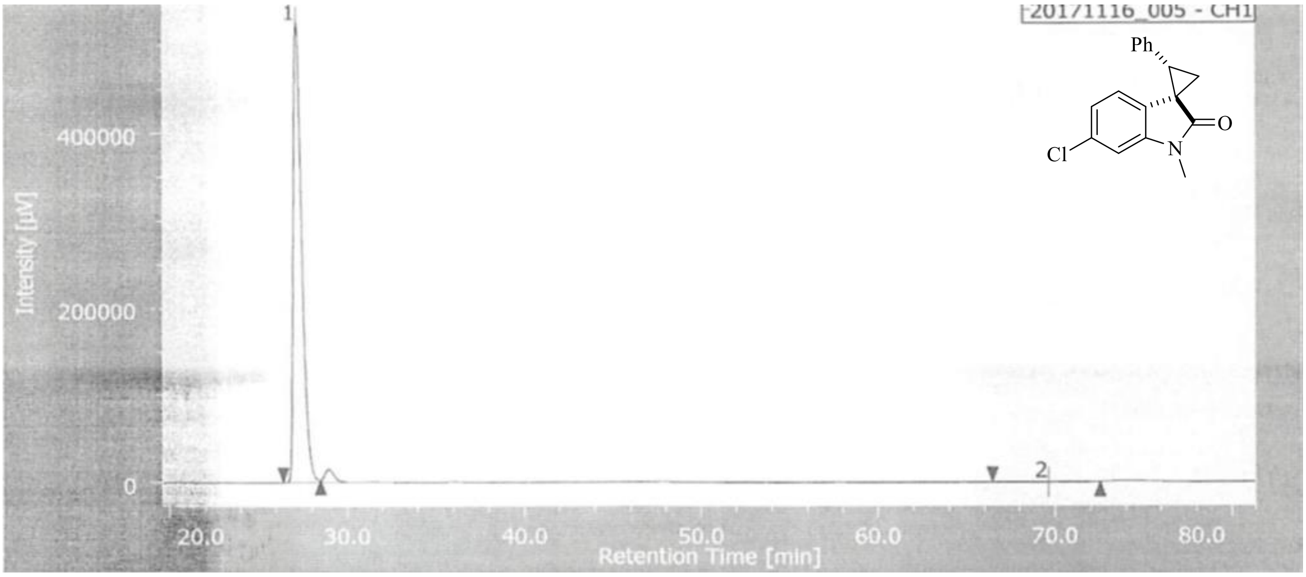

Peak	RT [min]	AREA [μV･sec]	HEIGHT [μV]	AREA %	HEIGHT %
1	27.158	527606	527606	99.707	99.963
2	69.608	196	196	0.293	0.037

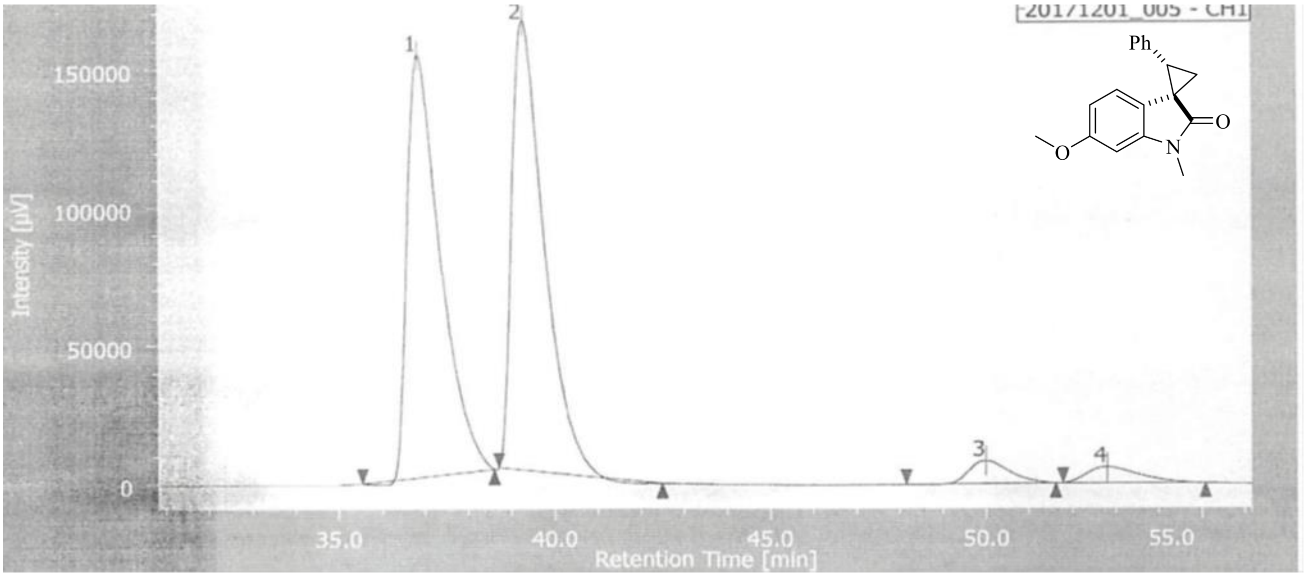

Peak	RT [min]	AREA [µV･sec]	HEIGHT [µV]	AREA %	HEIGHT %
1	36.842	8254408	152673	46.547	46.428
2	39.267	8483279	162189	47.837	49.322
3	49.983	530526	8331	2.992	2.534
4	52.800	465403	5644	2.624	1.716

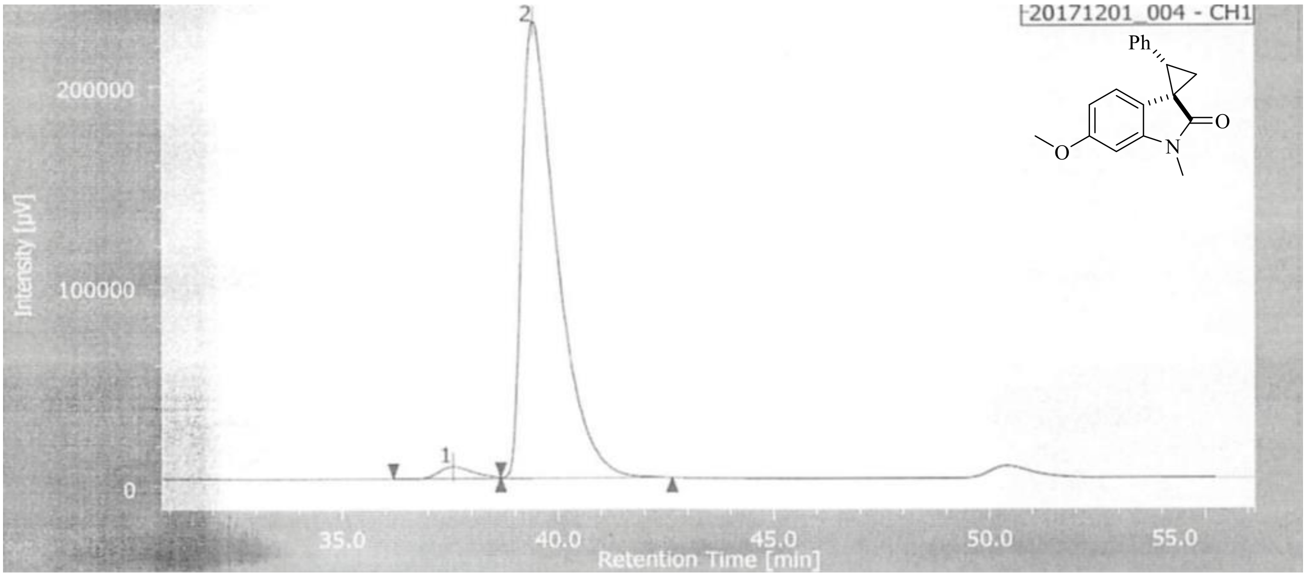

Peak	RT [min]	AREA [µV･sec]	HEIGHT [µV]	AREA %	HEIGHT %
1	37.567	325898	5900	2.469	2.520
2	39.425	12875174	228210	97.531	97.480

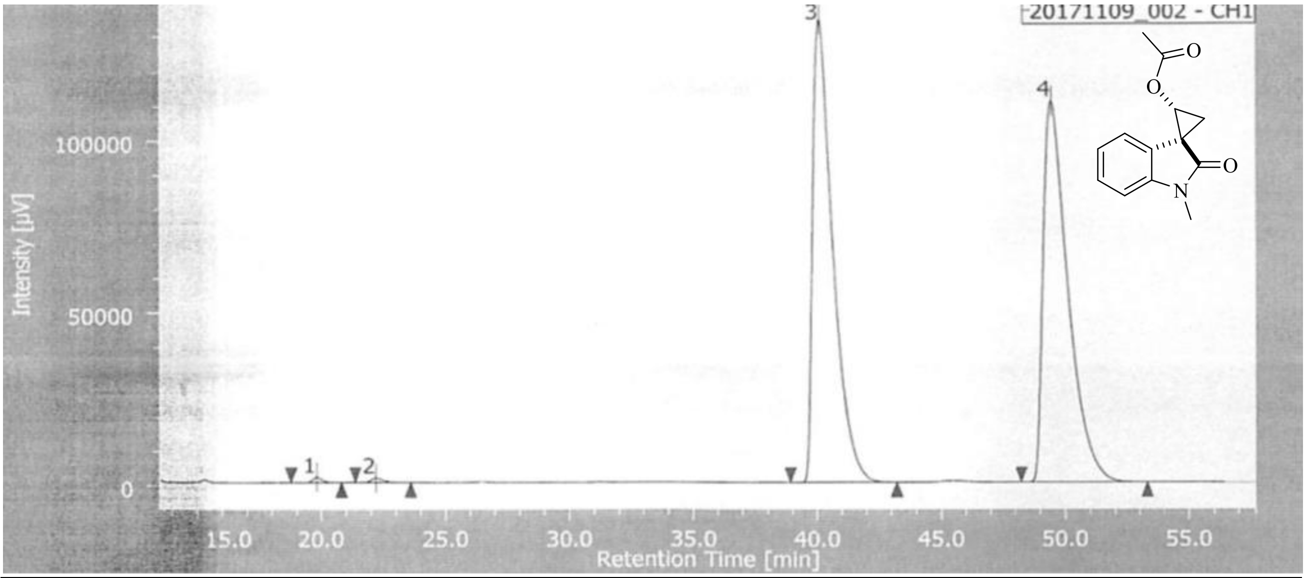

Peak	RT [min]	AREA [µV･sec]	HEIGHT [µV]	AREA %	HEIGHT %
1	19.817	42271	1608	0.278	0.651
2	22.192	42311	1305	0.279	0.529
3	40.075	7512484	133624	49.493	54.145
4	49.433	7581690	110253	49.949	44.675

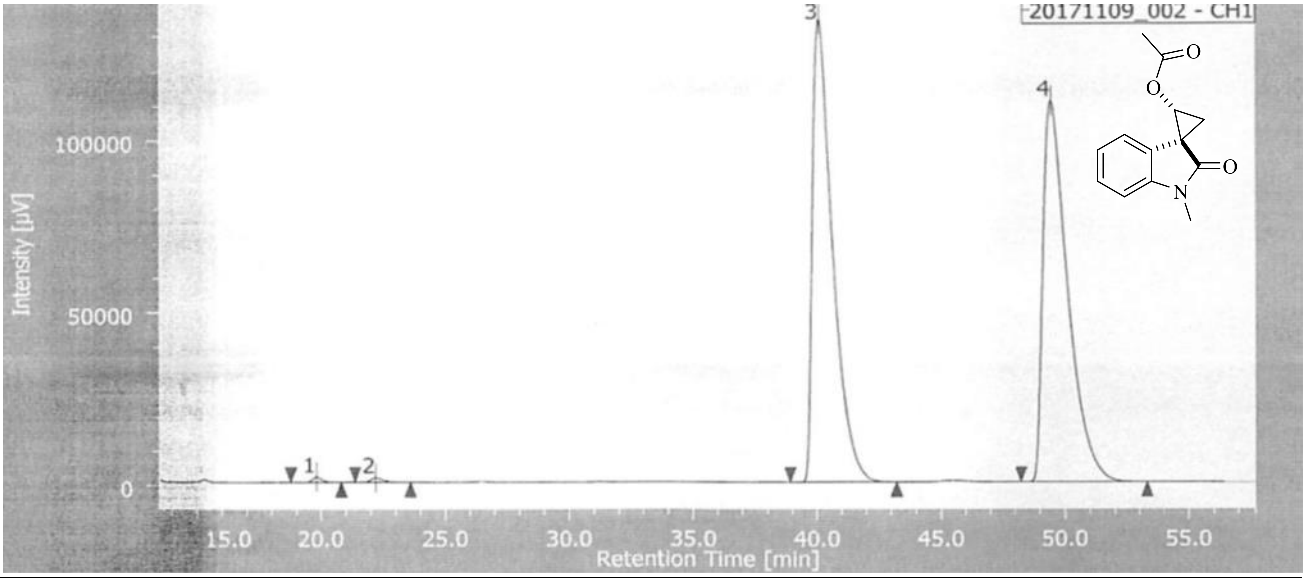

Peak	RT [min]	AREA [µV･sec]	HEIGHT [µV]	AREA %	HEIGHT %
1	40.792	2373971	45717	94.194	93.472
2	50.983	119566	2318	4.744	4.739

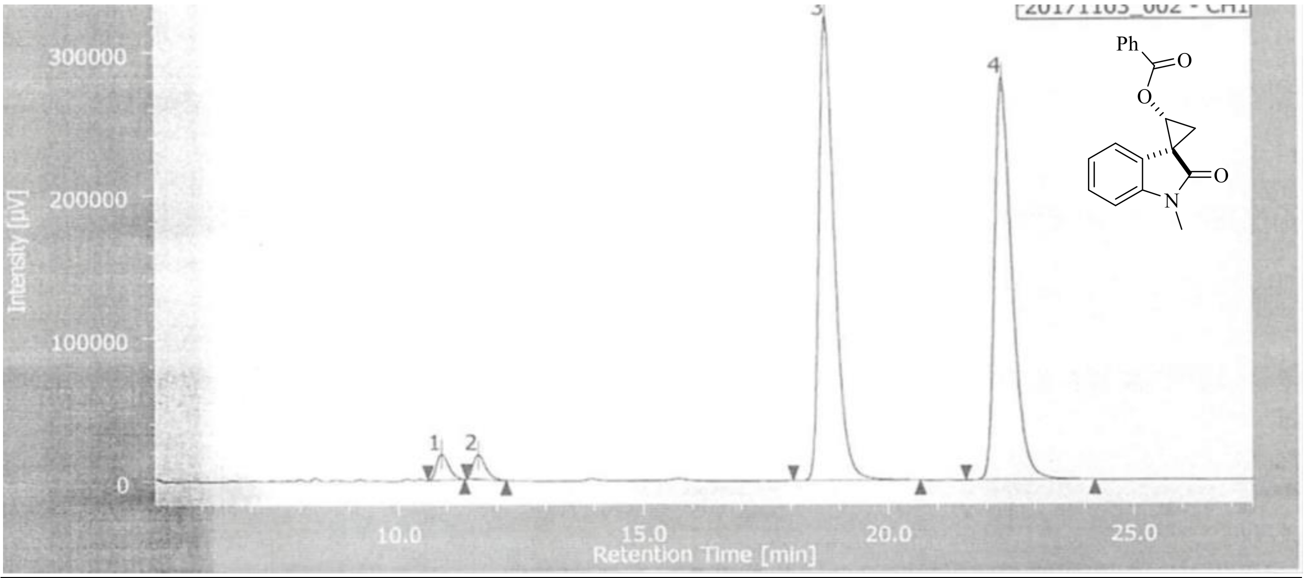

Peak	RT [min]	AREA [µV･sec]	HEIGHT [µV]	AREA %	HEIGHT %
1	10.883	307654	18025	2.062	2.818
2	11.625	305736	17528	2.049	2.741
3	18.725	7143709	323346	47.877	50.556
4	22.325	7163934	280675	48.012	43.885

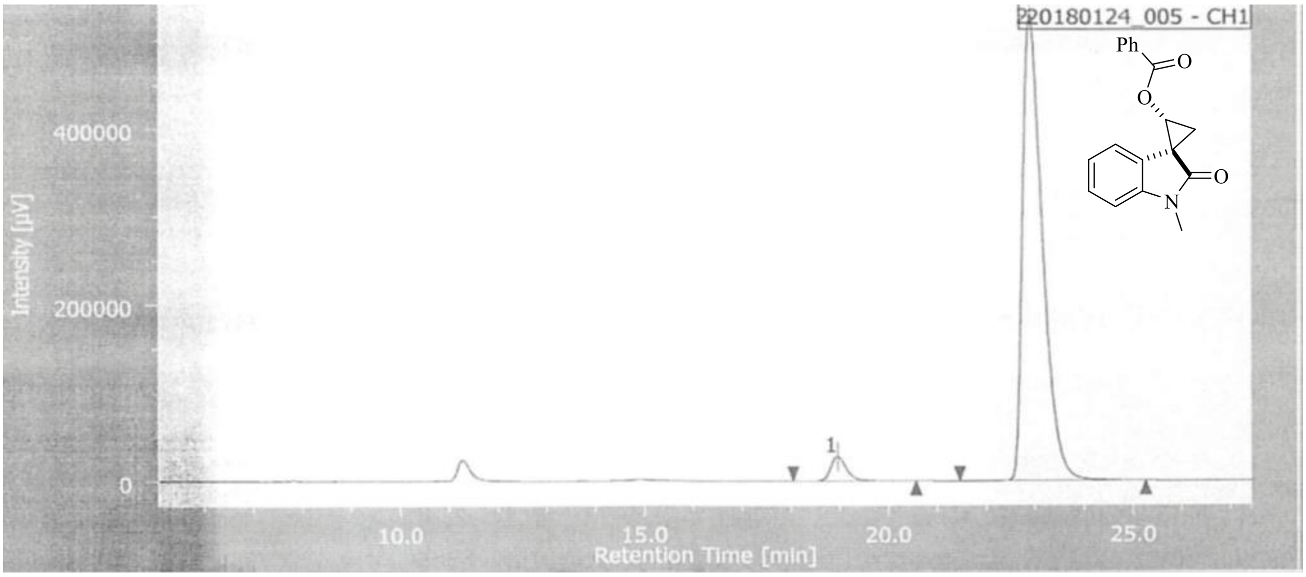

Peak	RT [min]	AREA [µV･sec]	HEIGHT [µV]	AREA %	HEIGHT %
1	18.950	624106	27989	4.025	5.013
2	22.892	14881704	530365	95.975	94.987

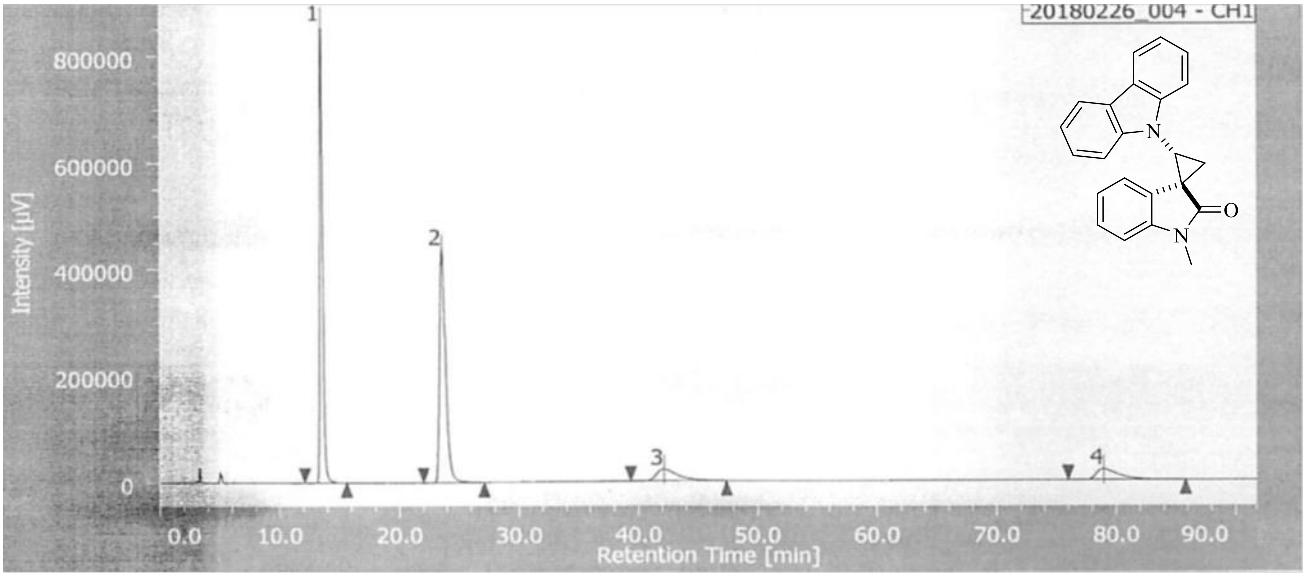

Peak	RT [min]	AREA [μV･sec]	HEIGHT [μV]	AREA %	HEIGHT %
1	13.442	14877777	862754	42.388	64.289
2	23.550	14909543	437127	42.478	32.573
3	42.108	2632031	21689	7.499	1.616
4	78.917	2680000	20425	7.635	1.522

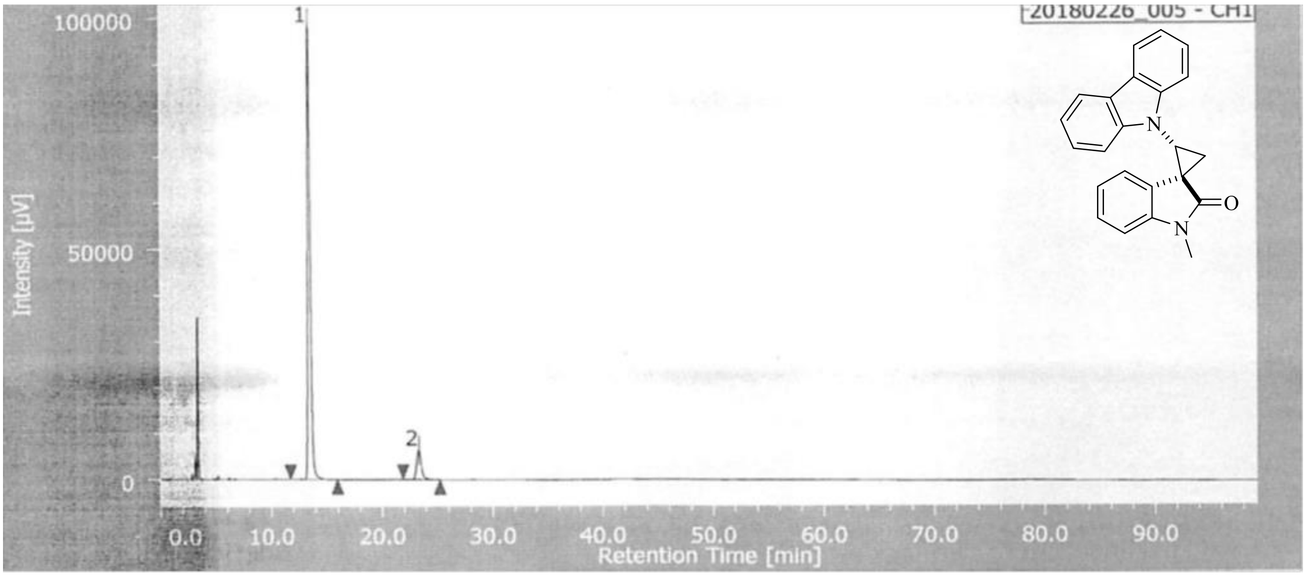

Peak	RT [min]	AREA [μV･sec]	HEIGHT [μV]	AREA %	HEIGHT %
1	13.408	1684704	98705	89.472	93.912
2	23.283	198226	6399	10.528	6.088

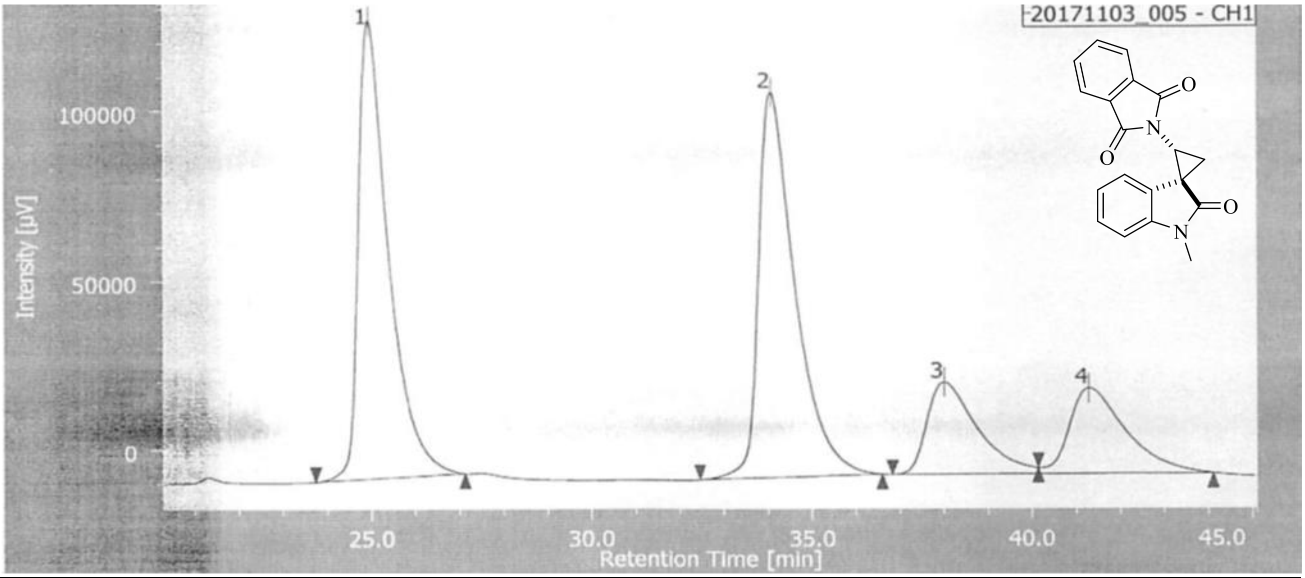

Peak	RT [min]	AREA [µV･sec]	HEIGHT [µV]	AREA %	HEIGHT %
1	24.942	6466531	135159	37.170	44.898
2	34.075	6533111	113479	37.553	37.696
3	37.992	2157434	27186	12.401	9.031
4	41.275	2239925	25213	12.875	8.375

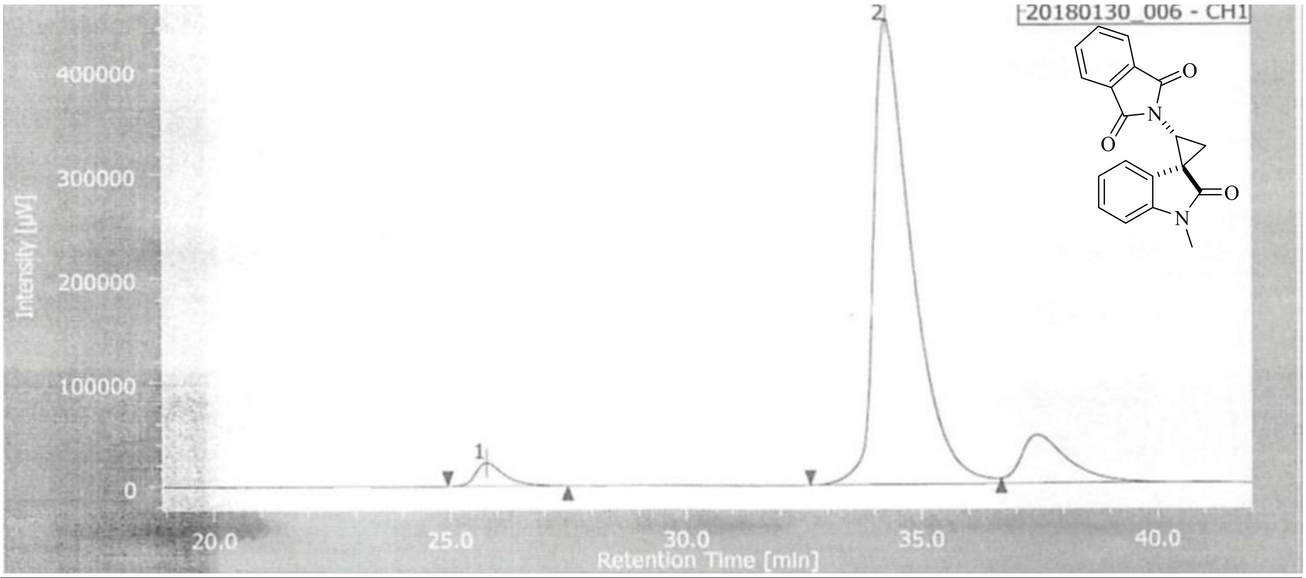

Peak	RT [min]	AREA [µV･sec]	HEIGHT [µV]	AREA %	HEIGHT %
1	25.767	922861	22111	3.566	4.716
2	34.250	24958820	446733	96.434	95.284

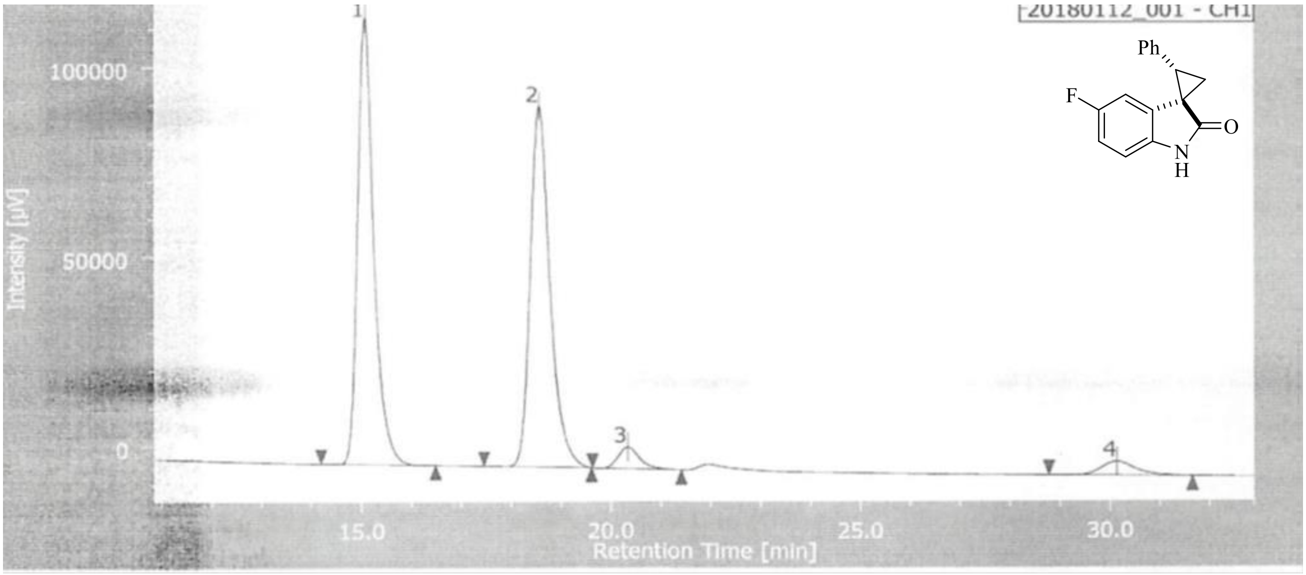

Peak	RT [min]	AREA [μV･sec]	HEIGHT [μV]	AREA %	HEIGHT %
1	15.075	2654746	117569	47.229	52.926
2	18.567	2624663	95140	46.694	42.829
3	20.333	167112	5736	2.973	2.582
4	30.125	174432	3695	3.103	1.663

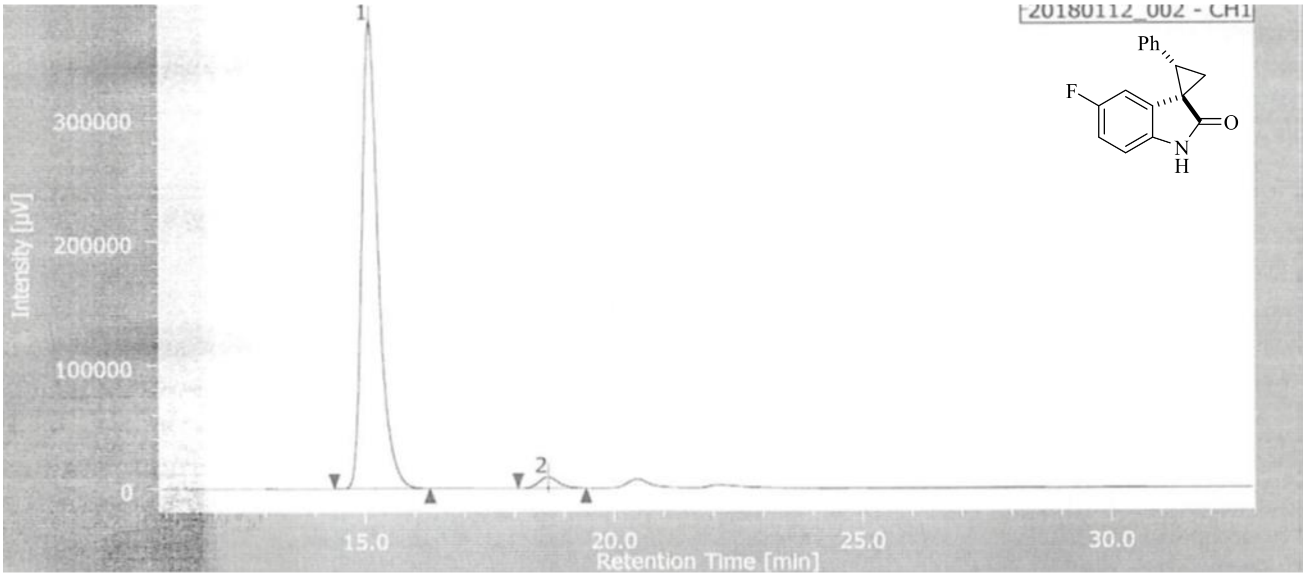

Peak	RT [min]	AREA [μV･sec]	HEIGHT [μV]	AREA %	HEIGHT %
1	15.092	8693295	378838	97.193	97.667
2	18.667	251057	9051	2.807	2.333

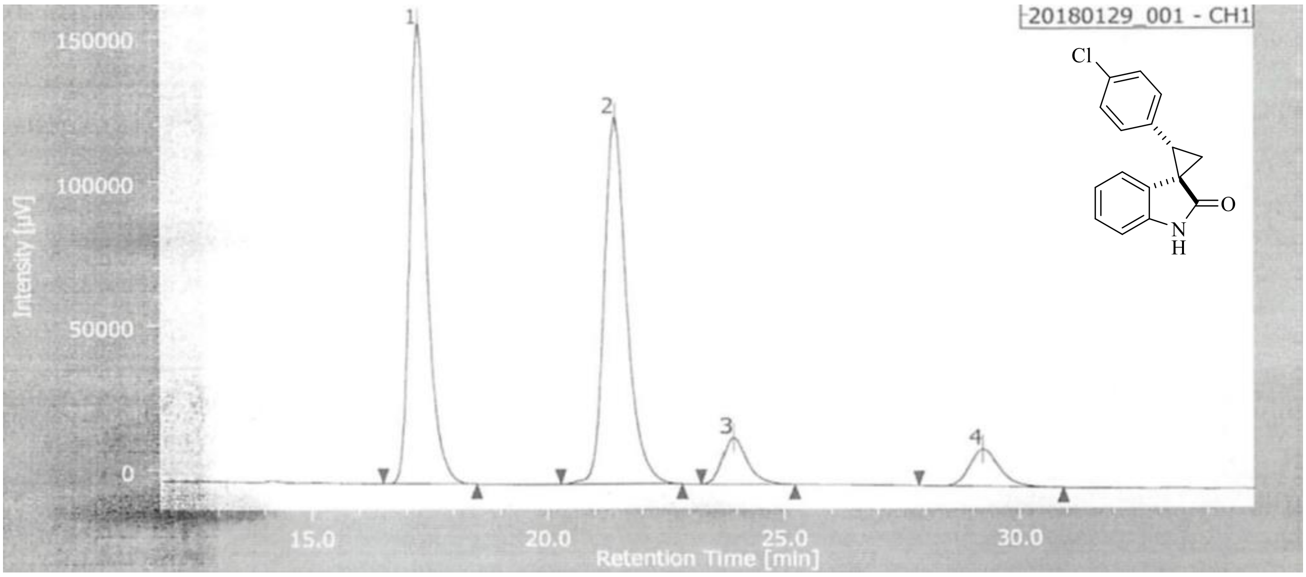

Peak	RT [min]	AREA [µV･sec]	HEIGHT [µV]	AREA %	HEIGHT %
1	17.242	4176783	159534	43.785	50.504
2	21.417	4217474	127255	44.211	40.285
3	23.933	574955	16104	6.027	5.098
4	29.208	570177	12993	5.977	4.113

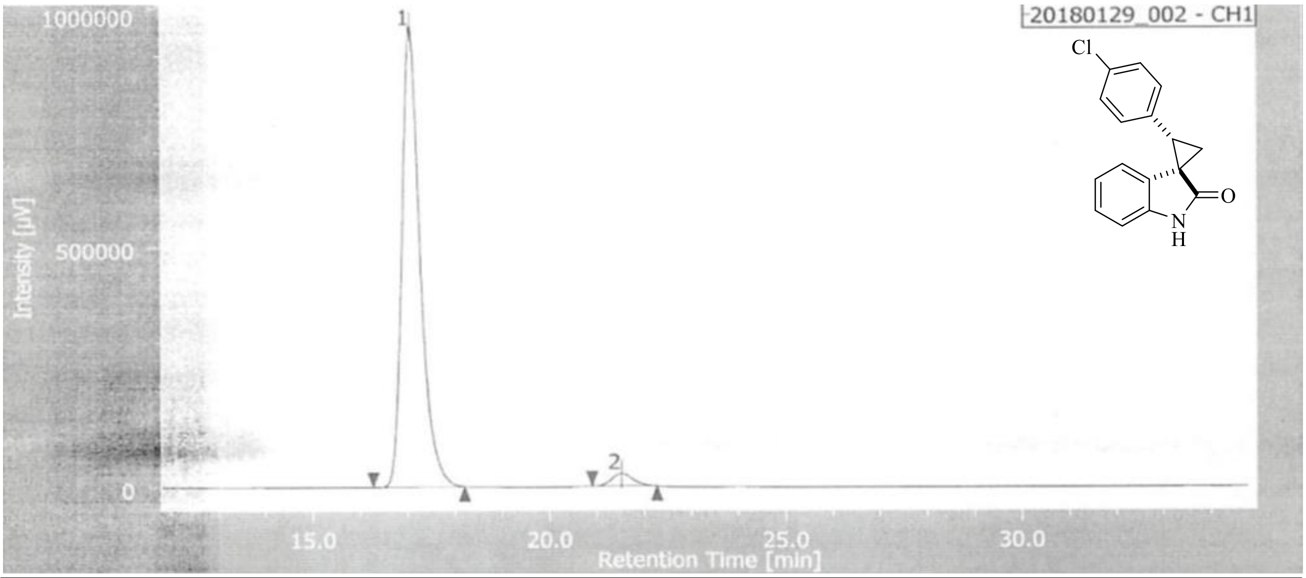

Peak	RT [min]	AREA [µV･sec]	HEIGHT [µV]	AREA %	HEIGHT %
1	17.058	26163934	959277	97.024	97.365
2	21.517	802597	2.976	2.976	2.635

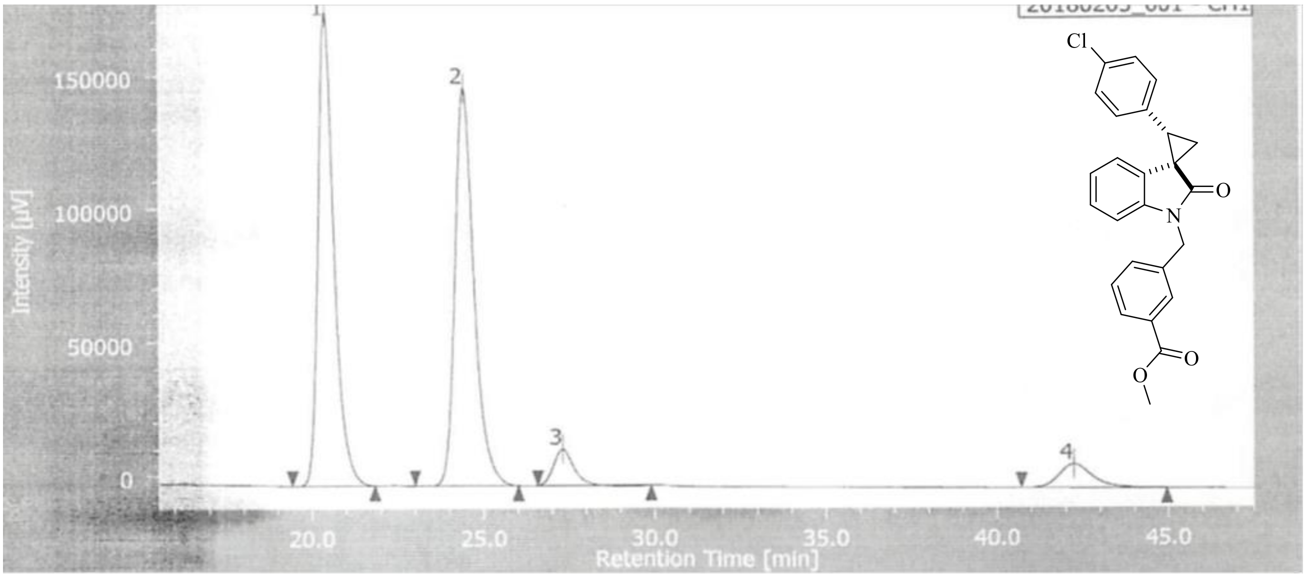

Peak	RT [min]	AREA [µV･sec]	HEIGHT [µV]	AREA %	HEIGHT %
1	20.400	5720761	177189	45.305	50.821
2	24.433	5739725	148882	45.455	42.702
3	27.300	568264	13706	4.500	3.931
4	42.225	598566	8877	4.740	2.546

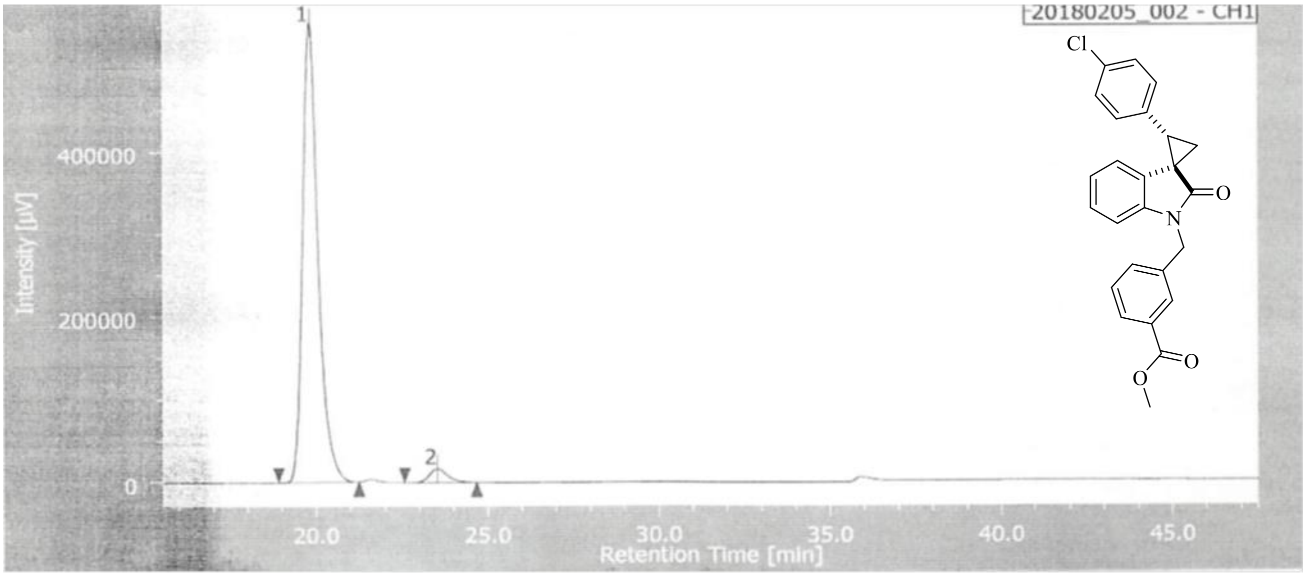

Peak	RT [min]	AREA [µV･sec]	HEIGHT [µV]	AREA %	HEIGHT %
1	19.825	17309097	555362	96.687	97.100
2	23.542	593182	16589	3.313	2.900

8. Calculation Method

To predict the possible conformations of Ru(II)-Pheox **6e**, the CONFLEX conformation search method [1,2] implemented in CONFLEX 8 [3] was used. The M06-2X hybrid functional with the LanL2DZ basis set for Ru and 6-31G(d) for other atoms were chosen to describe the potential energy surface (PES) using Gaussian 16 [4]. CONFLEX 8 can access the PES obtained by the electronic structure calculation directly because the interface to Gaussian (16 or 09) has been implemented.

9. Calculation result

Table S1. Optimization of metal-carbene complex (Ru(II)-Pheox **6e**) in toluene.

No.	Total energy (Hartree)	Relative energy (kcal/mol)
1	-1317.140442	0.00
2	-1317.136508	2.47
3	-1317.136296	2.60
4	-1317.132608	4.92
5	-1317.132045	5.27
6	-1317.127699	8.00
7	-1317.125439	9.41

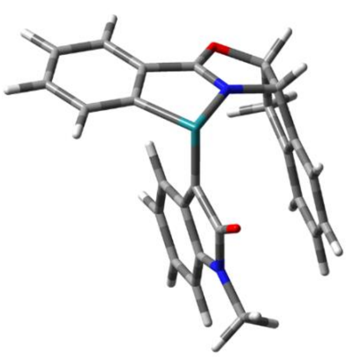
S1

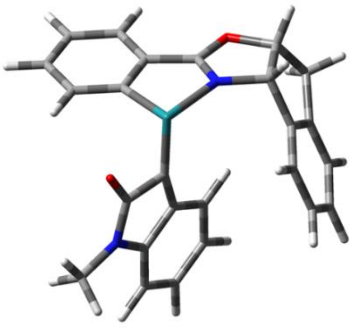
S2

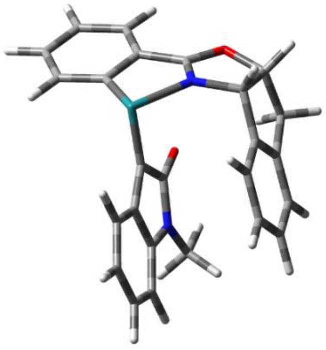
S3

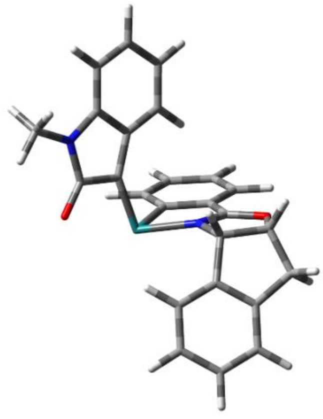

S4

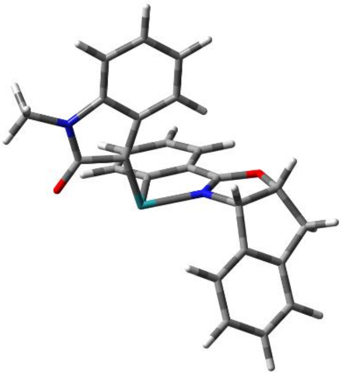

S5

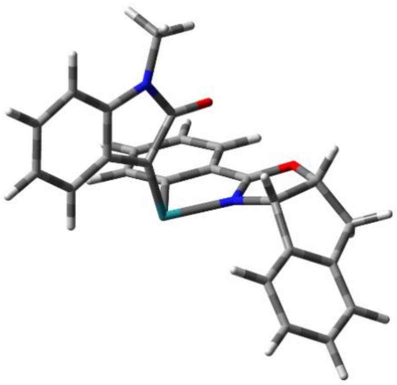

S6

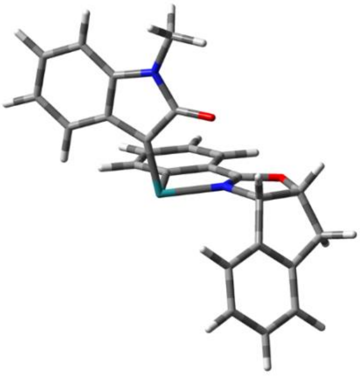

S7

Figure S1. Optimized structures of metal-carbene complex (Ru(II)-Pheox **6e**) in toluene.

10. Plausible Mechanism

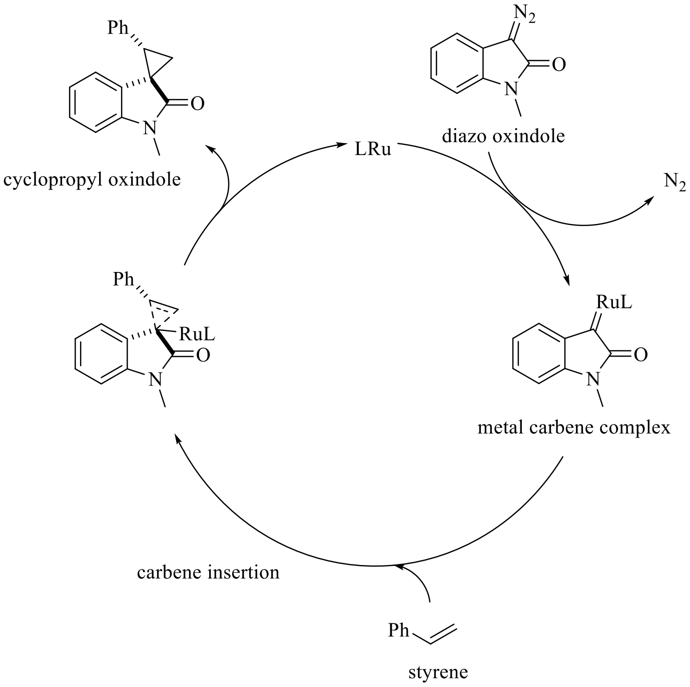

Figure S2. Reaction Mechanism.

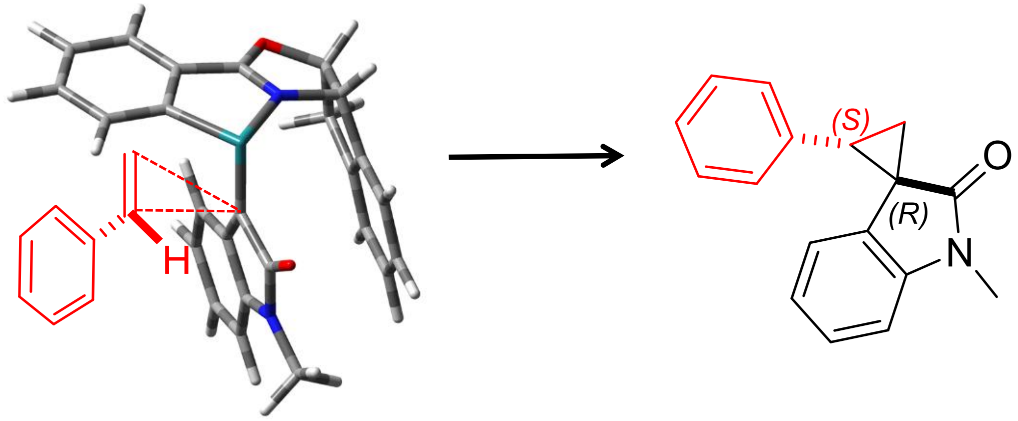

S1 + Styrene

Figure S3. Chiral Induction.

11. Reference

[1] C. Marit and E. M. Carreira, *J. Am. Chem. Soc*., **2005**, *127* (32), 11505.

[2] M. Ošeka, M. Kimm, S. Kaabel, I. Järving, K. Rissanen and T. Kanger, *Org. Lett*., **2016**, *18* (6), 1358.

[3] G. Kaupp and D. Matthies, *Chem. Ber.*, **1987**, *120* (11), 1897.

[4] X. K. Wee, T. Yang and M, L. Go, *ChemMedChem*, **2012**, *7* (5), 777.

[5] H. Ji, Y. Zhu, Y. Shao, J. Liu, Y. Yuan and X. Jia, *J. Org. Chem.*, **2017**, *82* (18), 9859.

[6] J. L. Meloche and B. L. Ashfeld, *Angew. Chem. Int. Ed.*, **2017**, 56 (23), 6604.

[7] G. K. Murphy, F. Z. Abbas and V. Poulton, *Adv. Synth. Catal.*, **2014**, *356*, 2919.

[8] M. Palomba, L. Rossi, L. Sancineto, E. Tramontano, A. Corona, L. Bagnoli, C. Santi, C. Pannecouque, O. Tabarrini and F. Marini, *Org. Biomol. Chem.*, **2016**, 14, 2015.

[9] L. Chen, M. Huang, L. Feng, Y. He and H. Yun, WO2011/069298 A1, 2011
